# Cobalt bis(acetylacetonate)–*tert*-butyl hydroperoxide–triethylsilane: a general reagent combination for the Markovnikov-selective hydrofunctionalization of alkenes by hydrogen atom transfer

**DOI:** 10.3762/bjoc.14.201

**Published:** 2018-08-28

**Authors:** Xiaoshen Ma, Seth B Herzon

**Affiliations:** 1 Department of Chemistry, Yale University, New Haven, Connecticut 06520, United States; 2 Department of Pharmacology, Yale School of Medicine, New Haven, Connecticut 06520, United States

**Keywords:** HAT, hydrogen atom transfer, hydrofunctionalization

## Abstract

We show that cobalt bis(acetylacetonate) [Co(acac)_2_], *tert*-butyl hydroperoxide (TBHP), and triethylsilane (Et_3_SiH) constitute an inexpensive, general, and practical reagent combination to initiate a broad range of Markovnikov-selective alkene hydrofunctionalization reactions. These transformations are believed to proceed by cobalt-mediated hydrogen atom transfer (HAT) to the alkene substrate, followed by interception of the resulting alkyl radical intermediate with a SOMOphile. In addition, we report the first reductive couplings of unactivated alkenes and aryldiazonium salts by an HAT pathway. The simplicity and generality of the Co(acac)_2_–TBHP–Et_3_SiH reagent combination suggests it as a useful starting point to develop HAT reactions in complex settings.

## Introduction

**Scheme 1:**
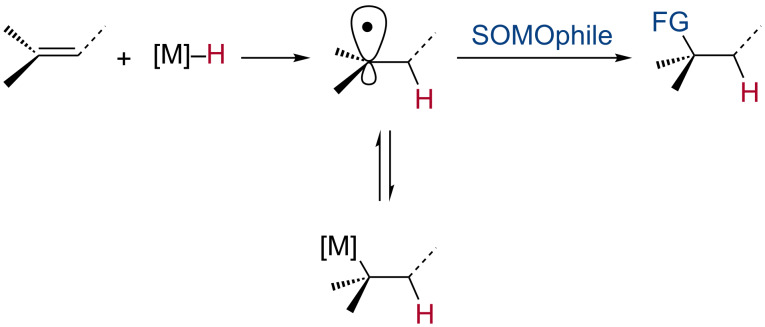
General mechanism of alkene hydrofunctionalization via HAT.

Many powerful methods to effect alkene hydrogenation [1–4] and Markovnikov-selective hydroheterofunctionalization (H–X addition, X = O [5–9], I [3], Br [3], Se [3], S [8–10], Cl [8,11], F [12–13], and N [8,14–17]) by metal-mediated hydrogen atom transfer (HAT) [18–21] are now known. Additionally, methods to achieve carbon–carbon bond formation to alkenes by HAT have been developed (e.g., reductive coupling [22–28], formal hydromethylation [29], cycloisomerization [8,30–31], hydrooximation [32], hydroheteroarylation [28,33–35], hydroarylation [36–38], and cross-coupling [37]). Many of these transformations have found applications in synthesis [6,39–47]. Although mechanistically-complex [28] the outcome of these reactions can be rationalized as initiating by HAT to the alkene, to form the kinetically- and thermodynamically-favored alkyl radical intermediate, which may be in equilibrium with the corresponding metal alkyl complex. This radical then undergoes addition to a second reagent (SOMOphile) to form the functionalized product (Scheme 1).

A wide range of manganese, cobalt, or iron-based complexes containing diverse supporting ligands have found use in these reactions. To the best of our knowledge, the iron oxalate–sodium borohydride system, introduced by Boger and co-workers [8], is the only reagent combination shown to accommodate a broad range of SOMOphiles. However, the cobalt–salen complexes that are commonly employed [10–11,13,15–16,30–32,36–37,48] contain many different ligand architectures [21], and often need to be prepared by multistep sequences. Here we report a uniform set of reaction conditions to achieve a broad range of HAT hydrofunctionalization reactions using the simple reagents cobalt acetoacetonate [Co(acac)_2_], *tert*-butyl hydroperoxide (TBHP), and triethylsilane (Et_3_SiH). The practicality and generality of this system should motivate its application in synthesis.

## Results and Discussion

**Scheme 2:**
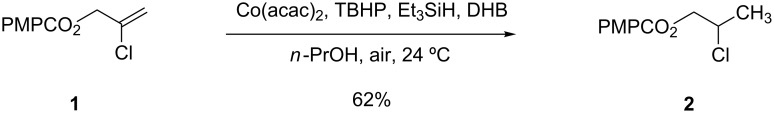
Reduction of the alkenyl chloride **1** by HAT.

In 2014, we reported the reduction of alkenyl halides (e.g., **1**, Scheme 2) utilizing Co(acac)_2_, TBHP, and two reductants, triethylsilane and 1,4-dihydrobenzene (DHB) [2]. Mechanistic studies showed that Et_3_SiH participates in the formation of a cobalt hydride intermediate that delivers a hydrogen atom to the less-substituted position of the alkene. The resulting alkyl radical is believed to abstract a second hydrogen atom from DHB to generate the reduced product [2]. This mechanism separates the alkyl radical formation and functionalization steps by employing two different reagents. Accordingly, we investigated the application of this system in other HAT reactions. In these studies, methallyl *p*-methoxybenzoate (**3a**) was used as substrate (Table 1).

**Table 1:** Markovnikov Hydrofunctionalization of **3a**.^a^

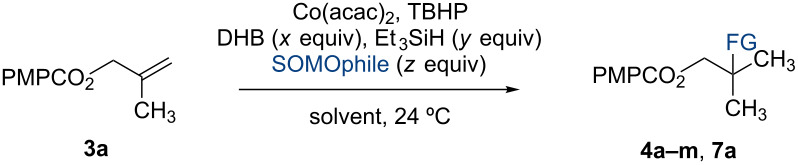							

entry	x	y	SOMOphile	z	solvent	product	yield^b^

1	5.00	5.00	–	–	*n*-propanol	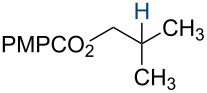 **4a**	86%
2	2.50	10.0	NFSI	2.50	CH_2_Cl_2_	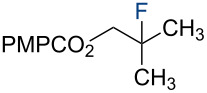 **4b**	36%
3	2.50	10.0	TsCl	2.50	*n*-propanol	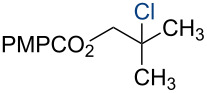 **4c**	92%
4	3.75	10.0	TsBr	2.50	*n*-propanol	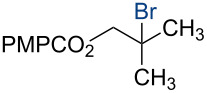 **4d**	95%
5	3.75	10.0	CH_2_I_2_	15.0	CH_2_Cl_2_	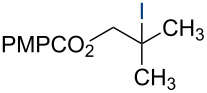 **4e**	89%
6	10.0	10.0	O_2_	–	*n*-propanol	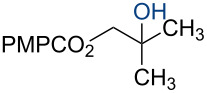 **4f**	69%
7	2.50	10.0	PhSO_2_SPh	2.50	*n*-propanol	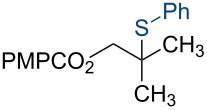 **4g**	96%
8	2.50	10.0	TsSePh	2.50	*n*-propanol	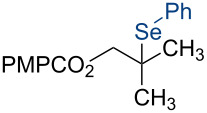 **4h**	89%
9	1.00	10.0	*p-*ABSA	5.00	CH_3_CN	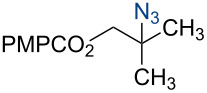 **4i**	79%
10	0	6.25	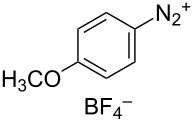 **5a**	1.50	CH_2_Cl_2_	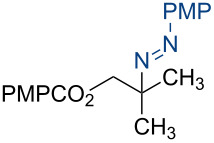 **7a**	92%
11	3.75	10.0	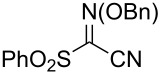 **6a**	2.50	*n*-propanol	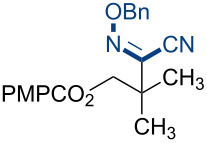 **4j**	60%
12	3.75	10.0	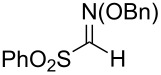 **6b**	2.50	*n*-propanol	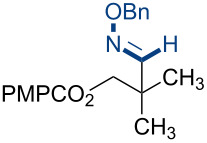 **4k**	48%
13	0	5.00	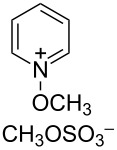 **6c**	5.00	CH_2_Cl_2_	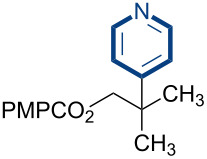 **4l**	66% 4.7:1 rr^b^
14	0	5.00	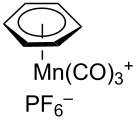 **6d**	1.00	CH_3_CN	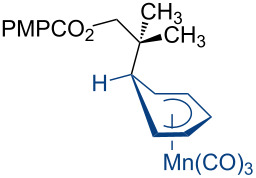 **4m**	50%

^a^For detailed reaction conditions, see Supporting Information File 1. ^b^Isolated yields after purification by flash-column chromatography. ^b^rr = ratio of regioisomers.

Under our standard reduction conditions, alkene **3a** was transformed to isobutyl *p*-methoxybenzoate (**4a**) in 86% yield (entry 1, Table 1). Inspired by the methods of Boger [12] and Hiroya [13], a number of fluorination reagents were examined to achieve hydrofluorination. Although no product was observed using SelectFluor^®^, diethylaminosulfur trifluoride (DAST), or tosyl fluoride (see Supporting Information File 1, Table S1, entries 2–4), *N*-fluorobenzenesulfonimide (NFSI) provided the desired hydrofluorination product **4b** in 36% yield (Table 1, entry 2). Carreira and co-workers reported the first hydrochlorination reaction via a cobalt-catalyzed HAT process [11]. By utilizing *p*-toluenesulfonyl chloride (TsCl) and *p-*toluenesulfonyl bromide (TsBr) under our conditions, the desired hydrochlorination and hydrobromination products **4c** and **4d** were obtained in 92% and 95% yields, respectively [3] (Table 1, entries 3 and 4). To our knowledge, the formation of **4d** represents the first Markovnikov-selective alkene hydrobromination by an HAT pathway. Attempts to extend this reaction to hydroiodination using related reagents, *p*-toluenesulfonyl iodide, *N*-iodosuccinimide, or molecular iodine failed to provide the expected product (see Supporting Information File 1, Table S1, entries 9–11) [3]. Surprisingly, diiodomethane, possessed the desired reactivity and the hydroiodination product **4e** was isolated in 89% yield (Table 1, entry 5) [3]. Ethyl iodoacetate, iodoacetonitrile, and 1,2-diiodoethane were also effective, but the yields of **4e** and conversion of **3a** were lower (see Supporting Information File 1, Table S1, entries 13–15). Mukaiyama and co-workers’ pioneering Markonikov-selective alkene hydration reaction [5,49–54] proceeds using dioxygen as the oxygen atom source. Exposure of the alkene **3a** to similar conditions provided the tertiary alcohol **4f** in 69% yield (Table 1, entry 6). Inspired by Girijavallabhan and co-workers’ report [10], we were able to trap the tertiary alkyl radical with *S*-phenyl benzene thiosulfonate (PhSO_2_SPh, Table 1, entry 7) and *Se*-phenyl 4-methylbenzenesulfonoselenoate (TsSePh, Table 1, entry 8) [3] to afford the corresponding products **4g** and **4h** in 96% and 89% yields, respectively. Carreira and co-workers reported the hydroazidation of alkenes using cobalt–salen complexes as hydrogen atom transfer agents and *para*-toluenesulfonyl azide as an azide source [16,48,55]. After careful optimization of the azidation reagent (*p*-acetamidobenzenesulfonyl azide (*p-*ABSA)) and additive equivalents (see Supporting Information File 1, Table S1, entries 19–28), the tertiary alkyl azide **4i** was obtained in 79% yield (Table 1, entry 9).

**Scheme 3:**
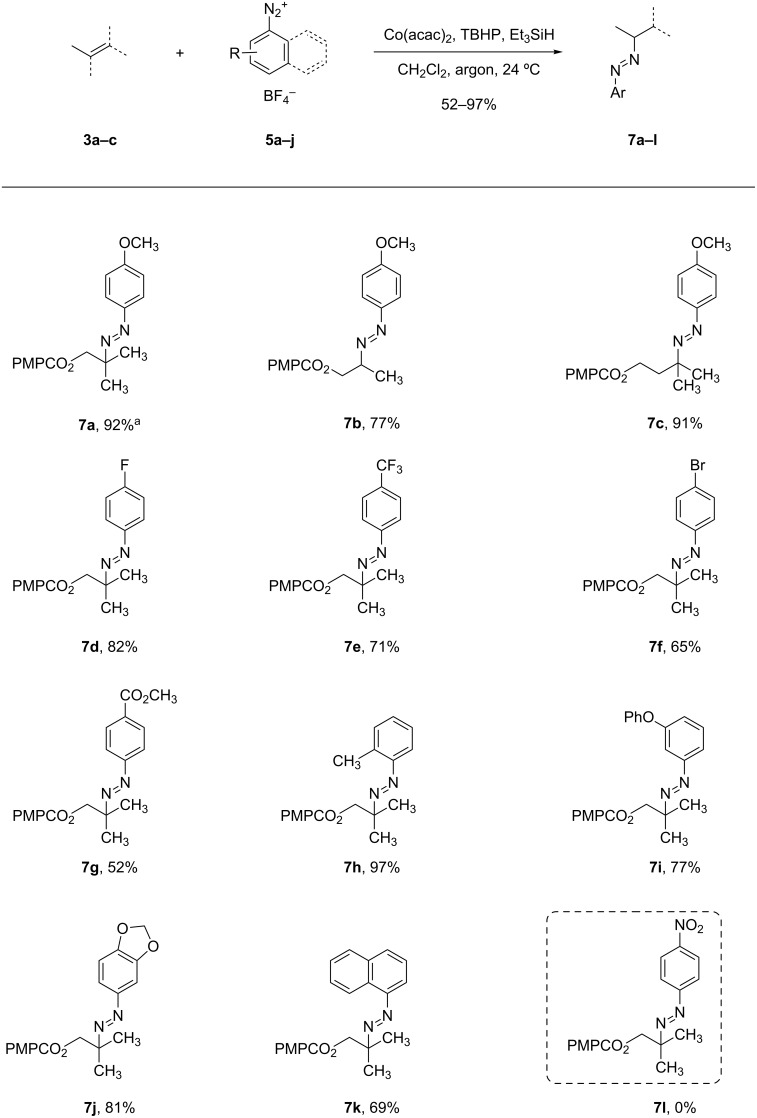
Substrate scope of alkyl-aryl azo compound synthesis via HAT. Conditions: alkene (0.250 mmol), diazonium salt (1.50 equiv), Co(acac)_2_ (1.00 equiv), TBHP (1.00 equiv), Et_3_SiH (6.25 equiv), CH_2_Cl_2_ (0.2 M), argon, 15–120 min. All yields are isolated yields after flash-column chromatography. ^a^Reaction conducted on 1.00 mmol scale.

To broaden the scope of C–N coupling process via HAT, we investigated other nitrogen-containing SOMOphiles in the HAT reaction. Employing 4-methoxyphenyldiazonium tetrafluoroborate (**5a**) in our HAT conditions, the alkyl aryl azo product **7a** was obtained in 92% yield (Table 1, entry 10). Due to the importance of azo compounds in synthetic organic chemistry, industrial dyes, and medicinal chemistry [56–57], we investigated the scope of this transformation (Scheme 3). By varying the alkene substitution pattern, we determined that the coupling of tertiary radicals is more efficient than secondary radicals. For example, methallyl *p*-methoxybenzoate (**3a**) and prenyl *p*-methoxybenzoate (**3c**, not shown) afforded the azo compounds **7a** and **7c** in 92% and 91% yields, respectively. By comparison the yield of the azo product using allyl *p*-methoxybenzoate (**3b**, now shown) as substrate was somewhat lower (77%). Diazonium salts bearing substituents with different steric and electronic properties were examined. These experiments revealed that this coupling is compatible with a broad range of functional groups and aryl substitution patterns (**7d–j**, 52–97%). The naphthylazo derivative **7k** was obtained in 69% yield using 1-naphthyldiazonium tetrafluoroborate. However, aryldiazonium salts bearing nitro substituents were not compatible with this HAT coupling. For example, the use of *p*-nitrobenzendiazonium tetrafluoroborate failed to provide the expected coupling product **7l**. This may be due to alternative reaction pathways involving reduction of the nitro substituent [17].

Additional carbon–carbon bond formation strategies were also examined using the Co(acac)_2_ HAT system. Sulfonyl oximes **6a** and **6b** [32] afforded the carbon–carbon coupled products **4j** and **4k** in 60% and 48% yields, respectively (Table 1, entries 11 and 12, respectively). Recently, our laboratory reported a formal intermolecular hydroheteroarylation using *N-*methoxy heteroarenium salts by Co(acac)_2_-mediated HAT [33–34]. In the original reports, 36 discrete unactivated alkenes were coupled with 38 different heteroarenium salts under mild conditions [33–34]. A representative example comprises the coupling of methallyl *p*-methoxybenzoate (**3a**) with *N*-methoxypyridinium methyl sulfate (**6c**) to form the hydropyridylation product **4l** in 66% yield and as a 4.7:1 ratio of regioisomers (Table 1, entry 13). We also demonstrated that the alkyl radical generated from the HAT process can be trapped by (η^6^-benzene)manganese tricarbonyl hexafluorophosphate (**6d**) to provide the reductive coupling product **4m** in 50% yield (Table 1, entry 14).

## Conclusion

In summary, we have demonstrated that under a consistent set of conditions, the Co(acac)_2_–TBHP–Et_3_SiH system effects a diverse array of Markovnikov-selective hydrofunctionalization reactions of unactivated alkenes (H–X addition, X = H, F, Cl, Br, I, O, S, Se, N, and C). We have also reported the first reductive coupling reactions of alkenes and aryldiazonium salts under HAT conditions. These transformations proceed in high regioselectivity and efficiency. Further efforts will focus on expanding the alkene scope and exploring the site-selectivity in polyene substrates.

## Supporting Information

File 1 Detailed experimental procedures and characterization data for all new compounds.
