# Supplementary material for: Cobalt bis(acetylacetonate)–tert-butyl hydroperoxide–triethylsilane: a general reagent combination for the Markovnikov-selective hydrofunctionalization of alkenes by hydrogen atom transfer
Source: Beilstein J Org Chem. 2018 Aug 28;14:2259–65. doi: 10.3762/bjoc.14.201 (PMC6122341; doi:10.3762/bjoc.14.201)
Supplement: File 1 — Detailed experimental procedures and characterization data for all new compounds. [file Beilstein_J_Org_Chem-14-2259-s001.pdf]

Supporting Information

for

Cobalt bis(acetylacetonate)–*tert*-butyl hydroperoxide–triethylsilane: a general reagent combination for the Markovnikov-selective hydrofunctionalization of alkenes by hydrogen atom transfer

Xiaoshen Ma[1] and Seth B. Herzon*[1,2]

Address: [1]Department of Chemistry, Yale University, New Haven, Connecticut 06520, United States and [2]Department of Pharmacology, Yale School of Medicine, New Haven, Connecticut 06520, United States

Email: Seth B. Herzon - seth.herzon@yale.edu

* Corresponding author

Detailed experimental procedures and characterization data for all new compounds

Index

Table S1..........S2
General experimental procedures..........S3
Materials..........S3
Instrumentation..........S3
Synthetic procedures..........S5
Catalog of nuclear magnetic resonance and infrared spectra of purified products..........S49
Bibliography..........S141

Table S1. Condition optimization of the Markovnikov-selective hydrofunctionalization.

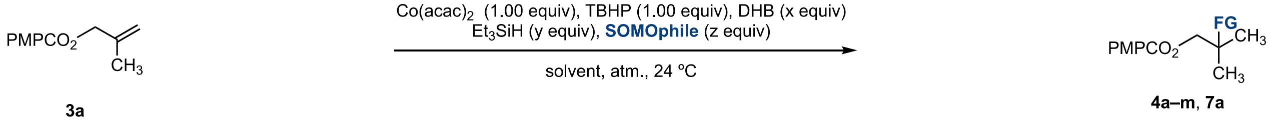

entry	x (equiv)	y (equiv)	SOMOphile	z (equiv)	solvent	atm.	product	conv.	yield
1	5.00	5.00	–	–	*n*-propanol	air	**4a**	>95%	86%
2	5.00	5.00	SelectFluor®	5.00	*n*-propanol	argon	**4b**	<5%	<5%
3	5.00	5.00	DAST	5.00	*n*-propanol	argon	**4b**	<5%	<5%
4	5.00	5.00	TsF	5.00	*n*-propanol	argon	**4b**	<5%	<5%
5	5.00	5.00	NFSI	5.00	*n*-propanol	argon	**4b**	60%	22%
6	2.50	10.0	NFSI	2.50	CH_2Cl_2	argon	**4b**	71%	36%
7	2.50	10.0	TsCl	2.50	*n*-propanol	argon	**4c**	>95%	92%
8	3.75	10.0	TsBr	2.50	*n*-propanol	argon	**4d**	>95%	95%
9	3.75	10.0	TsI	5.00	CH_2Cl_2	argon	**4e**	<5%	<5%
10	3.75	10.0	NIS	5.00	CH_2Cl_2	argon	**4e**	<5%	<5%
11	3.75	10.0	I_2	5.00	CH_2Cl_2	argon	**4e**	<5%	<5%
12	3.75	10.0	CH_2I_2	15.0	CH_2Cl_2	argon	**4e**	>95%	89%
13	3.75	10.0	ICH_2CO_2Et	15.0	CH_2Cl_2	argon	**4e**	51%	49%
14	3.75	10.0	ICH_2CN	15.0	CH_2Cl_2	argon	**4e**	44%	36%
15	3.75	10.0	$(CH_2I)_2$	15.0	CH_2Cl_2	argon	**4e**	<5%	<5%
16	10.0	10.0	O_2	–	*n*-propanol	O_2	**4f**	>95%	69%
17	2.50	10.0	$PhSO_2SPh$	2.50	*n*-propanol	argon	**4g**	>95%	96%
18	2.50	10.0	TsSePh	2.50	*n*-propanol	argon	**4h**	>95%	89%
19	5.00	10.0	*p*-ABSA	2.50	*n*-propanol	argon	**4i**	>95%	36%
20	5.00	10.0	DPPA	2.50	*n*-propanol	argon	**4i**	55%	<5%
21	5.00	10.0	*p*-ABSA	5.00	*n*-propanol	argon	**4i**	>95%	52%
22	5.00	10.0	*p*-ABSA	7.50	*n*-propanol	argon	**4i**	>95%	57%
23	5.00	10.0	*p*-ABSA	5.00	CH_2Cl_2	argon	**4i**	79%	44%
24	5.00	10.0	*p*-ABSA	5.00	THF	argon	**4i**	>95%	62%
25	5.00	10.0	*p*-ABSA	5.00	CH_3CN	argon	**4i**	>95%	71%
26	0	10.0	*p*-ABSA	5.00	CH_3CN	argon	**4i**	>95%	76%
27	1.00	10.0	*p*-ABSA	5.00	CH_3CN	argon	**4i**	>95%	79%
28	2.50	10.0	*p*-ABSA	5.00	CH_3CN	argon	**4i**	95%	78%
29	0	6.25	**5a**	1.50	CH_2Cl_2	argon	**7a**	>95%	92%
30	3.75	10.0	**6a**	2.50	*n*-propanol	argon	**4j**	>95%	60%
31	3.75	10.0	**6b**	2.50	*n*-propanol	argon	**4k**	>95%	48%
32	0	5.00	**6c**	5.00	CH_2Cl_2	argon	**4l**	>95%	66%
33	0	5.00	**6d**	1.00	CH_3CN	argon	**4m**	>95%	50%

General experimental procedures. All reactions were performed in single-neck, flame-dried, round-bottomed flasks fitted with rubber septa under a positive pressure of argon, unless otherwise noted. Air- and moisture-sensitive liquids were transferred via syringe or stainless steel cannula, or were handled in a nitrogen-filled drybox (working oxygen level <5 ppm). Organic solutions were concentrated by rotary evaporation at 30–33 °C. Intermediates were purified using a Biotage Isolera system, employing polypropylene cartridges preloaded with silica gel (60 Å, 40–63 µm particle size, purchased from Silicycle, Quebec City, Canada). Alternatively, intermediates were purified using a Teledyne ISCO system, employing RediSep Rf High Performance Gold cartridges (RediSep Rf Gold Silica, 20–40 um spherical, purchased from Teledyne ISCO, Dallas, Texas). Samples were eluted using a flow rate of 12–50 mL/min, with detection by UV (254 nm). Analytical thin-layered chromatography (TLC) was performed using glass plates pre-coated with silica gel (0.25 mm, 60 Å pore size) impregnated with a fluorescent indicator (254 nm). TLC plates were visualized by exposure to ultraviolet light (UV) and/or submersion in aqueous ceric ammonium molybdate solution (CAM), or aqueous potassium permanganate solution ($KMnO_4$), followed by brief heating on a hot plate (120 °C, 10–15 s).

Materials. Commercial solvents and reagents were used as received with the following exceptions. Acetonitrile was purified according to the method of Pangborn et al[1]. Pyridine was distilled from calcium hydride under an atmosphere of nitrogen immediately before use. Methanol was distilled from magnesium shavings under an atmosphere of nitrogen immediately before use. Commercial anhydrous ethanol was stored over 4 Å MS under an atmosphere of nitrogen before use. Tetrahydrofuran was distilled from sodium–benzophenone under an atmosphere of nitrogen immediately before use. *n*-Propanol was dried over calcium hydride for 12 h at 24 °C, degassed by three freeze–pump–thaw cycles, vacuum transferred, and stored under an atmosphere of argon before use. Triethylsilane was degassed by three freeze–pump–thaw cycles and stored under an atmosphere of argon before use. 1,4-Dihydrobenzene was degassed by three freeze–pump–thaw cycles, vacuum transferred, and stored under an atmosphere of argon at –10 °C before use. Cobalt bis(acetylacetonate) was dried by heating overnight in vacuo (70 °C, 200 mTorr), and stored under an atmosphere of argon before use. Tosyl iodide[2], (η^6-benzene) manganese tricarbonyl hexafluorophosphate[3], *N*-hydroxy-1-(phenylsulfonyl)methanimidoyl cyanide sodium salt[4], and phenyl *N*-benzyloxymethanimidothioate[4] were prepared according to published procedures. *p*-Toluenesulfonyl chloride was recrystallized from chloroform–pentane immediately before use. *N*-Iodosuccinimide was recrystallized from 1,4-dioxane–tetrachloromethane immediately before use. *m*-Chloroperbenzoic acid was recrystallized from dichloromethane immediately before use.

Instrumentation. Proton nuclear magnetic resonance spectra (^{1}H NMR) were recorded at 400, 500, or 600 MHz at 24 °C, unless otherwise noted. Chemical shifts are expressed in parts per million (ppm, δ scale) downfield from tetramethylsilane and are referenced to residual protium in the NMR solvent ($CHCl_3$, δ 7.26; $CHDCl_2$, δ 5.32). Data are represented as follows: chemical shift, multiplicity (s = singlet, d = doublet, t = triplet, q = quartet, m = multiplet and/or multiple resonances, br = broad), integration, coupling constant in Hertz, and assignment. Proton-decoupled carbon nuclear magnetic resonance spectra (^{13}C NMR) were recorded at 100, 125, or 150 MHz at 24 °C, unless otherwise noted. Chemical shifts are expressed in parts per million (ppm, δ scale) downfield from tetramethylsilane and are referenced to the carbon resonances of the solvent ($CDCl_3$, δ 77.0; CD_2Cl_2, δ 54.0). Distortionless enhancement by polarization transfer spectra [DEPT (135)] were recorded at 100, 125, or 150 MHz at 24 °C, unless otherwise noted. ^{13}C NMR and DEPT (135) data are combined and represented as follows: chemical shift, carbon type [obtained from DEPT (135) experiments]. Proton-decoupled fluorine nuclear magnetic resonance spectra (^{19}F NMR) were recorded at 375 MHz or 470 MHz at 24 °C, unless otherwise noted. Chemical shifts are expressed in parts per million (ppm, δ scale) downfield from fluorotrichloromethane. Attenuated total reflectance Fourier transform infrared spectra (ATR-FTIR) were obtained using a Thermo Electron Corporation Nicolet 6700 FTIR spectrometer referenced to a polystyrene standard. Data are represented as follows: frequency of absorption (cm^{-1}), intensity of absorption (s = strong, m = medium, w = weak, br = broad). High-resolution mass spectrometry (HRMS) were obtained on a Waters UPLC/HRMS

instrument equipped with a dual API/ESI high-resolution mass spectrometry detector and photodiode array detector. Unless otherwise noted, samples were eluted over a reverse-phase C18 column (1.7 μm particle size, 2.1 × 50 mm) with a linear gradient of 5% acetonitrile–water containing 0.1% formic acid→95% acetonitrile–water containing 0.1% formic acid over 1.6 min, followed by 100% acetonitrile containing 0.1% formic acid for 1 min, at a flow rate of 600 μL/min.

Synthetic procedures.

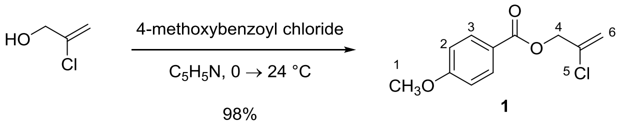

*Preparation of 2-chloroallyl 4-methoxybenzoate (**1**):*

4-Methoxybenzoyl chloride (1.02 g, 5.96 mmol, 1.10 equiv) was added dropwise via syringe to a solution of 2-chloro-2-propen-1-ol (500 mg, 5.41 mmol, 1 equiv) in pyridine (22 mL) at 0 °C. The reaction mixture was stirred for 30 min at 0 °C, and then the ice bath was removed. The reaction mixture was stirred for 24 h at 24 °C. The product mixture was transferred to a separatory funnel that had been charged with ethyl acetate (20 mL). The diluted product mixture was washed with saturated aqueous sodium bicarbonate solution (20 mL). The aqueous layer was isolated and the isolated aqueous layer was extracted with ethyl acetate (3 × 20 mL). The organic layers were combined and the combined organic layers were dried over sodium sulfate. The dried solution was filtered and the filtrate was concentrated. The residue obtained was purified by automated flash-column chromatography (eluting with 5% ethyl acetate–hexanes initially, grading to 10% ethyl acetate–hexanes, linear gradient) to afford 2-chloroallyl 4-methoxybenzoate (**1**) as a clear oil (1.20 g, 98%).

R_f = 0.52 (10% ethyl acetate–hexanes; UV, $KMnO_4$). ^{1}H NMR (500 MHz, $CDCl_3$) δ 8.04 (d, 2H, J = 9.0 Hz, H_3), 6.94 (d, 2H, J = 9.0 Hz, H_2), 5.55–5.53 (m, 1H, H_6), 5.45–5.42 (m, 1H, H_6), 4.87 (br s, 2H, H_4), 3.87 (s, 3H, H_1). ^{13}C NMR (125 MHz, $CDCl_3$) δ 165.0 (C), 163.4 (C), 135.9 (C), 131.6 (CH), 121.5 (C), 114.4 (CH_2), 113.5 (CH), 65.8 (CH_2), 55.2 (CH_3).

^{1}H and ^{13}C NMR data for 2-chloroallyl 4-methoxybenzoate (**1**) prepared in this way were in agreement with those previously described[5].

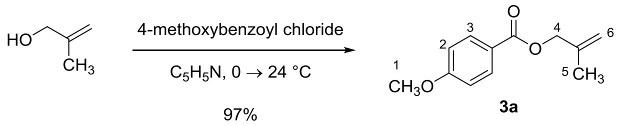

*Preparation of 2-methylallyl 4-methoxybenzoate (**3a**):*

4-Methoxybenzoyl chloride (1.50 g, 8.80 mmol, 1.10 equiv) was added dropwise via syringe to a solution of 2-methyl-2-propen-1-ol (576 mg, 8.00 mmol, 1 equiv) in pyridine (32 mL) at 0 °C. The reaction mixture was stirred for 30 min at 0 °C, and then the ice bath was removed. The reaction mixture was stirred for 24 h at 24 °C. The product mixture was transferred to a separatory funnel that had been charged with ethyl acetate (50 mL). The diluted product mixture was washed with saturated aqueous sodium bicarbonate solution (50 mL). The aqueous layer was isolated and the isolated aqueous layer was extracted with ethyl acetate (3 × 50 mL). The organic layers were combined and the combined organic layers were dried over sodium sulfate. The dried solution was filtered and the filtrate was concentrated. The residue obtained was purified by automated flash-column chromatography (eluting with 5% ethyl acetate–hexanes initially, grading to 10% ethyl acetate–hexanes, linear gradient) to afford 2-methylallyl 4-methoxybenzoate (**3a**) as a clear oil (1.60 g, 97%).

R_f = 0.55 (20% ethyl acetate–hexanes; UV, $KMnO_4$). ^{1}H NMR (600 MHz, $CDCl_3$) δ 8.02 (d, 2H, J = 8.4 Hz, H_3), 6.92 (d, 2H, J = 8.4 Hz, H_2), 5.05 (s, 1H, H_6), 4.96 (s, 1H, H_6), 4.71 (s, 2H, H_4), 3.87 (s, 3H, H_1), 1.82 (s, 3H, H_5). ^{13}C NMR (150 MHz, $CDCl_3$) δ 166.0 (C), 163.4 (C), 140.2 (C), 131.6 (CH), 122.6 (C), 113.6 (CH), 112.7 (CH_2), 67.8 (CH_2), 55.4 (CH_3), 19.6 (CH_3).

^{1}H and ^{13}C NMR data for 2-methylallyl 4-methoxybenzoate (**3a**) prepared in this way were in agreement with those previously described[6].

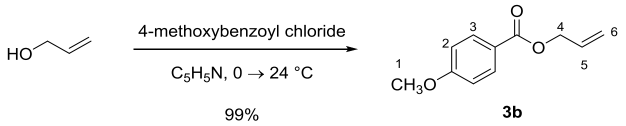

*Preparation of allyl 4-methoxybenzoate (**3b**):*

4-Methoxybenzoyl chloride (1.50 g, 8.80 mmol, 1.10 equiv) was added dropwise via syringe to a solution of allyl alcohol (464 mg, 8.00 mmol, 1 equiv) in pyridine (32 mL) at 0 °C. The reaction mixture was stirred for 30 min at 0 °C, and then the ice bath was removed. The reaction mixture was stirred for 24 h at 24 °C. The product mixture was transferred to a separatory funnel that had been charged with ethyl acetate (50 mL). The diluted product mixture was washed with saturated aqueous sodium bicarbonate solution (50 mL). The aqueous layer was isolated and the isolated aqueous layer was extracted with ethyl acetate (3 × 50 mL). The organic layers were combined and the combined organic layers were dried over sodium sulfate. The dried solution was filtered and the filtrate was concentrated. The residue obtained was purified by automated flash-column chromatography (eluting with 5% ethyl acetate–hexanes initially, grading to 10% ethyl acetate–hexanes, linear gradient) to afford allyl 4-methoxybenzoate (**3b**) as a clear oil (1.54 g, 99%).

R_f = 0.55 (20% ethyl acetate–hexanes; UV, $KMnO_4$). ^{1}H NMR (600 MHz, $CDCl_3$) δ 8.02 (d, 2H, J = 8.4 Hz, H_3), 6.92 (d, 2H, J = 8.4 Hz, H_2), 6.03 (ddt, J = 16.8, 10.2, 4.2 Hz, 1H, H_5), 5.40 (d, J = 16.8 Hz, 1H, H_6), 5.27 (d, J = 10.2 Hz, 1H, H_6), 4.80 (d, J =4.2 Hz, 2H, H_4), 3.86 (s, 3H, H_1). ^{13}C NMR (150 MHz, $CDCl_3$) δ 166.0 (C), 163.4 (C), 132.5 (CH), 131.6 (CH), 122.5 (C), 117.9 (CH_2), 113.6 (CH), 65.2 (CH_2), 55.4 (CH_3).

^{1}H and ^{13}C NMR data for 2-methylallyl allyl 4-methoxybenzoate (**3b**) prepared in this way were in agreement with those previously described[7].

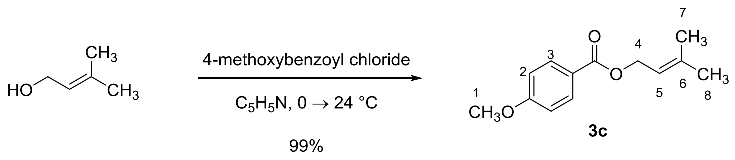

3c

*Preparation of 3-methylbut-2-en-1-yl 4-methoxybenzoate (**3c**):*

4-Methoxybenzoyl chloride (563 mg, 3.30 mmol, 1.10 equiv) was added dropwise via syringe to a solution of 3-methylbut-2-en-1-ol (258 mg, 3.00 mmol, 1 equiv) in pyridine (12 mL) at 0 °C. The reaction mixture was stirred for 30 min at 0 °C, and then the ice bath was removed. The reaction mixture was stirred for 24 h at 24 °C. The product mixture was transferred to a separatory funnel that had been charged with ethyl acetate (20 mL). The diluted product mixture was washed with saturated aqueous sodium bicarbonate solution (20 mL). The aqueous layer was isolated and the isolated aqueous layer was extracted with ethyl acetate (3 × 20 mL). The organic layers were combined and the combined organic layers were dried over sodium sulfate. The dried solution was filtered and the filtrate was concentrated. The residue obtained was purified by automated flash-column chromatography (eluting with 5% ethyl acetate–hexanes initially, grading to 10% ethyl acetate–hexanes, linear gradient) to afford 3-methylbut-2-en-1-yl 4-methoxybenzoate (**3c**) as a clear oil (661 mg, 99%).

R_f = 0.55 (20% ethyl acetate–hexanes; UV, $KMnO_4$). ^{1}H NMR (500 MHz, CD_2Cl_2) δ 7.98 (d, 2H, J = 8.5 Hz, H_3), 6.94 (d, 2H, J = 9.0 Hz, H_2), 5.47 (t, J = 7.0 Hz, 1H, H_5), 4.78 (d, J = 7.5 Hz, 2H, H_4), 3.86 (s, 3H, H_1), 1.80 (s, 3H, H_7), 1.78 (s, 3H, H_8). ^{13}C NMR (125 MHz, CD_2Cl_2) δ 166.6 (C), 163.9 (C), 139.4 (C), 131.9 (CH), 123.6 (C), 119.5 (CH), 114.1 (CH), 62.0 (CH_2), 56.0 (CH_3), 26.0 (CH_3), 18.4 (CH_3). IR (ATR-FTIR), cm^{-1}: 2935 (w), 1707 (s), 1606 (s), 1251 (s), 1096 (s). HRMS-ESI (m/z): $[M + Na]^+$ calcd for $C_{12}H_{12}NaO_3$, 243.0997; found, 243.1003.

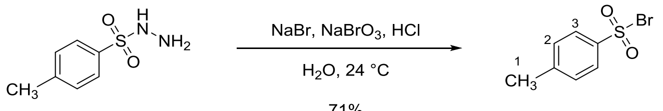

*Preparation of p-toluenesulfonyl bromide (**S1**):*

A solution of sodium bromide (815 mg, 7.92 mmol, 0.333 equiv) and sodium bromate (2.43 g, 16.1 mmol, 0.667 equiv) in water (14 mL) was added dropwise to a suspension of *p*-toluenesulfonyl hydrazide (4.47 g, 24.0 mmol, 1 equiv) in an aqueous solution of hydrochloric acid (10% w/w, 80 mL) at 24 °C. The product mixture was stirred for 10 min and filtered immediately. The residue obtained was recrystallized from petroleum ether to afford *p*-toluenesulfonyl bromide (**S1**) as a white crystalline solid (3.99 g, 71%).

^{1}H NMR (500 MHz, $CDCl_3$) δ 7.88 (d, 2H, J = 8.5 Hz, H_3), 7.39 (d, 2H, J = 8.5 Hz, H_2), 2.49 (s, 3H, H_1). ^{13}C NMR (125 MHz, $CDCl_3$) δ 146.7 (C), 144.6 (C), 130.1 (CH), 126.4 (CH), 21.8 (CH_3).

^{1}H and ^{13}C NMR data for *p*-toluenesulfonyl bromide (**S1**) prepared in this way were in agreement with those previously described[8].

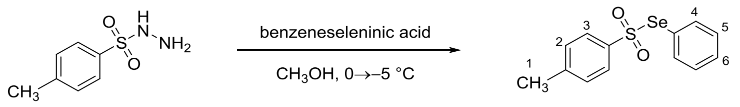

S2

*Preparation of Se-phenyl seleno-p-toluenesulfonate (**S2**):*

A suspension of *p*-toluenesulfonyl hydrazide (1.86 g, 10.0 mmol, 1 equiv) in methanol (8.0 mL) was added dropwise over 15 min to a suspension of benzeneseleninic acid (1.89 g, 10.0 mmol, 1.00 equiv) in methanol (8.0 mL) at 0 °C. Vigorous evolution of nitrogen gas was observed and a yellow precipitate was formed. The reaction mixture was cooled to –5 °C overnight. The suspension was filtered to afford *Se*-phenyl seleno-*p*-toluenesulfonate (**S2**) as a light yellow solid (2.83 g, 91%).

^{1}H NMR (500 MHz, $CDCl_3$) δ 7.51 (d, J = 8.0 Hz, 2H, H_4), 7.46 (t, J = 7.5 Hz, 1H, H_6), 7.39 (d, J = 7.0 Hz, 2H, H_3), 7.34 (t, J = 7.5 Hz, 2H, H_5), 7.18 (d, J = 8.0 Hz, 2H, H_2), 2.41 (s, 3H, H_1). ^{13}C NMR (125 MHz, $CDCl_3$) δ 144.5 (C), 142.7 (C), 137.2 (CH), 130.8 (CH), 129.5 (CH), 128.2 (CH), 128.0 (C), 127.0 (CH), 21.6 (CH_3).

^{1}H and ^{13}C NMR data for *Se*-phenyl seleno-*p*-toluenesulfonate (**S2**) prepared in this way were in agreement with those previously described[9].

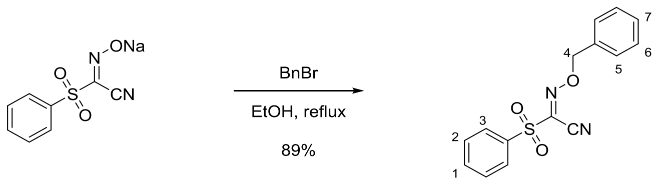

6a

*Preparation of N-(benzyloxy)-1-(phenylsulfonyl)methanimidoyl cyanide (**6a**):*

Benzylbromide (615 µL, 5.17 mmol, 1.20 equiv) was added to a suspension of *N*-hydroxy-1-(phenylsulfonyl)methanimidoyl cyanide sodium salt (1.00 g, 4.31 mmol, 1 equiv) in ethanol (10 mL). The resulting suspension was heated to reflux for 1 h. The product was concentrated to dryness and the residue was suspended with ether (100 mL). The resulting mixture was filtered through a pad of celite and the pad was rinsed with ether (50 mL). The filtrates were combined and the combined filtrates were concentrated to dryness. The residue obtained was purified by flash-column chromatography (eluting with 20% ethyl acetate–hexanes) to afford *N*-(benzyloxy)-1-(phenylsulfonyl)methanimidoyl cyanide (**6a**) as a white solid (1.15 g, 89%).

^{1}H NMR (500 MHz, $CDCl_3$) δ 7.97 (d, J = 7.5 Hz, 2H, H_3), 7.76 (t, J = 7.5 Hz, 1H, H_1), 7.62 (t, J = 8.0 Hz, 2H, H_2), 7.39–7.35 (m, 3H, 2 × H_5, 1 × H_7), 7.33–7.29 (m, 2H, H_6), 5.43 (s, 2H, H_4). ^{13}C NMR (125 MHz, $CDCl_3$) δ 136.5 (C), 135.5 (CH), 134.8 (C), 133.7 (CH), 130.8 (C), 129.9 (CH), 129.4 (CH), 129.2 (CH), 128.8 (CH), 105.7 (C), 81.6 (CH_2).

^{1}H and ^{13}C NMR data for *N*-(benzyloxy)-1-(phenylsulfonyl)methanimidoyl cyanide (**6a**) prepared in this way were in agreement with those previously described[4].

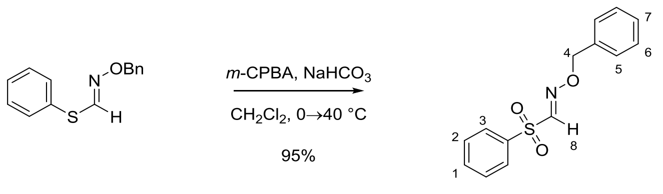

*Preparation of (phenylsulfonyl)methanal O-benzyl oxime (**6b**):*

Six equal portions of *m*-chloroperbenzoic acid (4.40 g, 25.5 mmol, 2.20 equiv) were added over 1 h to a suspension of phenyl *N*-(benzyloxy)methanimidothioate (2.82 g, 11.6 mmol, 1 equiv) and sodium bicarbonate (2.00 g, 23.8 mmol, 2.05 equiv) in dichloromethane (50 mL) at 0 °C. The resulting mixture was stirred at 0 °C for 15 min. The reaction vessel was then placed in an oil bath that had been previously heated to 40 °C. The reaction mixture was stirred and heated at 40 °C for 1 h. The product mixture was allowed to cool down to 24 °C over 30 min and the cooled product mixture was transferred to a separatory funnel that had been charged with dichloromethane (100 mL) and a saturated aqueous sodium bicarbonate solution (50 mL). The layers that formed was separated and the organic layer was washed with aqueous saturated sodium thiosulfate solution (3 × 50 mL). The organic layer was dried and the dried solution was filtered. The filtrate was concentrated to dryness and the residue obtained was purified by flash-column chromatography (eluting with 10% ethyl acetate–hexanes initially, grading to 20% ethyl acetate–hexanes, linear gradient) to afford (phenylsulfonyl)methanal *O*-benzyl oxime (**6b**) as a white solid (3.03 g, 95%).

^{1}H NMR (500 MHz, $CDCl_3$) δ 7.91 (d, J = 7.8 Hz, 2H, H_3), 7.63 (t, J = 7.2 Hz, 1H, H_1), 7.49–7.35 (m, 3H, 2 × H_2, 1 × H_8), 7.29–7.23 (m, 3H, 2 × H_5, 1 × H_7), 7.02 (d, J = 8.4 Hz, 2H, H_6), 5.11 (s, 2H, H_4). ^{13}C NMR (125 MHz, $CDCl_3$) δ 143.8 (CH), 139.1 (C), 135.3 (C), 134.3 (CH), 129.1 (CH), 128.8 (CH), 128.5 (CH), 128.4 (CH), 128.2 (CH), 78.7 (CH_2).

^{1}H and ^{13}C NMR data for (phenylsulfonyl)methanal *O*-benzyl oxime (**6b**) prepared in this way were in agreement with those previously described[4].

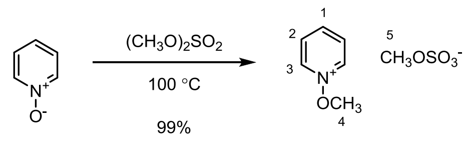

6c

*Preparation of N-methoxypyridinium methyl sulfate (**6c**):*

A 25-mL round-bottomed flask fitted with a rubber septum was charged with pyridine *N*-oxide (2.17 g, 22.8 mmol, 1 equiv). The reaction vessel was evacuated and refilled using a balloon of argon. This process was repeated twice. The reaction vessel was then cooled to 0 °C. Dimethyl sulfate (2.16 mL, 22.8 mmol, 1.00 equiv) was added to the reaction vessel dropwise via syringe over 5 min. The reaction vessel was placed in an oil bath that had been preheated to 100 °C. The reaction mixture was stirred and heated for 5 h at 100 °C. The product mixture was concentrated in vacuo (0.1 Torr) overnight to afford *N*-methoxy pyridinium methyl sulfate (**6c**) as a colorless low-melting solid (4.99 g, 99%).

^{1}H NMR (400 MHz, $(CD_3)_2SO$) δ 9.44 (d, J = 6.8 Hz, 2H, H_3), 8.61 (t, J = 8.0 Hz, 1H, H_1), 8.23 (t, J = 7.2 Hz, 2H, H_2), 4.42 (s, 3H, H_4), 3.37 (s, 3H, H_5). ^{13}C NMR (100 MHz, $(CD_3)_2SO$) δ 145.5 (CH), 141.3 (CH), 129.8 (CH), 69.9 (CH_3), 53.4 (CH_3). IR (ATR-FTIR), cm^{-1}: 3041 (w), 1479 (m), 1220 (s), 1001 (s), 730 (s), 668 (m), 576 (s). HRMS-ESI (m/z): $[M – CH_3OSO_3^{-}]^{+}$ calcd for C_6H_8NO, 110.0600; found, 110.0604.

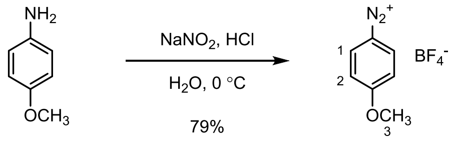

5a

*Preparation of 4-methoxybenzenediazonium tetrafluoroborate (**5a**):*

A solution of tetrafluoroboric acid in water (48% w/w, 6.15 mL, 33.6 mmol, 2.00 equiv) was added to a solution of *p*-anisidine (2.07 g, 16.8 mmol, 1 equiv) in water (8.0 mL) at 0 °C. The resulting suspension was stirred for 10 min at 0 °C. A solution of sodium nitrite (1.16 g, 16.8 mmol, 1.00 equiv) in water (2.0 mL) was added dropwise to the reaction vessel over 2 min at 0 °C. The reaction mixture was stirred for 15 min at 0 °C and the resulting mixture was filtered immediately. The residue obtained was recrystallized with acetone–ether to afford 4-methoxybenzenediazonium tetrafluoroborate (**5a**) as white needles (2.95 g, 79%).

^{1}H NMR (500 MHz, $(CD_3)_2SO$) δ 8.60 (d, J = 9.0 Hz, 2H, H_1), 7.48 (d, J = 9.0 Hz, 2H, H_2), 4.04 (s, 3H, H_3). ^{13}C NMR (125 MHz, $(CD_3)_2SO$) δ 169.2 (C), 136.5 (CH), 117.9 (CH), 103.7 (C), 57.9 (CH_3). ^{19}F NMR (470 MHz, $(CD_3)_2SO$) δ –148.2.

^{1}H and ^{13}C NMR data for 4-methoxybenzenediazonium tetrafluoroborate (**5a**) prepared in this way were in agreement with those previously described[10].

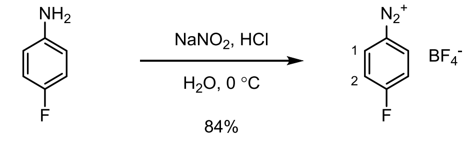

5b

*Preparation of 4-fluorobenzenediazonium tetrafluoroborate (**5b**):*

A solution of tetrafluoroboric acid in water (48% w/w, 3.66 mL, 17.6 mmol, 2.00 equiv) was added to a solution of 4-fluoroaniline (947 μL, 10.0 mmol, 1 equiv) in water (2.0 mL) at 0 °C. The resulting suspension was stirred for 10 min at 0 °C. A solution of sodium nitrite (690 mg, 1.0 mmol, 1.00 equiv) in water (1.0 mL) was added dropwise to the reaction vessel over 2 min at 0 °C. The reaction mixture was stirred for 15 min at 0 °C and the resulting mixture was filtered immediately. The residue obtained was recrystallized with acetone–ether to afford 4-fluorobenzenediazonium tetrafluoroborate (**5b**) as an off-white solid (1.76 g, 84%).

^{1}H NMR (500 MHz, $(CD_3)_2SO$) δ 8.86–8.75 (m, 2H, H_1), 7.93–7.84 (m, 2H, H_2). ^{13}C NMR (125 MHz, $(CD_3)_2SO$) δ 168.4 (d, J = 265.3 Hz, C), 137.0 (d, J = 12.4 Hz, CH), 119.4 (d, J = 25.1 Hz, CH), 111.8 (d, J = 2.9 Hz, C). ^{19}F NMR (470 MHz, $(CD_3)_2SO$) δ –78.2 (1F), –148.3 (4F).

^{1}H and ^{13}C NMR data for 4-fluorobenzenediazonium tetrafluoroborate (**5b**) prepared in this way were in agreement with those previously described[11].

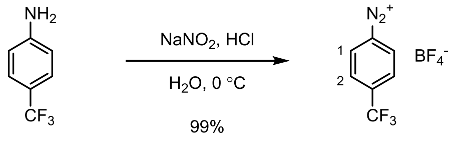

5c

*Preparation of 4-(trifluoromethyl)benzenediazonium tetrafluoroborate (**5c**):*

A solution of tetrafluoroboric acid in water (48% w/w, 3.66 mL, 20.0 mmol, 2.00 equiv) was added to a solution of 4-(trifluoromethyl)aniline (1.26 mL, 10.0 mmol, 1 equiv) in water (2.0 mL) at 0 °C. The resulting suspension was stirred for 10 min at 0 °C. A solution of sodium nitrite (690 mg, 10.0 mmol, 1.00 equiv) in water (1.0 mL) was added dropwise to the reaction vessel over 2 min at 0 °C. The reaction mixture was stirred for 15 min at 0 °C and the resulting mixture was filtered immediately. The residue obtained was recrystallized with acetone–ether to afford 4-(trifluoromethyl)benzenediazonium tetrafluoroborate (**5c**) as a light yellow solid (2.76 g, 99%).

^{1}H NMR (500 MHz, $(CD_3)_2SO$) δ 8.90 (d, J = 8.5 Hz, 2H, H_1), 8.41 (d, J = 9.0 Hz, 2H, H_2). ^{13}C NMR (125 MHz, $(CD_3)_2SO$) δ 138.2 (q, J = 33.4 Hz, C), 133.8 (CH), 128.3 (q, J = 3.8 Hz, CH), 122.3 (q, J = 272.4 Hz, C), 121.3 (C). ^{19}F NMR (470 MHz, $(CD_3)_2SO$) δ –62.7 (3F), –148.2 (4F). IR (ATR-FTIR), cm^{-1}: 3571 (br w), 3120 (w), 2307 (w), 1427 (w), 1318 (m), 1180 (m), 1139 (m), 1026 (s), 1009 (s), 853 (s), 724 (m), 585 (m), 524 (m). HRMS-ESI (m/z): $[M - BF_4^-]^+$ calcd for $C_7H_4F_3N_2^+$, 173.0321; found, 173.0320.

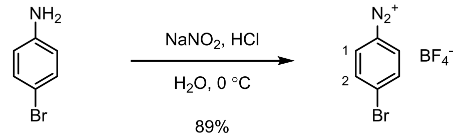

5d

*Preparation of 4-bromobenzenediazonium tetrafluoroborate (**5d**):*

A solution of tetrafluoroboric acid in water (48% w/w, 3.66 mL, 20.0 mmol, 2.00 equiv) was added to a solution of 4-(trifluoromethyl)aniline (1.72 g, 10.0 mmol, 1 equiv) in water (2.0 mL) at 0 °C. The resulting suspension was stirred for 10 min at 0 °C. A solution of sodium nitrite (690 mg, 10.0 mmol, 1.00 equiv) in water (1.0 mL) was added dropwise to the reaction vessel over 2 min at 0 °C. The reaction mixture was stirred for 15 min at 0 °C and the resulting mixture was filtered immediately. The residue obtained was recrystallized with acetone–ether to afford 4-bromobenzenediazonium tetrafluoroborate (**5d**) as a white solid (2.41 g, 89%).

^{1}H NMR (500 MHz, $(CD_3)_2SO$) δ 8.58 (d, J = 9.0 Hz, 2H, H_1), 8.26 (d, J = 9.0 Hz, 2H, H_2). ^{13}C NMR (125 MHz, $(CD_3)_2SO$) δ 136.5 (C), 134.5 (CH), 134.0 (CH), 116.2 (C). ^{19}F NMR (470 MHz, $(CD_3)_2SO$) δ –148.2.

^{1}H and ^{13}C NMR data for 4-bromobenzenediazonium tetrafluoroborate (**5d**) prepared in this way were in agreement with those previously described[12].

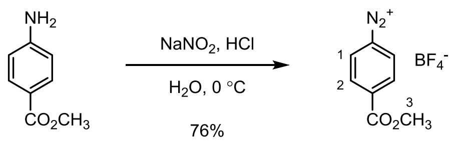

5e

*Preparation of 4-(methoxycarbonyl)benzenediazonium tetrafluoroborate (**5e**):*

A solution of tetrafluoroboric acid in water (48% w/w, 3.66 mL, 20.0 mmol, 2.00 equiv) was added to a solution of methyl 4-aminobenzoate (1.51 g, 10.0 mmol, 1 equiv) in water (2.0 mL) at 0 °C. The resulting suspension was stirred for 10 min at 0 °C. A solution of sodium nitrite (690 mg, 10.0 mmol, 1.00 equiv) in water (1.0 mL) was added dropwise to the reaction vessel over 2 min at 0 °C. The reaction mixture was stirred for 15 min at 0 °C and the resulting mixture was filtered immediately. The residue obtained was recrystallized with acetone–ether to afford 4-(methoxycarbonyl)benzenediazonium tetrafluoroborate (**5e**) as an orange solid (1.89 g, 76%).

^{1}H NMR (500 MHz, $(CD_3)_2SO$) δ 8.79 (d, J = 8.5 Hz, 2H, H_1), 8.44 (d, J = 8.5 Hz, 2H, H_2), 3.95 (s, 3H, H_3). ^{13}C NMR (125 MHz, $(CD_3)_2SO$) δ 163.9 (C), 139.3 (C), 133.2 (CH), 131.3 (CH), 120.2 (C), 53.4 (CH_3). ^{19}F NMR (470 MHz, $(CD_3)_2SO$) δ –148.2.

^{1}H and ^{13}C NMR data for 4-(methoxycarbonyl)benzenediazonium tetrafluoroborate (**5e**) prepared in this way were in agreement with those previously described[13].

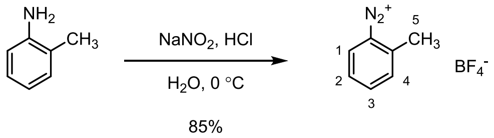

5f

*Preparation of 2-methylbenzenediazonium tetrafluoroborate (**5f**):*

A solution of tetrafluoroboric acid in water (48% w/w, 3.66 mL, 20.0 mmol, 2.00 equiv) was added to a solution of 2-methylaniline (1.06 mL, 10.0 mmol, 1 equiv) in water (2.0 mL) at 0 °C. The resulting suspension was stirred for 10 min at 0 °C. A solution of sodium nitrite (690 mg, 10.0 mmol, 1.00 equiv) in water (1.0 mL) was added dropwise to the reaction vessel over 2 min at 0 °C. The reaction mixture was stirred for 15 min at 0 °C and the resulting mixture was filtered immediately. The residue obtained was recrystallized with acetone–ether to afford 2-methylbenzenediazonium tetrafluoroborate (**5f**) as a light yellow solid (1.76 g, 85%).

^{1}H NMR (500 MHz, $(CD_3)_2SO$) δ 8.64 (d, J = 8.0 Hz, 1H, H_1), 8.15 (t, J = 7.8 Hz, 1H, H_3), 7.84 (d, J = 8.0 Hz, 1H, H_4), 7.80 (t, J = 8.0 Hz, 1H, H_2), 2.74 (s, 3H, H_5). ^{13}C NMR (125 MHz, $(CD_3)_2SO$) δ 143.8 (C), 140.7 (CH), 132.6 (CH), 132.5 (CH), 128.8 (CH), 116.0 (C), 18.2 (CH_3). ^{19}F NMR (470 MHz, $(CD_3)_2SO$) δ –148.3.

^{1}H and ^{13}C NMR data for 2-methylbenzenediazonium tetrafluoroborate (**5f**) prepared in this way were in agreement with those previously described[12].

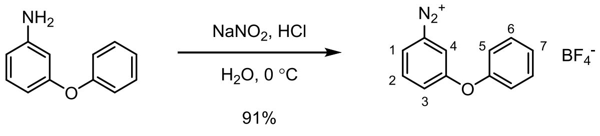

5g

*Preparation of 3-phenoxybenzenediazonium tetrafluoroborate (**5g**):*

A solution of tetrafluoroboric acid in water (48% w/w, 3.66 mL, 20.0 mmol, 2.00 equiv) was added to a solution of 3-phenoxyaniline (1.85 g, 10.0 mmol, 1 equiv) in water (2.0 mL) at 0 °C. The resulting suspension was stirred for 10 min at 0 °C. A solution of sodium nitrite (690 mg, 10.0 mmol, 1.00 equiv) in water (1.0 mL) was added dropwise to the reaction vessel over 2 min at 0 °C. The reaction mixture was stirred for 15 min at 0 °C and the resulting mixture was filtered immediately. The residue obtained was recrystallized with acetone–ether to afford 3-phenoxybenzenediazonium tetrafluoroborate (**5g**) as a beige solid (2.58 g, 91%).

^{1}H NMR (500 MHz, $(CD_3)_2SO$) δ 8.44–8.40 (m, 1H, H_1), 8.20 (s, 1H, H_4), 8.00–7.94 (m, 2H, 1 × H_2, 1 × H_3), 7.55 (t, J = 8.0 Hz, 2H, H_6), 7.36 (t, J = 7.5 Hz, 1H, H_7), 7.23 (d, J = 7.5 Hz, 2H, H_5). ^{13}C NMR (125 MHz, $(CD_3)_2SO$) δ 157.9 (C), 153.9 (C), 132.9 (CH), 130.9 (CH), 130.6 (CH), 127.4 (CH), 125.9 (CH), 120.3 (CH), 118.9 (CH), 117.0 (C). ^{19}F NMR (470 MHz, $(CD_3)_2SO$) δ –148.3. IR (ATR-FTIR), cm^{-1}: 3109 (m), 2302 (m), 1585 (m), 1476 (m), 1326 (w), 1283 (w), 1240 (m), 1019 (s), 926 (m), 873 (m), 807 (m), 775 (s), 692 (m), 663 (m), 522 (m), 466 (m). HRMS-ESI (m/z): $[M – BF_4^–]^+$ calcd for $C_{12}H_9N_2O$, 197.0709; found, 197.0705.

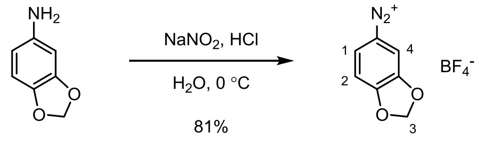

5h

*Preparation of 3,4-methylenedioxybenzenediazonium tetrafluoroborate (**5h**):*

A solution of tetrafluoroboric acid in water (48% w/w, 3.66 mL, 20.0 mmol, 2.00 equiv) was added to a solution of 3,4-methylenedioxyaniline (1.37 g, 10.0 mmol, 1 equiv) in water (2.0 mL) at 0 °C. The resulting suspension was stirred for 10 min at 0 °C. A solution of sodium nitrite (690 mg, 10.0 mmol, 1.00 equiv) in water (1.0 mL) was added dropwise to the reaction vessel over 2 min at 0 °C. The reaction mixture was stirred for 15 min at 0 °C and the resulting mixture was filtered immediately. The residue obtained was recrystallized with acetone–ether to afford 3,4-methylenedioxybenzenediazonium tetrafluoroborate (**5h**) as a black solid (1.90 g, 81%).

^{1}H NMR (500 MHz, $(CD_3)_2SO$) δ 8.42 (dd, J = 8.5, 2.0 Hz, 1H, H_1), 8.05 (d, J = 2.0 Hz, 1H, H_4), 7.49 (d, J = 8.5 Hz, 1H, H_2), 6.45 (s, 2H, H_3). ^{13}C NMR (125 MHz, $(CD_3)_2SO$) δ 158.8 (C), 148.5 (C), 134.2 (CH), 110.6 (CH), 109.4 (CH), 105.5 (CH_2), 104.4 (C). ^{19}F NMR (470 MHz, $(CD_3)_2SO$) δ –148.3.

^{1}H and ^{13}C NMR data for 3,4-methylenedioxybenzenediazonium tetrafluoroborate (**5h**) prepared in this way were in agreement with those previously described[12].

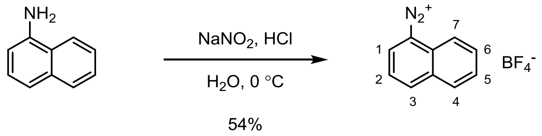

5i

*Preparation of 1-naphthlenediazonium tetrafluoroborate (**5i**):*

A solution of tetrafluoroboric acid in water (48% w/w, 3.66 mL, 20.0 mmol, 2.00 equiv) was added to a solution of 1-naphthylamine (1.43 g, 10.0 mmol, 1 equiv) in water (2.0 mL) at 0 °C. The resulting suspension was stirred for 10 min at 0 °C. A solution of sodium nitrite (690 mg, 10.0 mmol, 1.00 equiv) in water (1.0 mL) was added dropwise to the reaction vessel over 2 min at 0 °C. The reaction mixture was stirred for 15 min at 0 °C and the resulting mixture was filtered immediately. The residue obtained was recrystallized with acetone–ether to afford 1-naphthlenediazonium tetrafluoroborate (**5i**) as a purple solid (1.31 g, 54%).

^{1}H NMR (500 MHz, $(CD_3)_2SO$) δ 9.20 (d, J = 8.0 Hz, 1H, H_4), 8.94 (d, J = 8.5 Hz, 1H, H_3), 8.51 (d, J = 8.5 Hz, 1H, H_7), 8.43 (d, J = 8.5 Hz, 1H, H_1), 8.12 (t, J = 7.8 Hz, 1H, H_2), 8.06 (t, J = 8.0 Hz, 1H, H_5), 7.98 (t, J = 7.5 Hz, 1H, H_6). ^{13}C NMR (125 MHz, $(CD_3)_2SO$) δ 142.6 (CH), 137.2 (CH), 132.6 (C), 132.2 (CH), 130.3 (CH), 129.9 (CH), 127.2 (C), 126.4 (CH), 122.4 (CH), 111.1 (C). ^{19}F NMR (470 MHz, $(CD_3)_2SO$) δ –148.3.

^{1}H and ^{13}C NMR data for 1-naphthlenediazonium tetrafluoroborate (**5i**) prepared in this way were in agreement with those previously described[14].

Selective reduction of 2-chloroallyl 4-methoxybenzoate (1) to 2-chloropropyl 4-methoxybenzoate (2, Scheme 1)

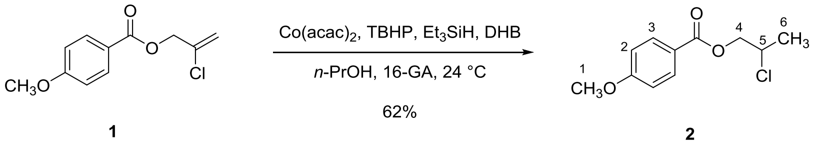

A 10-mL round-bottomed flask fitted with a rubber septum was charged sequentially with 2-chloroallyl 4-methoxybenzoate (**1**, 56.7 mg, 250 μmol, 1 equiv) and cobalt bis(acetylacetonate) (64.3 mg, 250 μmol, 1 equiv). A 16-gauge needle was penetrated through the septum to keep the reaction mixture under air (atmospheric pressure). *n*-Propanol (830 μL), 1,4-cyclohexadiene (119 μL, 1.25 mmol, 5.00 equiv), a solution of *tert*-butyl hydroperoxide in nonane (~5.5 M, 45.5 μL, 250 μmol, 1.00 equiv), and triethylsilane (200 μL, 1.25 mmol, 5.00 equiv) were added sequentially to the reaction vessel via syringe. The reaction mixture was stirred at 24 °C until the consumption of **1** was complete (as determined by TLC analysis, 40 min for **1**). The product mixture was concentrated to dryness and the residue obtained was purified by automated flash-column chromatography (eluting with 5% ethyl acetate–hexanes initially, grading to 10% ethyl acetate–hexanes, linear gradient) to afford 2-chloropropyl 4-methoxybenzoate (**2**) as a clear oil (35.5 mg, 62%).

R_f = 0.52 (20% ethyl acetate–hexanes; UV). ^{1}H NMR (500 MHz, $CDCl_3$) δ 8.02 (d, 2H, J = 9.0 Hz, H_3), 6.93 (d, 2H, J = 9.0 Hz, H_2), 4.42–4.38 (m, 2H, H_4), 4.34–4.27 (m, 1H, H_5), 3.87 (s, 3H, H_1), 1.60 (d, 3H, J = 6.5 Hz, H_6). ^{13}C NMR (150 MHz, $CDCl_3$) δ 165.6 (C), 163.4 (C), 131.7 (CH), 121.9 (C), 113.6 (CH), 68.6 (CH_2), 55.3 (CH_3), 54.1 (CH), 21.5 (CH_3). IR (ATR-FTIR), cm^{-1}: 2936 (w), 1712 (m), 1605 (m), 1252 (s), 1167 (s). HRMS-ESI (m/z): $[M+H]^+$ calcd for $C_{11}H_{14}{}^{35/37}ClO_3$, 229.0631/231.0602; found, 229.0638/231.0614.

Hydrogenation of 2-methylallyl 4-methoxybenzoate (3a) to isobutyl 4-methoxybenzoate (4a, Table 1, entry 1)

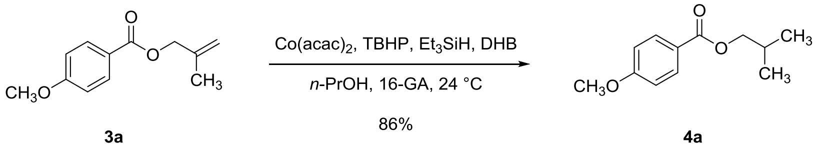

A 25 mL round-bottomed flask fitted with a rubber septum was charged sequentially with 2-methylallyl 4-methoxybenzoate (**3a**, 51.6 mg, 250 μmol, 1 equiv) and cobalt bis(acetylacetonate) (64.3 mg, 250 μmol, 1.00 equiv). A 16-gauge needle was penetrated through the septum. *n*-Propanol (830 μL), 1,4-dihydrobenzene (238 μL, 2.50 mmol, 10.0 equiv), a solution of *tert*-butyl hydroperoxide in nonane (~5.5 M, 45.5 μL, 250 μmol, 1.00 equiv), and triethylsilane (400 μL, 2.50 mmol, 10.0 equiv) were added sequentially to the reaction vessel via syringe. The reaction mixture was stirred at 24 °C for 3.5 h. The product mixture was concentrated to dryness and the residue obtained was purified by automated flash-column chromatography (eluting with 5% ethyl acetate–hexanes initially, grading to 10% ethyl acetate–hexanes, linear gradient) to afford isobutyl 4-methoxybenzoate (**4a**) as a clear oil (44.6 mg, 86%).

R_f = 0.54 (20% ethyl acetate–hexanes; UV). ^{1}H NMR (400 MHz, $CDCl_3$) δ 8.01 (d, 2H, J = 8.8 Hz, H_3), 6.92 (d, 2H, J = 8.8 Hz, H_2), 4.07 (d, J = 6.8 Hz, 2H, H_4), 3.86 (s 3H, H_1), 2.12–2.02 (m, H, H_5), 1.02 (d, J = 6.8 Hz, 6H, H_6). ^{13}C NMR (100 MHz, $CDCl_3$) δ 166.4 (C), 163.2 (C), 131.5 (CH), 123.0 (C), 113.5 (CH), 70.7 (CH_2), 55.4 (CH_3), 27.9 (CH), 19.2 (CH_3).

^{1}H and ^{13}C NMR data for isobutyl 4-methoxybenzoate (**4a**) prepared in this way were in agreement with those previously described[15].

Hydrofluorination of 2-methylallyl 4-methoxybenzoate (3a) to 2-fluoro-2-methylpropyl 4-methoxybenzoate (4b, Table 1, entry 2)

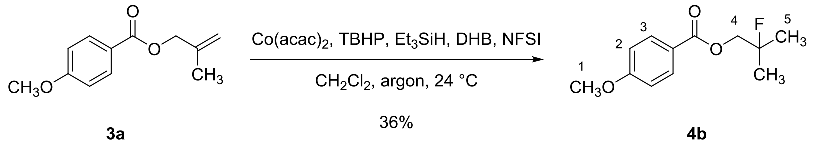

A 10 mL round-bottomed flask fitted with a rubber septum was charged sequentially with 2-methylallyl 4-methoxybenzoate (**3a**, 51.6 mg, 250 μmol, 1 equiv), *N*-fluorobenzenesulfonimide (197 mg, 625 μmol, 2.50 equiv), and cobalt bis(acetylacetonate) (64.3 mg, 250 μmol, 1.00 equiv). The reaction vessel was evacuated and refilled using a balloon of argon. This process was repeated twice. Dichloromethane (833 μL), 1,4-dihydrobenzene (57.4 μL, 938 μmol, 2.50 equiv), triethylsilane (400 μL, 2.50 mmol, 10.0 equiv), and a solution of *tert*-butyl hydroperoxide in nonane (~5.5 M, 45.5 μL, 250 μmol, 1.00 equiv) were added sequentially to the reaction vessel via syringe. The reaction mixture was stirred for 12 h at 24 °C. The product mixture was concentrated to dryness and the residue obtained was purified by flash-column chromatography (eluting with 2% ethyl acetate–hexanes initially, grading to 5% ethyl acetate–hexanes, linear gradient) to afford separately 2-fluoro-2-methylpropyl 4-methoxybenzoate (**4b**, clear oil, 20.5 mg, 36%) and 2-methylallyl 4-methoxybenzoate (**3a**, clear oil, 14.8 mg, 29%).

2-Fluoro-2-methylpropyl 4-methoxybenzoate (**4b**): R_f = 0.47 (20% ethyl acetate–hexanes; UV). ^{1}H NMR (500 MHz, CD_2Cl_2) δ 8.01 (d, J = 8.5 Hz, 2H, H_3), 6.95 (d, J = 8.5 Hz, 2H, H_2), 4.28 (d, J = 20.0 Hz, 2H, H_4), 3.86 (s, 3H, H_1), 1.46 (d, J = 21.0 Hz, 6H, H_5). ^{13}C NMR (125 MHz, CD_2Cl_2) δ 166.2 (C), 164.2 (C), 132.1 (CH), 122.9 (C), 114.2 (CH), 94.1 (d, J = 168.5 Hz, C), 69.8 (d, J = 25.1 Hz, CH_2), 56.1 (CH_3), 24.2 (d, J = 24.0 Hz, CH_3). ^{19}F NMR (470 MHz, CD_2Cl_2) δ –146.4. IR (ATR-FTIR), cm^{-1}: 2983 (w), 1713 (s), 1606 (s), 1581 (w), 1512 (m), 1462 (w), 1379 (m), 1278 (s), 1250 (s), 1165 (s), 1100 (s), 1027 (s), 885 (m), 847 (s), 769 (s), 696 (m), 635 (w), 613 (m). HRMS-ESI (m/z): $[M + H]^+$ calcd for $C_{12}H_{16}FO_3$, 227.1083; found, 227.1083.

Hydrochlorination of 2-methylallyl 4-methoxybenzoate (3a) to 2-chloro-2-methylpropyl 4-methoxybenzoate (4c, Table 1, entry 3)

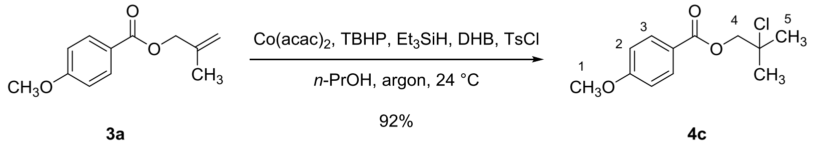

A 10 mL round-bottomed flask fitted with a rubber septum was charged sequentially with 2-methylallyl 4-methoxybenzoate (**3a**, 51.6 mg, 250 µmol, 1 equiv), *p*-toluenesulfonyl chloride (119 mg, 625 µmol, 2.50 equiv), and cobalt bis(acetylacetonate) (64.3 mg, 250 µmol, 1.00 equiv). The reaction vessel was evacuated and refilled using a balloon of argon. This process was repeated twice. *n*-Propanol (833 µL), 1,4-dihydrobenzene (57.4 µL, 938 µmol, 2.50 equiv), triethylsilane (400 µL, 2.50 mmol, 10.0 equiv), and a solution of *tert*-butyl hydroperoxide in nonane (~5.5 M, 45.5 µL, 250 µmol, 1.00 equiv) were added sequentially to the reaction vessel via syringe. The reaction mixture was stirred for 12 h at 24 °C. The product mixture was concentrated to dryness and the residue obtained was purified by flash-column chromatography (eluting with 2% ethyl acetate–hexanes initially, grading to 5% ethyl acetate–hexanes, linear gradient) to afford 2-chloro-2-methylpropyl 4-methoxybenzoate (**4c**) as a clear oil (55.7 mg, 92%).

R_f = 0.47 (20% ethyl acetate–hexanes; UV). ^{1}H NMR (400 MHz, CD_2Cl_2) δ 8.03 (d, J = 8.8 Hz, 2H, H_3), 6.96 (d, J = 8.8 Hz, 2H, H_2), 4.35 (s, 2H, H_4), 3.86 (s, 3H, H_1), 1.68 (s, 6H, H_5). ^{13}C NMR (100 MHz, CD_2Cl_2) δ 166.0 (C), 164.2 (C), 132.2 (CH), 122.8 (C), 114.3 (CH), 72.7 (CH_2), 67.5 (C), 56.1 (CH_3), 29.8 (CH_3). IR (ATR-FTIR), cm^{-1}: 2983 (w), 1713 (s), 1606 (s), 1581 (w), 1512 (m), 1462 (w), 1379 (m), 1278 (s), 1250 (s), 1165 (s), 1100 (s), 1027 (s), 885 (m), 847 (s), 769 (s), 696 (m), 635 (w), 613 (m). HRMS-ESI (m/z): $[M + H]^+$ calcd for $C_{12}H_{16}FO_3$, 227.1083; found, 227.1083.

Hydrobromination of 2-methylallyl 4-methoxybenzoate (3a) to 2-bromo-2-methylpropyl 4-methoxybenzoate (4d, Table 1, entry 4)

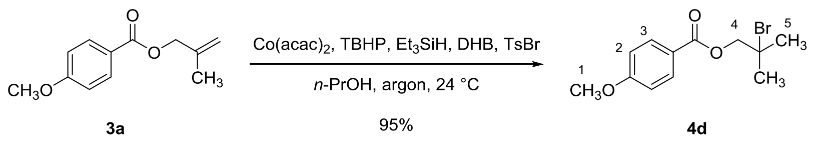

A 10 mL round-bottomed flask fitted with a rubber septum was charged sequentially with 2-methylallyl 4-methoxybenzoate (**3a**, 51.6 mg, 250 μmol, 1 equiv), tosyl bromide (147 mg, 625 μmol, 2.50 equiv), and cobalt bis(acetylacetonate) (64.3 mg, 250 μmol, 1.00 equiv). The reaction vessel was evacuated and refilled using a balloon of argon. This process was repeated twice. *n*-Propanol (830 μL), 1,4-dihydrobenzene (86.0 μL, 938 μmol, 3.75 equiv), a solution of *tert*-butyl hydroperoxide in nonane (~5.5 M, 45.5 μL, 250 μmol, 1.00 equiv), and triethylsilane (400 μL, 2.50 mmol, 10.0 equiv) were added sequentially to the reaction vessel via syringe. The reaction mixture was stirred for 4.0 h at 24 °C. The product mixture was filtered through a short column of silica gel and the short column was rinsed with 20% ethyl acetate–hexanes (100 mL). The filtrates were combined and the combined filtrates were concentrated. The residue obtained was purified by flash-column chromatography (eluting with 2% ethyl acetate–hexanes initially, grading to 7% ethyl acetate–hexanes, linear gradient) to 2-bromo-2-methylpropyl 4-methoxybenzoate (**4d**) as a clear oil (71.5 mg, 95%).

R_f = 0.47 (20% ethyl acetate–hexanes; UV). ^{1}H NMR (500 MHz, CD_2Cl_2) δ 8.03 (d, J = 8.5 Hz, 2H, H_3), 6.96 (d, J = 8.5 Hz, 2H, H_2), 4.40 (s, 2H, H_4), 3.87 (s, 3H, H_1), 1.85 (s, 6H, H_5). ^{13}C NMR (125 MHz, CD_2Cl_2) δ 165.9 (C), 164.2 (C), 132.2 (CH), 122.7 (C), 114.3 (CH), 73.5 (CH_2), 62.3 (C), 56.1 (CH_3), 31.4 (CH_3). IR (ATR-FTIR), cm^{-1}: 2970 (w), 1713 (m), 1605 (m), 1252 (s), 1096 (s). HRMS-ESI (m/z): $[M + H]^+$ calcd for $C_{12}H_{16}{}^{79/81}BrO_3$, 287.0283/289.0262; found, 287.0280/289.0261.

Hydroiodination of 2-methylallyl 4-methoxybenzoate (3a) to 2-iodo-2-methylpropyl 4-methoxybenzoate (4e, Table 2, entry 5)

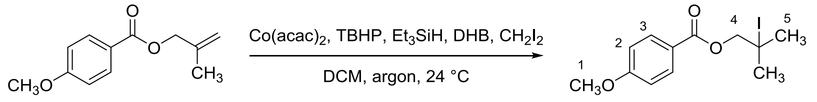

3a **4e**

A 10 mL round-bottomed flask fitted with a rubber septum was charged sequentially with 2-methylallyl 4-methoxybenzoate (**3a**, 51.6 mg, 250 μmol, 1 equiv) and cobalt bis(acetylacetonate) (64.3 mg, 250 μmol, 1.00 equiv). The reaction vessel was evacuated and refilled using a balloon of argon. This process was repeated twice. The reaction vessel was protected from light with aluminum foil. Dichloromethane (830 μL), diiodomethane (302 μL, 3.75 mmol, 15.0 equiv), 1,4-dihydrobenzene (115 μL, 1.25 mmol, 5.00 equiv), a solution of *tert*-butyl hydroperoxide in nonane (~5.5 M, 45.5 μL, 250 μmol, 1.00 equiv), and triethylsilane (400 μL, 2.50 mmol, 10.0 equiv) were added sequentially to the reaction vessel via syringe. The reaction mixture was stirred for 50 h at 24 °C. The product mixture was concentrated and the residue obtained was purified by flash-column chromatography (eluting with 2% ethyl acetate–hexanes initially, grading to 7% ethyl acetate–hexanes, linear gradient) to 2-iodo-2-methylpropyl 4-methoxybenzoate (**4e**) as a clear oil (74.3 mg, 89%).

R_f = 0.47 (20% ethyl acetate–hexanes; UV). ^{1}H NMR (400 MHz, CD_2Cl_2) δ 8.04 (d, J = 8.8 Hz, 2H, H_3), 6.96 (d, J = 8.8 Hz, 2H, H_2), 4.32 (s, 2H, H_4), 3.87 (s, 3H, H_1), 2.01 (s, 6H, H_5). ^{13}C NMR (100 MHz, CD_2Cl_2) δ 165.8 (C), 164.3 (C), 132.2 (CH), 122.7 (C), 114.3 (CH), 76.1 (CH_2), 56.1 (CH_3), 43.5 (C), 34.8 (CH_3). IR (ATR-FTIR), cm^{-1}: 2963 (w), 1714 (m), 1605 (m), 1254 (s), 1099 (s). HRMS-ESI (m/z): $[M + H]^+$ calcd for $C_{12}H_{16}IO_3$,335.0144; found, 335.0148.

Hydration of 2-methylallyl 4-methoxybenzoate (3a) to 2-hydroxy-2-methylpropyl 4-methoxybenzoate (4f, Table 2, entry 6)

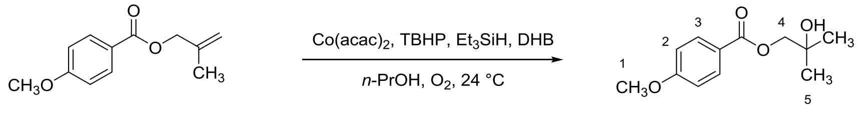

A 25 mL round-bottomed flask fitted with a rubber septum was charged sequentially with 2-methylallyl 4-methoxybenzoate (**3a**, 51.6 mg, 250 μmol, 1 equiv) and cobalt bis(acetylacetonate) (16.1 mg, 62.5 μmol, 0.250 equiv). The reaction vessel was left open to air. *n*-Propanol (830 μL), 1,4-dihydrobenzene (238 μL, 2.50 mmol, 10.0 equiv), a solution of *tert*-butyl hydroperoxide in nonane (~5.5 M, 11.4 μL, 62.5 μmol, 0.250 equiv), and triethylsilane (400 μL, 2.50 mmol, 10.0 equiv) were added sequentially to the reaction vessel via syringe. The reaction vessel was evacuated and refilled using a balloon of oxygen. This process was repeated twice. The reaction mixture was stirred at 24 °C until the consumption of **3a** was complete (as determined by TLC analysis, 180 min for **3a**). The product mixture was concentrated to dryness and the residue obtained was purified by automated flash-column chromatography (eluting with 2% ether–hexanes initially, grading to 20% ether–hexanes, linear gradient) to afford 2-hydroxy-2-methylpropyl 4-methoxybenzoate (**4f**, clear oil, 38.5 mg, 69%).

R_f = 0.29 (20% ether–hexanes; UV). ^{1}H NMR (400 MHz, CD_2Cl_2) δ 9.46 (s, 1H, OH), 8.03 (d, J = 8,8 Hz, 2H, H_3), 6.96 (d, J = 9.2 Hz, 2H, H_2), 4.36 (s, 2H, H_4), 3.87 (s, 3H, H_1), 1.27 (s, 6H, H_5). ^{13}C NMR (100 MHz, CD_2Cl_2) δ 168.4 (C), 164.5 (C), 132.5 (CH), 122.4 (C), 114.3 (CH), 82.1 (C), 67.0 (CH_2), 56.1 (CH_3), 21.7 (CH_3). IR (ATR-FTIR), cm^{-1}: 3342 (w), 2983 (m), 1688 (m), 1255 (m), 1167 (s). HRMS-ESI (m/z): $[M + Na]^+$ calcd for $C_{12}H_{16}NaO_4$, 247.0946; found, 247.0942.

Hydrothioetherification of 2-methylallyl 4-methoxybenzoate (3a) to 2-methyl-2-(phenylthio)propyl 4-methoxybenzoate (4g, Table 2, entry 7)

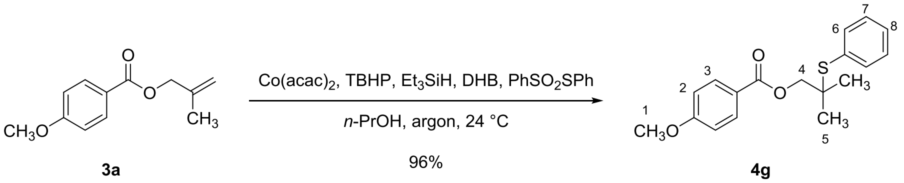

A 10 mL round-bottomed flask fitted with a rubber septum was charged sequentially with 2-methylallyl 4-methoxybenzoate (**3a**, 28.2 mg, 125 μmol, 1 equiv) and cobalt bis(acetylacetonate) (32.1 mg, 125 μmol, 1.00 equiv). The reaction vessel was evacuated and refilled using a balloon of argon. This process was repeated twice. The reaction vessel was protected from light with aluminum foil. *n*-Propanol (400 μL), *S*-phenyl benzenesulfonothioate (78.2 mg, 313 μmol, 2.50 equiv), 1,4-dihydrobenzene (28.7 μL, 313 μmol, 2.50 equiv), a solution of *tert*-butyl hydroperoxide in nonane (~5.5 M, 45.5 μL, 250 μmol, 1.00 equiv), and triethylsilane (400 μL, 2.50 mmol, 10.0 equiv) were added sequentially to the reaction vessel via syringe. The reaction mixture was stirred for 12 h at 24 °C. The product mixture was concentrated and the residue obtained was purified by flash-column chromatography (eluting with 2% ethyl acetate–hexanes initially, grading to 10% ethyl acetate–hexanes, linear gradient) to 2-phenylthio-2-methylpropyl 4-methoxybenzoate (**4g**) as a clear oil (38.1 mg, 96%).

R_f = 0.45 (20% ethyl acetate–hexanes; UV). ^{1}H NMR (400 MHz, CD_2Cl_2) δ 7.98 (d, J = 8.8 Hz, 2H, H_3), 7.58–7.56 (m, 2H, H_7), 7.40–7.32 (m, 3H, 2 × H_6, 1 × H_8), 6.94 (d, J = 8.8 Hz, 2H, H_2), 4.17 (s, 2H, H_4), 3.86 (s, 3H, H_1), 1.36 (s, 6H, H_5). ^{13}C NMR (100 MHz, CD_2Cl_2) δ 166.3 (C), 164.1 (C), 138.2 (CH), 132.1 (CH), 131.7 (C), 129.7 (CH), 129.3 (CH) 123.1 (C), 114.2 (CH), 71.5 (CH_2), 56.0 (CH_3), 48.1 (C), 29.8 (CH_3). IR (ATR-FTIR), cm^{-1}: 2966 (w), 1709 (m), 1605 (m), 1250 (s), 1098 (s). HRMS-ESI (m/z): $[M + Na]^+$ calcd for $C_{18}H_{20}NaSO_3$, 339.1031; found, 339.1035.

Hydroselenation of 2-methylallyl 4-methoxybenzoate (3a) to 2-methyl-2-(phenylselanyl)propyl 4-methoxybenzoate (4h, Table 1, entry 8)

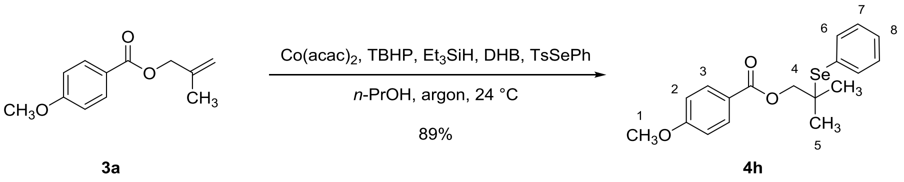

A 10 mL round-bottomed flask fitted with a rubber septum was charged sequentially with 2-methylallyl 4-methoxybenzoate (**3a**, 51.6 mg, 250 μmol, 1 equiv), *Se*-phenyl 4-methylbenzenesulfonoselenoate (195 mg, 625 μmol, 2.50 equiv), and cobalt bis(acetylacetonate) (64.3 mg, 250 μmol, 1.00 equiv). The reaction vessel was evacuated and refilled using a balloon of argon. This process was repeated twice. *n*-Propanol (830 μL), 1,4-dihydrobenzene (86.0 μL, 938 μmol, 3.75 equiv), a solution of *tert*-butyl hydroperoxide in nonane (~5.5 M, 45.5 μL, 250 μmol, 1.00 equiv), and triethylsilane (400 μL, 2.50 mmol, 10.0 equiv) were added sequentially to the reaction vessel via syringe. The reaction mixture was stirred for 4.0 h at 24 °C. The product mixture was filtered through a short column of silica gel and the short column was rinsed with 20% ethyl acetate–hexanes (100 mL). The filtrates were combined and the combined filtrates were concentrated. The residue obtained was purified by flash-column chromatography (eluting with 2% ethyl acetate–hexanes initially, grading to 6% ethyl acetate–hexanes, linear gradient) to 2-methyl-2-(phenylselanyl)propyl 4-methoxybenzoate (**4h**) as a clear oil (83.9 mg, 89%).

R_f = 0.47 (20% ethyl acetate–hexanes; UV, CAM). ^{1}H NMR (400 MHz, CD_2Cl_2) δ 7.98–7.95 (m, 2H, H_3), 7.69–7.66 (m, 2H, H_7), 7.42–7.38 (m, 1H, H_8), 7.34–7.30 (m, 2H, H_6), 6.95–6.92 (m, 2H, H_2), 4.24 (s, 2H, H_4), 3.86 (s, 3H, H_1), 1.46 (s, 6H, H_5). ^{13}C NMR (100 MHz, CD_2Cl_2) δ 166.2 (C), 164.1 (C), 138.9 (CH), 132.1 (CH), 129.4 (CH), 129.4 (CH), 127.4 (C), 123.1 (C), 114.2 (CH), 72.7 (CH_2), 56.0 (CH_3), 45.0 (C), 27.0 (CH_3). IR (ATR-FTIR), cm^{-1}: 2960 (w), 1712 (m), 1606 (m), 1256 (s), 1167 (m). HRMS-ESI (m/z): $[M + H]^+$ calcd for $C_{18}H_{21}SeO_3$, 365.0656; found, 365.0648.

Hydroazidation of 2-methylallyl 4-methoxybenzoate (3a) to 2-azido-2-methylpropyl 4-methoxybenzoate (4i, Table 1 entry 9)

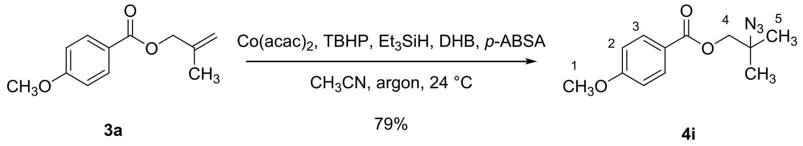

A 10 mL round-bottomed flask fitted with a rubber septum was charged sequentially with 2-methylallyl 4-methoxybenzoate (**3a**, 25.8 mg, 125 μmol, 1 equiv), 4-acetamidobenzenesulfonyl azide (150 mg, 625 μmol, 5.00 equiv), and cobalt bis(acetylacetonate) (32.1 mg, 125 μmol, 1.00 equiv). The reaction vessel was evacuated and refilled using a balloon of argon. This process was repeated twice. Acetonitrile (400 μL), 1,4-dihydrobenzene (11.5 μL, 125 μmol, 1.00 equiv), a solution of *tert*-butyl hydroperoxide in nonane (~5.5 M, 22.8 μL, 125 μmol, 1.00 equiv), and triethylsilane (200 μL, 1.25 mmol, 10.0 equiv) were added sequentially to the reaction vessel via syringe. The reaction mixture was stirred for 1.5 h at 24 °C. The product mixture was filtered through a short column of silica gel and the short column was rinsed with 20% ethyl acetate–hexanes (100 mL). The filtrates were combined and the combined filtrates were concentrated. The residue obtained was purified by flash-column chromatography (eluting with 3% ethyl acetate–hexanes initially, grading to 7% ethyl acetate–hexanes, linear gradient) to 2-azido-2-methylpropyl 4-methoxybenzoate (**4i**) as a clear oil (24.6 mg, 79%).

R_f = 0.42 (20% ethyl acetate–hexanes; UV). ^{1}H NMR (600 MHz, CD_2Cl_2) δ 8.02 (d, J = 9.0 Hz, 2H, H_3), 6.95 (d, J = 9.0 Hz, 2H, H_2), 4.21 (s, 2H, H_4), 3.86 (s, 3H, H_1), 1.37 (s, 6H, H_5). ^{13}C NMR (100 MHz, CD_2Cl_2) δ 166.1 (C), 164.2 (C), 132.2 (CH), 122.6 (C), 114.2 (CH), 71.4 (CH_2), 60.9 (C), 56.0 (CH_3), 23.6 (CH_3). IR (ATR-FTIR), cm^{-1}: 2977 (w), 2095 (m), 1715 (m), 1606 (m), 1255 (s). HRMS-ESI (m/z): $[M + Na]^+$ calcd for $C_{12}H_{15}N_3NaO_3$, 272.1011; found, 272.0998.

Hydrooximation of 2-methylallyl 4-methoxybenzoate (3a) to 3-((benzyloxy)imino)-3-cyano-2,2-dimethylpropyl 4-methoxybenzoate (4j)

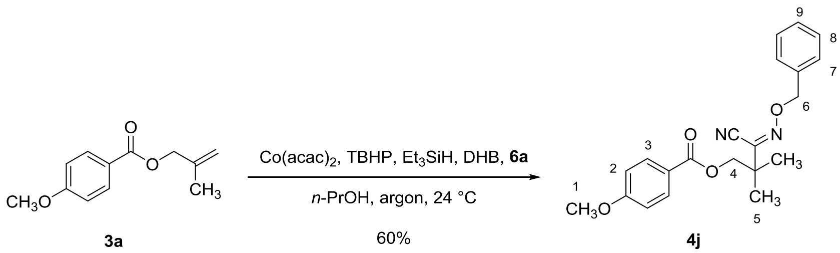

A 10 mL round-bottomed flask fitted with a rubber septum was charged sequentially with 2-methylallyl 4-methoxybenzoate (**3a**, 25.8 mg, 125 μmol, 1 equiv), *N*-(benzyloxy)-1-(phenylsulfonyl)methanimidoyl cyanide (**6a**, 93.9 mg, 313 μmol, 2.50 equiv), and cobalt bis(acetylacetonate) (32.1 mg, 125 μmol, 1.00 equiv). The reaction vessel was evacuated and refilled using a balloon of argon. This process was repeated twice. *n*-Propanol (400 μL), 1,4-dihydrobenzene (43.0 μL, 470 μmol, 3.75 equiv), a solution of *tert*-butyl hydroperoxide in nonane (~5.5 M, 22.8 μL, 125 μmol, 1.00 equiv), and triethylsilane (200 μL, 1.25 mmol, 10.0 equiv) were added sequentially to the reaction vessel via syringe. The reaction mixture was stirred for 8.0 h at 24 °C. The product mixture was filtered through a short column of silica gel and the short column was rinsed with 20% ethyl acetate–hexanes (100 mL). The filtrates were combined and the combined filtrates were concentrated. The residue obtained was purified by flash-column chromatography (eluting with 2% ethyl acetate–hexanes initially, grading to 10% ethyl acetate–hexanes, linear gradient) to 3-((benzyloxy)imino)-3-cyano-2,2-dimethylpropyl 4-methoxybenzoate (**4j**) as a clear oil (27.5 mg, 60%).

R_f = 0.35 (20% ethyl acetate–hexanes; UV, CAM). ^{1}H NMR (600 MHz, CD_2Cl_2) δ 7.92 (d, J = 8.4 Hz, 2H, H_3), 7.36–7.32 (m, 5H, 2 × H_7, 2 × H_8, 1 × H_9), 6.91 (d, J = 8.4 Hz, 2H, H_2), 5.26 (s, 2H, H_6), 4.21 (s, 2H, H_4), 3.86 (s, 3H, H_1), 1.36 (s, 6H, H_5). ^{13}C NMR (100 MHz, CD_2Cl_2) δ 166.1 (C), 164.2 (C), 138.1 (C), 136.6 (C), 132.1 (CH), 129.1 (CH), 129.0 (CH), 128.9 (CH), 122.7 (C), 114.2 (CH), 109.9 (C), 78.7 (CH_2), 70.2 (CH_2), 56.0 (CH_3), 40.5 (C), 23.2 (CH_3). IR (ATR-FTIR), cm^{-1}: 2973 (w), 1713 (m), 1605 (m), 1511 (m), 1254 (s). HRMS-ESI (m/z): $[M + H]^+$ calcd for $C_{21}H_{23}N_2O_4$, 367.1658; found, 367.1653.

Hydrooximation of 2-methylallyl 4-methoxybenzoate (3a) to 3-((benzyloxy)imino)-2,2-dimethylpropyl 4-methoxybenzoate (4k)

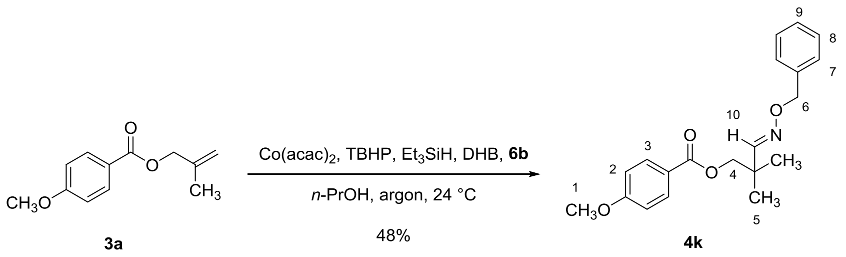

A 10 mL round-bottomed flask fitted with a rubber septum was charged sequentially with 2-methylallyl 4-methoxybenzoate (**3a**, 25.8 mg, 125 μmol, 1 equiv), (phenylsulfonyl)methanal *O*-benzyl oxime (**6b**, 86.0 mg, 313 μmol, 2.50 equiv), and cobalt bis(acetylacetonate) (32.1 mg, 125 μmol, 1.00 equiv). The reaction vessel was evacuated and refilled using a balloon of argon. This process was repeated twice. *n*-Propanol (400 μL), 1,4-dihydrobenzene (43.0 μL, 470 μmol, 3.75 equiv), a solution of *tert*-butyl hydroperoxide in nonane (~5.5 M, 22.8 μL, 125 μmol, 1.00 equiv), and triethylsilane (200 μL, 1.25 mmol, 10.0 equiv) were added sequentially to the reaction vessel via syringe. The reaction mixture was stirred for 6.0 h at 24 °C. The product mixture was filtered through a short column of silica gel and the short column was rinsed with 20% ethyl acetate–hexanes (100 mL). The filtrates were combined and the combined filtrates were concentrated. The residue obtained was purified by flash-column chromatography (eluting with 2% ethyl acetate–hexanes initially, grading to 10% ethyl acetate–hexanes, linear gradient) to 3-((benzyloxy)imino)-2,2-dimethylpropyl 4-methoxybenzoate (**4k**) as a clear oil (20.4 mg, 48%).

R_f = 0.40 (20% ethyl acetate–hexanes; UV, CAM). ^{1}H NMR (500 MHz, CD_2Cl_2) δ 7.96 (d, J = 8.5 Hz, 2H, H_3), 7.47 (s, 1H, H_{10}), 7.35–7.28 (m, 5H, 2 × H_7, 2 × H_8, 1 × H_9), 6.93 (d, J = 8.5 Hz, 2H, H_2), 5.04 (s, 2H, H_6), 4.17 (s, 2H, H_4), 3.86 (s, 3H, H_1), 1.21 (s, 6H, H_5). ^{13}C NMR (125 MHz, CD_2Cl_2) δ 166.4 (C), 164.0 (C), 155.6 (CH), 138.5 (C), 132.0 (CH), 128.8 (CH), 128.7 (CH), 128.2 (CH), 123.1 (C), 114.2 (CH), 76.2 (CH_2), 71.1 (CH_2), 56.0 (CH_3), 38.1 (C), 23.2 (CH_3). IR (ATR-FTIR), cm^{-1}: 2969 (w), 1713 (m), 1607 (m), 1511 (W), 1258 (s). HRMS-ESI (m/z): $[M + Na]^+$ calcd for $C_{20}H_{23}NNaO_4$, 364.1525; found, 364.1517.

Hydropyridylation of 2-methylallyl 4-methoxybenzoate (3a) to 2-methyl-2-(pyridin-4-yl)propyl 4-methoxybenzoate (4l) and 2-methyl-2-(pyridin-2-yl)propyl 4-methoxybenzoate (4la)

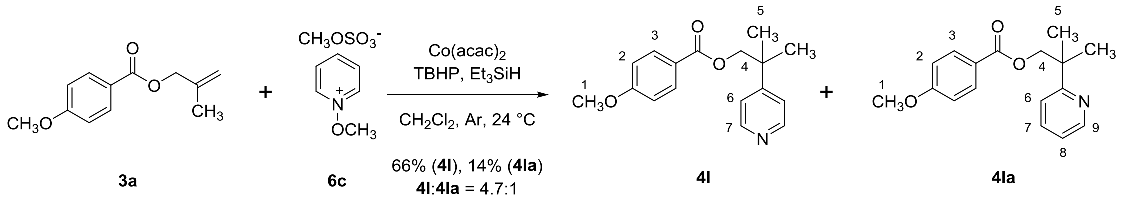

A 25-mL round-bottomed flask fitted with a rubber septum was charged sequentially with 2-methylallyl 4-methoxybenzoate (**3a**, 206 mg, 1.00 mmol, 1 equiv), *N*-methoxy pyridinium methylsulfate (**6c**, 1.11 g, 5.00 mmol, 5.00 equiv), and cobalt bis(acetylacetonate) (257 mg, 1.00 mmol, 1.00 equiv). The reaction vessel was evacuated and refilled using a balloon of argon. This process was repeated twice. Dichloromethane (5.0 mL), triethylsilane (800 µL, 5.00 mmol, 5.00 equiv), and a solution of *tert*-butyl hydroperoxide in nonane (~5.5 M, 182 µL, 1.00 mmol, 1.00 equiv) were added sequentially to the reaction vessel via syringe. The reaction vessel was protected from light with aluminum foil. The reaction mixture was stirred at 24 °C for 16 h. The product mixture was transfer to a separatory funnel that had been charged with ethyl acetate (200 mL). The diluted product mixture was washed with 3 M aqueous ammonium hydroxide solution (3 × 50 mL). The organic layer was isolated and the isolated organic layer was dried over sodium sulfate. The dried solution was filtered and the filtrate was concentrated. The residue obtained was purified by automated flash-column chromatography (eluting with dichloromethane initially, grading to 10% methanol–dichloromethane, linear gradient). The mixture obtained was further purified by flash-column chromatography (eluting with 10% ethyl acetate–10% dichloromethane–10% triethylamine–hexanes) to afford 2-methyl-2-(pyridin-4-yl)propyl 4-methoxybenzoate (**4l**) as a colorless oil (189 mg, 66%). The mixture containing the minor regioisomer was further purified three times by automated flash-column chromatography (eluting with hexanes initially, grading to 33% ether–hexanes, linear gradient) to afford 2-methyl-2-(pyridin-2-yl)propyl 4-methoxybenzoate (**4la**) as a colorless oil (38.9 mg, 14%).

2-Methyl-2-(pyridin-4-yl)propyl 4-methoxybenzoate (**4l**): R_f = 0.55 (10% methanol–dichloromethane; UV). ^{1}H NMR (400 MHz, $CDCl_3$) δ 8.36 (br s, 2H, H_7), 7.85 (d, J = 8.8 Hz, 2H, H_2), 7.33 (d, J = 5.2 Hz, 2H, H_6), 6.87 (d, J = 8.8 Hz, 2H, H_3), 4.35 (s, 2H, H_4), 3.83 (s, 3H, H_1), 1.43 (s, 6H, H_5). ^{13}C NMR (100 MHz, $CDCl_3$) δ 166.0 (C), 163.4 (C), 155.4 (C), 149.9 (CH), 131.5 (CH), 122.2 (C), 121.3 (CH), 113.6 (CH), 72.1 (CH_2), 55.4 (CH_3), 38.5(C), 25.4 (CH_3). IR (ATR-FTIR), cm^{-1}: 2970 (w), 1709 (m), 1605 (m), 1271 (m), 1254 (s), 1166 (m), 1101 (m), 769 (m). HRMS-ESI (m/z): $[M + H]^+$ calcd for $C_{17}H_{20}NO_3$, 286.1443; found, 286.1434.

2-Methyl-2-(pyridin-2-yl)propyl 4-methoxybenzoate (**4la**): R_f = 0.61 (40% ether–hexanes; UV). ^{1}H NMR (400 MHz, $CDCl_3$) δ 8.59 (d, J = 4.8 Hz, 1H, H_9), 7.85 (d, J = 8.4 Hz, 2H, H_2), 7.64 (t, J = 7.4 Hz, 1H, H_7), 7.38 (d, J = 8.4 Hz, 1H, H_6), 7.12 (dd, J = 6.4, 5.2 Hz, 1H, H_8), 6.86 (d, J = 8.4 Hz, 2H, H_3), 4.50 (s, 2H, H_4), 3.82 (s, 3H, H_1), 1.48 (s, 6H, H_5). ^{13}C NMR (100 MHz, $CDCl_3$) δ 166.1 (C), 165.3 (C), 163.2 (C), 148.9 (CH), 136.2 (CH), 131.5 (CH), 122.7 (C), 121.2 (CH), 120.0 (CH), 113.5 (CH), 72.5 (CH_2), 55.4 (CH_3), 41.2 (C), 25.1 (CH_3). IR (ATR-FTIR), cm^{-1}: 2971 (w), 1709 (s), 1606 (s), 1512 (m), 1273 (m), 1256 (s), 1167 (s), 1102 (w), 1029 (s), 770 (m). HRMS-ESI (m/z): $[M + H]^+$ calcd for $C_{17}H_{20}NO_3$, 286.1443; found, 286.1440.

Hydrofunctionalization of 2-methylallyl 4-methoxybenzoate (3a) to the manganese(I) complex 4m

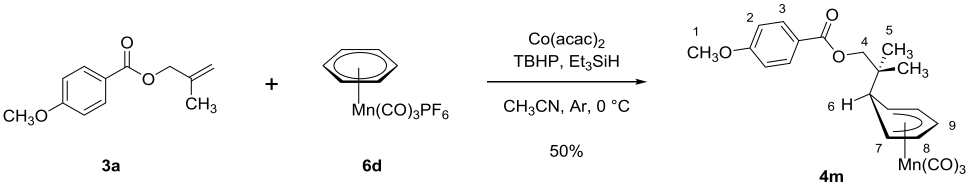

3a **6d** **4m**

A 10 mL round-bottomed flask fitted with a rubber septum was charged sequentially with 2-methylallyl 4-methoxybenzoate (**3a**, 51.5 mg, 250 μmol, 1 equiv), (η^6-benzene) manganese tricarbonyl hexafluorophosphate (**6d**, 90.5 mg, 250 μmol, 1.00 equiv), and cobalt bis(acetylacetonate) (64.3 mg, 250 μmol, 1.00 equiv). The reaction vessel was evacuated and refilled using a balloon of argon. This process was repeated twice. Acetonitrile (1.25 mL) was added to the reaction vessel via syringe and the reaction vessel was cooled to 0 °C. Triethylsilane (200 μL, 1.25 mmol, 5.00 equiv) and a solution of *tert*-butyl hydroperoxide in nonane (~5.5 M, 45.5 μL, 250 μmol, 1.00 equiv) were added sequentially to the reaction vessel via syringe. The reaction vessel was protected from light with aluminum foil. The reaction mixture was stirred for 12 h at 0 °C. The product mixture was concentrated to dryness and the residue obtained was purified by automated flash-column chromatography (eluting with hexanes initially, grading to 33% ethyl acetate–hexanes, linear gradient) to afford 4-(1-((4-methoxybenzoyl)oxy)-2-methylpropan-2-yl)cyclohexa-2,5-dien-1-yl tricarbonyl manganese(I) (**4m**) as a pale yellow oil (53.6 mg, 50%).

R_f = 0.29 (33% ether–hexanes; UV). ^{1}H NMR (400 MHz, $CDCl_3$) δ 7.97 (d, J = 8.4 Hz, 2H, H_2), 6.93 (d, J = 8.4 Hz, 2H, H_3), 5.68 (t, J = 4.6 Hz, 1H, H_6), 4.92 (t, J = 6.0 Hz, 2H, H_7), 3.87 (s, 3H, H_1), 3.82 (s, 2H, H_4), 3.21 (t, J = 6.0 Hz, 2H, H_8), 2.80 (t, J = 5.4 Hz, 1H, H_9), 0.69 (s, 6H, H_5). ^{13}C NMR (100 MHz, $CDCl_3$) δ 222.8 (C), 166.2 (C), 163.4 (C), 131.5 (CH), 122.6 (C), 113.7 (CH), 97.8 (CH), 78.8 (CH), 69.4 (CH_2), 56.2 (CH_3), 55.5 (CH), 42.5 (CH), 41.4 (C), 20.7 (CH_3). IR (ATR-FTIR), cm^{-1}: 2964 (w), 2961 (w), 2008 (s), 1907 (s), 1708 (m), 1606 (m), 1253 (s), 1166 (m), 1101 (m), 1029 (m), 658 (m), 636 (s), 613 (s). HRMS-ESI (m/z): $[M + H]^+$ calcd for $C_{21}H_{22}MnO_6$, 425.0797; found, 425.0772.

Hydrodiazenation of 2-methylallyl 4-methoxybenzoate (3a) to 2-((4-methoxyphenyl)diazenyl)-2-methylpropyl 4-methoxybenzoate (7a)

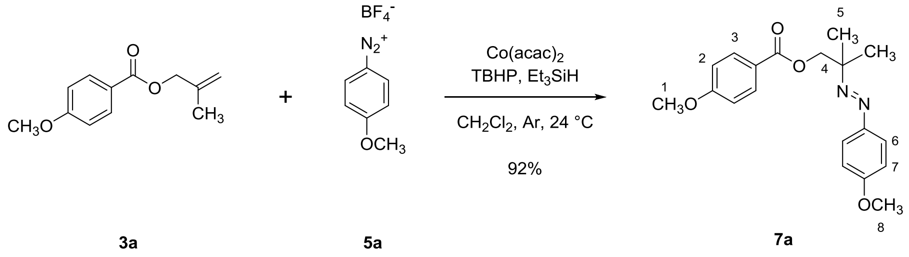

A 25 mL round-bottomed flask fitted with a rubber septum was charged sequentially with 2-methylallyl 4-methoxybenzoate (**3a**, 206 mg, 1.00 mmol, 1 equiv), 4-methoxybenzenediazonium tetrafluoroborate (**5a**, 333 mg, 1.50 mmol, 1.50 equiv), and cobalt bis(acetylacetonate) (257 mg, 1.00 mmol, 1.00 equiv). The reaction vessel was evacuated and refilled using a balloon of argon. This process was repeated twice. Dichloromethane (5.0 mL), triethylsilane (998 μL, 6.25 mmol, 6.25 equiv), and a solution of *tert*-butyl hydroperoxide in nonane (~5.5 M, 182 μL, 1.00 mmol, 1.00 equiv) were added sequentially to the reaction vessel via syringe. The reaction vessel was protected from light with aluminum foil. The reaction mixture was stirred at 24 °C for 30 min. The product mixture was concentrated to dryness and the residue obtained was purified by automated flash-column chromatography (eluting with hexanes initially, grading to 25% ether–hexanes, linear gradient) to afford 2-((4-methoxyphenyl)diazenyl)-2-methylpropyl 4-methoxybenzoate (**7a**) as a bright yellow oil (316 mg, 92%).

R_f = 0.33 (25% ether–hexanes; UV). ^{1}H NMR (400 MHz, $CDCl_3$) δ 7.95 (d, J = 8.8 Hz, 2H, H_2), 7.69 (d, J = 8.4 Hz, 2H, H_6), 6.94 (d, J = 8.8 Hz, 2H, H_7), 6.88 (d, J = 8.8 Hz, 2H, H_3), 4.60 (s, 2H, H_4), 3.85 (s, 3H, H_8), 3.83 (s, 3H, H_1), 1.41 (s, 6H, H_5). ^{13}C NMR (100 MHz, $CDCl_3$) δ 166.2 (C), 163.3 (C), 161.5 (C), 146.3 (C), 131.6 (CH), 123.9 (CH), 122.7 (C), 113.9 (CH), 113.6 (CH), 70.1 (CH_2), 69.1 (C), 55.5 (CH_3), 55.4 (CH_3), 22.4 (CH_3). IR (ATR-FTIR), cm^{-1}: 2952 (w), 1719 (s), 1607 (m), 1512 (w), 1436 (w), 1365 (w), 1275 (s), 1257 (s), 1167 (m), 1103 (m), 1030 (w), 848 (w), 770 (m). HRMS-ESI (m/z): $[M + H]^+$ calcd for $C_{19}H_{23}N_2O_4$, 343.1658; found, 343.1661.

Hydrodiazenation of allyl 4-methoxybenzoate (3b) to 2-((4-methoxyphenyl)diazenyl)propyl 4-methoxybenzoate (7b)

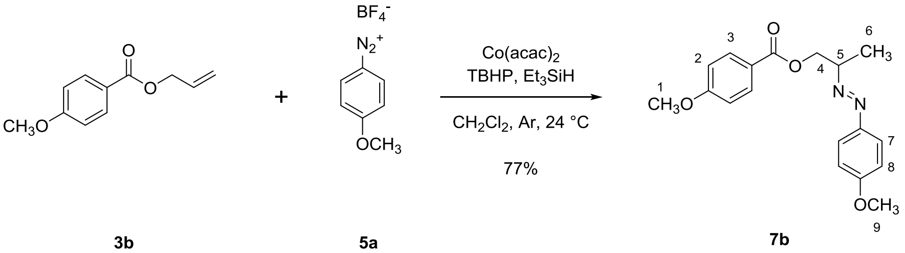

3b **5a** **7b**

A 10 mL round-bottomed flask fitted with a rubber septum was charged sequentially with allyl 4-methoxybenzoate (**3b**, 48.1 mg, 250 μmol, 1 equiv), 4-methoxybenzenediazonium tetrafluoroborate (**5a**, 83.2 mg, 375 μmol, 1.50 equiv), and cobalt bis(acetylacetonate) (64.3 mg, 250 μmol, 1.00 equiv). The reaction vessel was evacuated and refilled using a balloon of argon. This process was repeated twice. Dichloromethane (1.3 mL), triethylsilane (250 μL, 1.56 mmol, 6.25 equiv), and a solution of *tert*-butyl hydroperoxide in nonane (~5.5 M, 45.5 μL, 250 μmol, 1.00 equiv) were added sequentially to the reaction vessel via syringe. The reaction vessel was protected from light with aluminum foil. The reaction mixture was stirred at 24 °C for 30 min. The product mixture was concentrated to dryness and the residue obtained was purified by automated flash-column chromatography (eluting with hexanes initially, grading to 20% ethyl acetate–hexanes, linear gradient) to afford 2-((4-methoxyphenyl)diazenyl)propyl 4-methoxybenzoate (**7b**) as a bright yellow oil (63.2 mg, 77%).

R_f = 0.45 (20% ethyl acetate–hexanes; UV). ^{1}H NMR (400 MHz, $CDCl_3$) δ 7.92 (d, J = 8.8 Hz, 2H, H_2), 7.69 (d, J = 8.4 Hz, 2H, H_7), 6.95 (d, J = 8.8 Hz, 2H, H_8), 6.87 (d, J = 8.8 Hz, 2H, H_3), 4.75 (dd, J = 11.2, 8.4 Hz, 1H, 1 × H_4), 4.63 (dd, J = 11.2, 4.4 Hz, 1H, 1 × H_4), 4.16–4.10 (m, 1H, H_5), 3.86 (s, 3H, H_1), 3.83 (s, 3H, H_9), 1.42 (d, J = 6.8 Hz, 3H, H_6). ^{13}C NMR (100 MHz, $CDCl_3$) δ 166.1 (C), 163.3 (C), 161.6 (C), 146.2 (C), 131.6 (CH), 124.1 (CH), 122.5 (C), 114.0 (CH), 113.5 (CH), 71.0 (CH), 66.6 (CH_2), 55.5 (CH_3), 55.4 (CH_3), 15.5 (CH_3). IR (ATR-FTIR), cm^{-1}: 2977 (w), 1719 (s), 1606 (m), 1512 (w), 1436 (w), 1315 (w), 1275 (s), 1256 (s), 1167 (m), 1103 (m), 1030 (w), 849 (w), 770 (m). HRMS-ESI (m/z): $[M + H]^+$ calcd for $C_{18}H_{21}N_2O_4$, 329.1501; found, 329.1500.

Hydrodiazenation of prenyl 4-methoxybenzoate (3c) to 3-((4-methoxyphenyl)diazenyl)-3-methylbutyl 4-methoxybenzoate (7c)

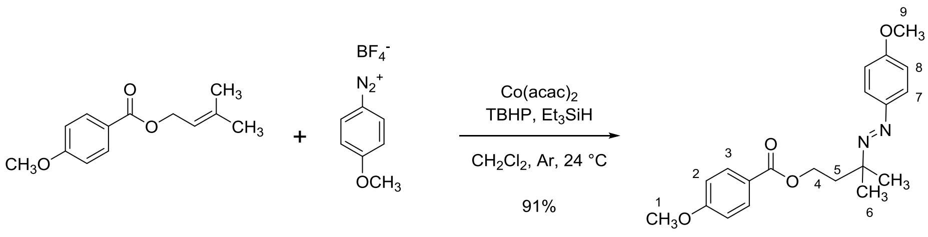

3c **5a** **7c**

A 10 mL round-bottomed flask fitted with a rubber septum was charged sequentially with prenyl 4-methoxybenzoate (**3c**, 48.1 mg, 250 µmol, 1 equiv), 4-methoxybenzenediazonium tetrafluoroborate (**5a**, 83.2 mg, 375 µmol, 1.50 equiv), and cobalt bis(acetylacetonate) (64.3 mg, 250 µmol, 1.00 equiv). The reaction vessel was evacuated and refilled using a balloon of argon. This process was repeated twice. Dichloromethane (1.3 mL), triethylsilane (250 µL, 1.56 mmol, 6.25 equiv), and a solution of *tert*-butyl hydroperoxide in nonane (~5.5 M, 45.5 µL, 250 µmol, 1.00 equiv) were added sequentially to the reaction vessel via syringe. The reaction vessel was protected from light with aluminum foil. The reaction mixture was stirred at 24 °C for 30 min. The product mixture was concentrated to dryness and the residue obtained was purified by automated flash-column chromatography (eluting with hexanes initially, grading to 20% ethyl acetate–hexanes, linear gradient) to afford 3-((4-methoxyphenyl)diazenyl)-3-methylbutyl 4-methoxybenzoate (**7c**) as a bright yellow oil (63.2 mg, 91%).

R_f = 0.45 (20% ethyl acetate–hexanes; UV). ^{1}H NMR (500 MHz, $CDCl_3$) δ 7.93 (d, J = 8.5 Hz, 2H, H_2), 7.66 (d, J = 8.5 Hz, 2H, H_7), 6.92 (d, J = 8.5 Hz, 2H, H_8), 6.85 (d, J = 8.5 Hz, 2H, H_3), 4.41 (t, J = 7.0 Hz, 2H, H_4), 3.85 (s, 3H, H_1), 3.84 (s, 3H, H_9), 2.28 (t, J = 7.0 Hz, 2H, H_5), 1.37 (s, 6H, H_6). ^{13}C NMR (125 MHz, $CDCl_3$) δ 166.3 (C), 163.2 (C), 161.3 (C), 146.2 (C), 131.5 (CH), 123.8 (CH), 122.8 (C), 113.9 (CH), 113.5 (CH), 68.3 (C), 61.5 (CH_2), 55.5 (CH_3), 55.4 (CH_3), 39.2 (CH_2), 25.3 (CH_3). IR (ATR-FTIR), cm^{-1}: 2966 (w), 1708 (m), 1605 (m), 1587 (w), 1152 (m), 1461 (w), 1315 (w), 1274 (m), 1247 (s), 1166 (s), 1101 (s), 1028 (s), 836 (s), 769 (s), 696 (m), 613 (m). HRMS-ESI (m/z): $[M + H]^+$ calcd for $C_{20}H_{25}N_2O_4$, 357.1814; found, 357.1815.

Hydrodiazenation of 2-methylallyl 4-methoxybenzoate (3a) to 2-((4-fluorophenyl)diazenyl)-2-methylpropyl 4-methoxybenzoate (7d)

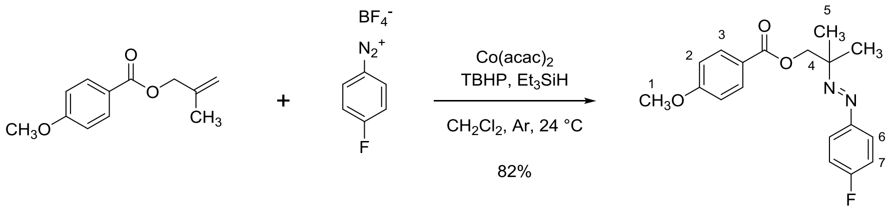

3a **5b** **7d**

A 10 mL round-bottomed flask fitted with a rubber septum was charged sequentially with allyl 4-methoxybenzoate (**3a**, 51.6 mg, 250 μmol, 1 equiv), 4-fluorobenzenediazonium tetrafluoroborate (**5b**, 78.7 mg, 375 μmol, 1.50 equiv), and cobalt bis(acetylacetonate) (64.3 mg, 250 μmol, 1.00 equiv). The reaction vessel was evacuated and refilled using a balloon of argon. This process was repeated twice. Dichloromethane (1.3 mL), triethylsilane (250 μL, 1.56 mmol, 6.25 equiv), and a solution of *tert*-butyl hydroperoxide in nonane (~5.5 M, 45.5 μL, 250 μmol, 1.00 equiv) were added sequentially to the reaction vessel via syringe. The reaction vessel was protected from light with aluminum foil. The reaction mixture was stirred at 24 °C for 30 min. The product mixture was concentrated to dryness and the residue obtained was purified by automated flash-column chromatography (eluting with hexanes initially, grading to 20% ethyl acetate–hexanes, linear gradient) to afford 2-((4-fluorophenyl)diazenyl)-2-methylpropyl 4-methoxybenzoate (**7d**) as a bright yellow oil (67.9 mg, 82%).

R_f = 0.45 (20% ethyl acetate–hexanes; UV). ^{1}H NMR (500 MHz, $CDCl_3$) δ 7.93 (d, J = 8.0 Hz, 2H, H_2), 7.70 (dd, J = 8.0, 5.5 Hz, 2H, H_6), 7.12 (t, J = 8.5 Hz, 2H, H_7), 6.89 (d, J = 8.0 Hz, 2H, H_3), 4.61 (s, 2H, H_4), 3.84 (s, 3H, H_1), 1.42 (s, 6H, H_5). ^{13}C NMR (125 MHz, $CDCl_3$) δ 166.1 (C), 164.0 (d, J = 249.0 Hz, C), 163.4 (C), 148.5 (d, J = 3.0 Hz, C), 131.6 (CH), 124.1 (d, J = 8.8 Hz, CH), 122.6 (CH), 115.7 (d, J = 22.6 Hz, CH), 113.6 (C), 69.9 (CH_2), 69.7 (C), 55.4 (CH_3), 22.3 (CH_3). ^{19}F NMR (470 MHz, $CDCl_3$) δ –110.8. IR (ATR-FTIR), cm^{-1}: 2977 (w), 1714 (m), 1607 (m), 1152(w), 1316 (w) 1274 (m), 1256 (s), 1167 (s), 1102 (m), 844 (m), 769 (m). HRMS-ESI (m/z): $[M + H]^+$ calcd for $C_{18}H_{20}FN_2O_3$, 331.1458; found, 331.1451.

Hydrodiazenation of 2-methylallyl 4-methoxybenzoate (3a) to 2-((4-(trifluoromethyl)phenyl)diazenyl)-2-methylpropyl 4-methoxybenzoate (7e)

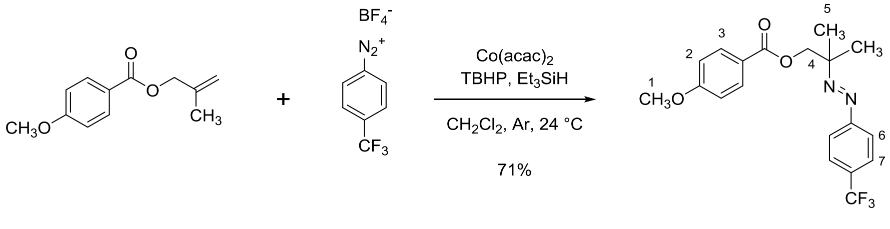

A 10 mL round-bottomed flask fitted with a rubber septum was charged sequentially with allyl 4-methoxybenzoate (**3a**, 51.6 mg, 250 μmol, 1 equiv), 4-(trifluoromethyl)benzenediazonium tetrafluoroborate (**5c**, 97.5 mg, 375 μmol, 1.50 equiv), and cobalt bis(acetylacetonate) (64.3 mg, 250 μmol, 1.00 equiv). The reaction vessel was evacuated and refilled using a balloon of argon. This process was repeated twice. Dichloromethane (1.3 mL), triethylsilane (250 μL, 1.56 mmol, 6.25 equiv), and a solution of *tert*-butyl hydroperoxide in nonane (~5.5 M, 45.5 μL, 250 μmol, 1.00 equiv) were added sequentially to the reaction vessel via syringe. The reaction vessel was protected from light with aluminum foil. The reaction mixture was stirred at 24 °C for 30 min. The product mixture was concentrated to dryness and the residue obtained was purified by automated flash-column chromatography (eluting with hexanes initially, grading to 12% ethyl acetate–hexanes, linear gradient) to afford 2-((4-(trifluoromethyl)phenyl)diazenyl)-2-methylpropyl 4-methoxybenzoate (**7e**) as a bright yellow oil (67.5 mg, 71%).

R_f = 0.43 (33% ethyl acetate–hexanes; UV). ^{1}H NMR (500 MHz, $CDCl_3$) δ 7.93 (d, J = 9.0 Hz, 2H, H_2), 7.75 (d, J = 8.0 Hz, 2H, H_6), 7.71 (d, J = 8.5 Hz, 2H, H_7), 6.89 (d, J = 9.0 Hz, 2H, H_3), 4.64 (s, 2H, H_4), 3.84 (s, 3H, H_1), 1.44 (s, 6H, H_5). ^{13}C NMR (125 MHz, $CDCl_3$) δ 166.0 (C), 163.4 (C), 154.0 (C), 131.9 (q, J = 21.7 Hz, CH), 131.6 (CH), 126.2 (q, J = 4.2 Hz, CH), 123.9 (q, J = 270.8 Hz, C), 122.4 (C), 122.3 (CH), 113.6 (C), 70.7 (C), 69.6 (CH_2), 55.4 (CH_3), 22.2 (CH_3). ^{19}F NMR (470 MHz, $CDCl_3$) δ –62.6. IR (ATR-FTIR), cm^{-1}: 1715 (m), 1678 (w), 1508 (m), 1512 (w), 1323 (s), 1302 (m), 1257 (s), 1167 (s), 1110 (s), 1064 (m), 845 (m), 770 (w). HRMS-ESI (m/z): $[M + H]^+$ calcd for $C_{19}H_{20}F_3N_2O_3$, 381.1426; found, 381.1429.

Hydrodiazenation of 2-methylallyl 4-methoxybenzoate (3a) to 2-((4-bromophenyl)diazenyl)-2-methylpropyl 4-methoxybenzoate (7f)

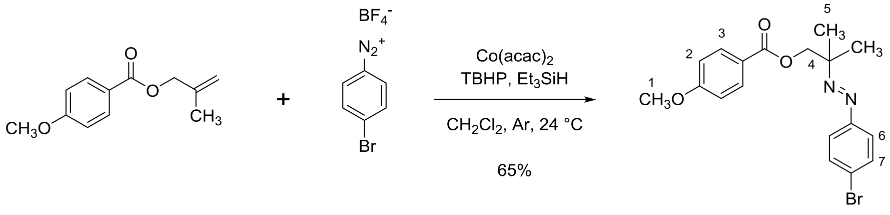

A 10 mL round-bottomed flask fitted with a rubber septum was charged sequentially with allyl 4-methoxybenzoate (**3a**, 51.6 mg, 250 μmol, 1 equiv), 4-bromobenzenediazonium tetrafluoroborate (**5d**, 102 mg, 375 μmol, 1.50 equiv), and cobalt bis(acetylacetonate) (64.3 mg, 250 μmol, 1.00 equiv). The reaction vessel was evacuated and refilled using a balloon of argon. This process was repeated twice. Dichloromethane (1.3 mL), triethylsilane (250 μL, 1.56 mmol, 6.25 equiv), and a solution of *tert*-butyl hydroperoxide in nonane (~5.5 M, 45.5 μL, 250 μmol, 1.00 equiv) were added sequentially to the reaction vessel via syringe. The reaction vessel was protected from light with aluminum foil. The reaction mixture was stirred at 24 °C for 30 min. The product mixture was concentrated to dryness and the residue obtained was purified by automated flash-column chromatography (eluting with hexanes initially, grading to 12% ethyl acetate–hexanes, linear gradient) to afford 2-((4-bromophenyl)diazenyl)-2-methylpropyl 4-methoxybenzoate (**7f**) as a bright yellow oil (63.2 mg, 65%).

R_f = 0.42 (33% ethyl acetate–hexanes; UV). ^{1}H NMR (400 MHz, $CDCl_3$) δ 7.92 (d, J = 8.8 Hz, 2H, H_2), 7.60–7.53 (m, 4H, 2 × H_6, 2 × H_7), 6.88 (d, J = 8.8 Hz, 2H, H_3), 4.61 (s, 2H, H_4), 3.84 (s, 3H, H_1), 1.42 (s, 6H, H_5). ^{13}C NMR (100 MHz, $CDCl_3$) δ 166.0 (C), 163.4 (C), 150.8 (C), 132.1 (CH), 131.6 (CH), 124.7 (C), 123.7 (CH), 122.5 (C), 113.6 (CH), 70.1 (CH_2), 69.8 (C), 55.4 (CH_3), 22.2 (CH_3). IR (ATR-FTIR), cm^{-1}: 2976 (w), 1713 (s), 1606 (m), 1580 (w), 1511 (w), 1470 (w), 1364 (w), 1316 (w), 1273 (m), 1255 (s), 1167 (s), 1102 (m), 1031 (w), 1009 (w), 847 (w), 832 (w), 769 (w). HRMS-ESI (m/z): $[M + H]^+$ calcd for $C_{18}H_{20}Br^{79/81}N_2O_3$, 391.0657/393.0637; found, 391.0652/393.0637.

Hydrodiazenation of 2-methylallyl 4-methoxybenzoate (3a) to 2-((4-(methoxycarbonyl)phenyl)diazenyl)-2-methylpropyl 4-methoxybenzoate (7g)

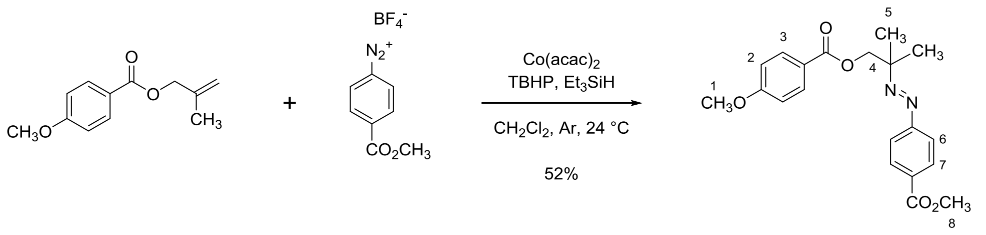

3a **5e** **7g**

A 10 mL round-bottomed flask fitted with a rubber septum was charged sequentially with allyl 4-methoxybenzoate (**3a**, 51.6 mg, 250 μmol, 1 equiv), 4-(methoxycarbonyl)benzenediazonium tetrafluoroborate (**5e**, 93.7 mg, 375 μmol, 1.50 equiv), and cobalt bis(acetylacetonate) (64.3 mg, 250 μmol, 1.00 equiv). The reaction vessel was evacuated and refilled using a balloon of argon. This process was repeated twice. Dichloromethane (1.3 mL), triethylsilane (250 μL, 1.56 mmol, 6.25 equiv), and a solution of *tert*-butyl hydroperoxide in nonane (~5.5 M, 45.5 μL, 250 μmol, 1.00 equiv) were added sequentially to the reaction vessel via syringe. The reaction vessel was protected from light with aluminum foil. The reaction mixture was stirred at 24 °C for 30 min. The product mixture was concentrated to dryness and the residue obtained was purified by automated flash-column chromatography (eluting with hexanes initially, grading to 20% ethyl acetate–hexanes, linear gradient) to afford 2-((4-(methoxycarbonyl)phenyl)diazenyl)-2-methylpropyl 4-methoxybenzoate (**7g**) as a bright yellow oil (47.8 mg, 52%).

R_f = 0.32 (20% ethyl acetate–hexanes; UV). ^{1}H NMR (400 MHz, $CDCl_3$) δ 8.12 (d, J = 8.4 Hz, 2H, H_6), 7.92 (d, J = 8.8 Hz, 2H, H_2), 7.67 (d, J = 8.4 Hz, 2H, H_7), 6.88 (d, J = 8.8 Hz, 2H, H_3), 4.63 (s, 2H, H_4), 3.93 (s, 3H, H_8), 3.83 (s, 3H, H_1), 1.42 (s, 6H, H_5). ^{13}C NMR (100 MHz, $CDCl_3$) δ 166.5 (C), 166.0 (C), 163.4 (C), 154.7 (C), 131.6 (CH), 131.4 (C), 130.5 (CH), 122.4 (C), 121.9 (CH), 113.6 (CH), 70.7 (CH_2), 69.7 (C), 55.4 (CH_3), 52.3 (CH_3), 22.2 (CH_3). IR (ATR-FTIR), cm^{-1}: 2951 (w), 1714 (s), 1606 (m), 1459 (w), 1435 (w), 1315 (w), 1272 (s), 1252 (s), 1165 (s), 1096 (s), 1028 (m), 1013 (m), 847 (m), 767 (s), 696 (m), 634 (w), 612 (w), 510 (w). HRMS-ESI (m/z): $[M + H]^+$ calcd for $C_{20}H_{23}N_2O_5$, 371.1607; found, 371.1605.

Hydrodiazenation of 2-methylallyl 4-methoxybenzoate (3a) to 2-((2-methylphenyl)diazenyl)-2-methylpropyl 4-methoxybenzoate (7h)

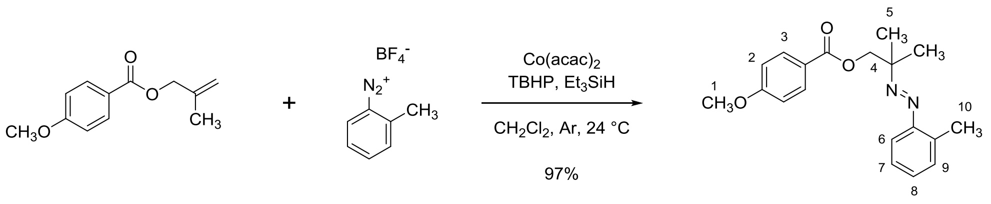

3a **5f** **7h**

A 10 mL round-bottomed flask fitted with a rubber septum was charged sequentially with allyl 4-methoxybenzoate (**3a**, 51.6 mg, 250 μmol, 1 equiv), 2-methylbenzenediazonium tetrafluoroborate (**5f**, 77.2 mg, 375 μmol, 1.50 equiv), and cobalt bis(acetylacetonate) (64.3 mg, 250 μmol, 1.00 equiv). The reaction vessel was evacuated and refilled using a balloon of argon. This process was repeated twice. Dichloromethane (1.3 mL), triethylsilane (250 μL, 1.56 mmol, 6.25 equiv), and a solution of *tert*-butyl hydroperoxide in nonane (~5.5 M, 45.5 μL, 250 μmol, 1.00 equiv) were added sequentially to the reaction vessel via syringe. The reaction vessel was protected from light with aluminum foil. The reaction mixture was stirred at 24 °C for 30 min. The product mixture was concentrated to dryness and the residue obtained was purified by automated flash-column chromatography (eluting with hexanes initially, grading to 20% ethyl acetate–hexanes, linear gradient) to afford 2-((2-methylphenyl)diazenyl)-2-methylpropyl 4-methoxybenzoate (**7h**) as a bright yellow oil (79.5 mg, 97%).

R_f = 0.37 (20% ethyl acetate–hexanes; UV). ^{1}H NMR (500 MHz, CD_2Cl_2) δ 7.94 (d, J = 8.0 Hz, 2H, H_2), 7.33–7.26 (m, 3H, 1 × H_6, 1 × H_7, 1 × H_9), 7.22–7.17 (m, 1H, 1 × H_8), 6.90 (d, J = 8.0 Hz, 2H, H_3), 4.61 (s, 2H, H_4), 3.84 (s, 3H, H_1), 2.56 (s, 3H, H_{10}), 1.44 (s, 6H, H_5). ^{13}C NMR (125 MHz, CD_2Cl_2) δ 165.8 (C), 163.4 (C), 150.4 (C), 136.2 (C), 131.4 (CH), 130.9 (CH), 130.0 (CH), 126.2 (CH), 122.6 (C), 115.5 (CH), 113.5 (CH), 70.2 (CH_2), 69.7 (C), 55.4 (CH_3), 22.1 (CH_3), 16.8 (CH_3). IR (ATR-FTIR), cm^{-1}: 2975 (w), 1714 (s), 1607 (m), 1512 (w), 1316 (w), 1274 (m), 1256 (s), 1167 (s), 1102 (m), 1031 (w), 847 (w), 769 (m), 696 (w). HRMS-ESI (m/z): $[M + H]^+$ calcd for $C_{19}H_{23}N_2O_3$, 327.1709; found, 327.1704.

Hydrodiazenation of 2-methylallyl 4-methoxybenzoate (3a) to 2-((3-phenoxyphenyl)diazenyl)-2-methylpropyl 4-methoxybenzoate (7i)

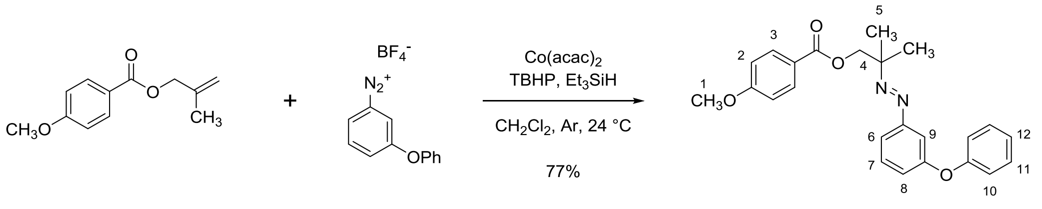

3a **5g** **7i**

A 10 mL round-bottomed flask fitted with a rubber septum was charged sequentially with allyl 4-methoxybenzoate (**3a**, 51.6 mg, 250 μmol, 1 equiv), 3-phenoxybenzenediazonium tetrafluoroborate (**5g**, 107 mg, 375 μmol, 1.50 equiv), and cobalt bis(acetylacetonate) (64.3 mg, 250 μmol, 1.00 equiv). The reaction vessel was evacuated and refilled using a balloon of argon. This process was repeated twice. Dichloromethane (1.3 mL), triethylsilane (250 μL, 1.56 mmol, 6.25 equiv), and a solution of *tert*-butyl hydroperoxide in nonane (~5.5 M, 45.5 μL, 250 μmol, 1.00 equiv) were added sequentially to the reaction vessel via syringe. The reaction vessel was protected from light with aluminum foil. The reaction mixture was stirred at 24 °C for 30 min. The product mixture was concentrated to dryness and the residue obtained was purified by automated flash-column chromatography (eluting with hexanes initially, grading to 12% ethyl acetate–hexanes, linear gradient) to afford 2-((3-phenoxyphenyl)diazenyl)-2-methylpropyl 4-methoxybenzoate (**7i**) as a bright yellow oil (78.1 mg, 77%).

R_f = 0.32 (20% ethyl acetate–hexanes; UV). ^{1}H NMR (500 MHz, $CDCl_3$) δ 7.93 (d, J = 9.0 Hz, 2H, H_2), 7.44–7.38 (m, 2H, 1 × H_8, 1 × H_9), 7.37–7.32 (m, 3H, 1 × H_6, 2 × H_{11}), 7.13 (t, J = 5.0 Hz, 1H, H_{12}), 7.09–7.01 (m, 3H, 1 × H_7, 2 × H_{10}), 6.88 (d, J = 9.0 Hz, 2H, H_3), 4.59 (s, 2H, H_4), 3.85 (s, 3H, H_1), 1.41 (s, 6H, H_5). ^{13}C NMR (125 MHz, $CDCl_3$) δ 166.1 (C), 163.3 (C), 157.9 (C), 156.8 (C), 153.6 (C), 131.5 (CH), 123.0 (CH), 129.8 (CH), 123.5 (CH), 122.6 (C), 120.5 (CH), 119.0 (CH), 117.3 (CH), 113.6 (CH), 112.1 (CH), 70.0 (CH_2), 69.8 (C), 55.4 (CH_3), 22.2 (CH_3). IR (ATR-FTIR), cm^{-1}: 2974 (w), 1713 (s), 1606 (s), 1586 (m), 1511 (m), 1490 (m), 1473 (m), 1315 (w), 1254 (s), 1209 (w), 1167 (s), 1102 (m), 1030 (w), 847 (w), 769 (m), 694 (m). HRMS-ESI (m/z): $[M + H]^+$ calcd for $C_{24}H_{25}N_2O_4$, 405.1814; found, 405.1814.

Hydrodiazenation of 2-methylallyl 4-methoxybenzoate (3a) to 2-((3,4-methylenedioxyphenyl)diazenyl)-2-methylpropyl 4-methoxybenzoate (7j)

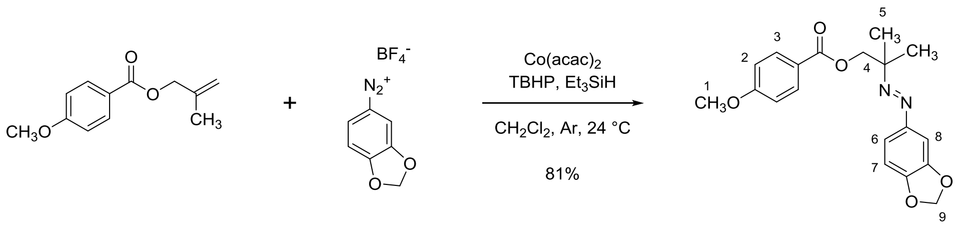

3a **5h** **7j**

A 10 mL round-bottomed flask fitted with a rubber septum was charged sequentially with allyl 4-methoxybenzoate (**3a**, 51.6 mg, 250 μmol, 1 equiv), 3,4-methylenedioxybenzenediazonium tetrafluoroborate (**5h**, 88.5 mg, 375 μmol, 1.50 equiv), and cobalt bis(acetylacetonate) (64.3 mg, 250 μmol, 1.00 equiv). The reaction vessel was evacuated and refilled using a balloon of argon. This process was repeated twice. Dichloromethane (1.3 mL), triethylsilane (250 μL, 1.56 mmol, 6.25 equiv), and a solution of *tert*-butyl hydroperoxide in nonane (~5.5 M, 45.5 μL, 250 μmol, 1.00 equiv) were added sequentially to the reaction vessel via syringe. The reaction vessel was protected from light with aluminum foil. The reaction mixture was stirred at 24 °C for 30 min. The product mixture was concentrated to dryness and the residue obtained was purified by automated flash-column chromatography (eluting with hexanes initially, grading to 12% ethyl acetate–hexanes, linear gradient) to afford 2-((3,4-methylenedioxyphenyl)diazenyl)-2-methylpropyl 4-methoxybenzoate (**7j**) as a bright yellow oil (72.4 mg, 81%).

R_f = 0.34 (20% ethyl acetate–hexanes; UV). ^{1}H NMR (500 MHz, $CDCl_3$) δ 7.94 (d, J = 9.0 Hz, 2H, H_2), 7.38 (d, J = 9.5 Hz, 1H, H_6), 7.18 (s, 1H, H_8), 6.89–6.87 (m, 3H, 1 × H_7, 2 × H_3), 6.01 (s, 2H, H_9), 4.58 (s, 2H, H_4), 3.84 (s, 3H, H_1), 1.40 (s, 6H, H_5). ^{13}C NMR (125 MHz, $CDCl_3$) δ 166.1 (C), 163.3 (C), 149.6 (C), 148.5 (C), 147.8 (C), 131.6 (CH), 122.6 (C), 121.7 (CH), 113.6 (CH), 107.7 (CH), 101.7 (CH_2), 99.1 (CH), 70.0 (CH_2), 69.1 (C), 55.4 (CH_3), 22.4 (CH_3). IR (ATR-FTIR), cm^{-1}: 2974 (w), 1710 (s), 1606 (s), 1511 (m), 1473 (s), 1364 (w), 1473 (s), 1316 (m), 1250 (s), 1165 (s), 1100 (s), 1031 (s), 931 (m), 846 (m), 810 (m), 769 (s), 634 (w), 612 (m), 526 (w). HRMS-ESI (m/z): $[M + H]^+$ calcd for $C_{19}H_{21}N_2O_5$, 357.1450; found, 357.1451.

Hydrodiazenation of 2-methylallyl 4-methoxybenzoate (3a) to 2-((1-naphthyl)diazenyl)-2-methylpropyl 4-methoxybenzoate (7k)

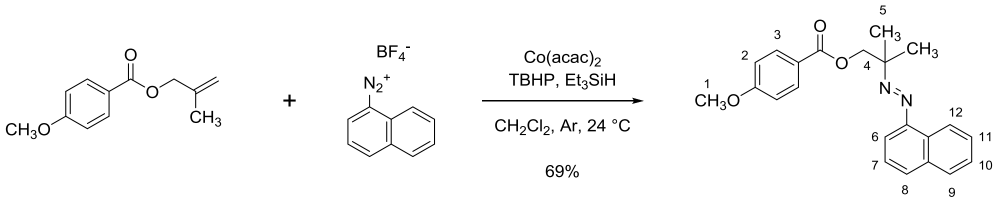

3a **5i** **7k**

A 10 mL round-bottomed flask fitted with a rubber septum was charged sequentially with allyl 4-methoxybenzoate (**3a**, 51.6 mg, 250 μmol, 1 equiv), 1-naphthylenediazonium tetrafluoroborate (**5i**, 90.7 mg, 375 μmol, 1.50 equiv), and cobalt bis(acetylacetonate) (64.3 mg, 250 μmol, 1.00 equiv). The reaction vessel was evacuated and refilled using a balloon of argon. This process was repeated twice. Dichloromethane (1.3 mL), triethylsilane (250 μL, 1.56 mmol, 6.25 equiv), and a solution of *tert*-butyl hydroperoxide in nonane (~5.5 M, 45.5 μL, 250 μmol, 1.00 equiv) were added sequentially to the reaction vessel via syringe. The reaction vessel was protected from light with aluminum foil. The reaction mixture was stirred at 24 °C for 30 min. The product mixture was concentrated to dryness and the residue obtained was purified by automated flash-column chromatography (eluting with hexanes initially, grading to 12% ethyl acetate–hexanes, linear gradient) to afford 2-((1-naphthyl)diazenyl)-2-methylpropyl 4-methoxybenzoate (**7k**) as a bright orange oil (62.3 mg, 69%).

R_f = 0.31 (20% ethyl acetate–hexanes; UV). ^{1}H NMR (500 MHz, $CDCl_3$) δ 8.67–8.65 (m, 1H, H_{12}), 7.96–7.89 (m, 4H, 2 × H_2, 1 × H_6, 1 × H_8), 7.59–7.53 (m, 2H, 1 × H_7, 1 × H_{11}), 7.50 (t, J = 7.8 Hz, 1H, H_{10}), 7.44 (d, J = 9.0 Hz, 1H, H_9), 6.86 (d, J = 8.5 Hz, 2H, H_3), 4.72 (s, 2H, H_4), 3.83 (s, 3H, H_1), 1.54 (s, 6H, H_5). ^{13}C NMR (125 MHz, $CDCl_3$) δ 166.1 (C), 163.4 (C), 147.6 (C), 134.1 (C), 131.6 (CH), 130.4 (CH), 130.3 (C), 127.8 (CH), 126.6 (CH), 126.4 (CH), 125.6 (CH), 123.2 (CH), 122.5 (C), 113.6 (CH), 111.8 (CH), 70.9 (CH_2), 69.8 (C), 55.4 (CH_3), 22.5 (CH_3). IR (ATR-FTIR), cm^{-1}: 2977 (w), 1711 (s), 1606 (s), 1511 (m), 1459 (w), 1364 (w), 1316 (w), 123 (s), 1165 (s), 1100 (s), 1029 (m), 1010 (w), 846 (w), 801 (m), 769 9s), 696 (w), 612 (m). HRMS-ESI (m/z): $[M + H]^+$ calcd for $C_{22}H_{23}N_2O_3$, 363.1709; found, 363.1710.

Attempted hydrodiazenation of 2-methylallyl 4-methoxybenzoate (3a) to 2-((4-nitrophenyl)diazenyl)-2-methylpropyl 4-methoxybenzoate (7l)

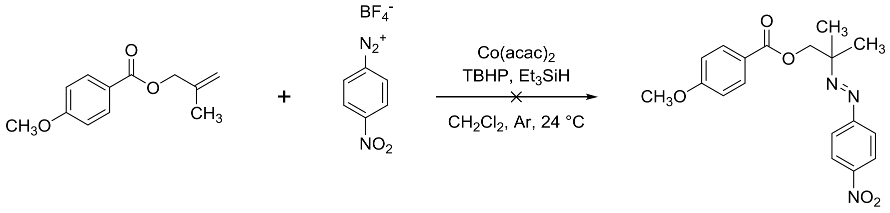

3a **5k** **7l**

A 10 mL round-bottomed flask fitted with a rubber septum was charged sequentially with allyl 4-methoxybenzoate (**3a**, 20.6 mg, 100 μmol, 1 equiv), 4-nitrobenzenediazonium tetrafluoroborate (**5k**, 35.5 mg, 150 μmol, 1.50 equiv), and cobalt bis(acetylacetonate) (25.7 mg, 100 μmol, 1.00 equiv). The reaction vessel was evacuated and refilled using a balloon of argon. This process was repeated twice. Dichloromethane (500 μL), triethylsilane (79.9 μL, 625 μmol, 6.25 equiv), and a solution of *tert*-butyl hydroperoxide in nonane (~5.5 M, 18.2 μL, 100 μmol, 1.00 equiv) were added sequentially to the reaction vessel via syringe. The reaction vessel was protected from light with aluminum foil. The reaction mixture was stirred at 24 °C for 30 min. The product mixture was concentrated to dryness and the residue obtained filtered through a short column of silica gel. The column was rinsed with ether (100 mL). The filtrates were combined and the combined filtrates were concentrated to dryness. ^{1}H NMR analysis of the unpurified mixture showed complex decomposition of **3a**.

Catalog of nuclear magnetic resonance and infrared spectra

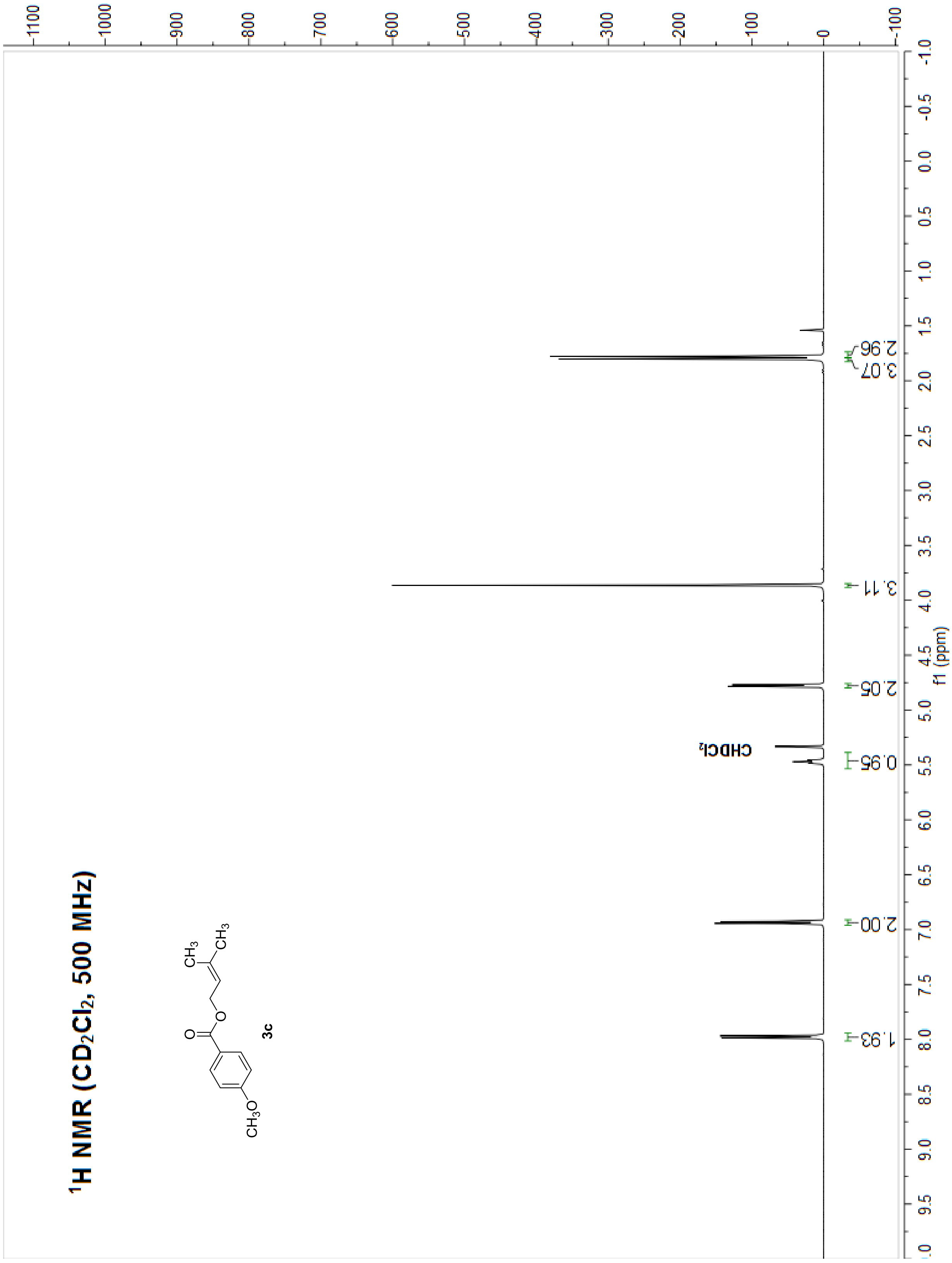

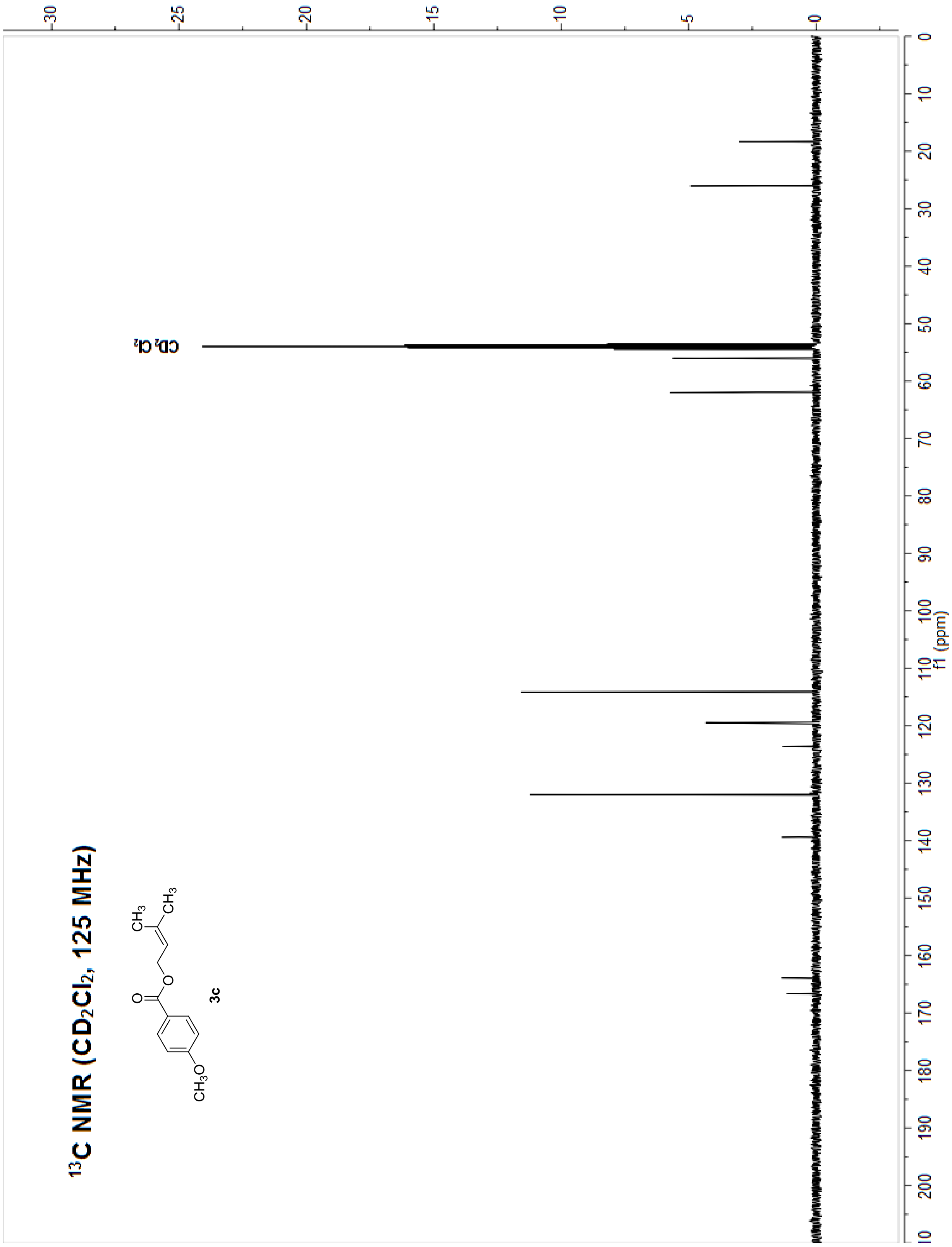

13C NMR (CD2Cl2, 125 MHz)
3c
CD2Cl2
f1 (ppm)

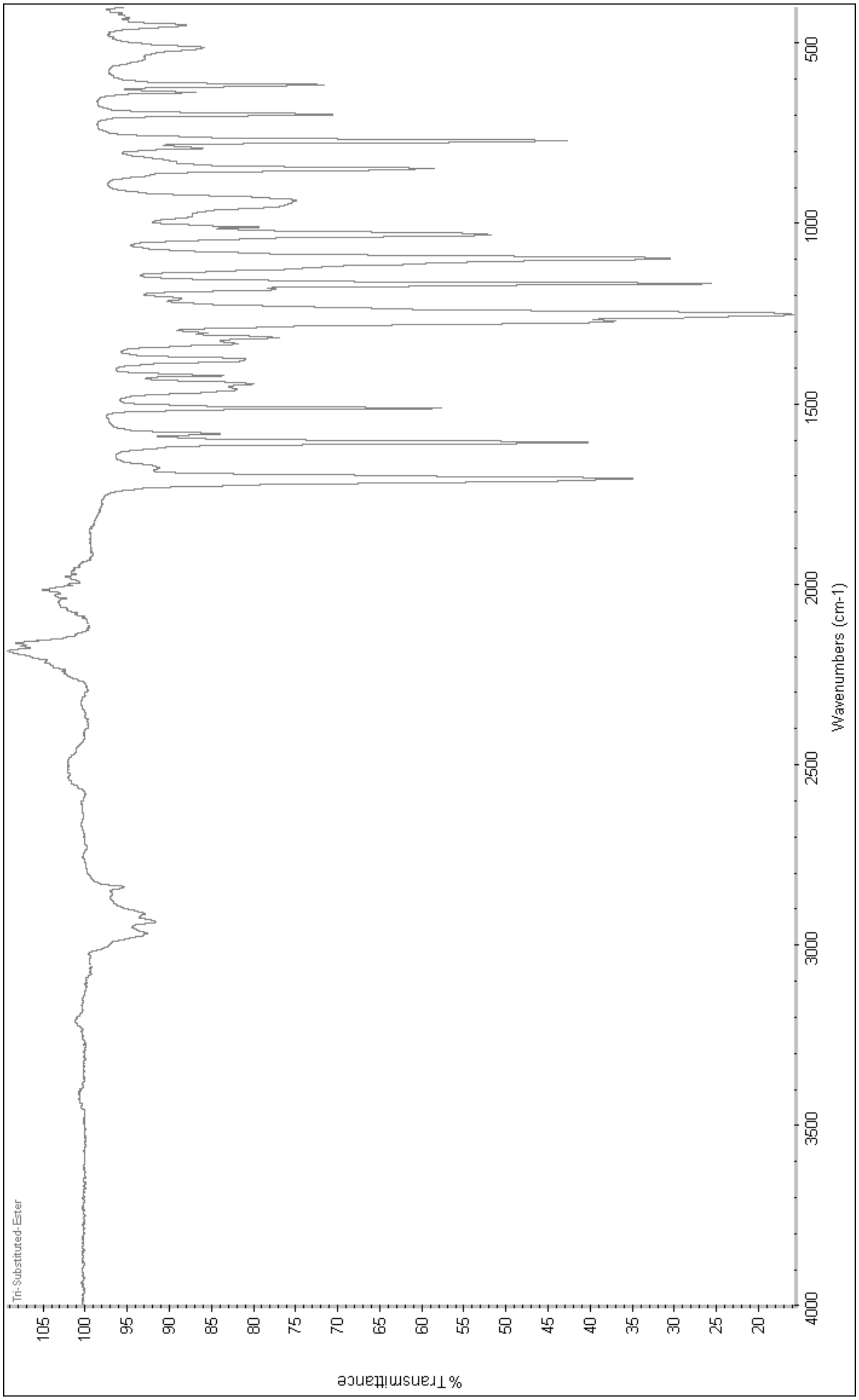
Tri-Substituted-Ester
%Transmittance
Wavenumbers (cm-1)

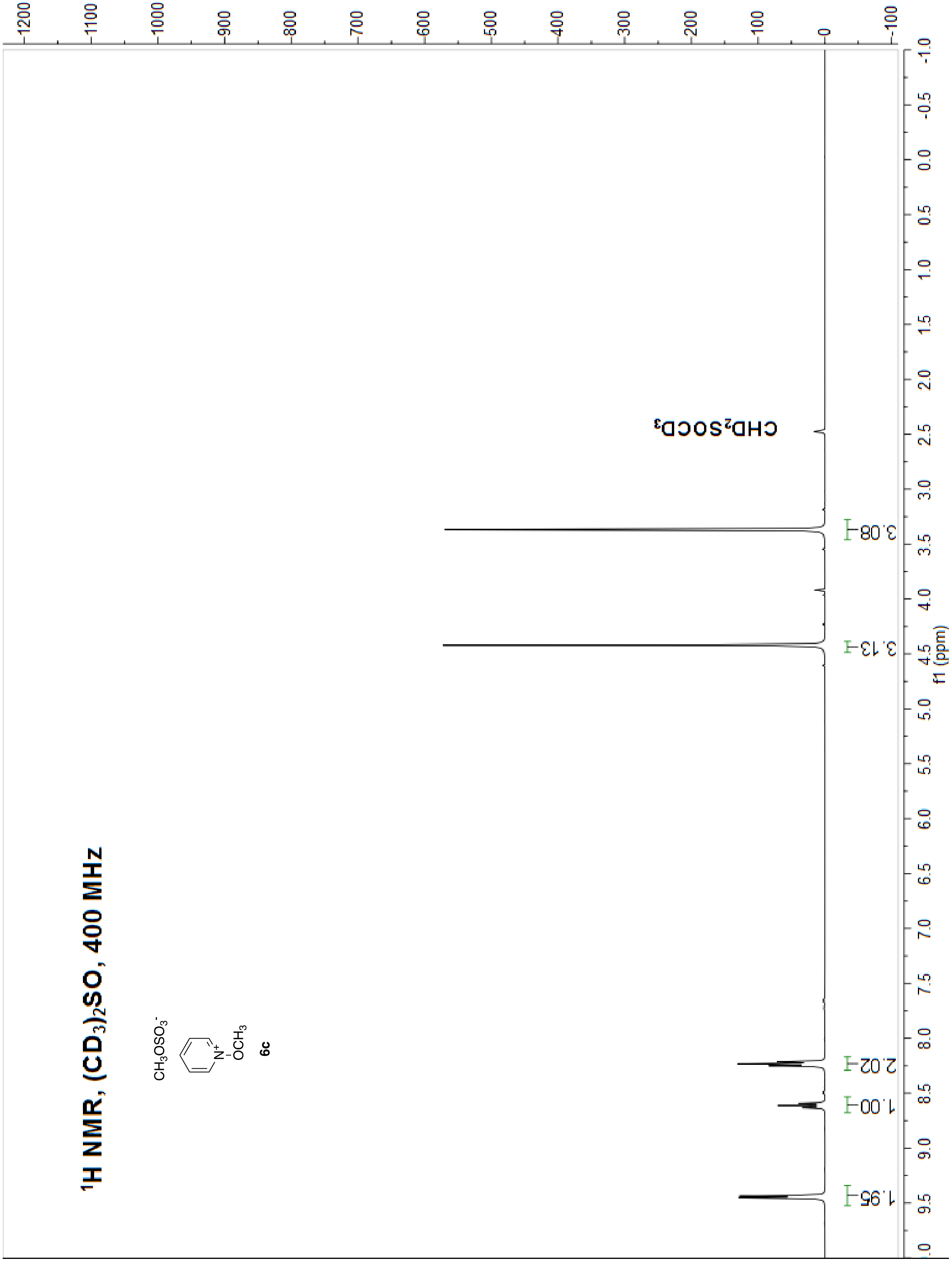

1H NMR, (CD3)2SO, 400 MHz
CH3OSO3-
N+
OCH3
6c
CHD2SOCD3
1.95
1.00
2.02
3.13
3.08
f1 (ppm)

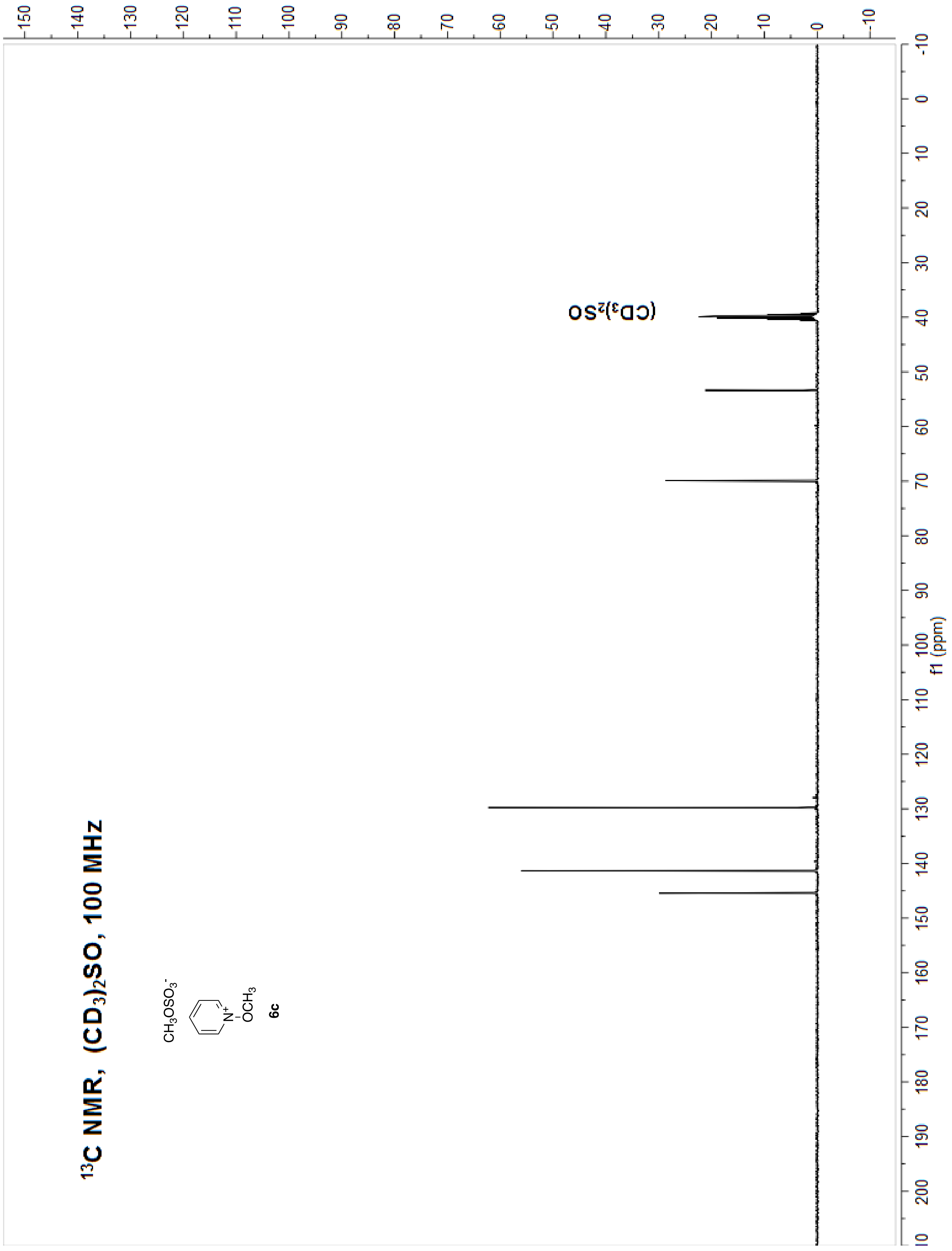
13C NMR, (CD3)2SO, 100 MHz
CH3OSO3-
N+
OCH3
6c
(CD3)2SO
f1 (ppm)

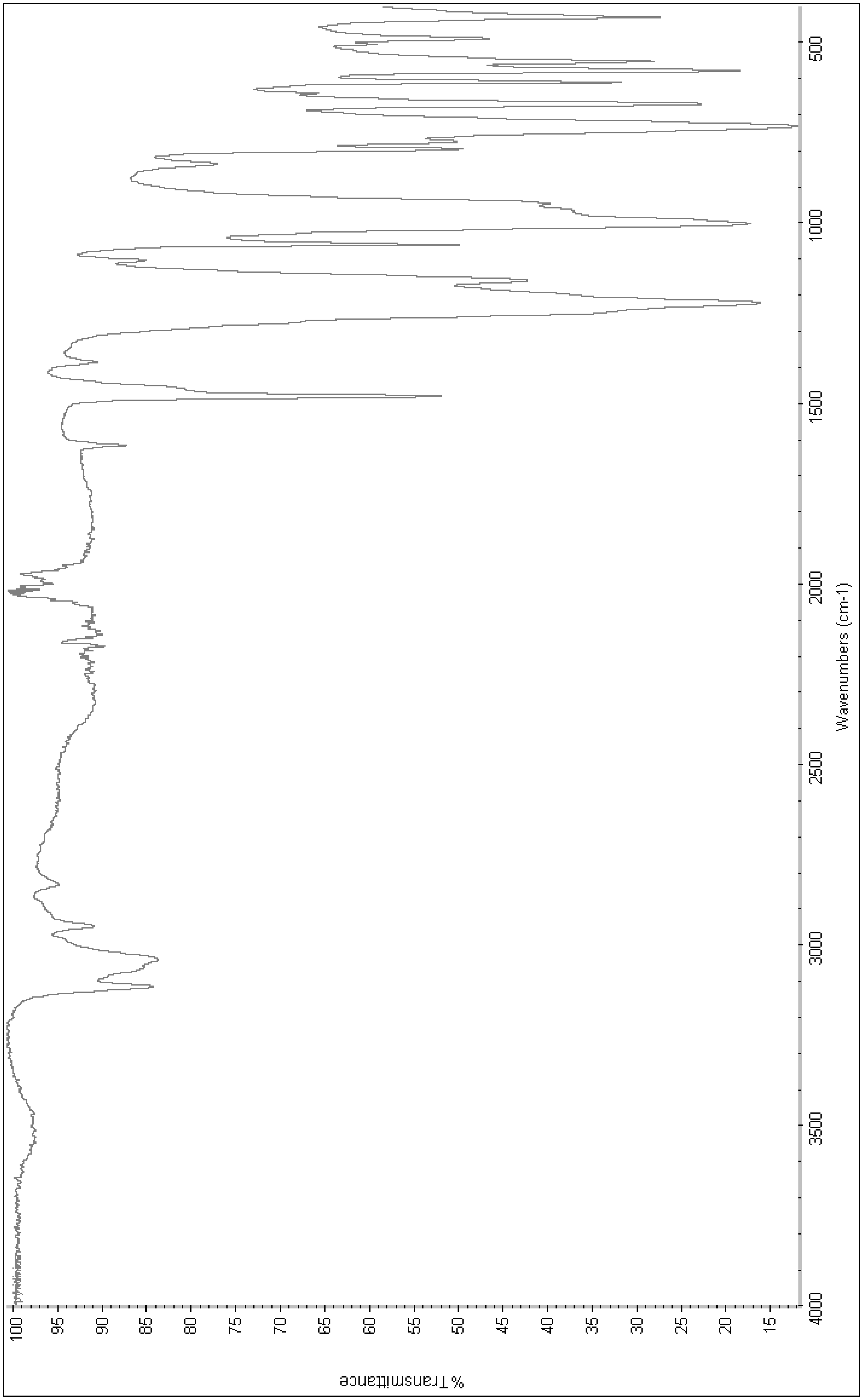
%Transmittance
Wavenumbers (cm-1)

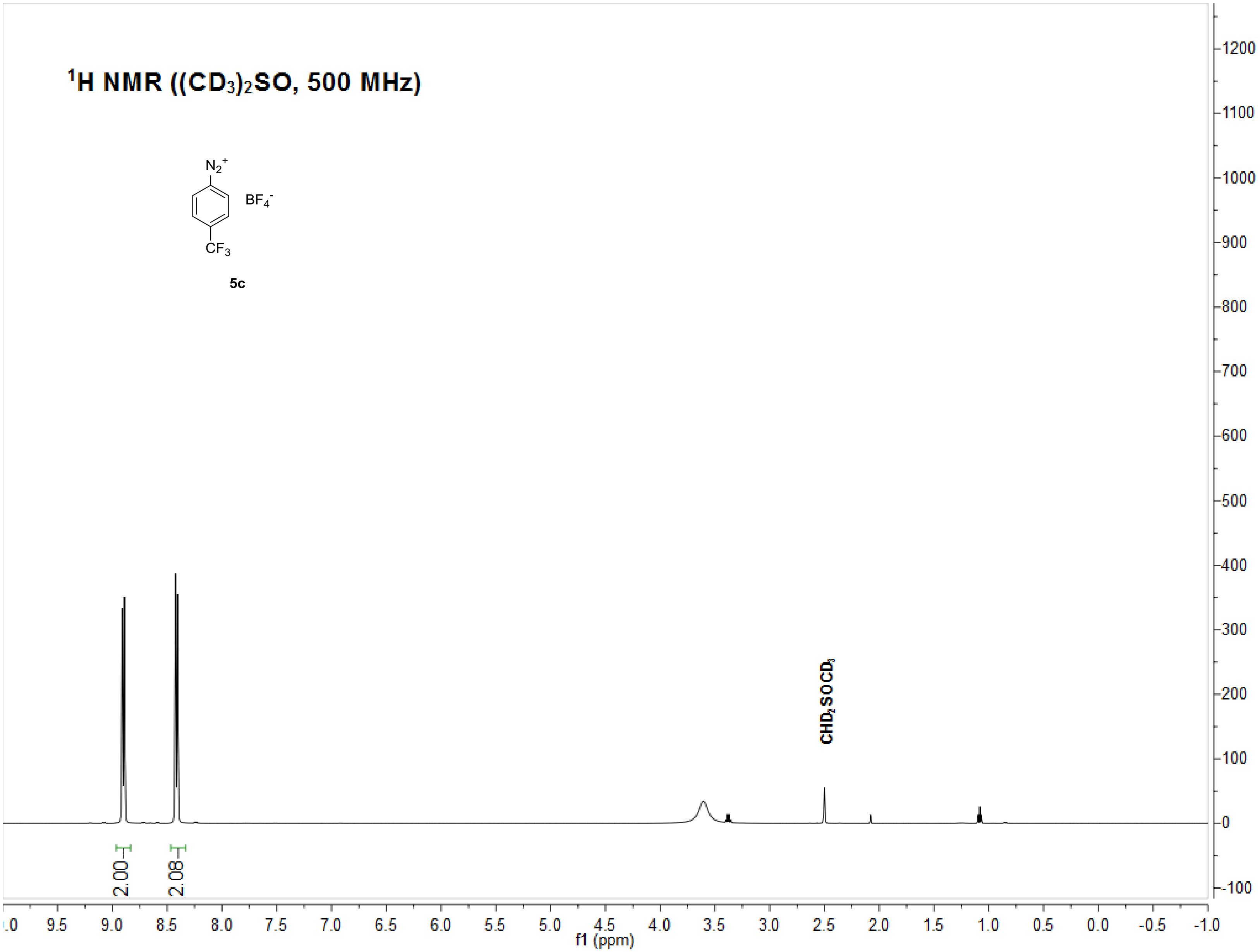

^{1}H NMR ($(CD_3)_2SO$, 500 MHz)
N_2^+
BF_4^-
CF_3
5c
CHD_2SOCD_3
2.00
2.08
f1 (ppm)

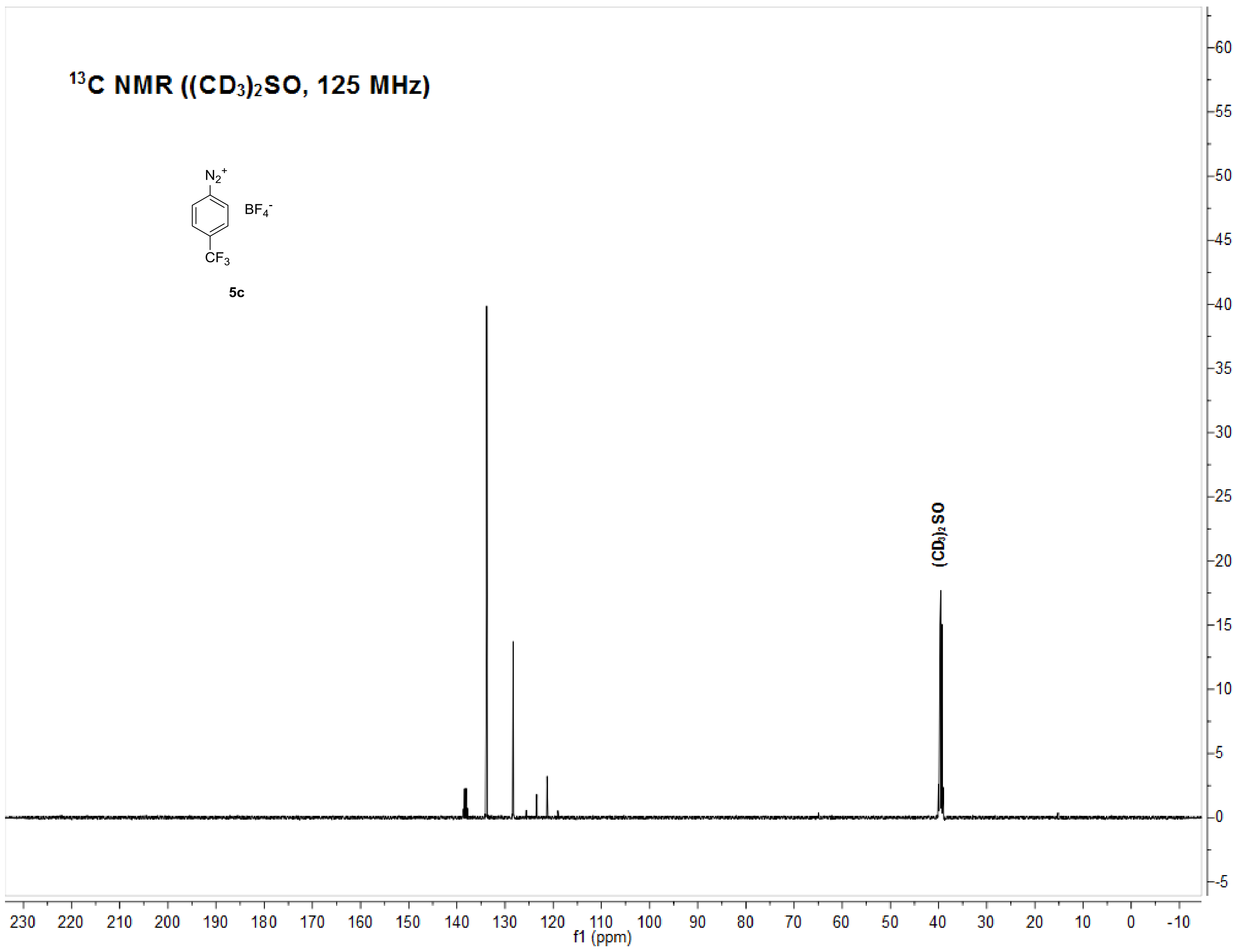
^{13}C NMR ($(CD_3)_2SO$, 125 MHz)
N_2^+
BF_4^-
CF_3
5c
$(CD_3)_2SO$
f1 (ppm)

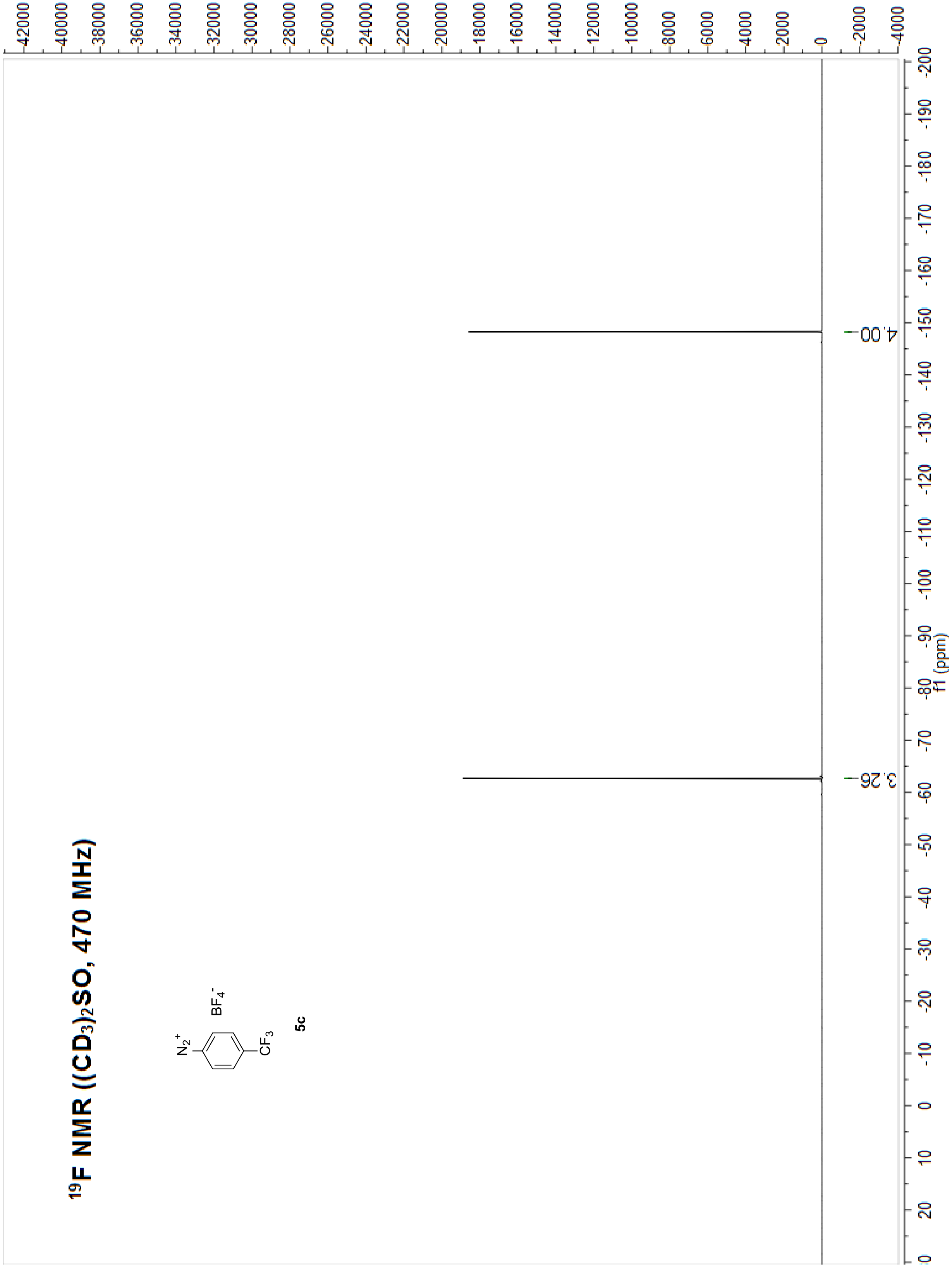

19F NMR ((CD3)2SO, 470 MHz)
N2+
BF4-
CF3
5c
4.00
3.26
f1 (ppm)

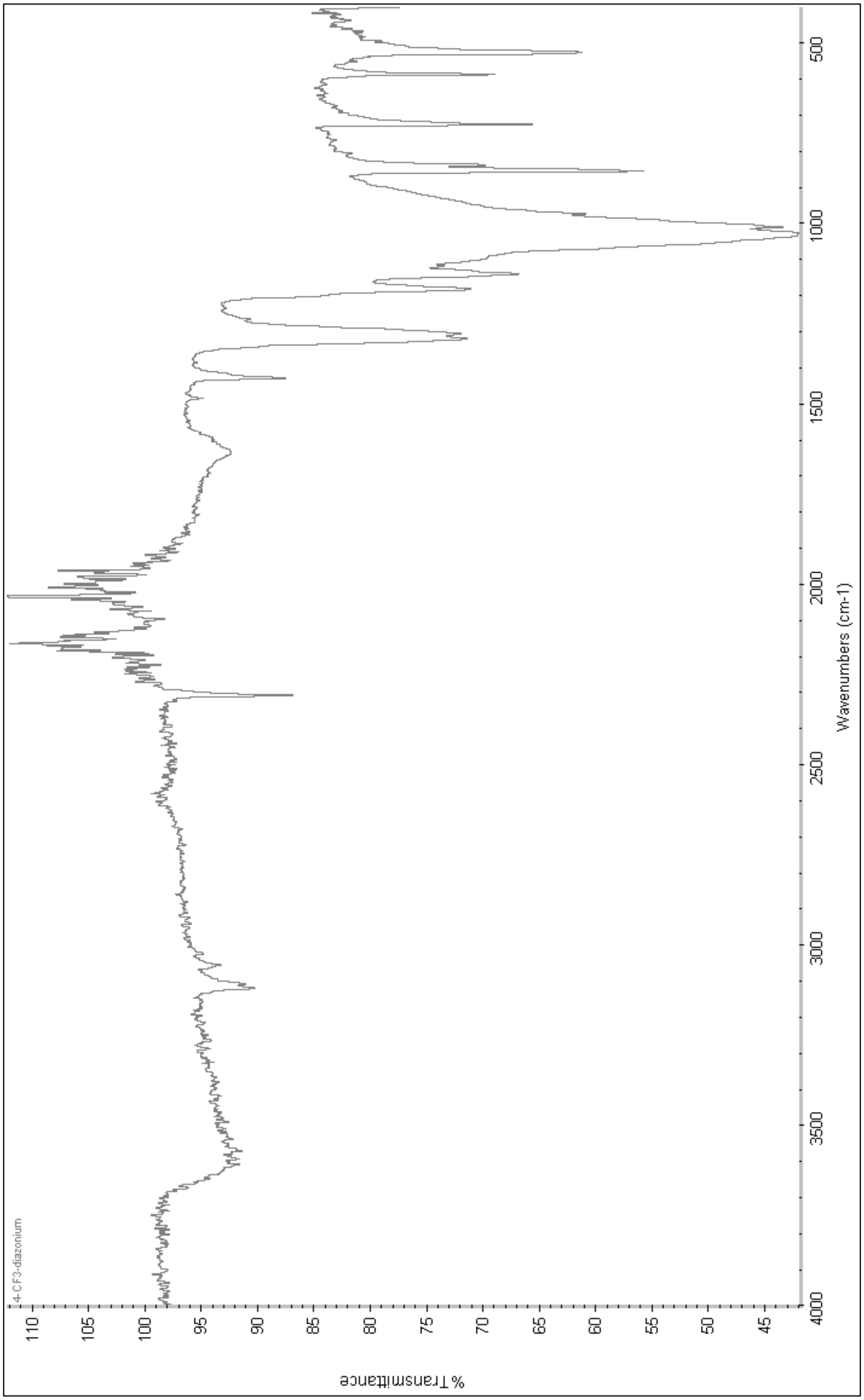
4-CF3-diazonium
%Transmittance
Wavenumbers (cm-1)

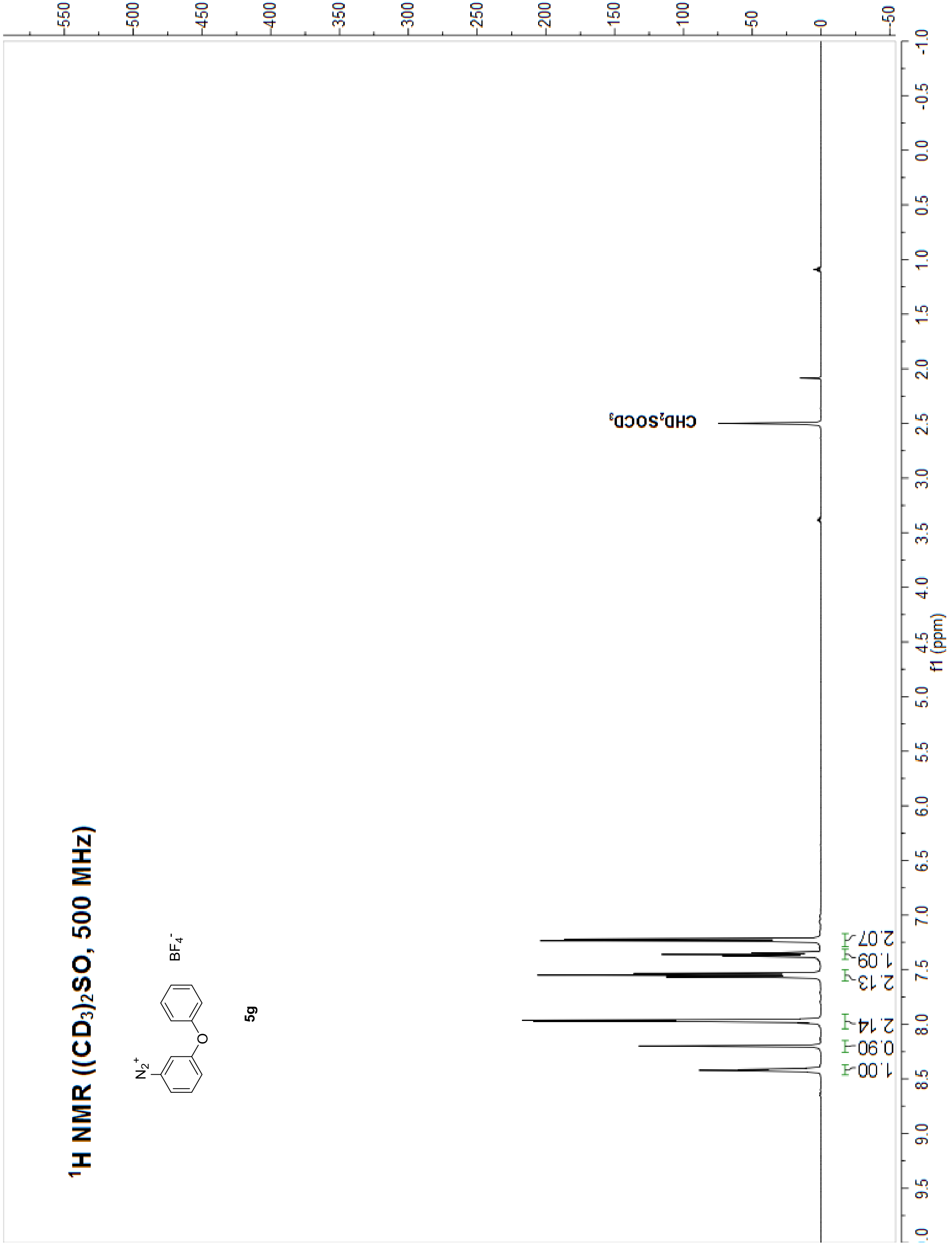

^{1}H NMR ((CD$_3$)$_2$SO, 500 MHz)
N_2^+
BF_4^-
5g
CHD_2SOCD_3
2.07
1.09
2.13
2.14
0.90
1.00
f1 (ppm)

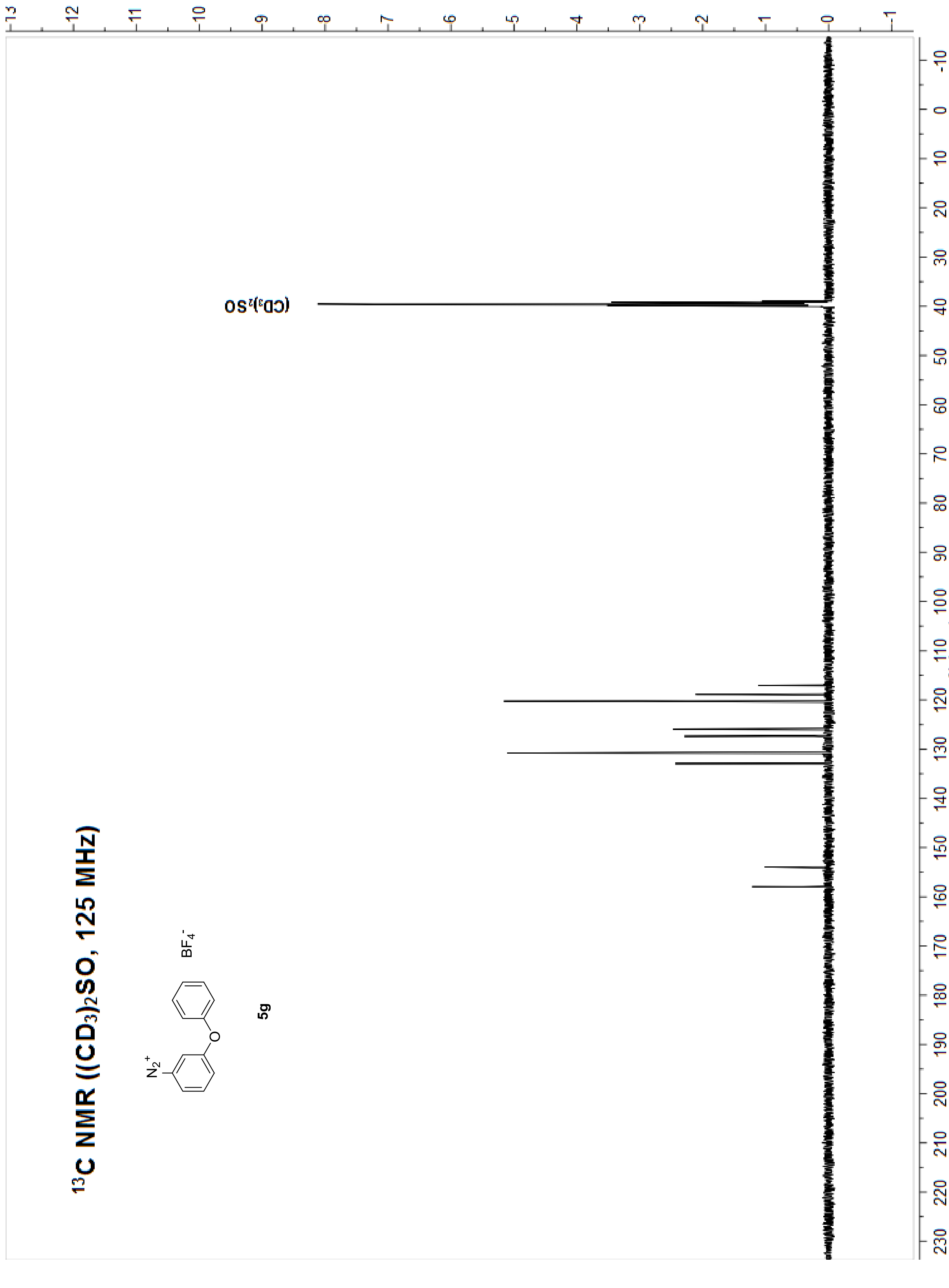
13C NMR ((CD3)2SO, 125 MHz)
N2+
O
BF4-
5g
(CD3)2SO
f1 (ppm)

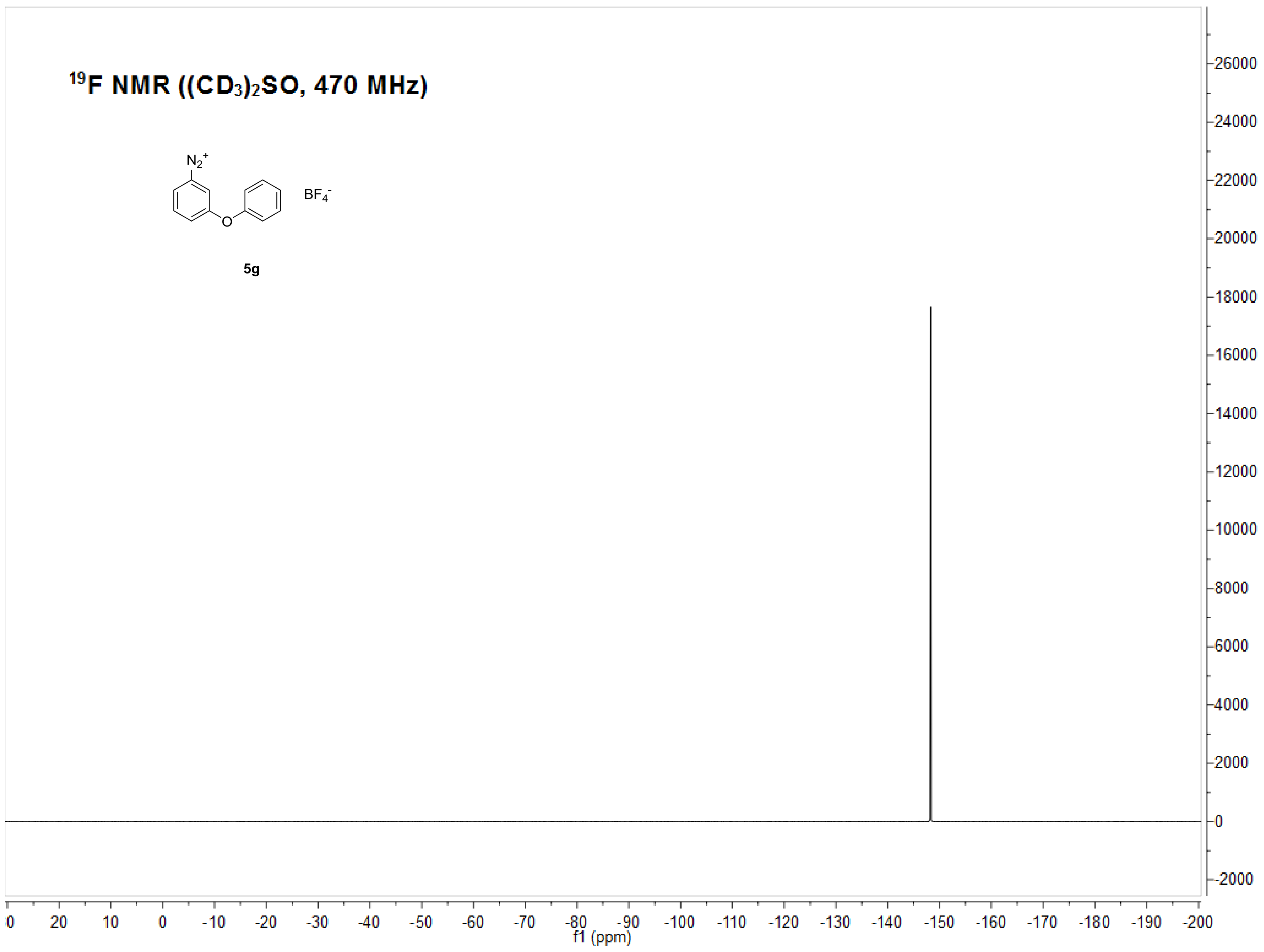

19F NMR ((CD3)2SO, 470 MHz)
N2+
O
BF4-
5g
f1 (ppm)

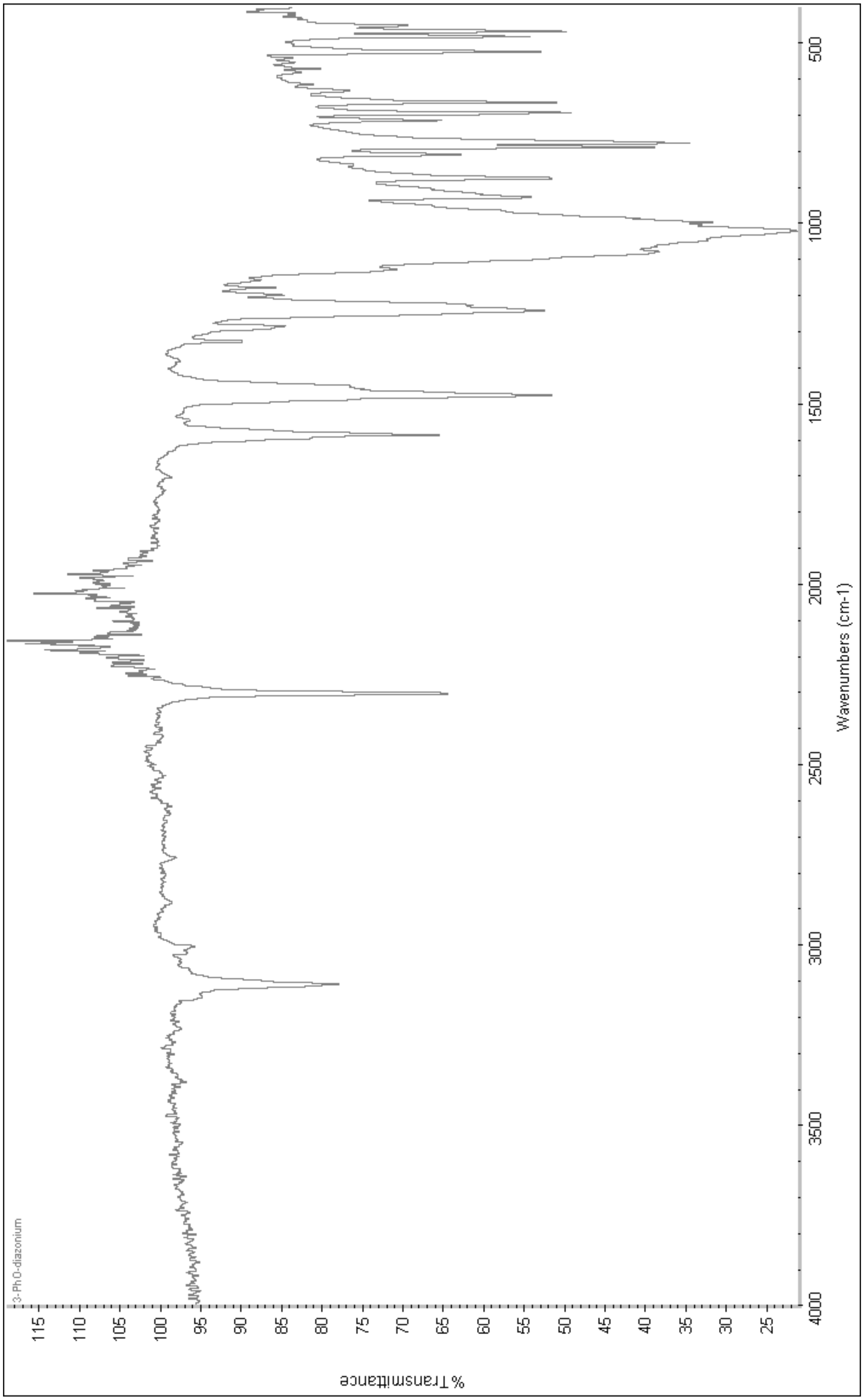
3-PhO-diazonium
%Transmittance
Wavenumbers (cm-1)

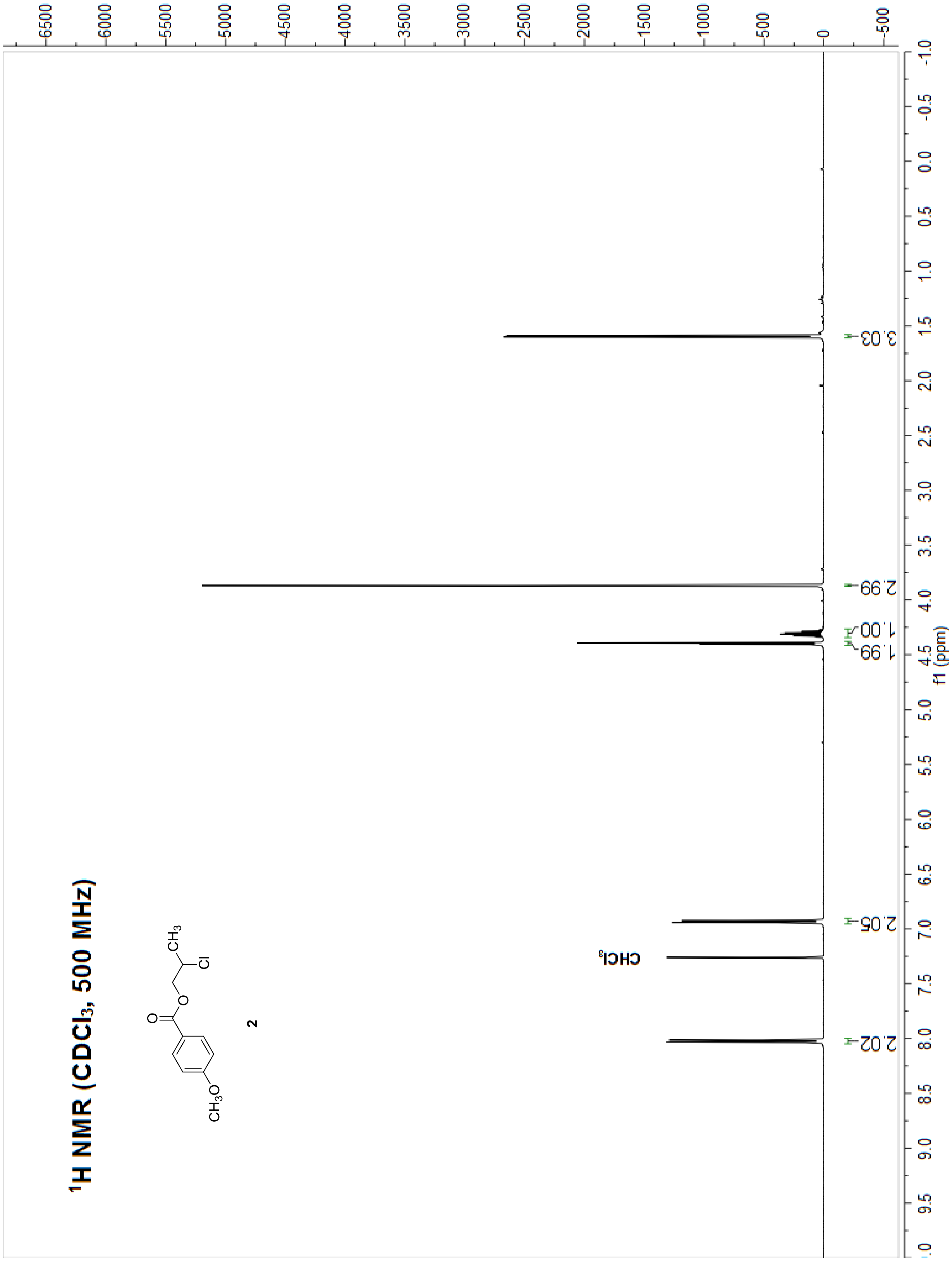

1H NMR (CDCl3, 500 MHz)
2
CHCl3
3.03
2.99
1.00
1.99
2.05
2.02
f1 (ppm)

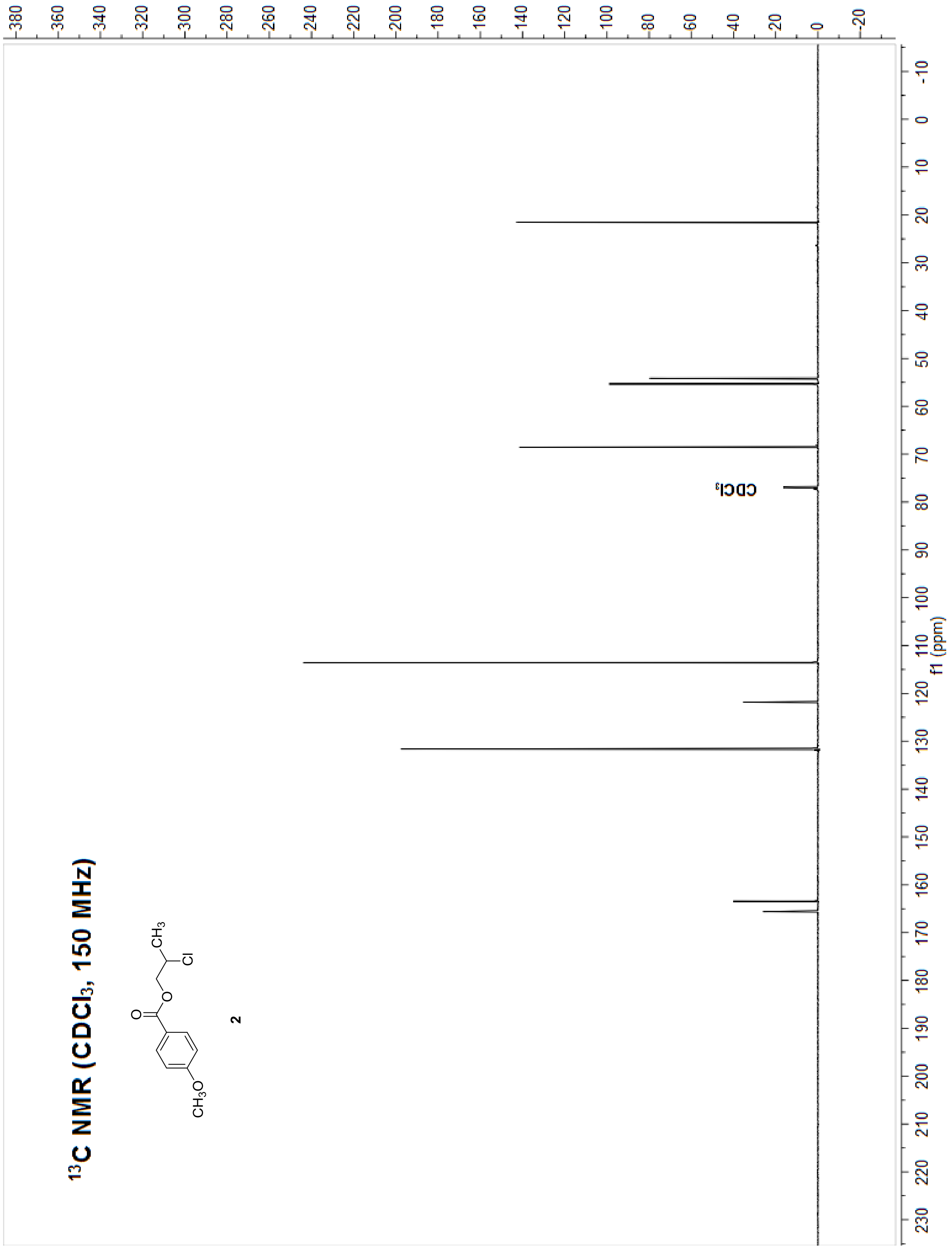

^{13}C NMR (CDCl$_3$, 150 MHz)
CH$_3$O
O
O
Cl
CH$_3$
2
CDCl$_3$
f1 (ppm)

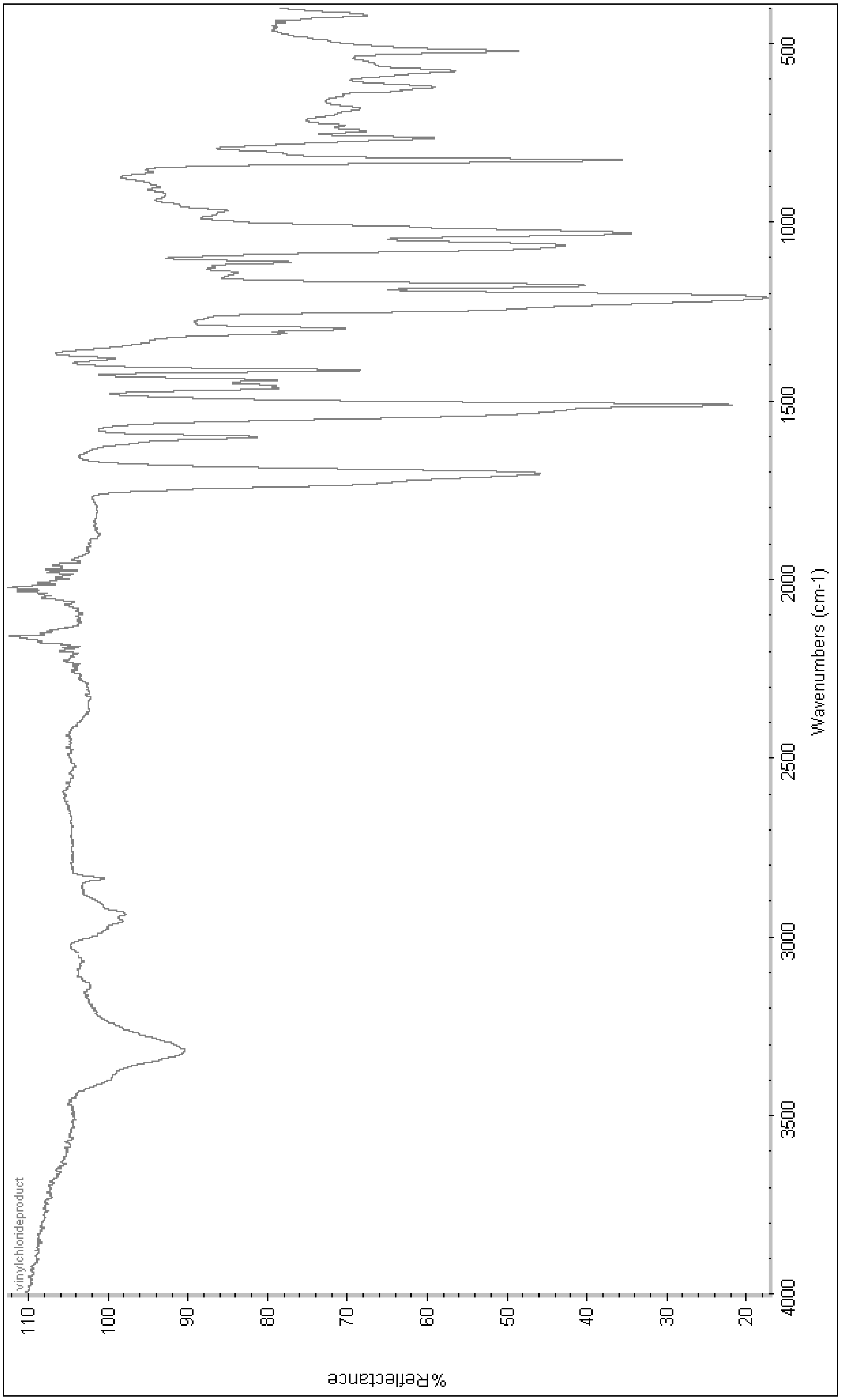

vinylchlorideproduct
%Reflectance
Wavenumbers (cm-1)

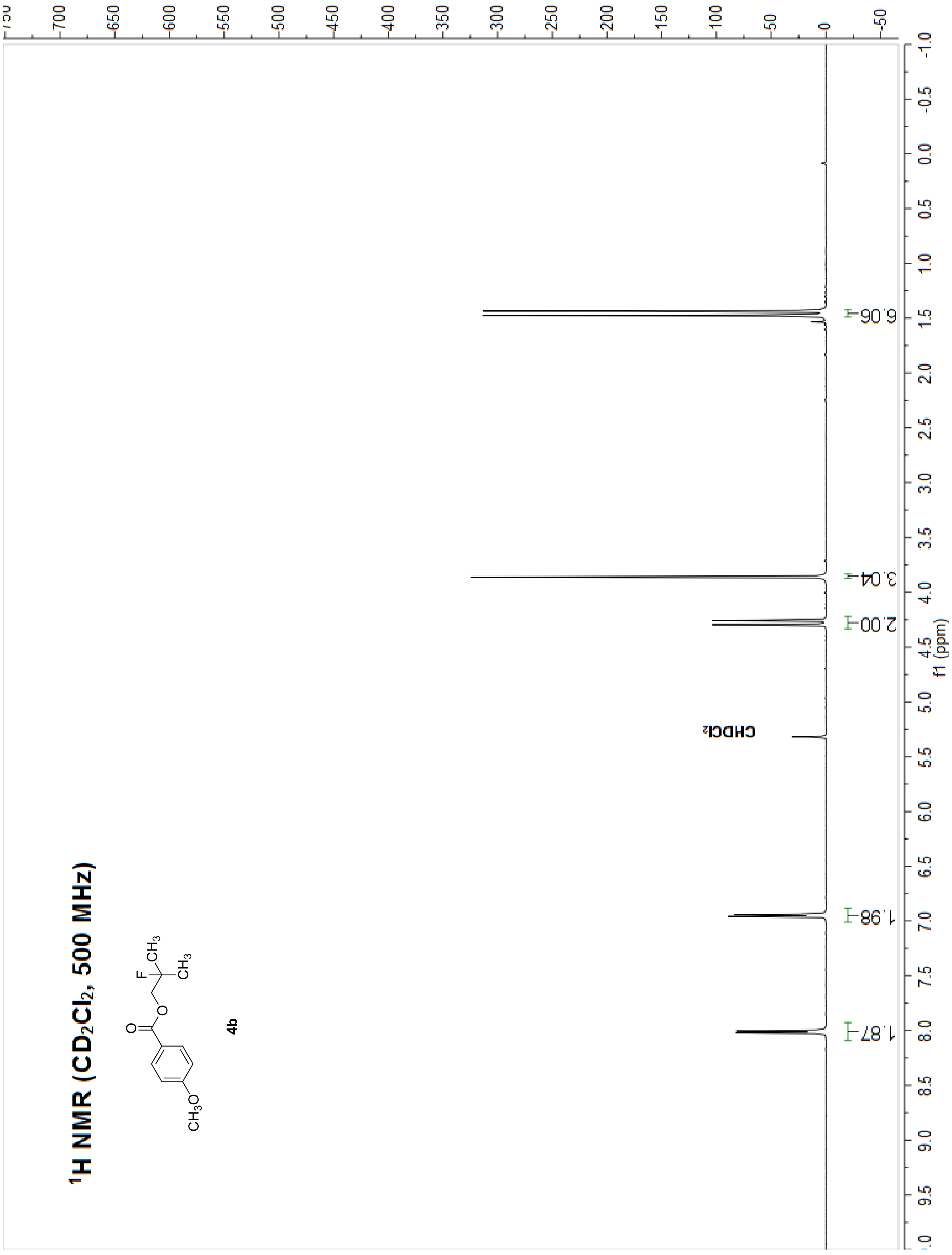

1H NMR (CD2Cl2, 500 MHz)
4b
CHDCl2
6.06
3.04
2.00
1.98
1.87
f1 (ppm)

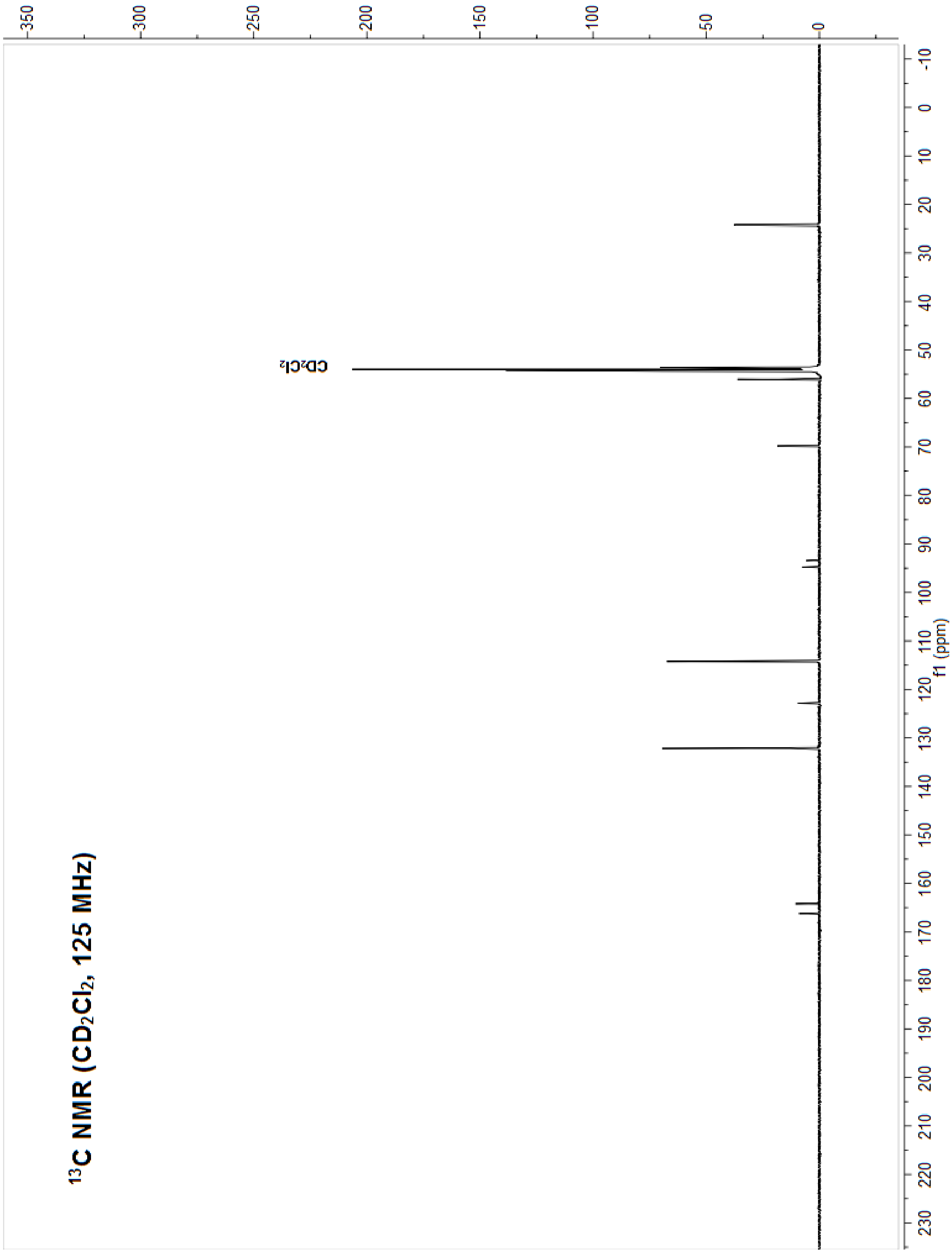

^{13}C NMR (CD_2Cl_2, 125 MHz)
CD_2Cl_2
f1 (ppm)

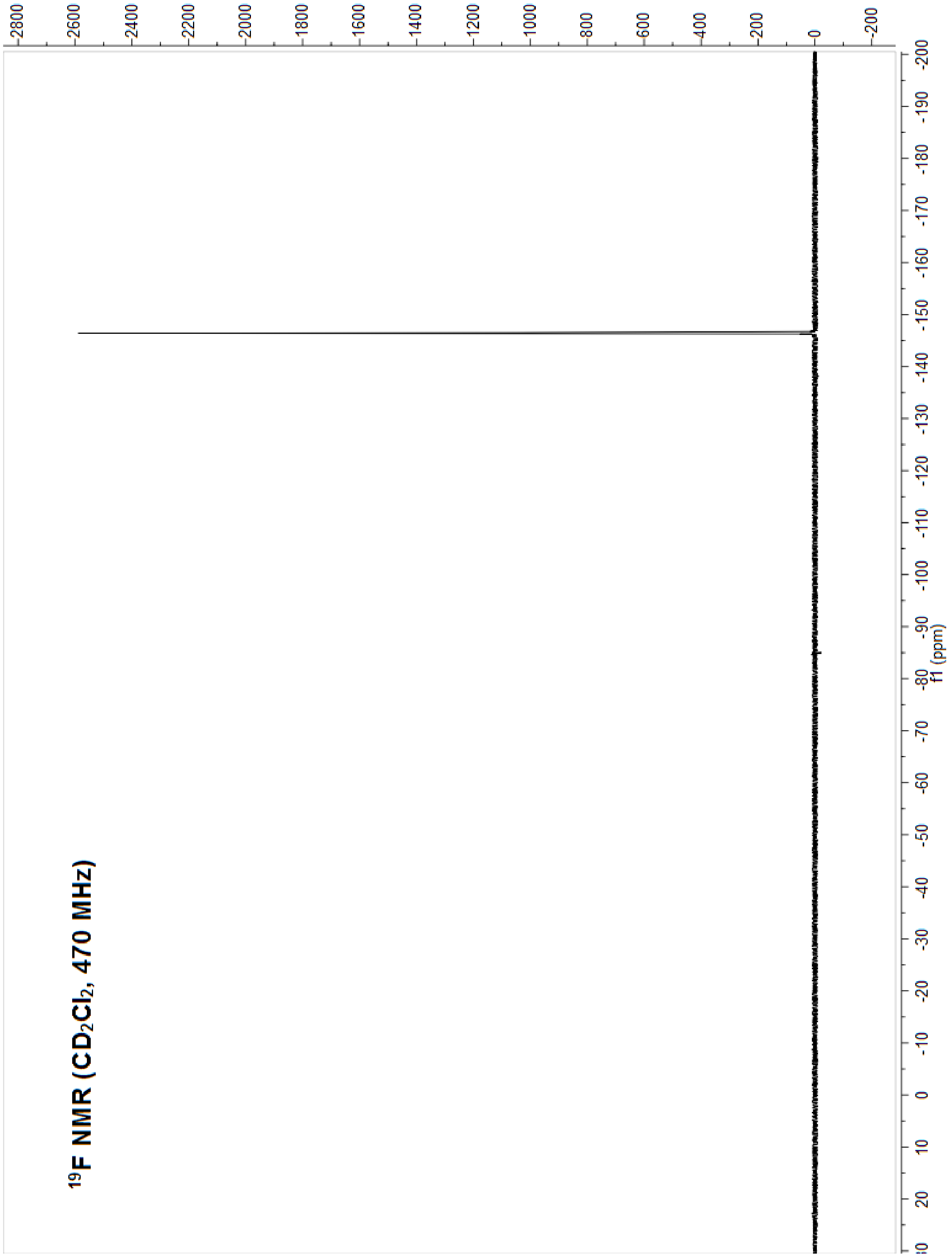

19F NMR (CD2Cl2, 470 MHz)
f1 (ppm)

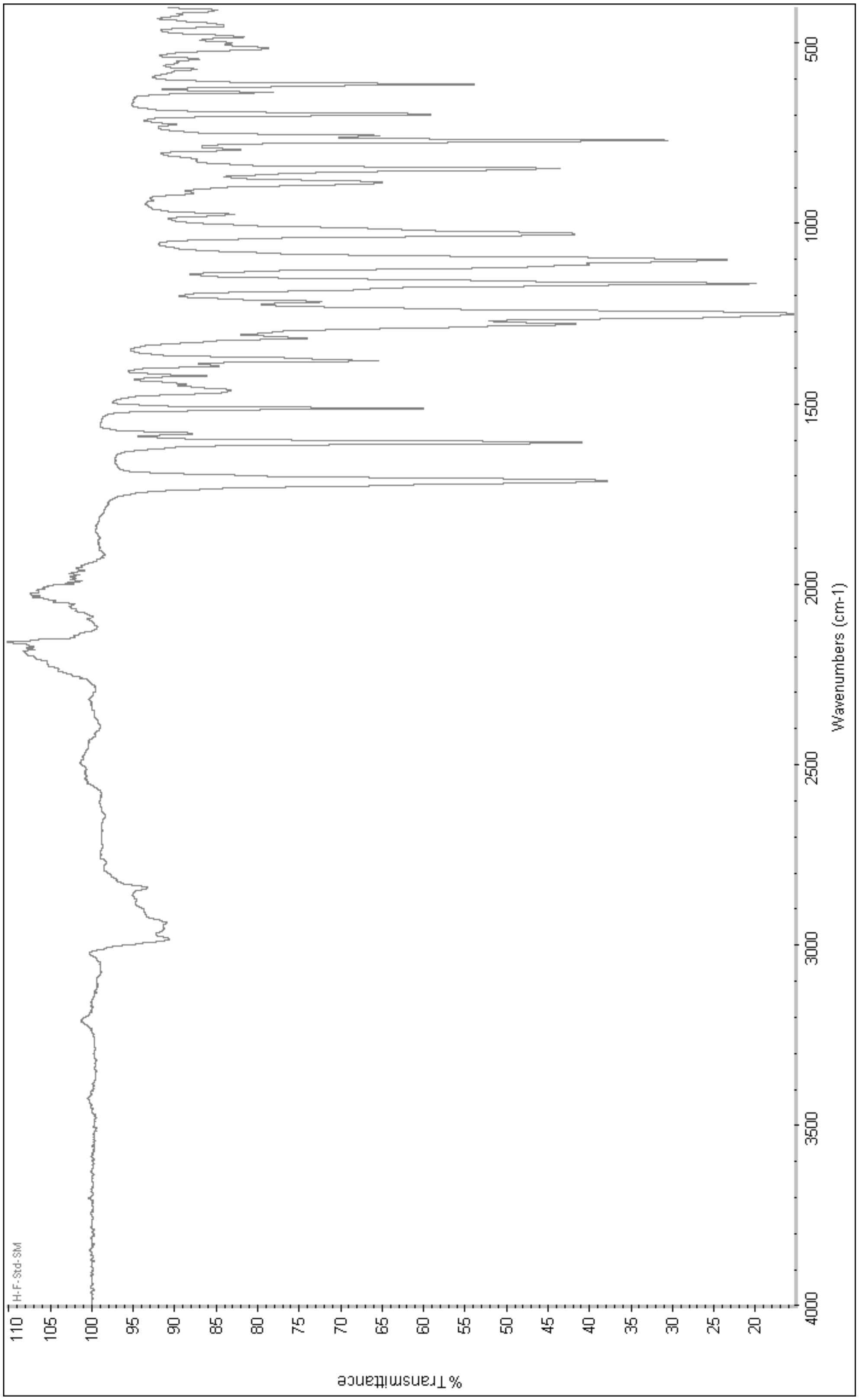
H-F-Std-SM
%Transmittance
Wavenumbers (cm-1)

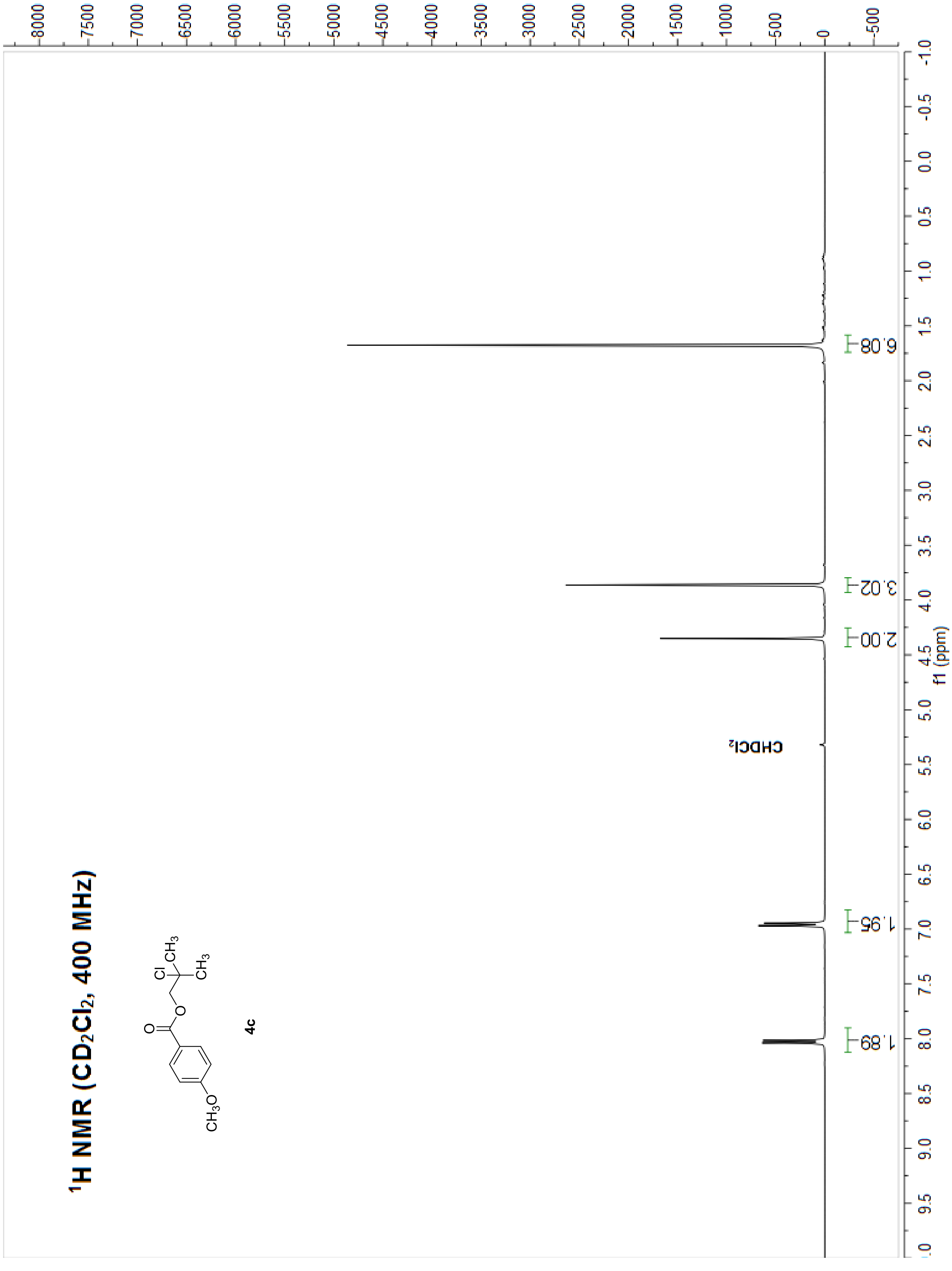

1H NMR (CD2Cl2, 400 MHz)
4c
CHDCl2
6.08
3.02
2.00
1.95
1.89
f1 (ppm)

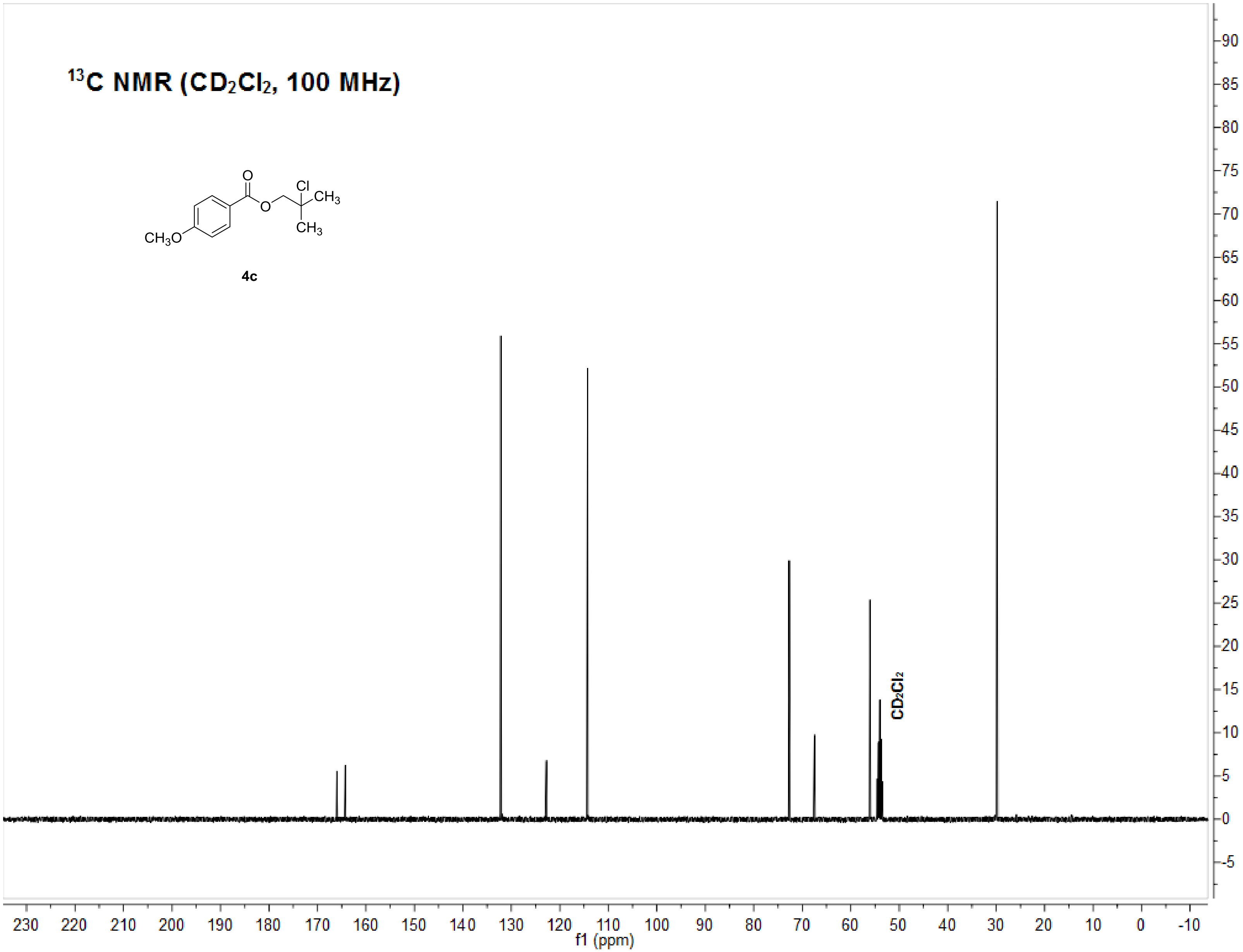

13C NMR (CD2Cl2, 100 MHz)
O
Cl
CH3
CH3
CH3O
O
4c
CD2Cl2
f1 (ppm)

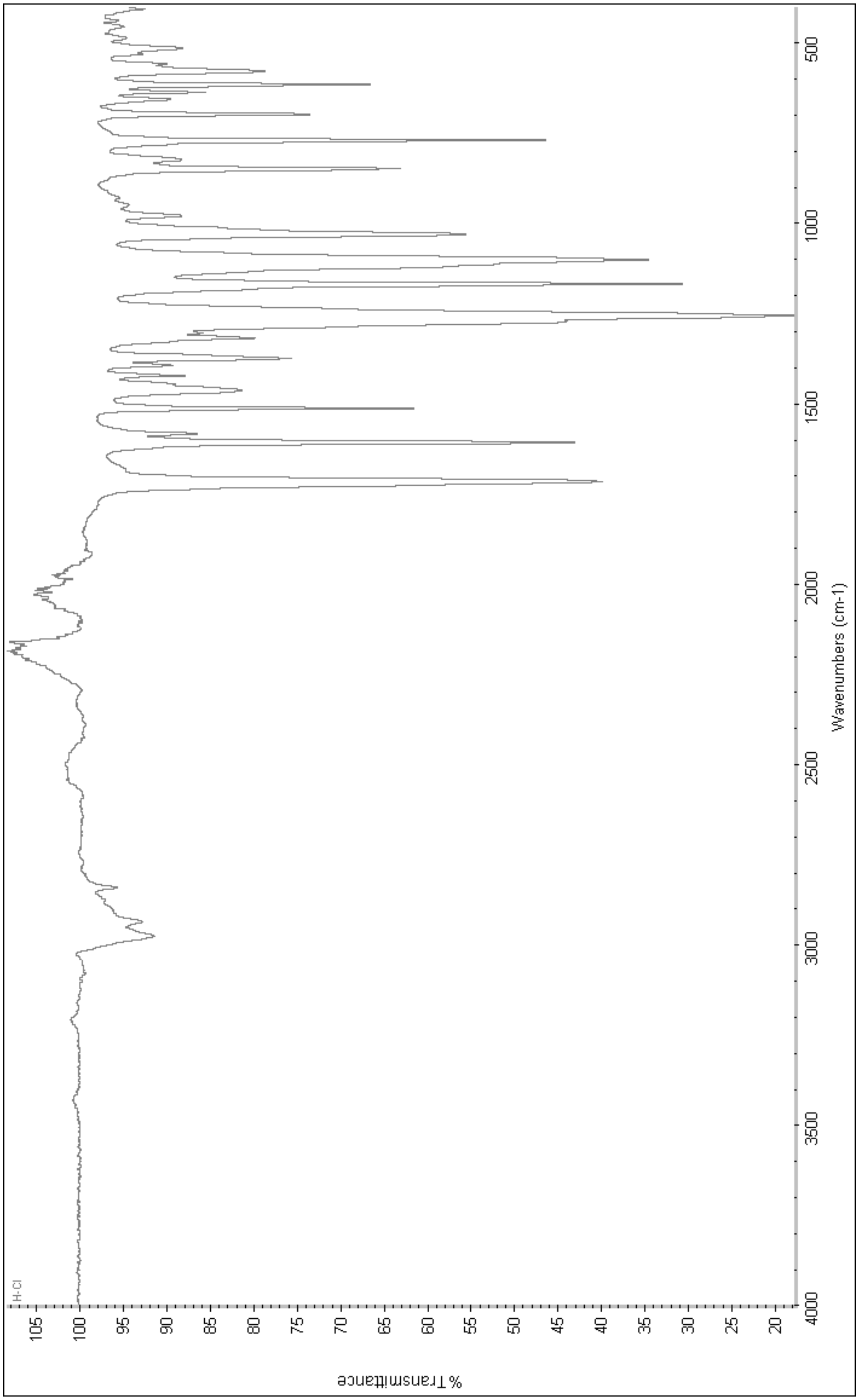
H-Cl
%Transmittance
Wavenumbers (cm-1)

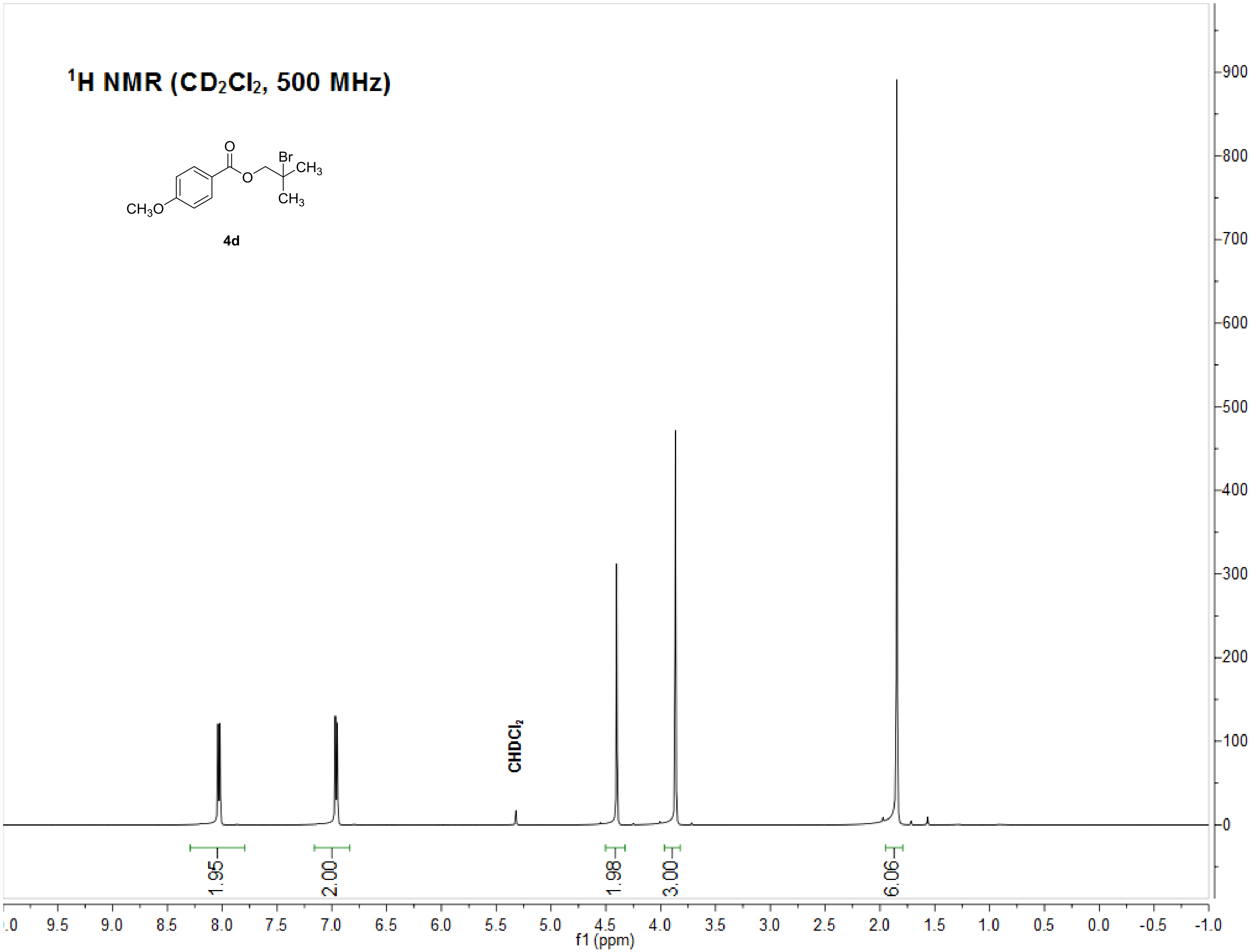

1H NMR (CD2Cl2, 500 MHz)
Br
CH3
CH3
CH3O
O
O
4d
CHDCl2
1.95
2.00
1.98
3.00
6.06
f1 (ppm)

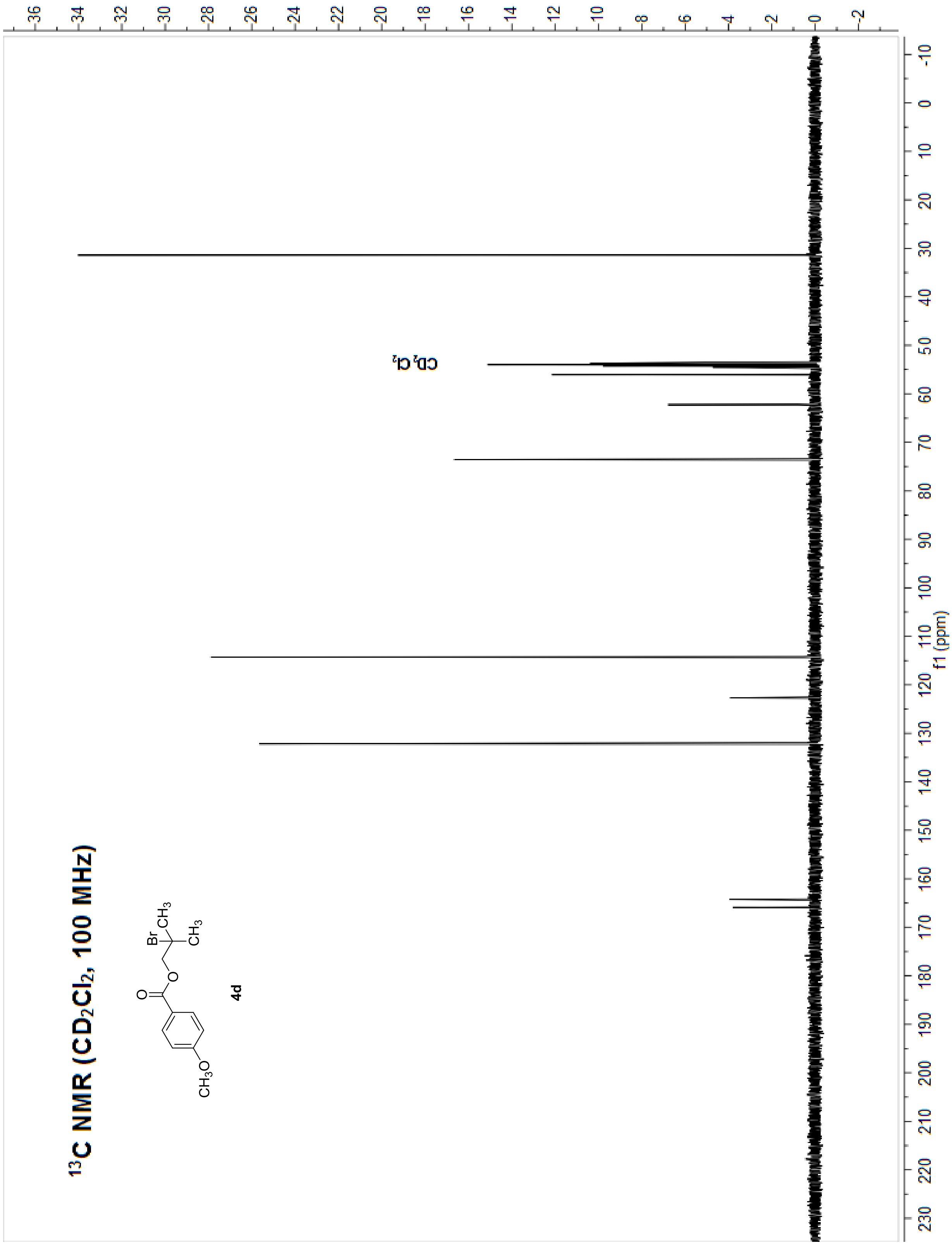
13C NMR (CD2Cl2, 100 MHz)
4d
CD2Cl2
f1 (ppm)

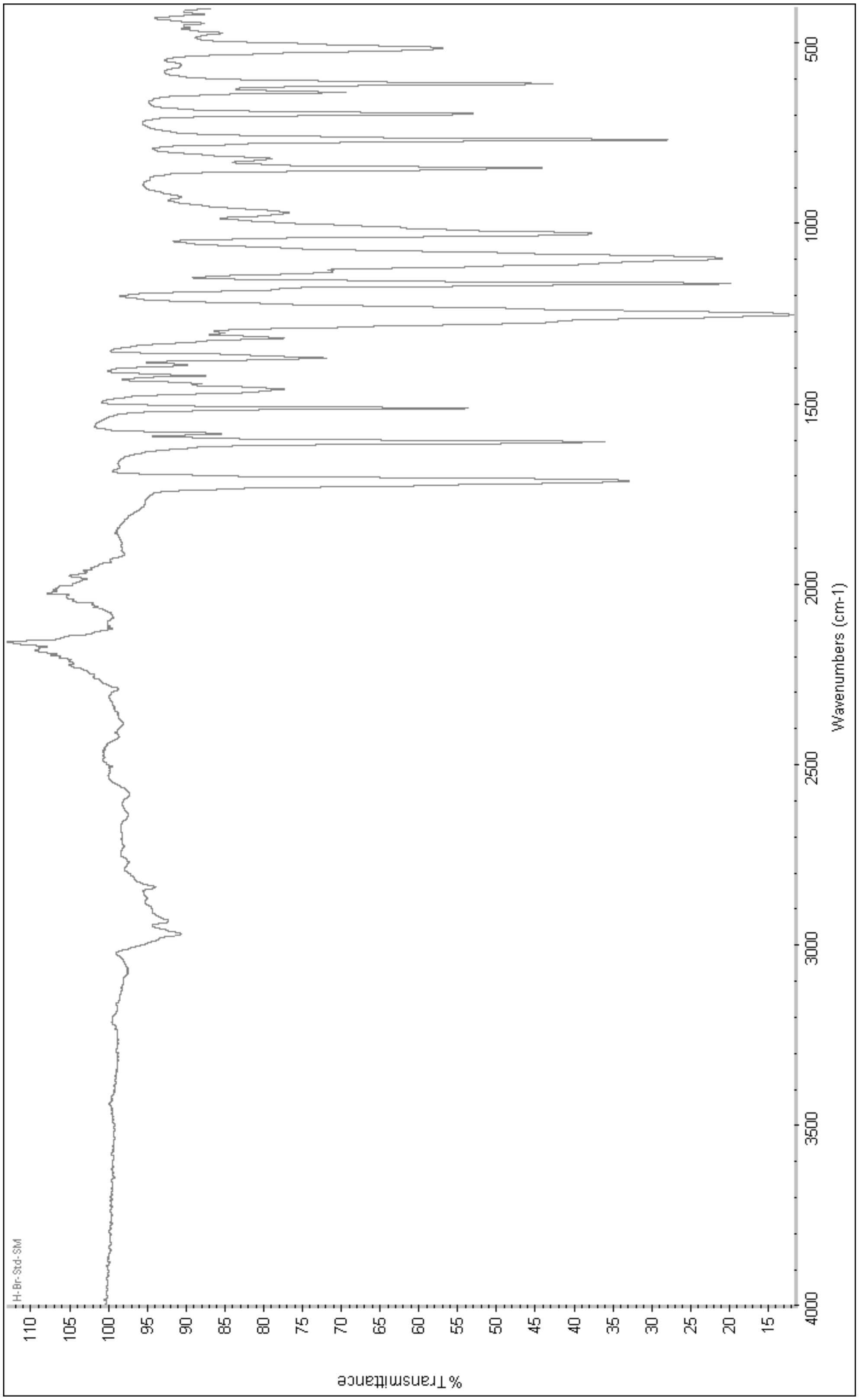
H-Br-Std-SM
%Transmittance
Wavenumbers (cm-1)

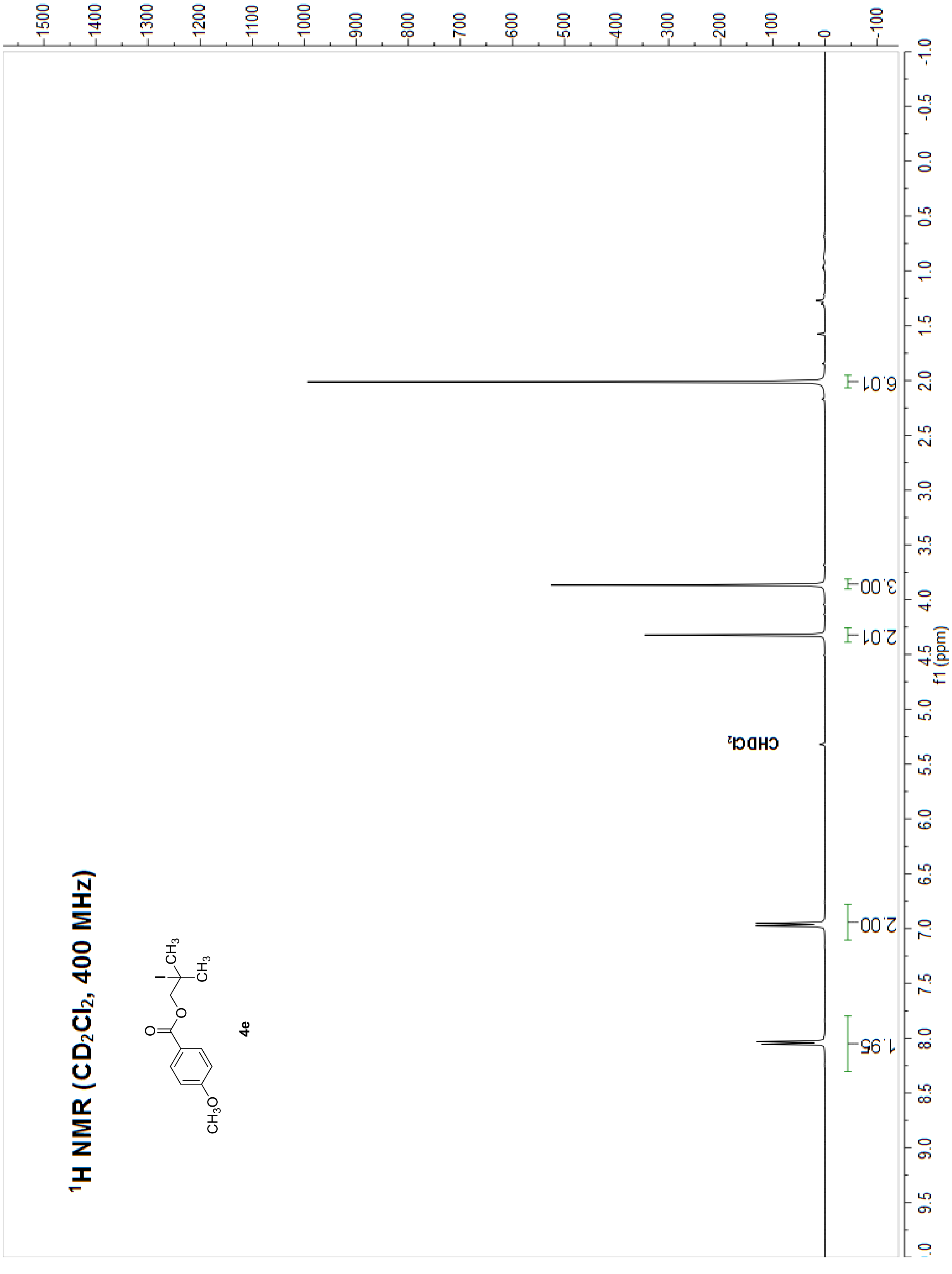

1H NMR (CD2Cl2, 400 MHz)
4e
CHDCl2
6.01
3.00
2.01
2.00
1.95
f1 (ppm)

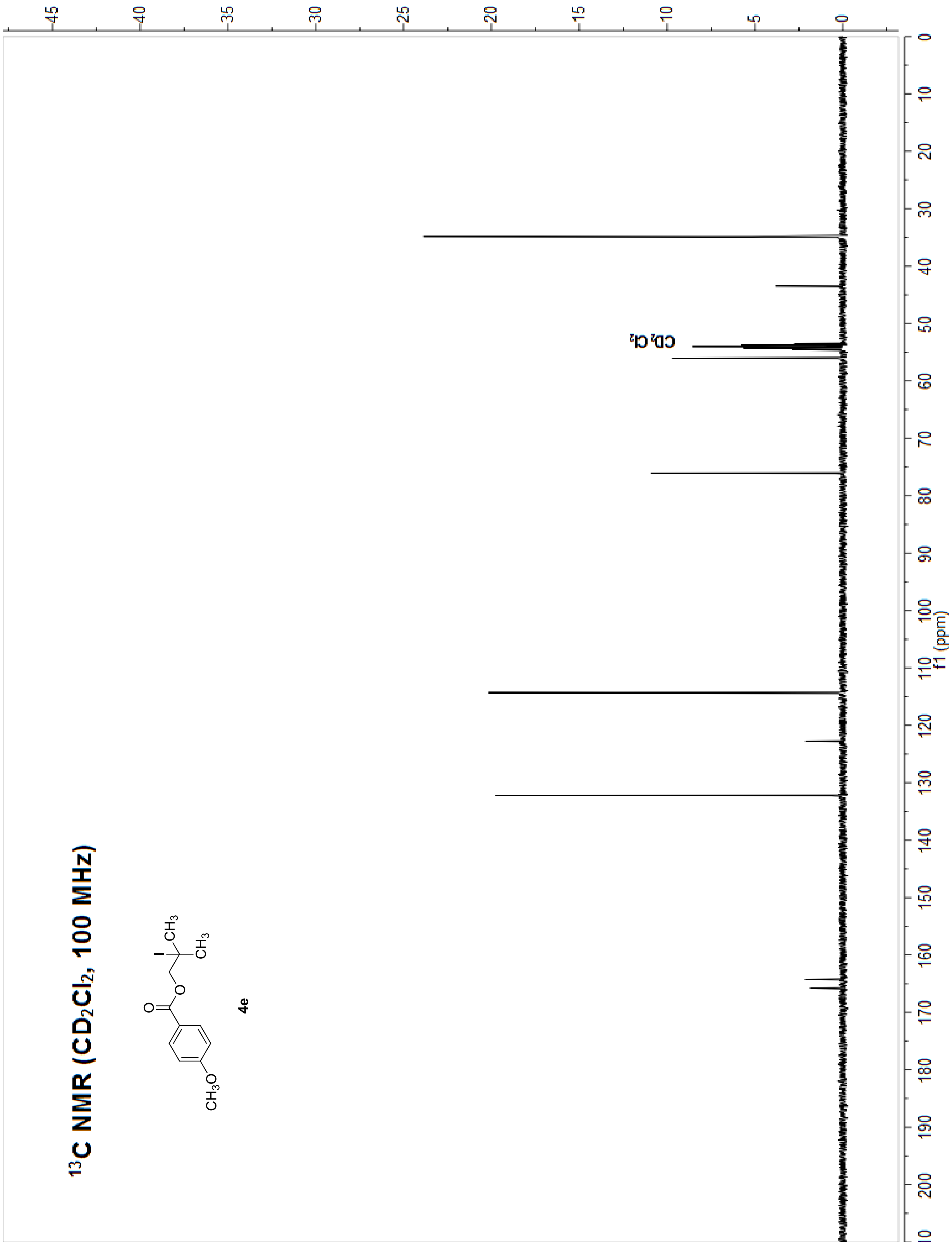

13C NMR (CD2Cl2, 100 MHz)
4e
CD2Cl2
f1 (ppm)

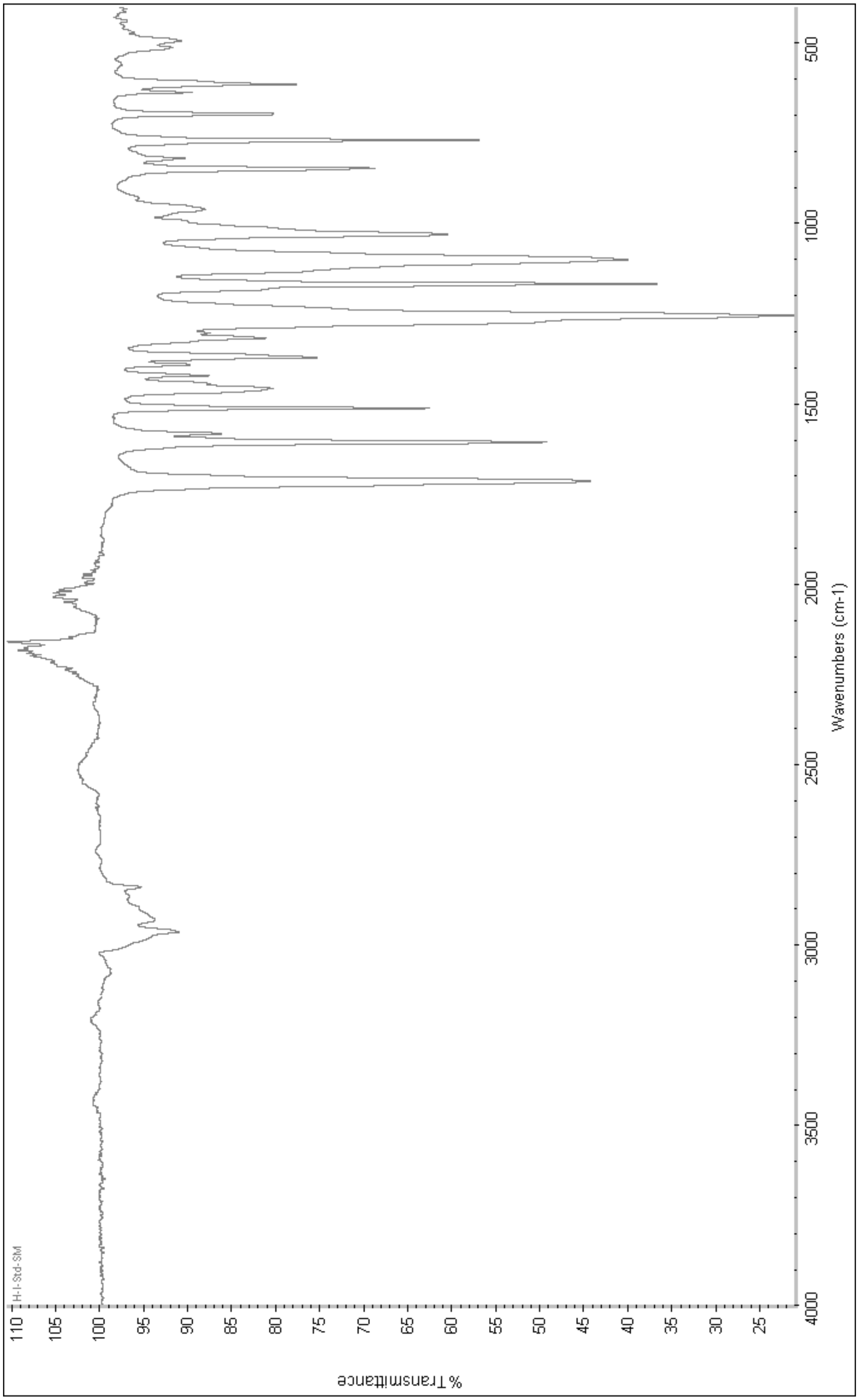

H-I-Std-SM
%Transmittance
Wavenumbers (cm-1)
110
105
100
95
90
85
80
75
70
65
60
55
50
45
40
35
30
25
4000
3500
3000
2500
2000
1500
1000
500

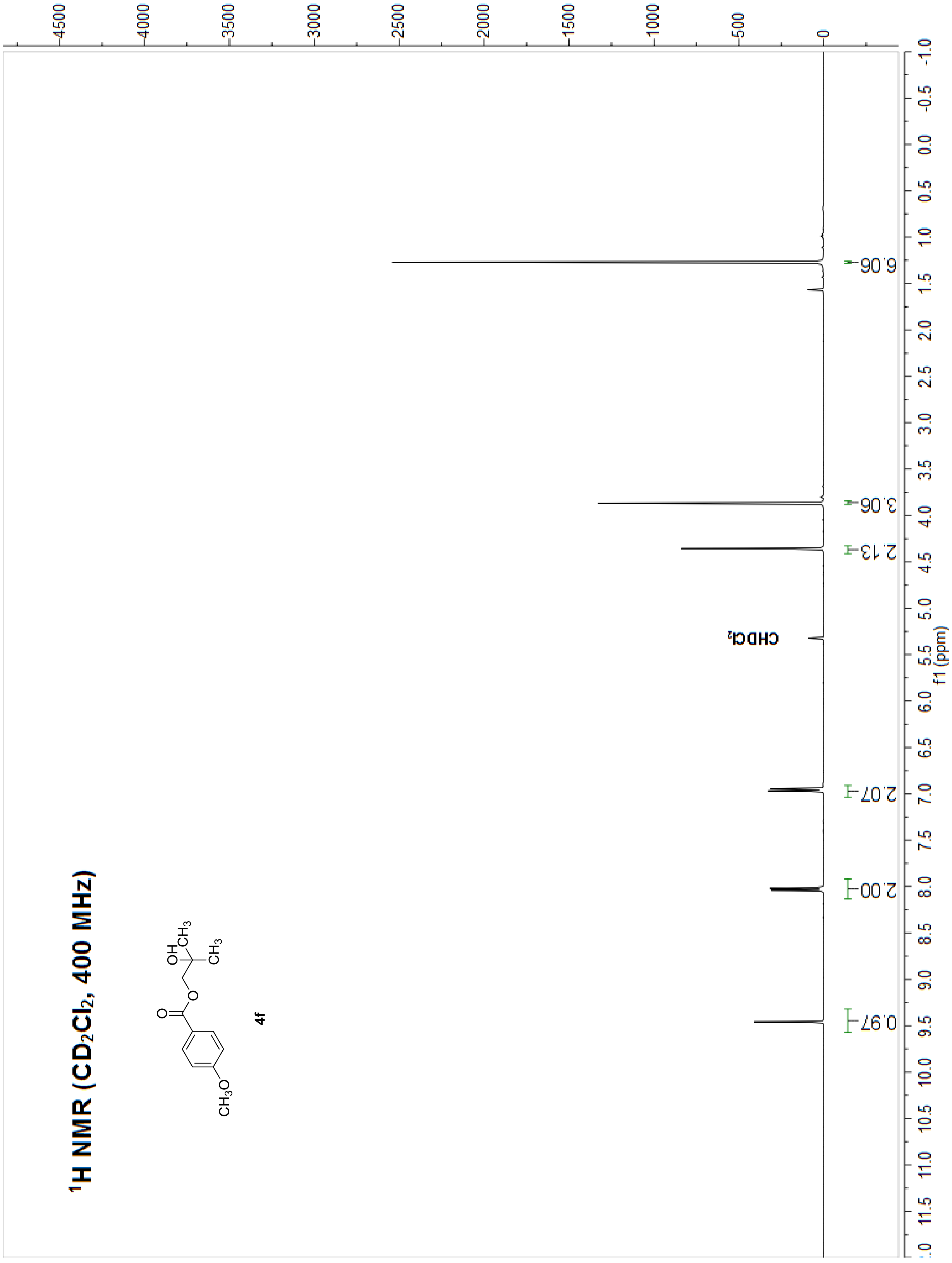

1H NMR (CD2Cl2, 400 MHz)
4f
CHDCl2
f1 (ppm)
0.97
2.00
2.07
2.13
3.06
6.06

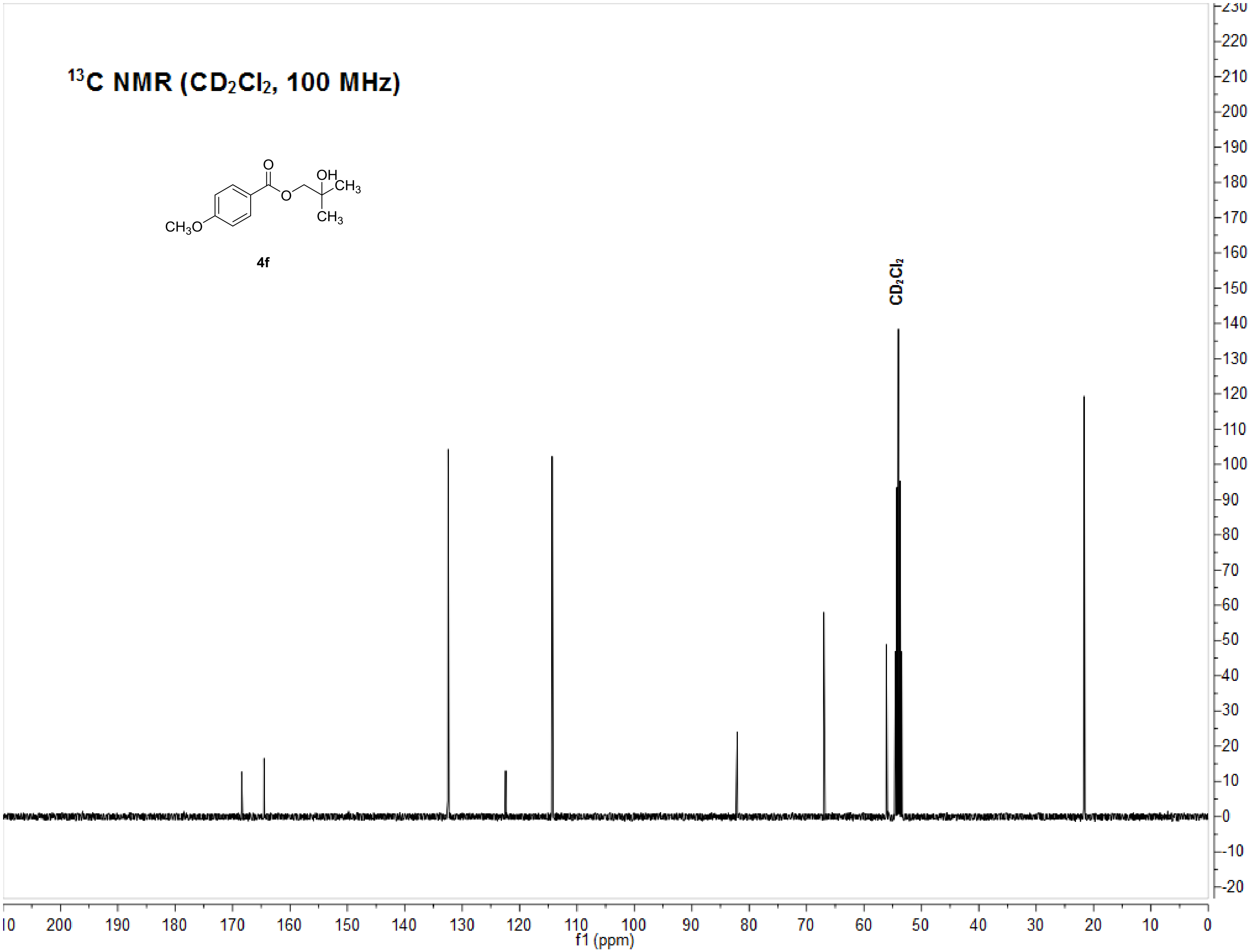

^{13}C NMR (CD_2Cl_2, 100 MHz)
4f
CD2Cl2
f1 (ppm)

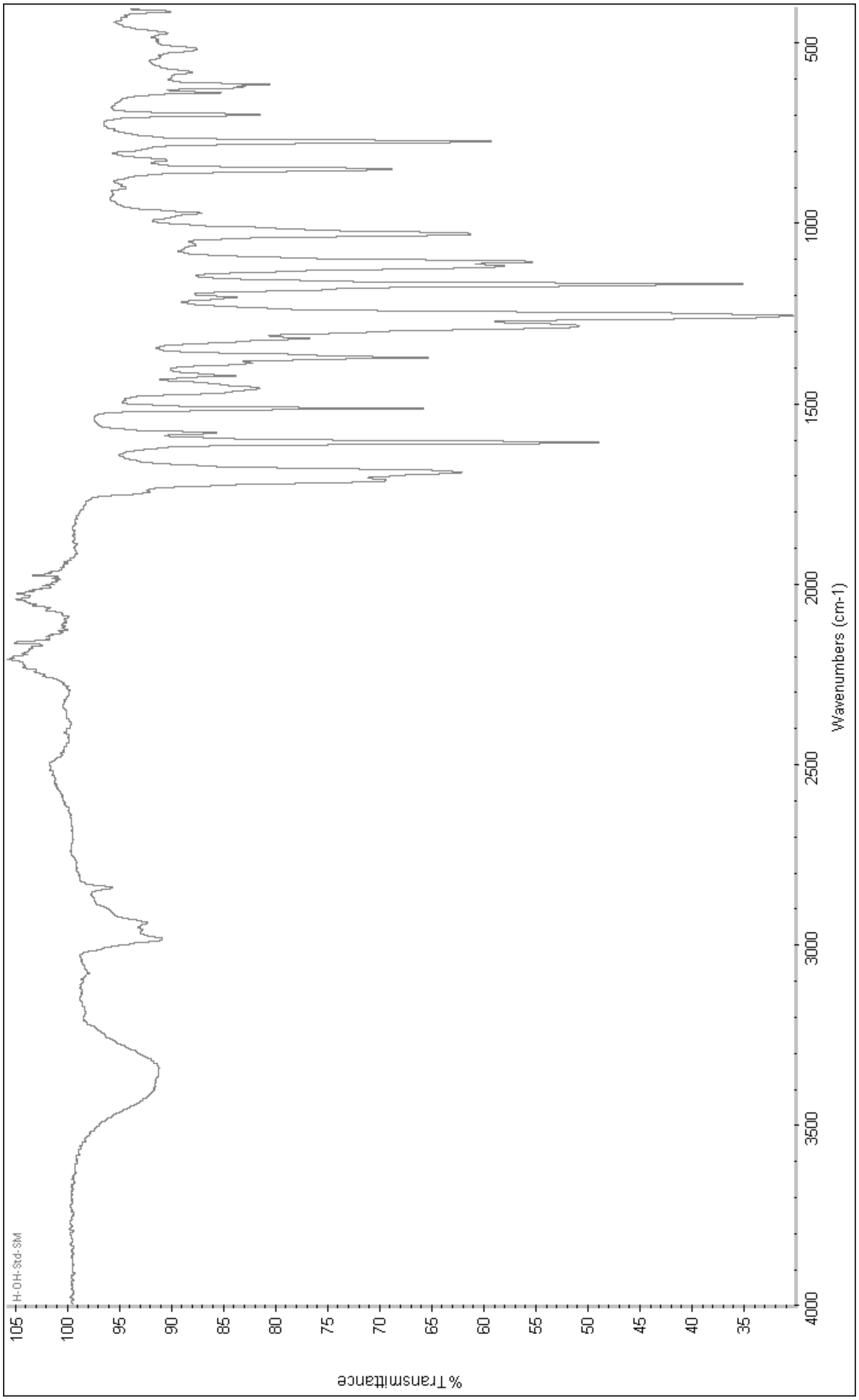
H-OH-Std-SM
%Transmittance
Wavenumbers (cm-1)

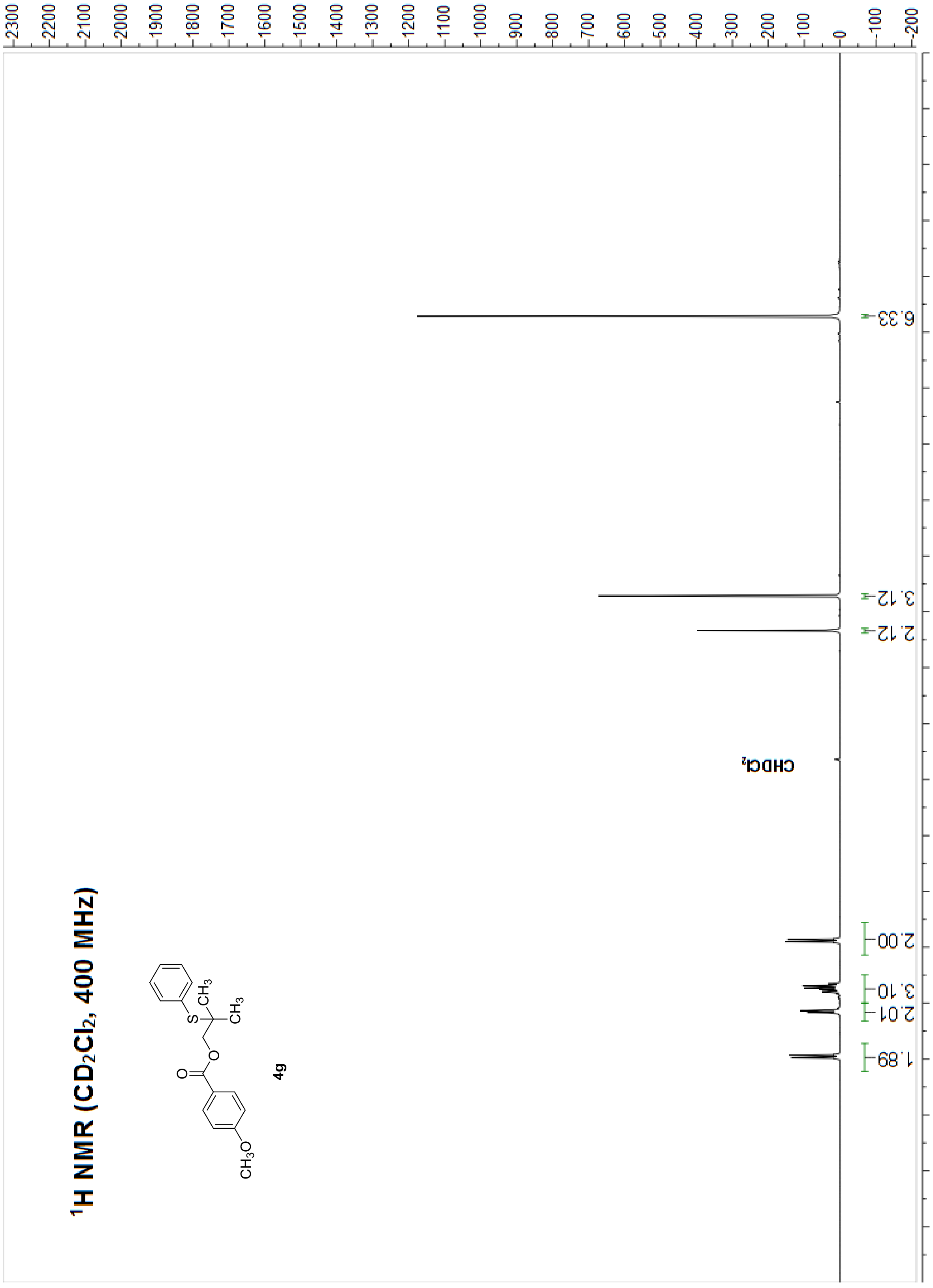
1H NMR (CD2Cl2, 400 MHz)
4g
CHDCl2
f1 (ppm)
6.33
3.12
2.12
2.00
3.10
2.01
1.89

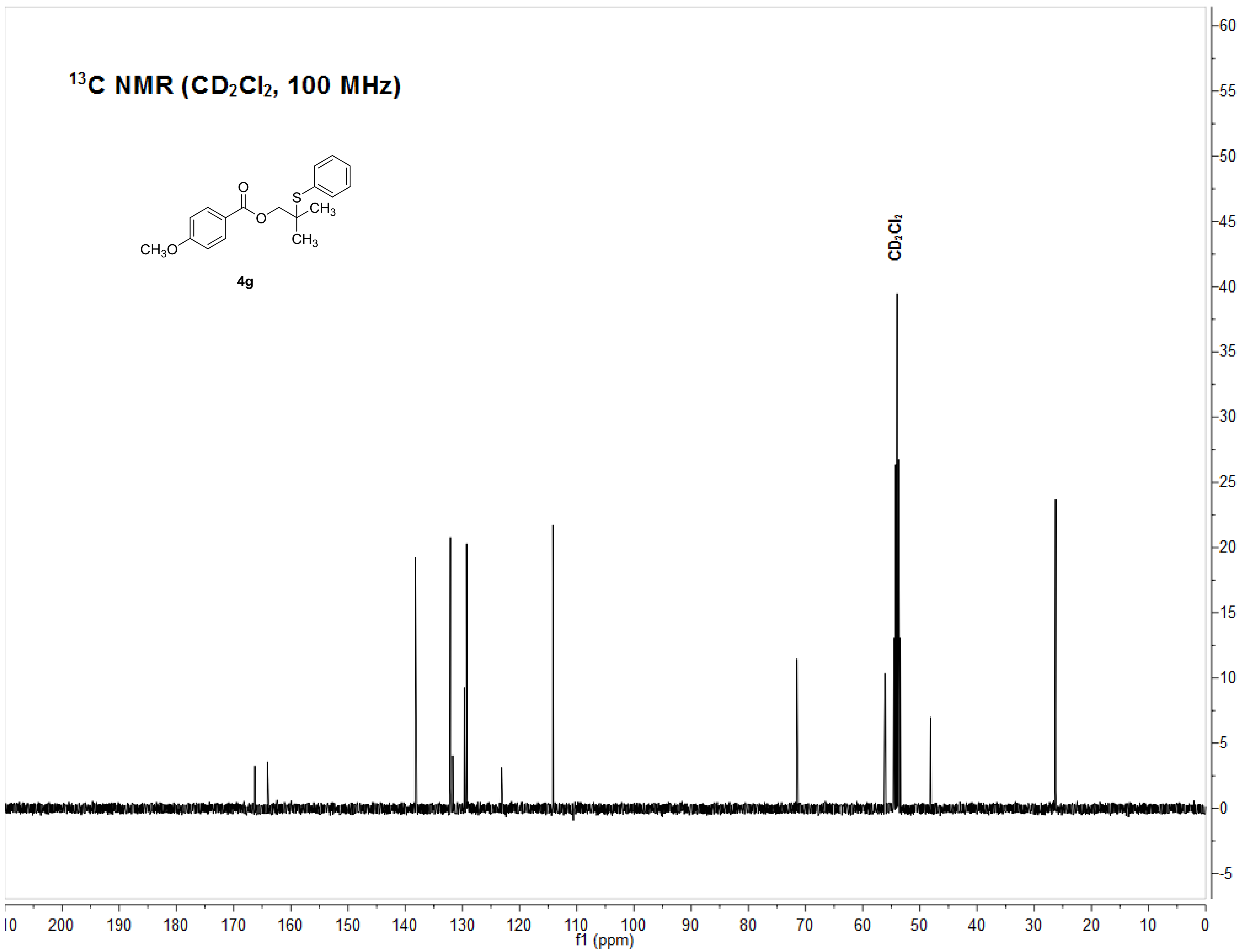

13C NMR (CD2Cl2, 100 MHz)
4g
CH3O
O
S
CH3
CD2Cl2
f1 (ppm)

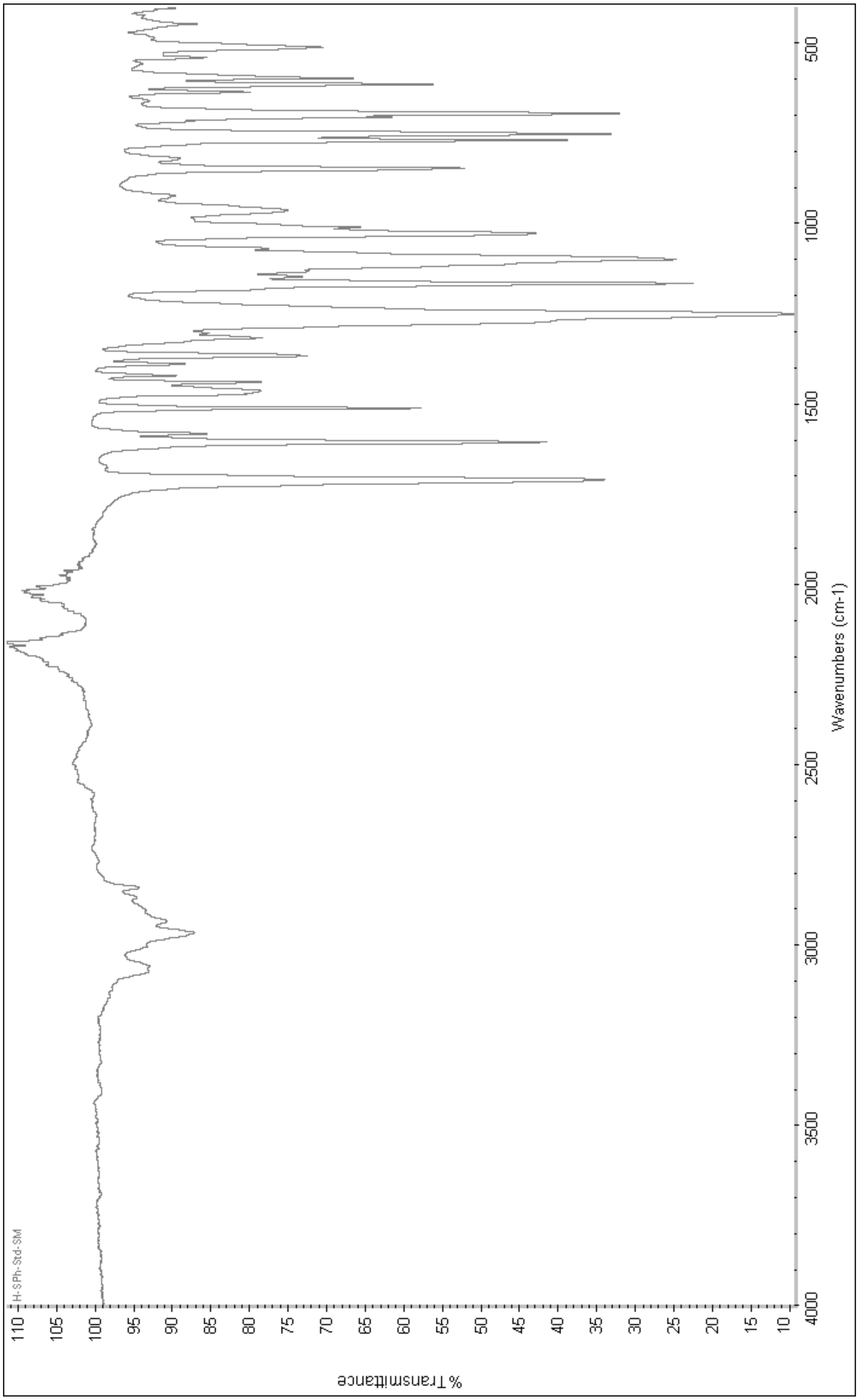
H-SPh-Std-SM
%Transmittance
Wavenumbers (cm-1)

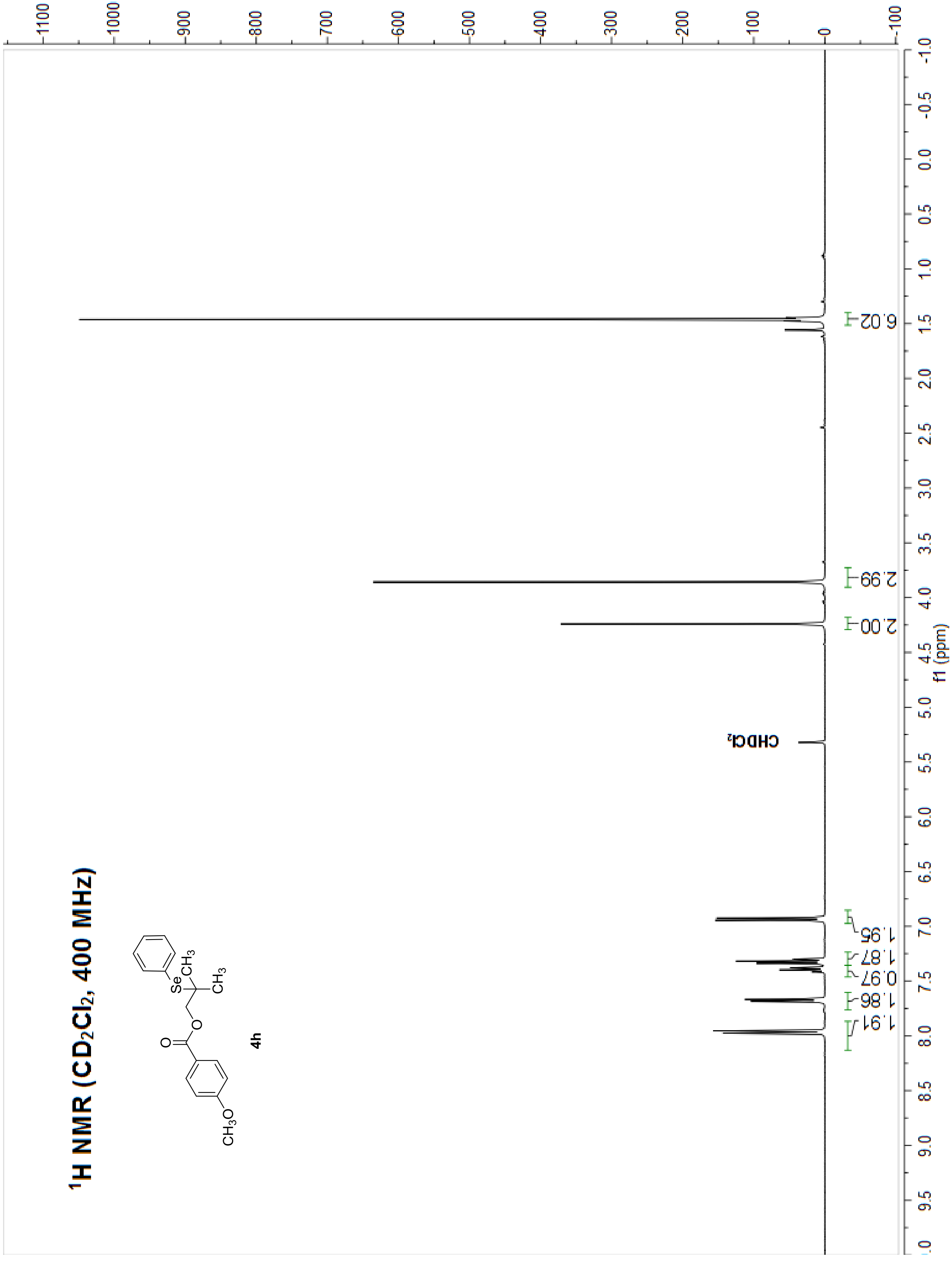
1H NMR (CD2Cl2, 400 MHz)
4h
CHDCl2
6.02
2.99
2.00
1.95
1.87
0.97
1.86
1.91
f1 (ppm)

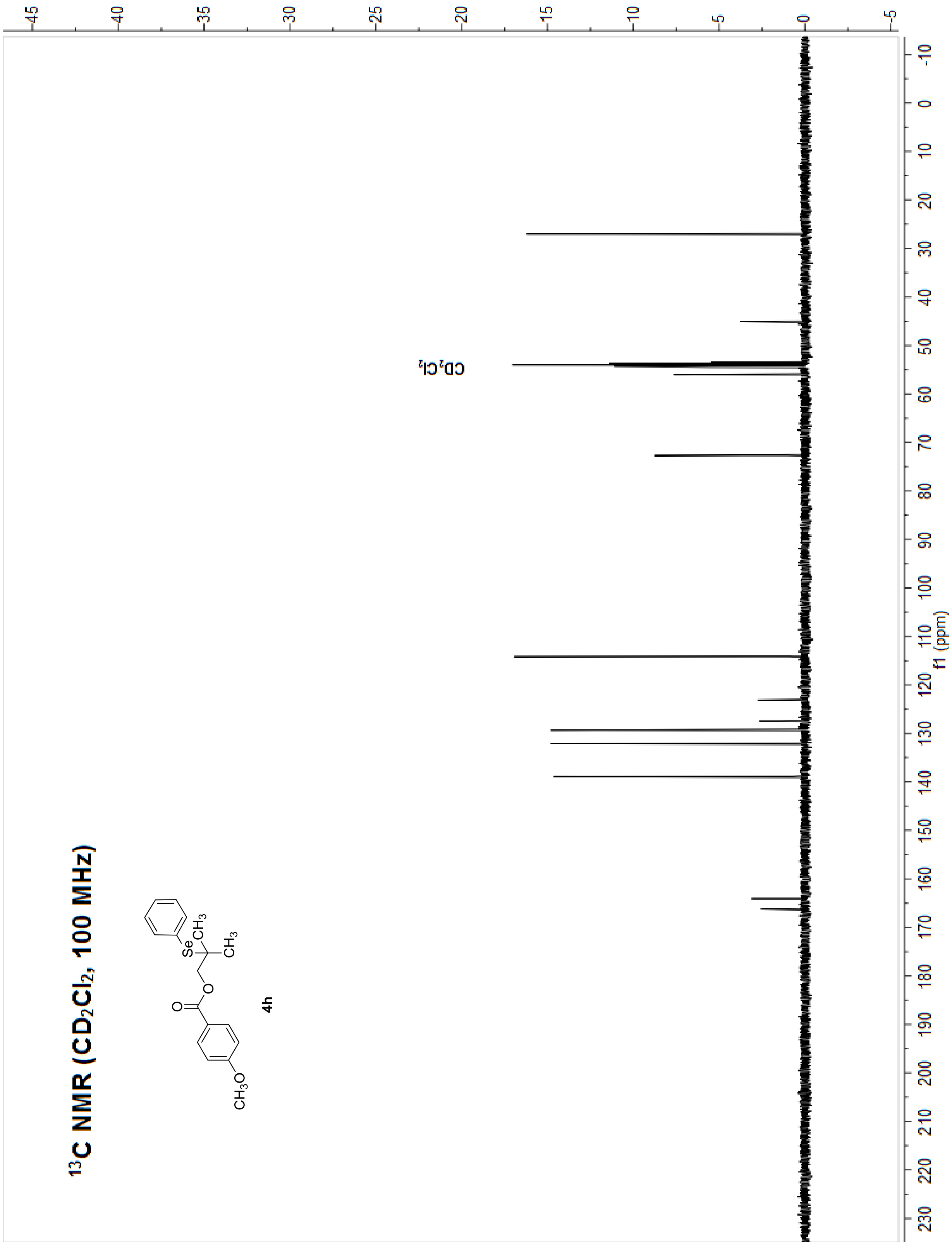

13C NMR (CD2Cl2, 100 MHz)
4h
CD2Cl2
f1 (ppm)

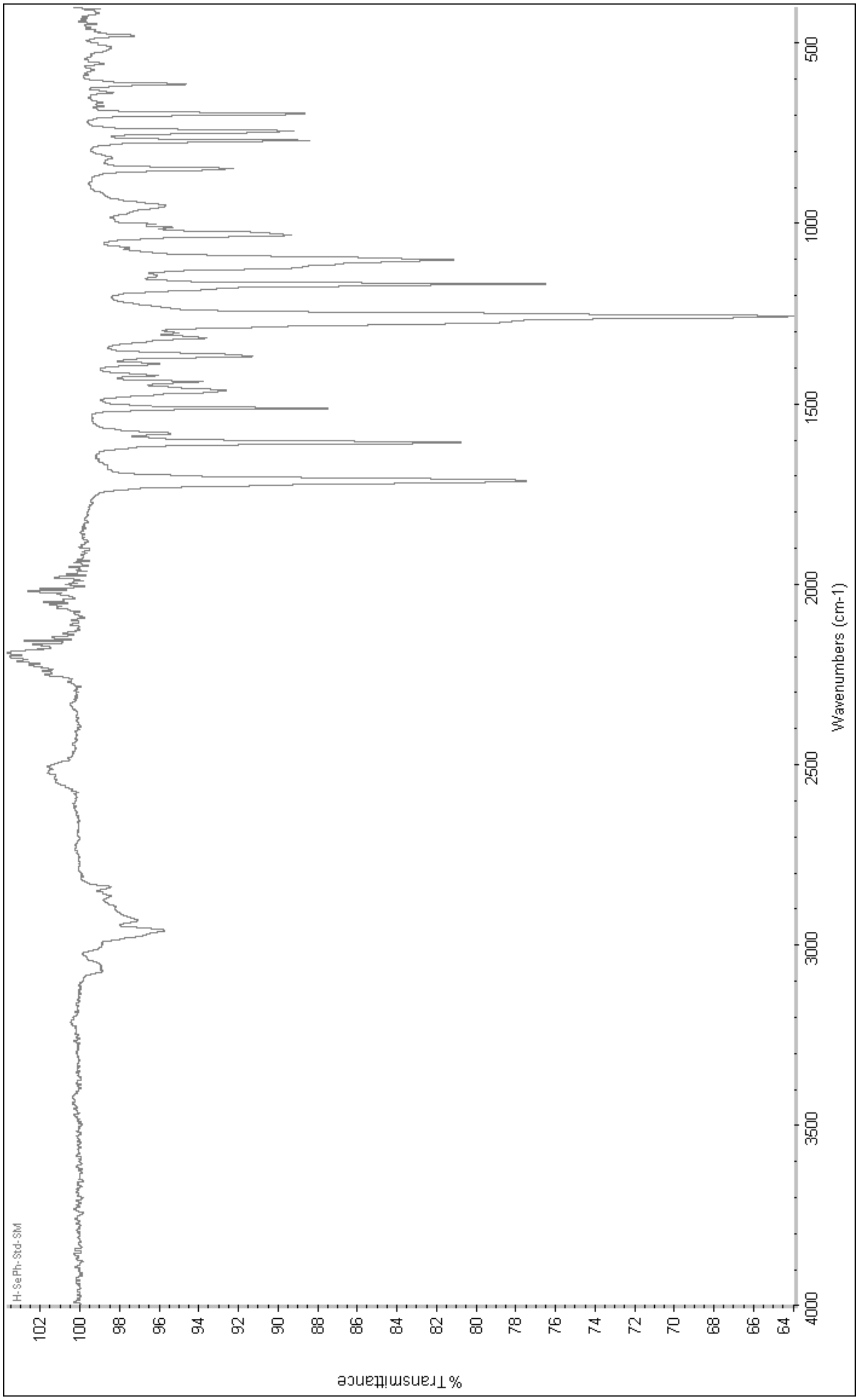
H-SePh-Std-SM
%Transmittance
Wavenumbers (cm-1)

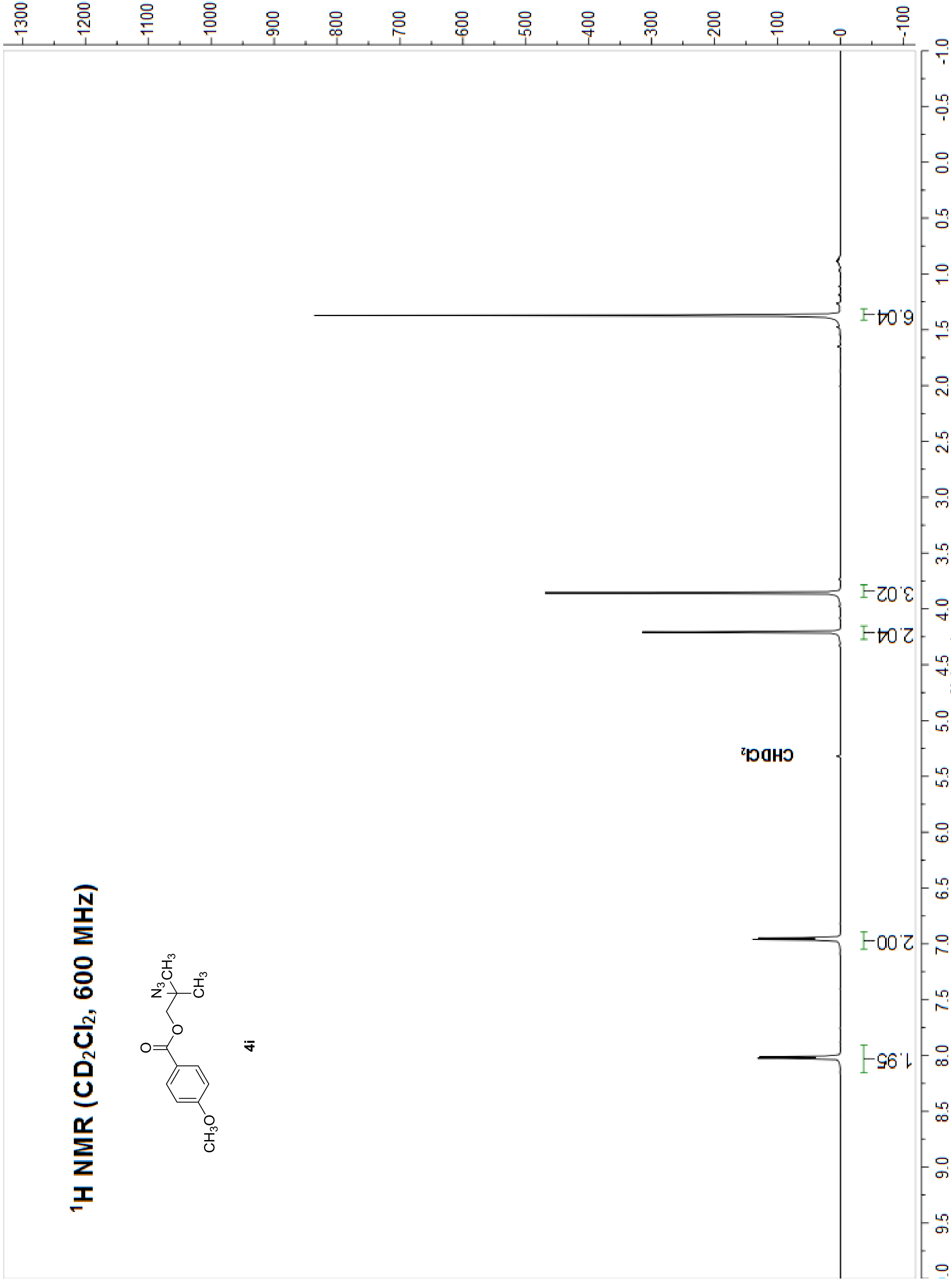

1H NMR (CD2Cl2, 600 MHz)
4i
CHDCl2
6.04
3.02
2.04
2.00
1.95
f1 (ppm)

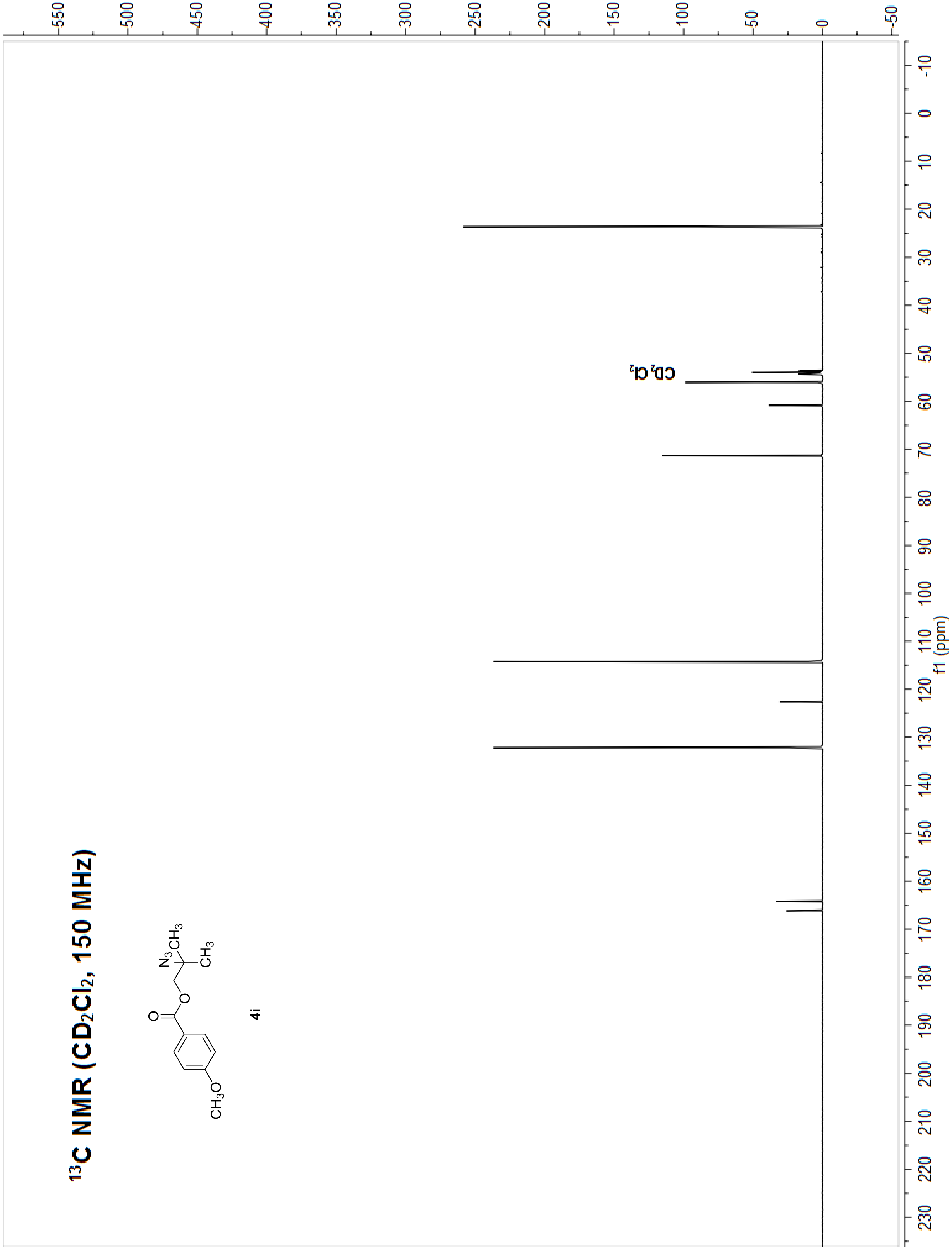
13C NMR (CD2Cl2, 150 MHz)
4i
CD2Cl2
f1 (ppm)

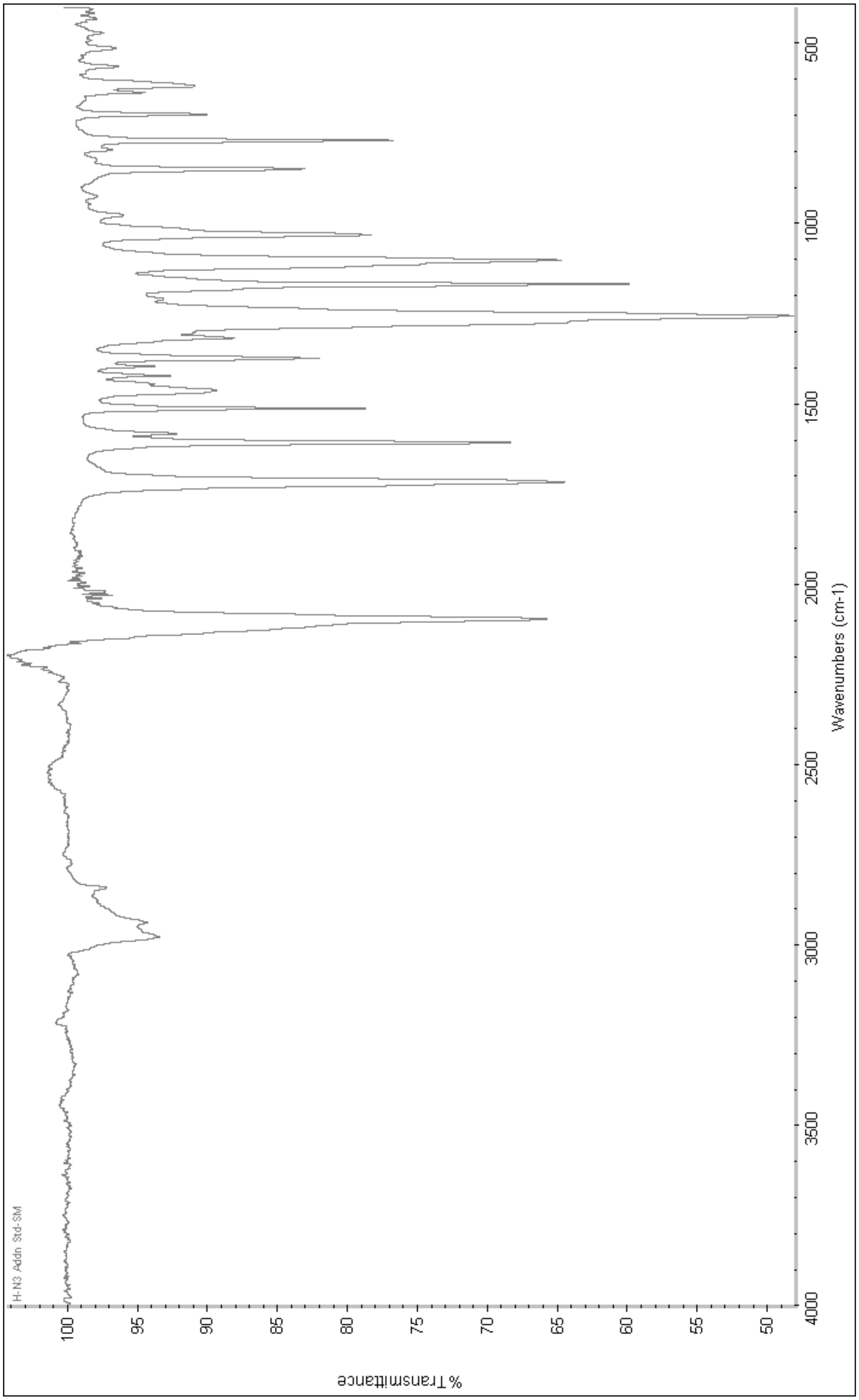
H-N3 Addn Std-SM
%Transmittance
Wavenumbers (cm-1)
100
95
90
85
80
75
70
65
60
55
50
4000
3500
3000
2500
2000
1500
1000
500

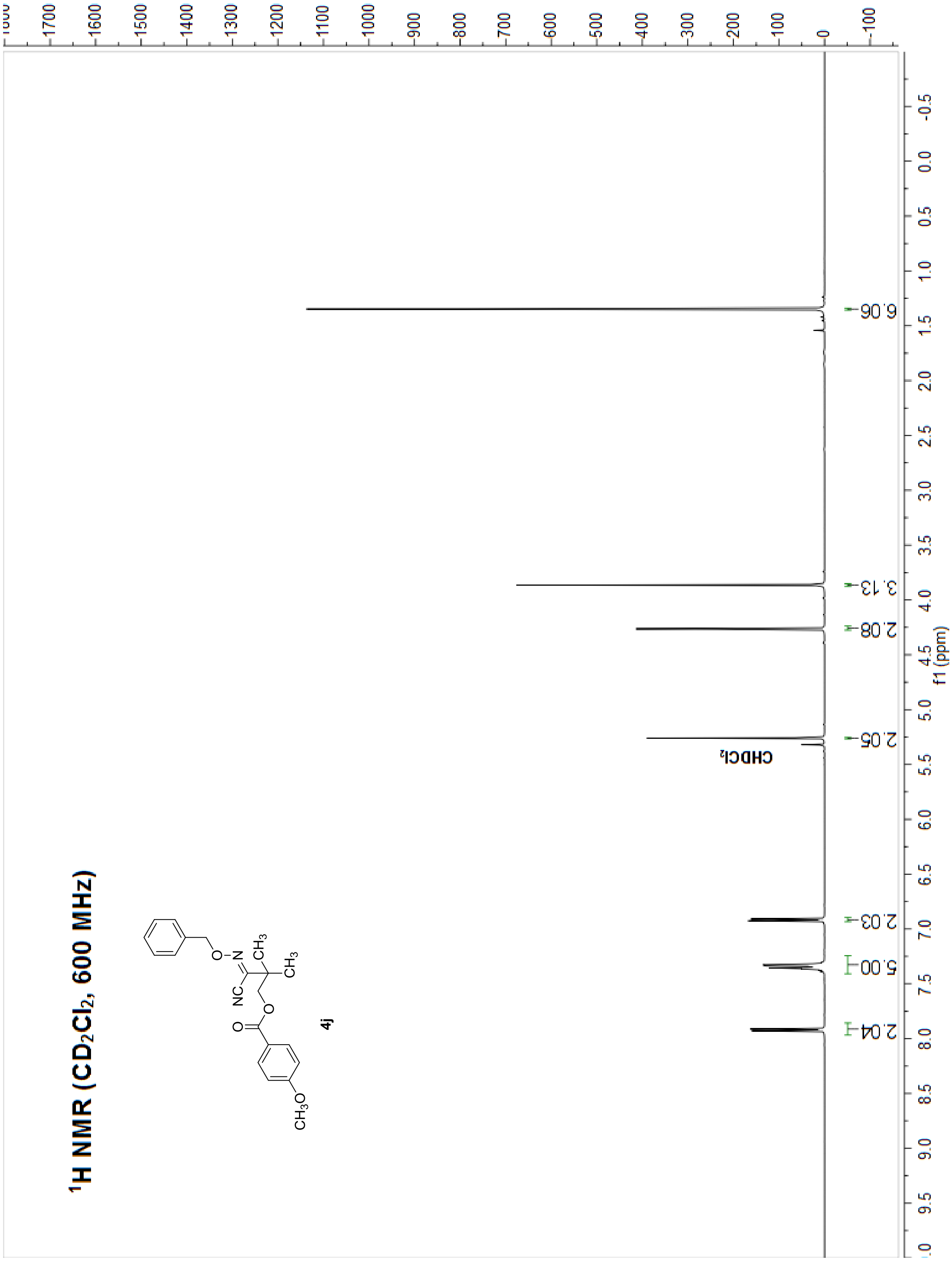
1H NMR (CD2Cl2, 600 MHz)
4j
CHDCl2
f1 (ppm)
6.06
3.13
2.08
2.05
2.03
5.00
2.04

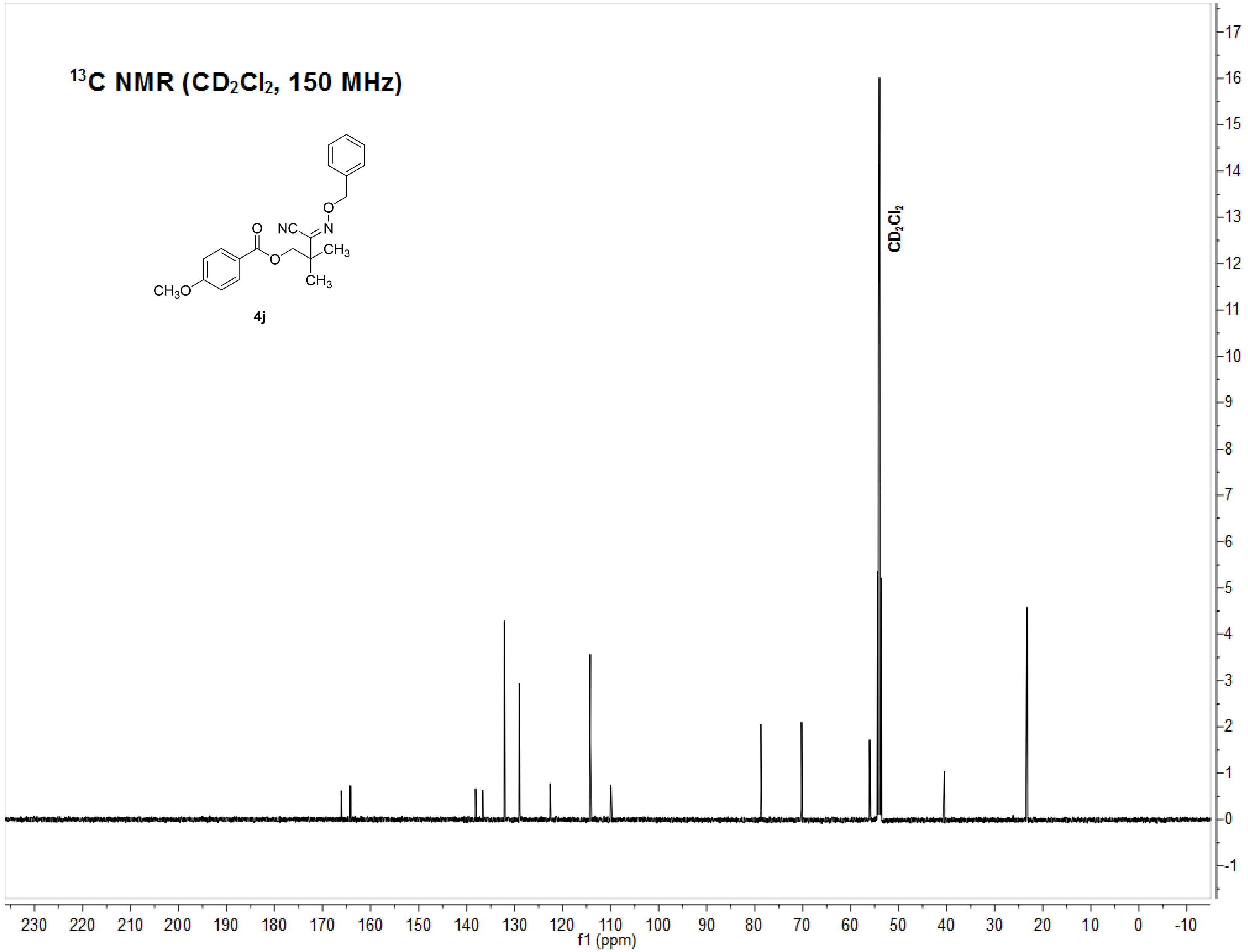

13C NMR (CD2Cl2, 150 MHz)
NC
N
O
O
O
CH3
CH3
CH3O
4j
CD2Cl2
f1 (ppm)

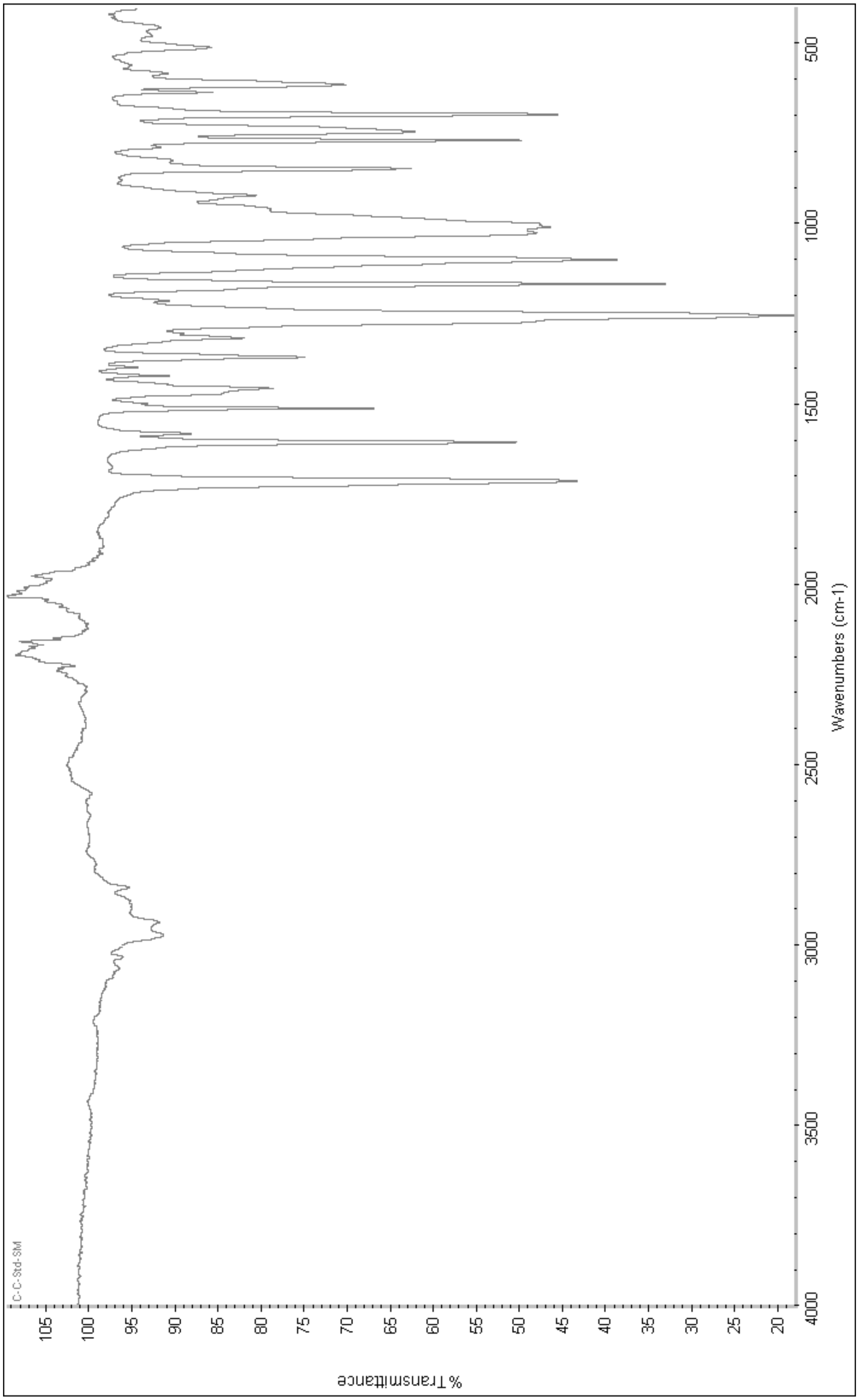
C-C-Std-SM
%Transmittance
Wavenumbers (cm-1)

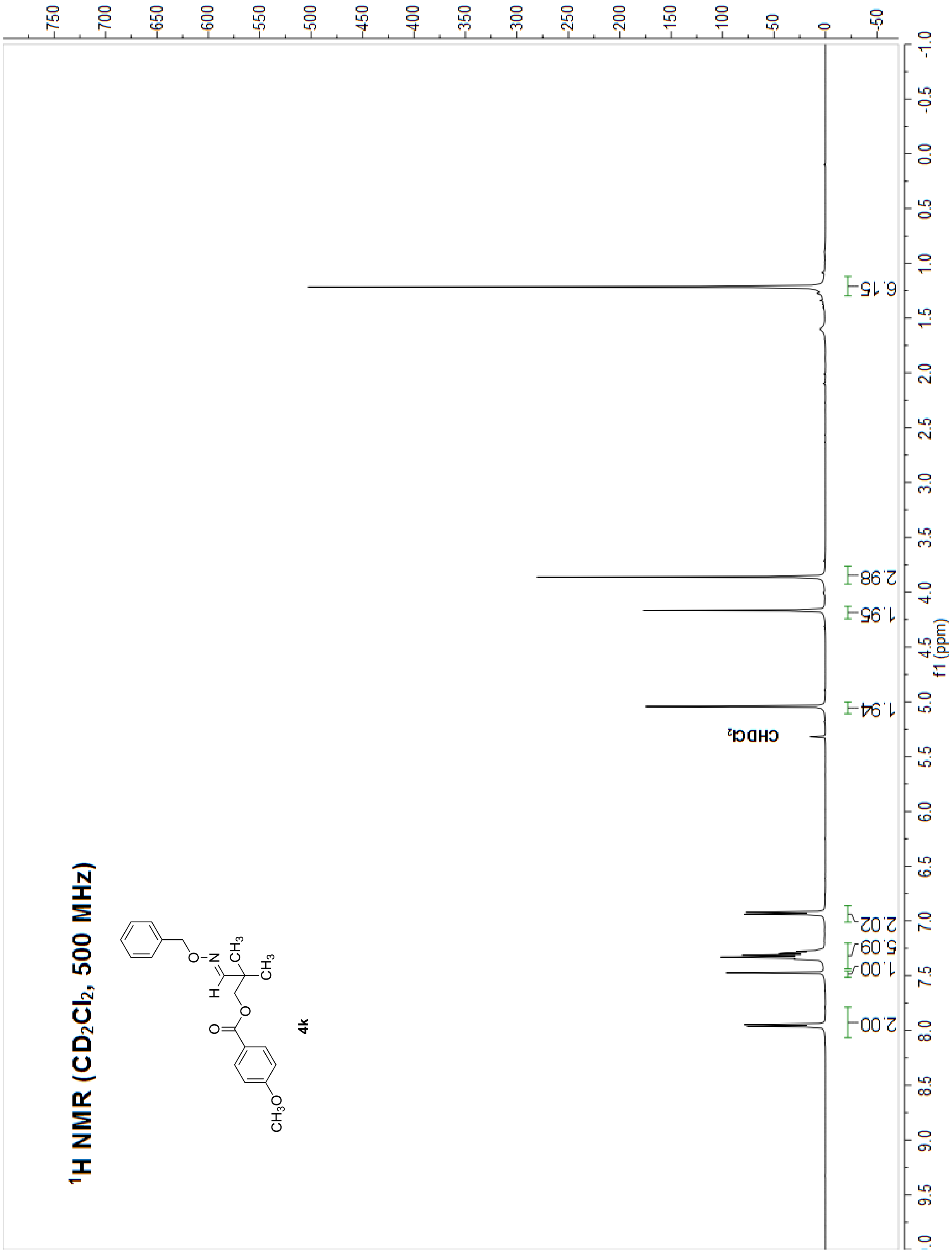
¹H NMR (CD₂Cl₂, 500 MHz)
4k
CHDCl₂
f1 (ppm)

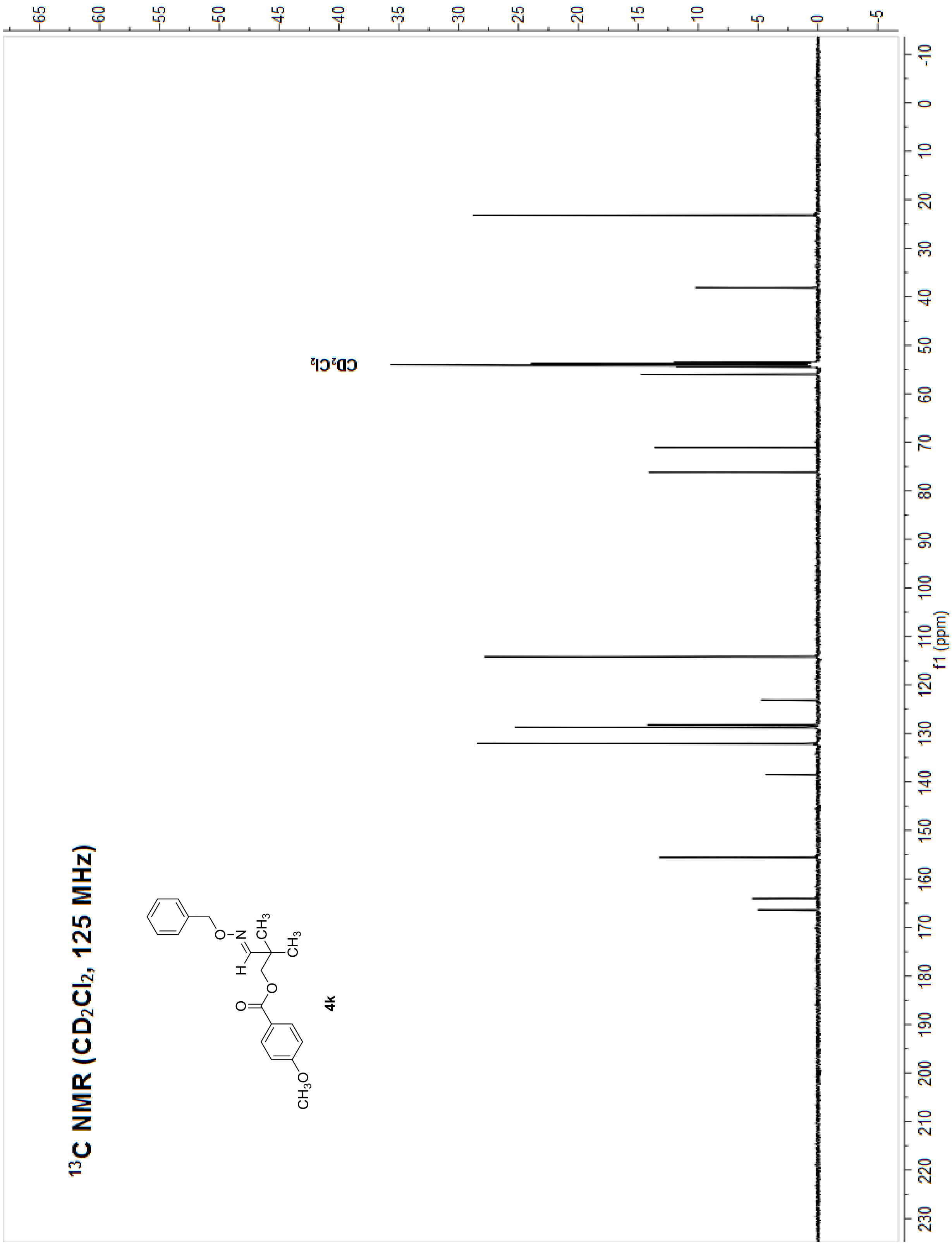

13C NMR (CD2Cl2, 125 MHz)
4k
CD2Cl2
f1 (ppm)

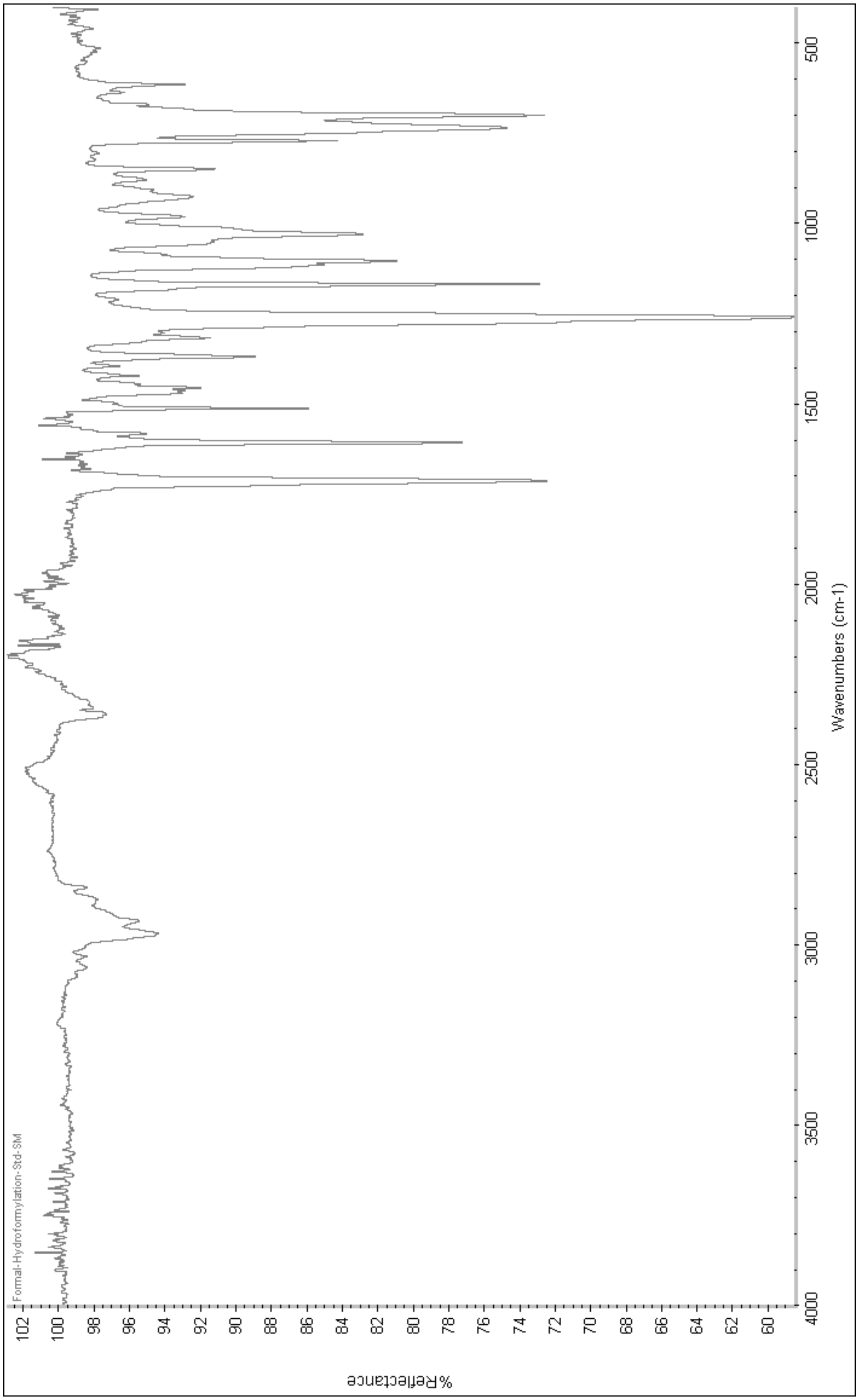
Formal-Hydroformylation-Std-SM
%Reflectance
Wavenumbers (cm-1)

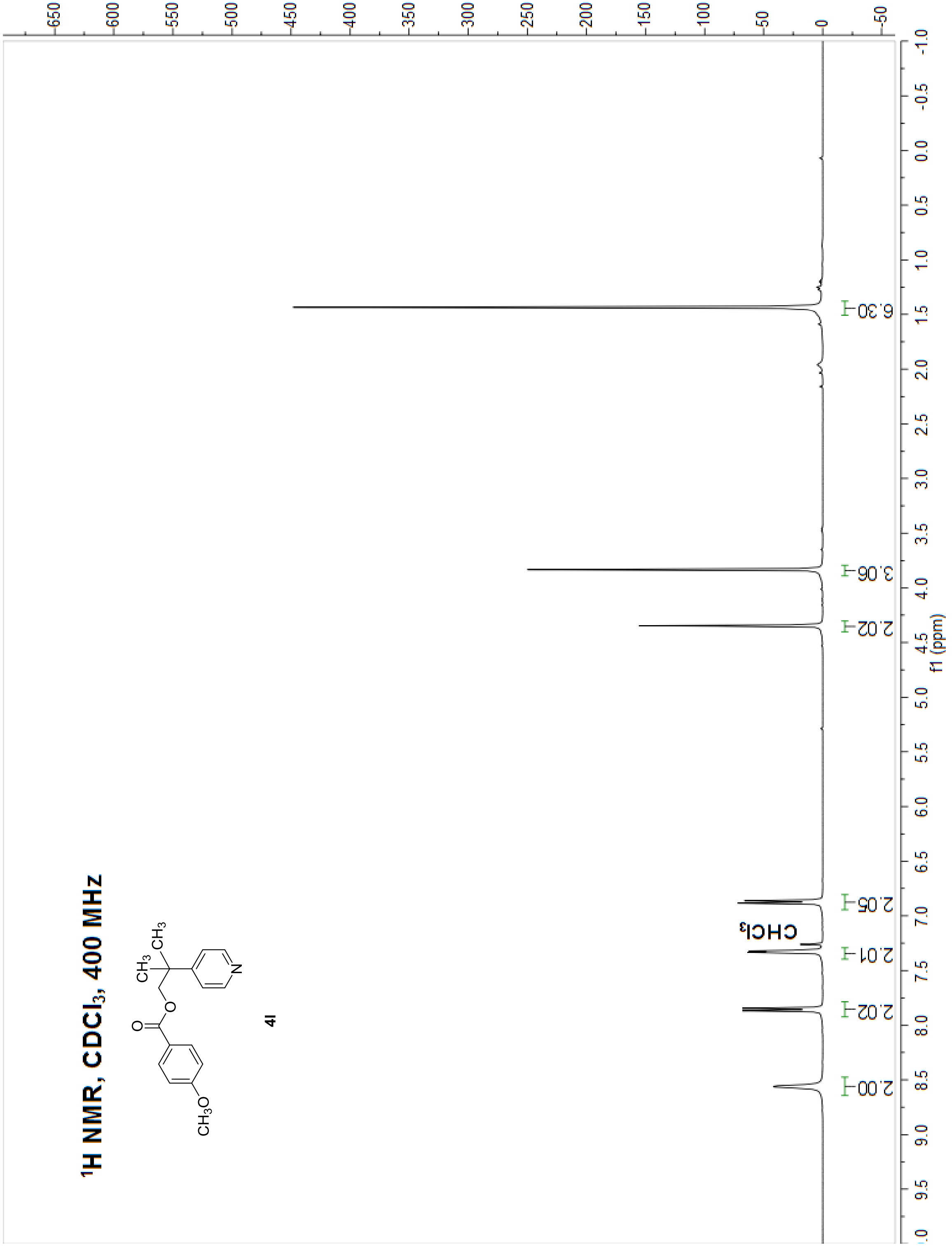
1H NMR, CDCl3, 400 MHz
4l
CHCl3
6.30
3.06
2.02
2.05
2.01
2.02
2.00
f1 (ppm)

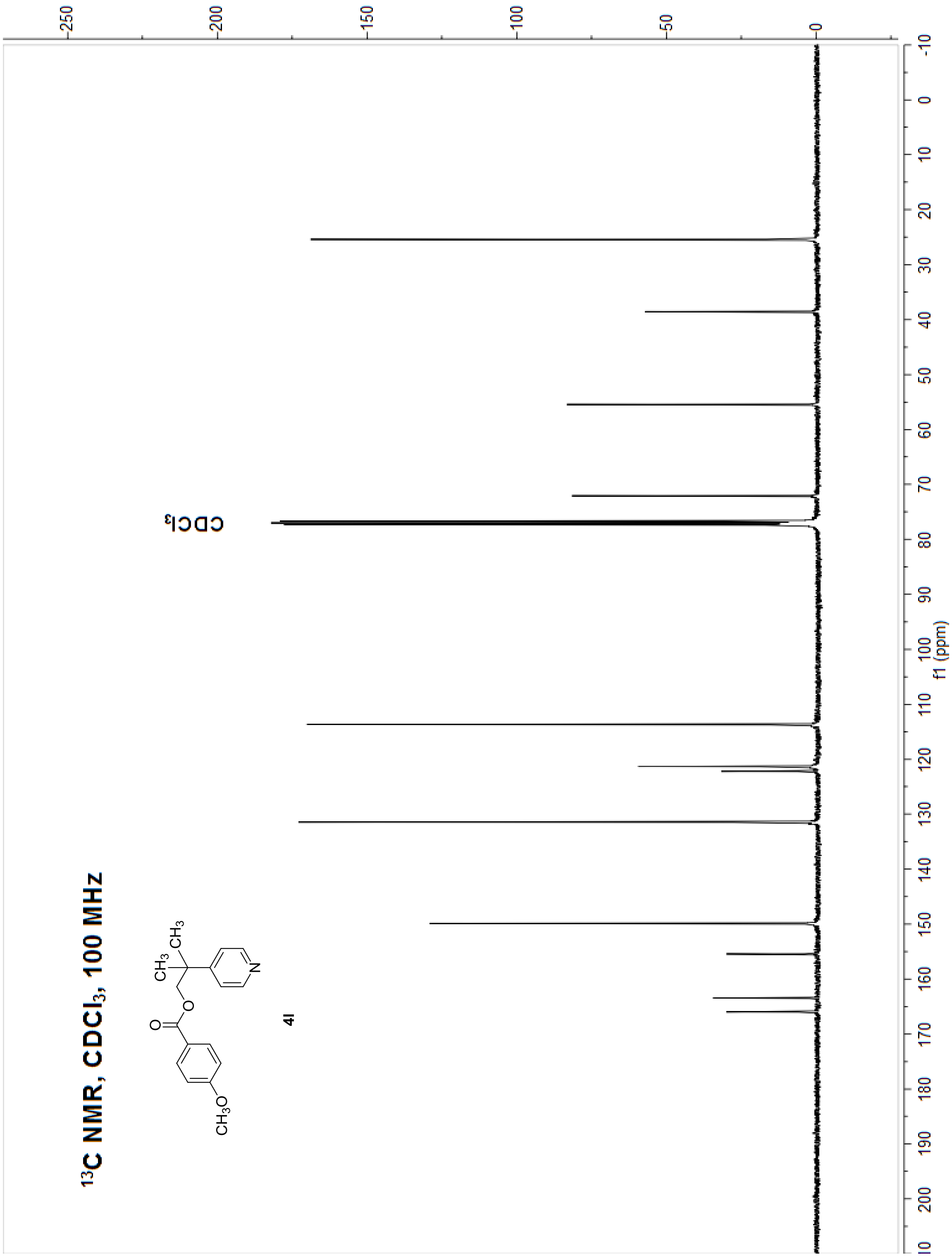
13C NMR, CDCl3, 100 MHz
CH3
CH3
CH3O
O
O
N
4l
CDCl3
f1 (ppm)

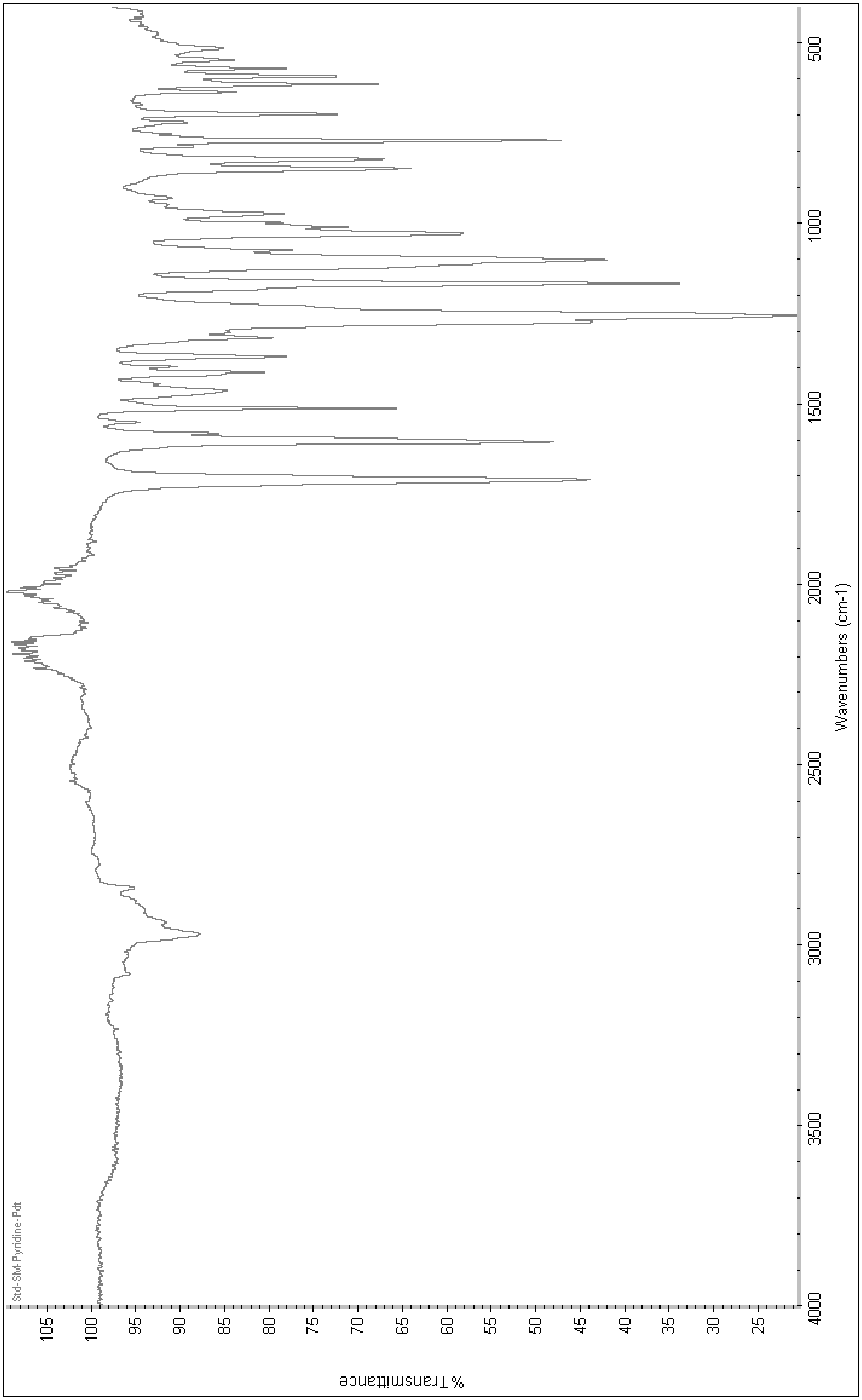
Std-SM-Pyridine-Pdt
%Transmittance
Wavenumbers (cm-1)

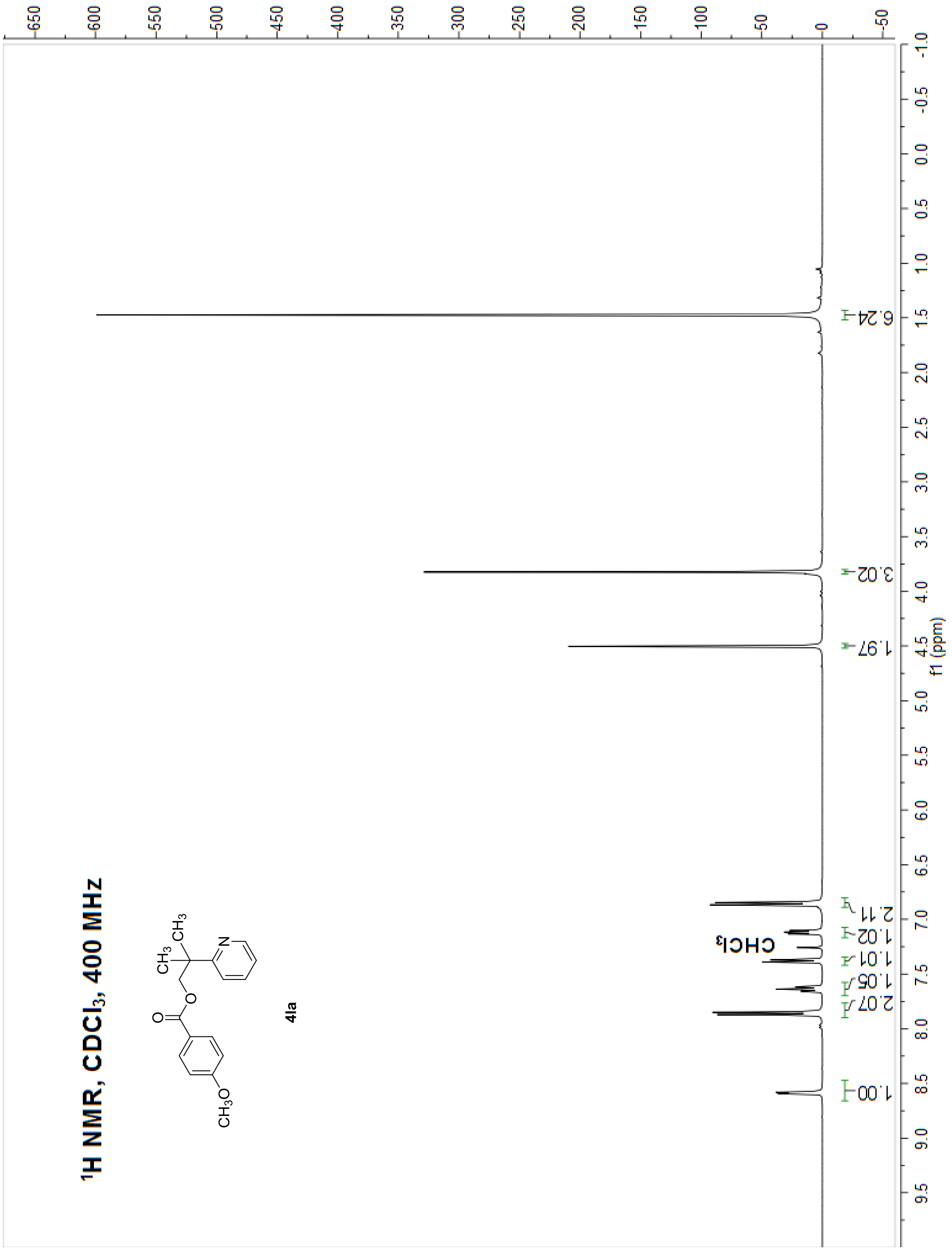
1H NMR, CDCl3, 400 MHz
4la
CHCl3
f1 (ppm)
6.24
3.02
1.97
2.11
1.02
1.01
1.05
2.07
1.00

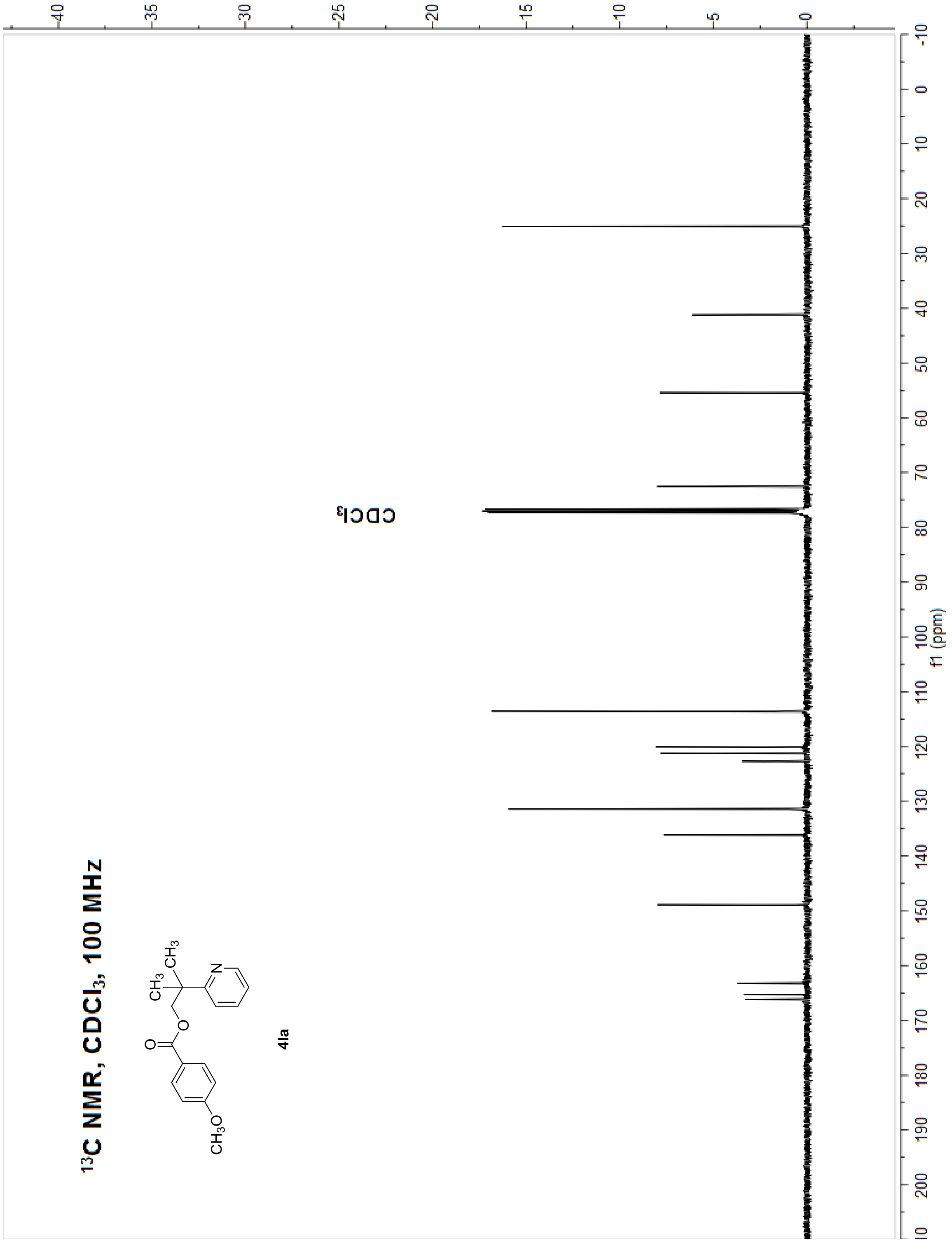

13C NMR, CDCl3, 100 MHz
CH3O
O
O
CH3
CH3
N
4la
CDCl3
f1 (ppm)

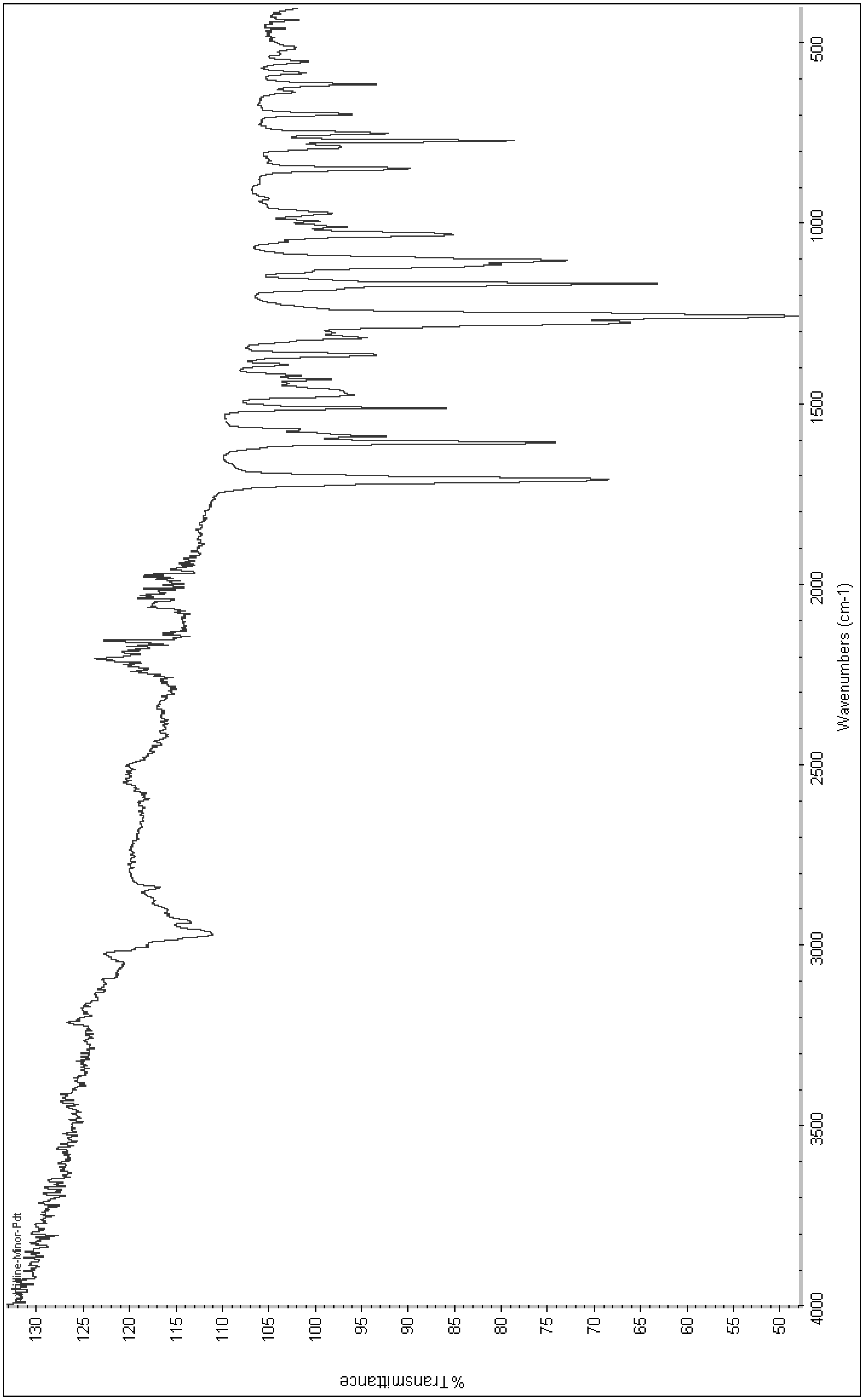
%Transmittance
Wavenumbers (cm-1)

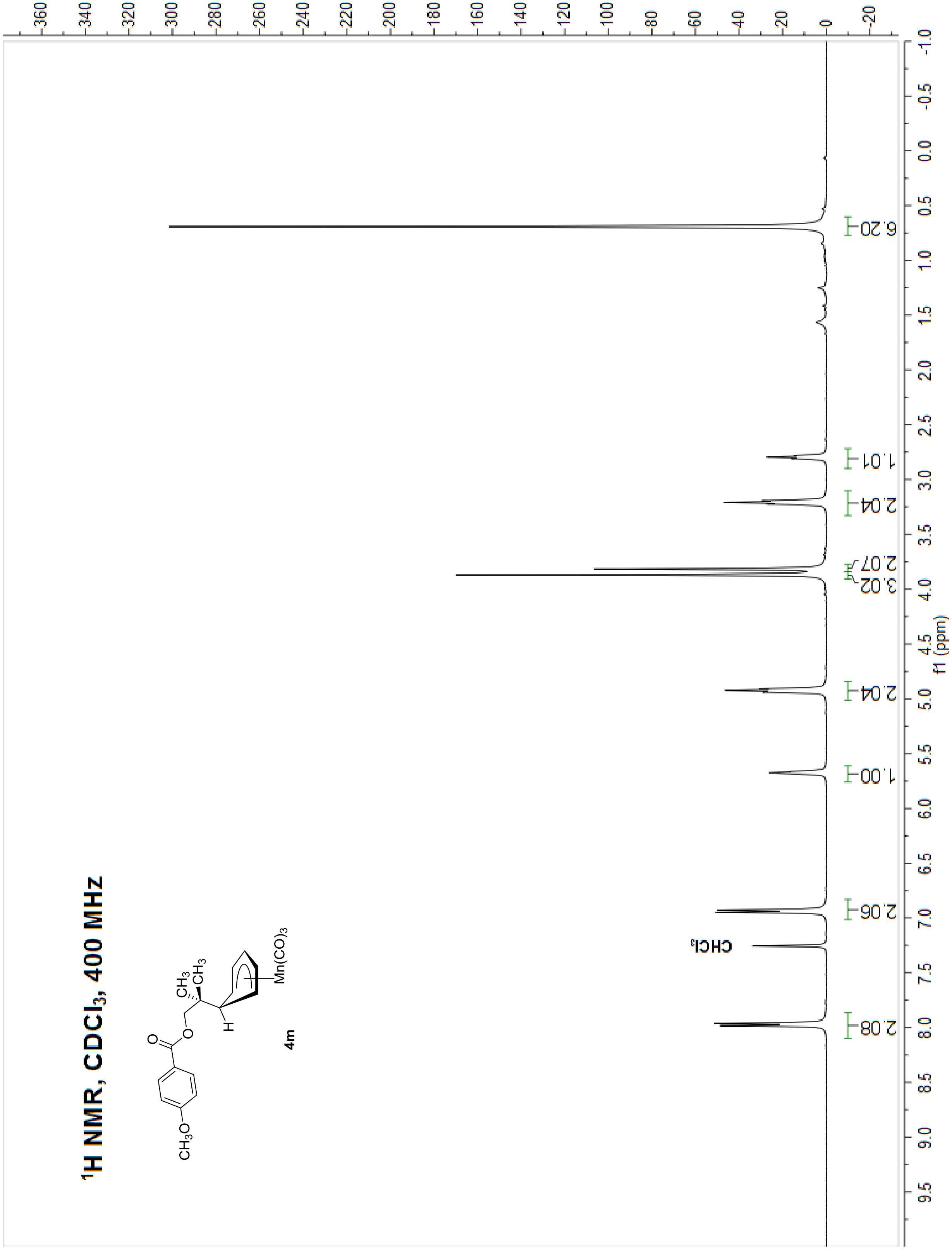

1H NMR, CDCl3, 400 MHz
4m
CHCl3
f1 (ppm)

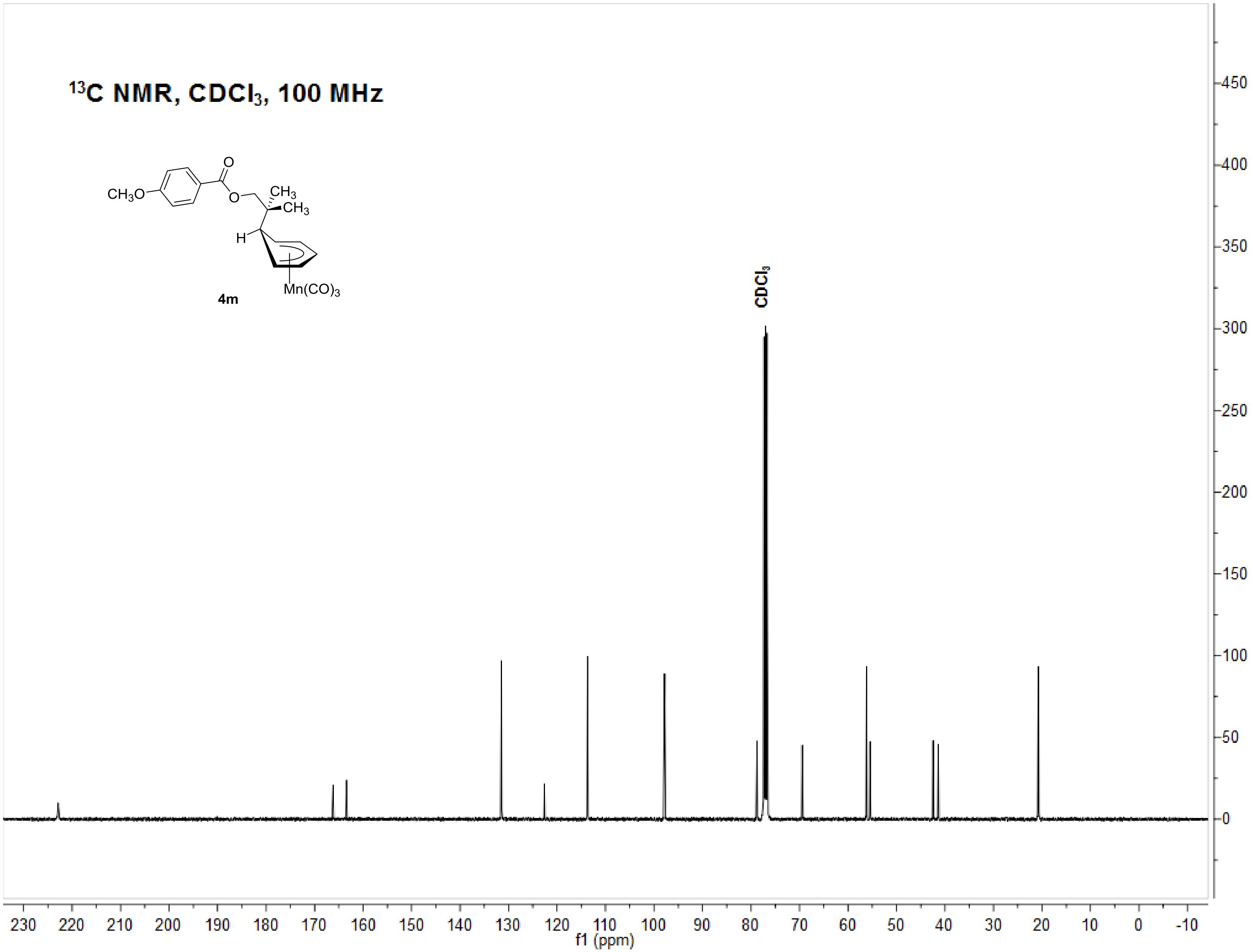

^{13}C NMR, CDCl$_3$, 100 MHz
CH$_3$O
O
CH$_3$
CH$_3$
H
Mn(CO)$_3$
4m
CDCl$_3$
450
400
350
300
250
200
150
100
50
0
230 220 210 200 190 180 170 160 150 140 130 120 110 100 90 80 70 60 50 40 30 20 10 0 -10
f1 (ppm)

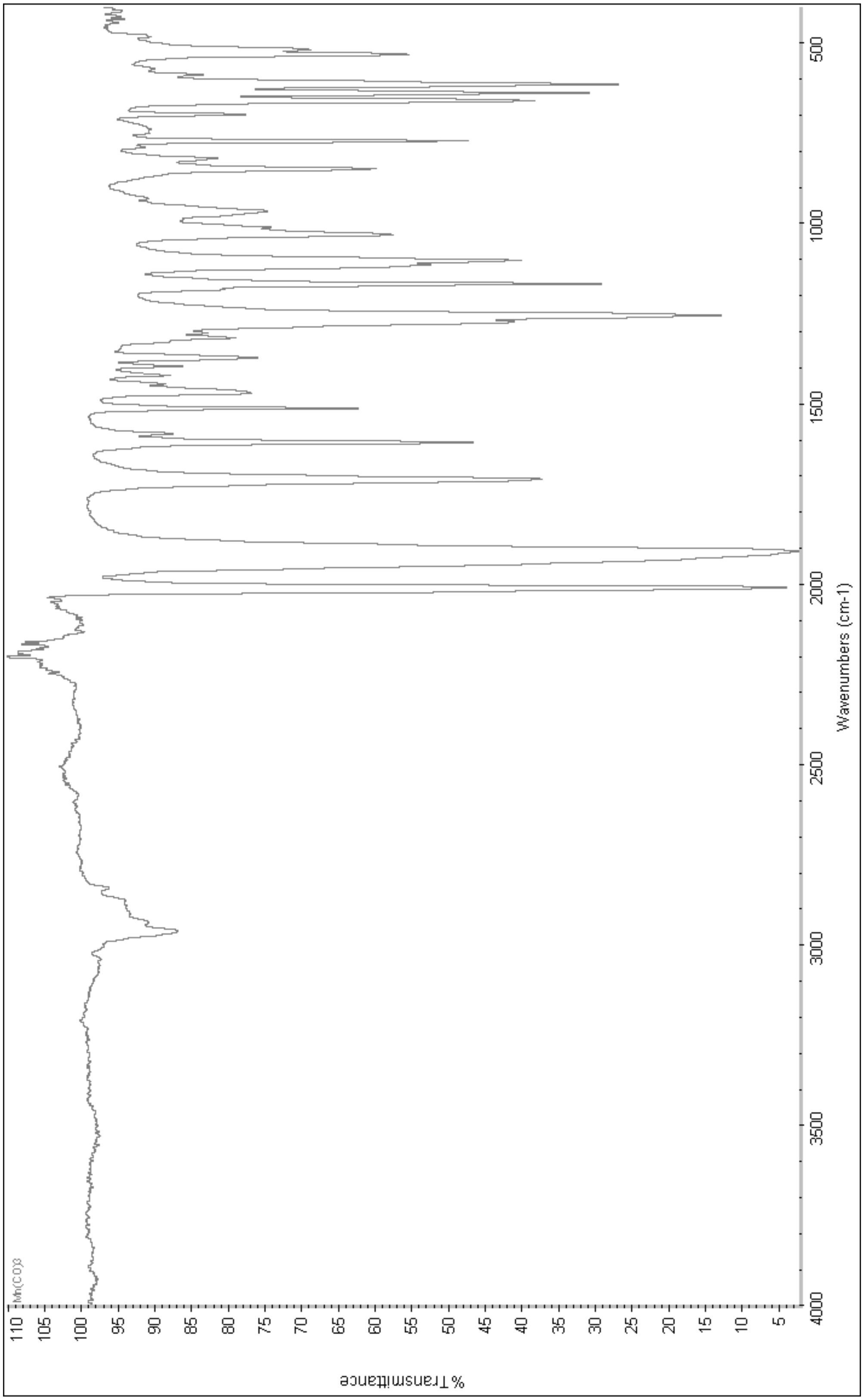
Mn(CO)3
%Transmittance
Wavenumbers (cm-1)

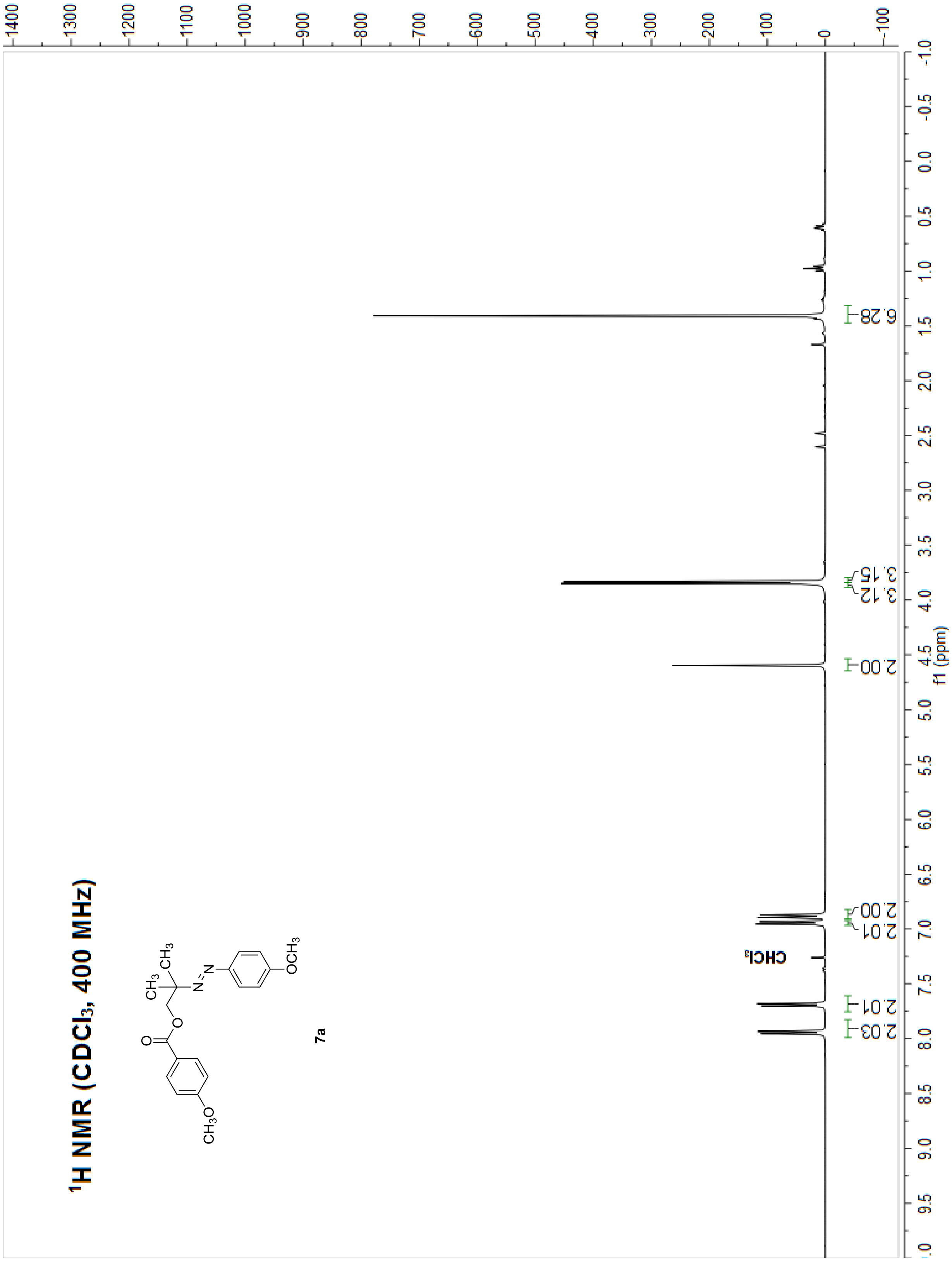

1H NMR (CDCl3, 400 MHz)
7a
CHCl3
f1 (ppm)

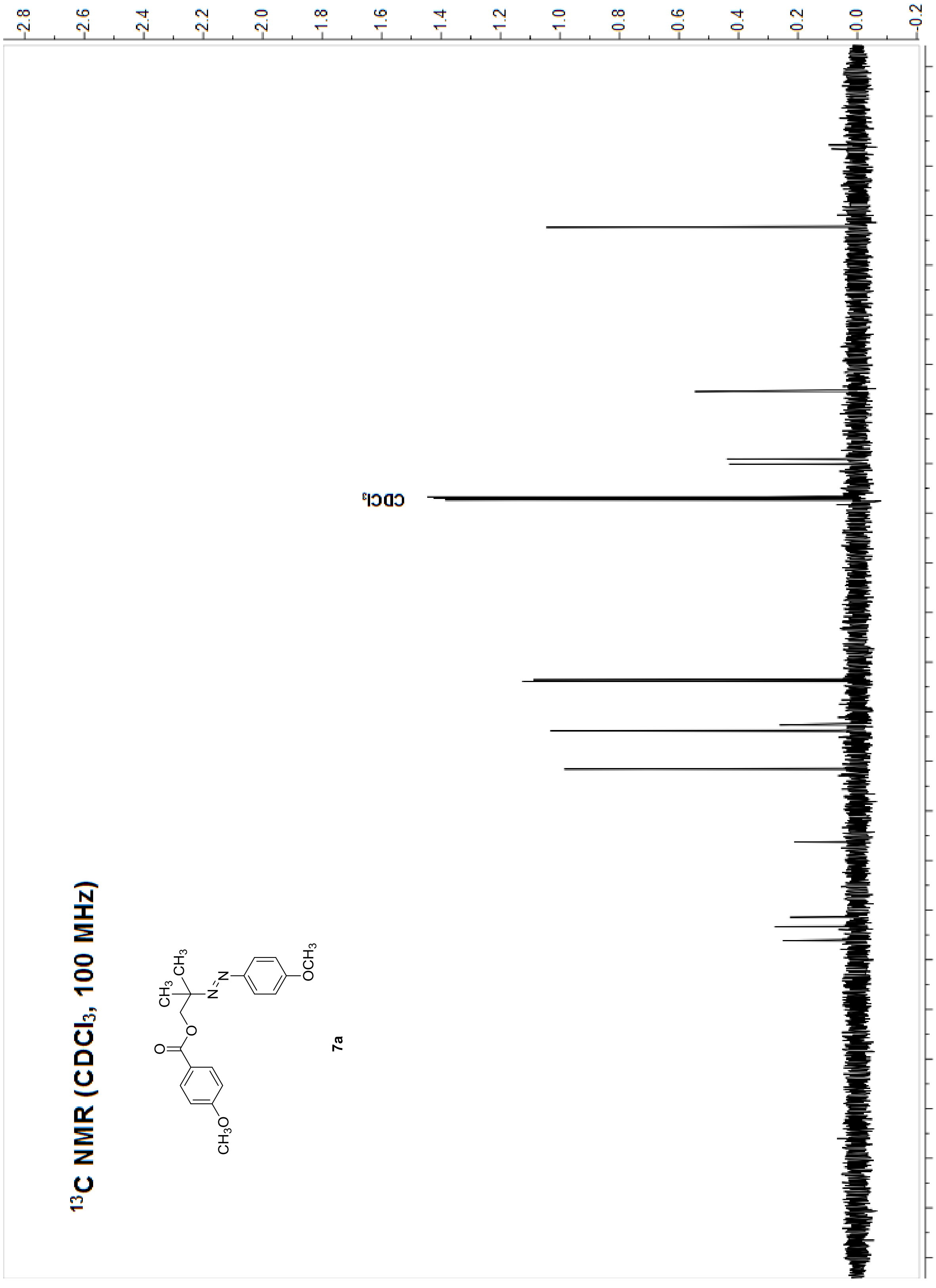

^{13}C NMR (CDCl$_3$, 100 MHz)
7a
CDCl$_3$
f1 (ppm)

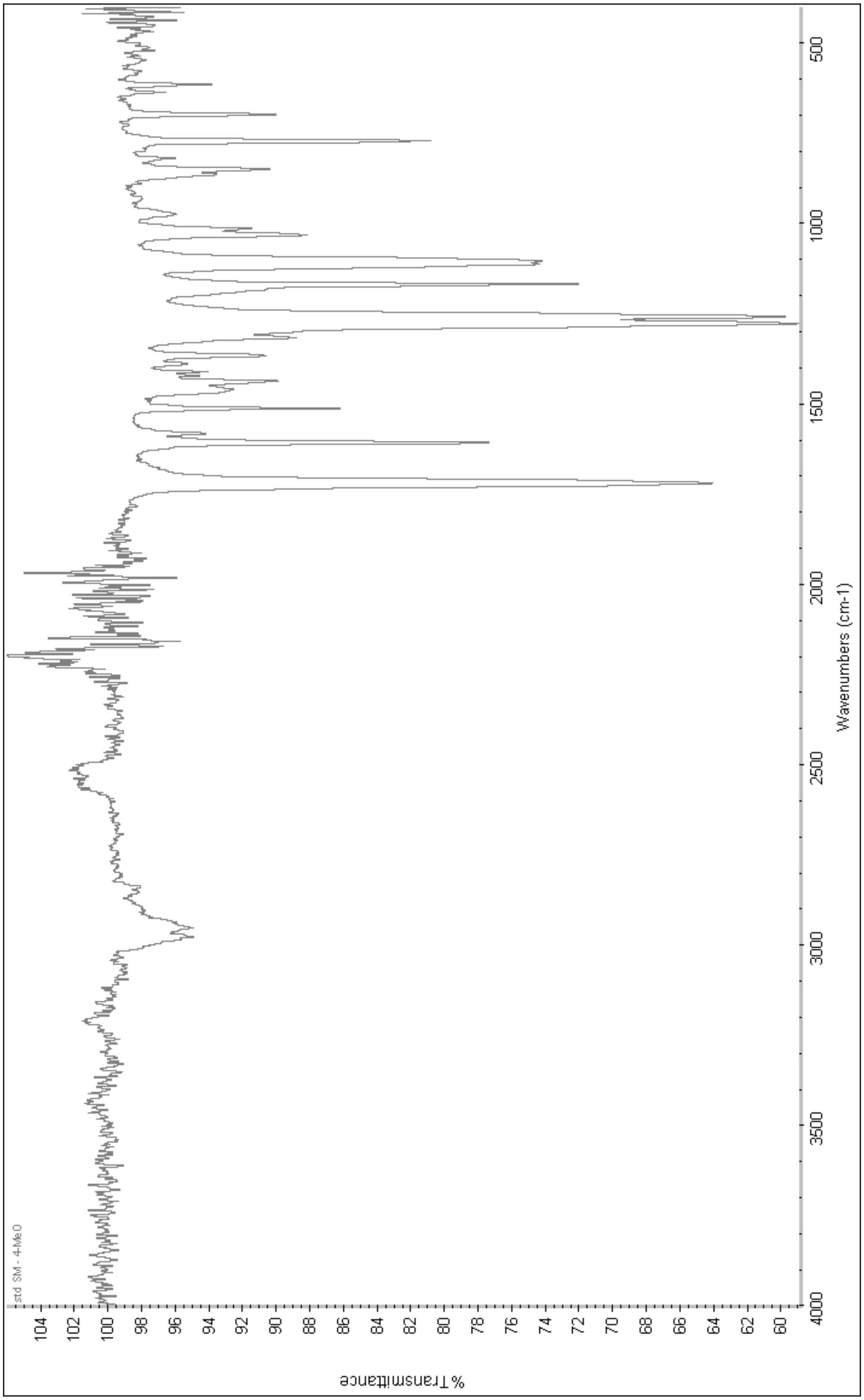
std SM - 4-MeO
%Transmittance
Wavenumbers (cm-1)

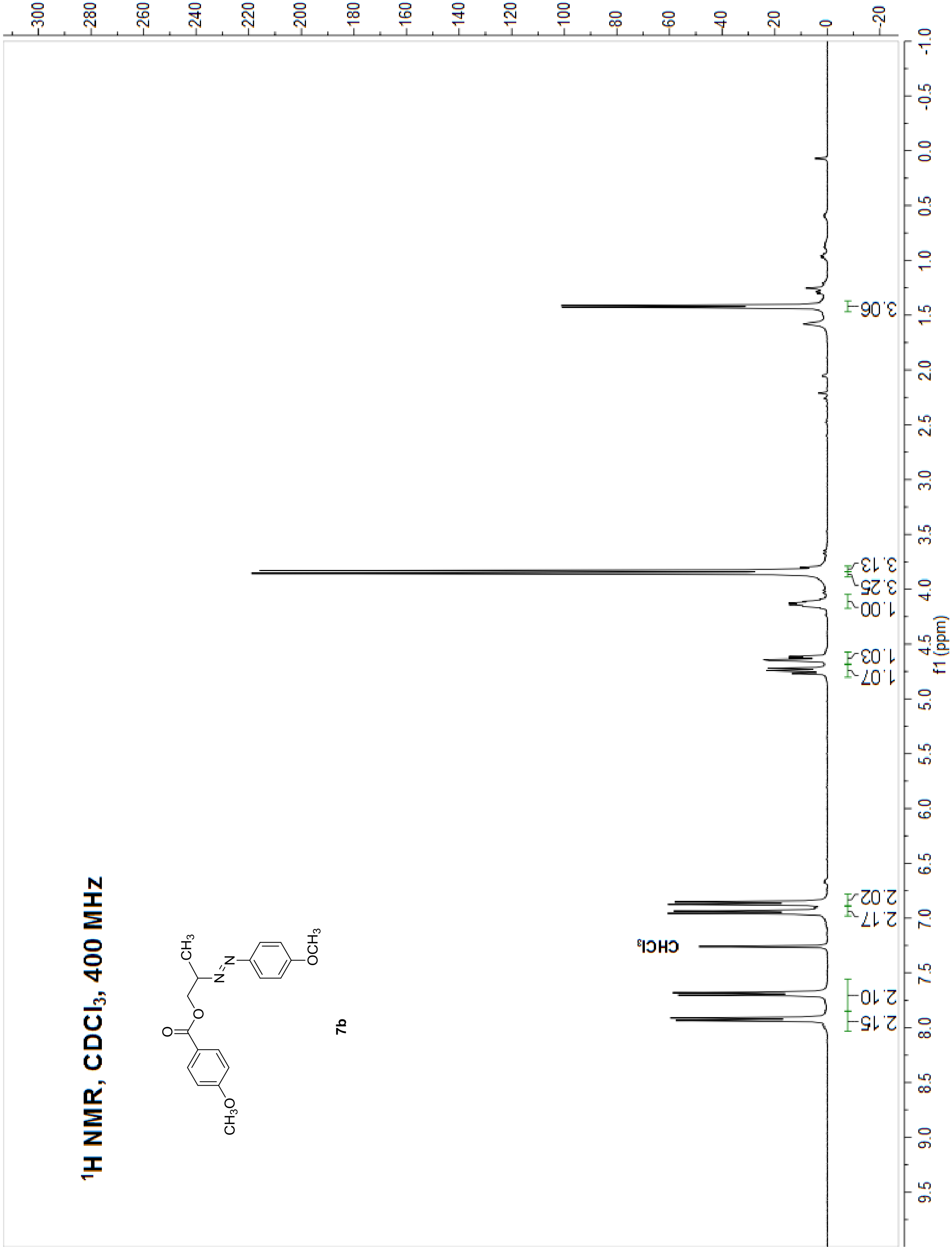

1H NMR, CDCl3, 400 MHz
7b
CHCl3
f1 (ppm)

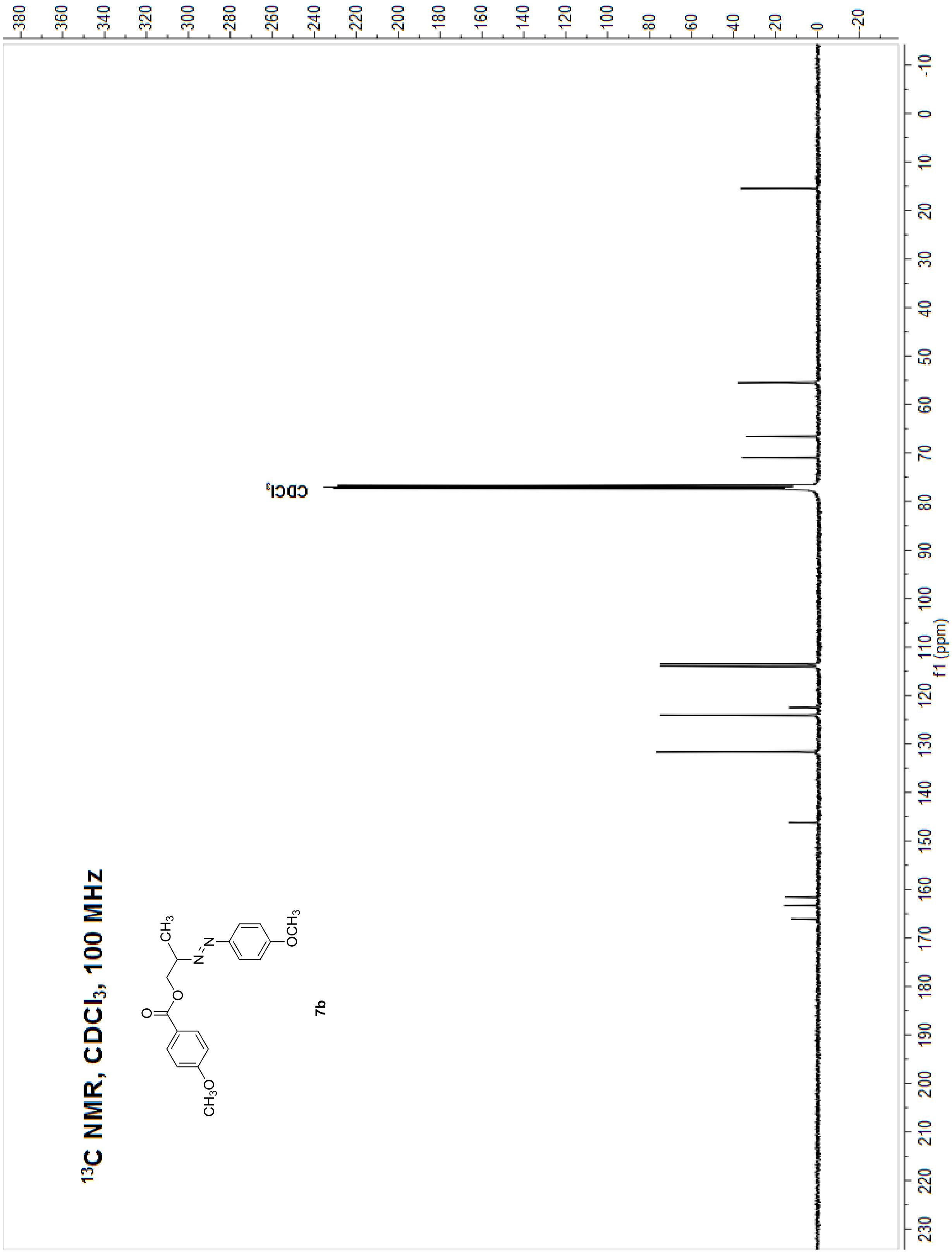

13C NMR, CDCl3, 100 MHz
7b
CDCl3
f1 (ppm)

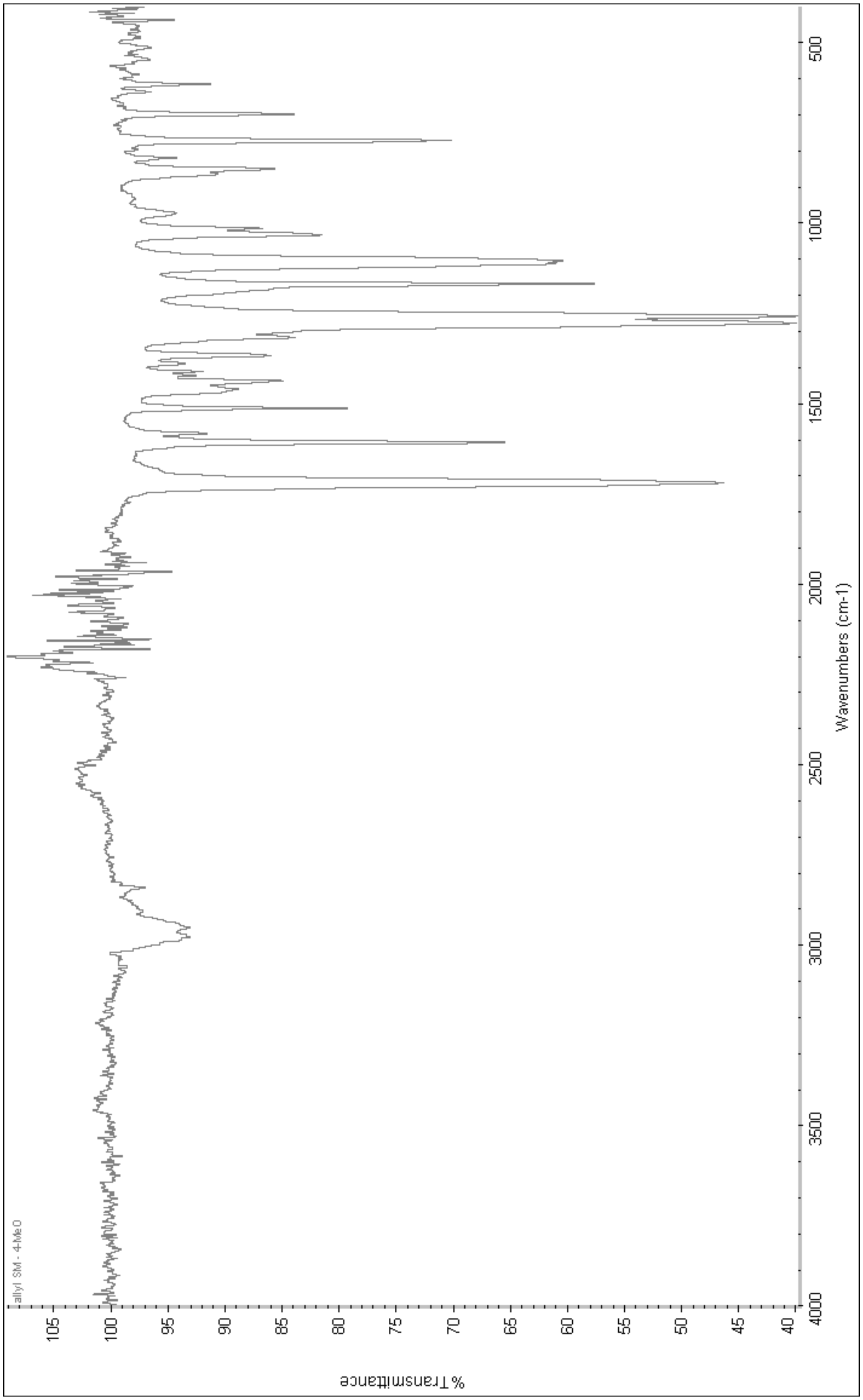
allyl SM - 4-MeO
%Transmittance
Wavenumbers (cm-1)
105
100
95
90
85
80
75
70
65
60
55
50
45
40
4000
3500
3000
2500
2000
1500
1000
500

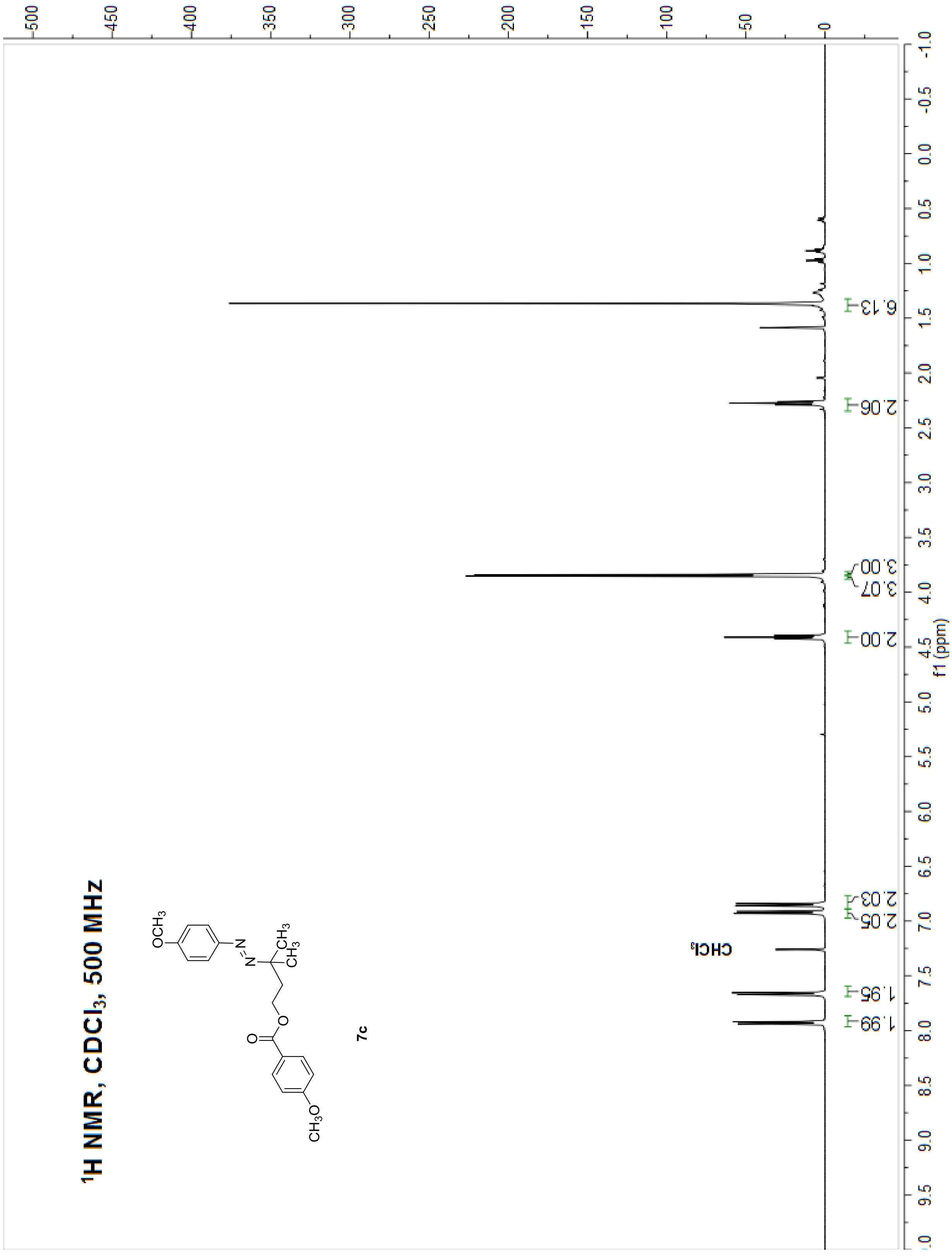

1H NMR, CDCl3, 500 MHz
7c
CHCl3
f1 (ppm)

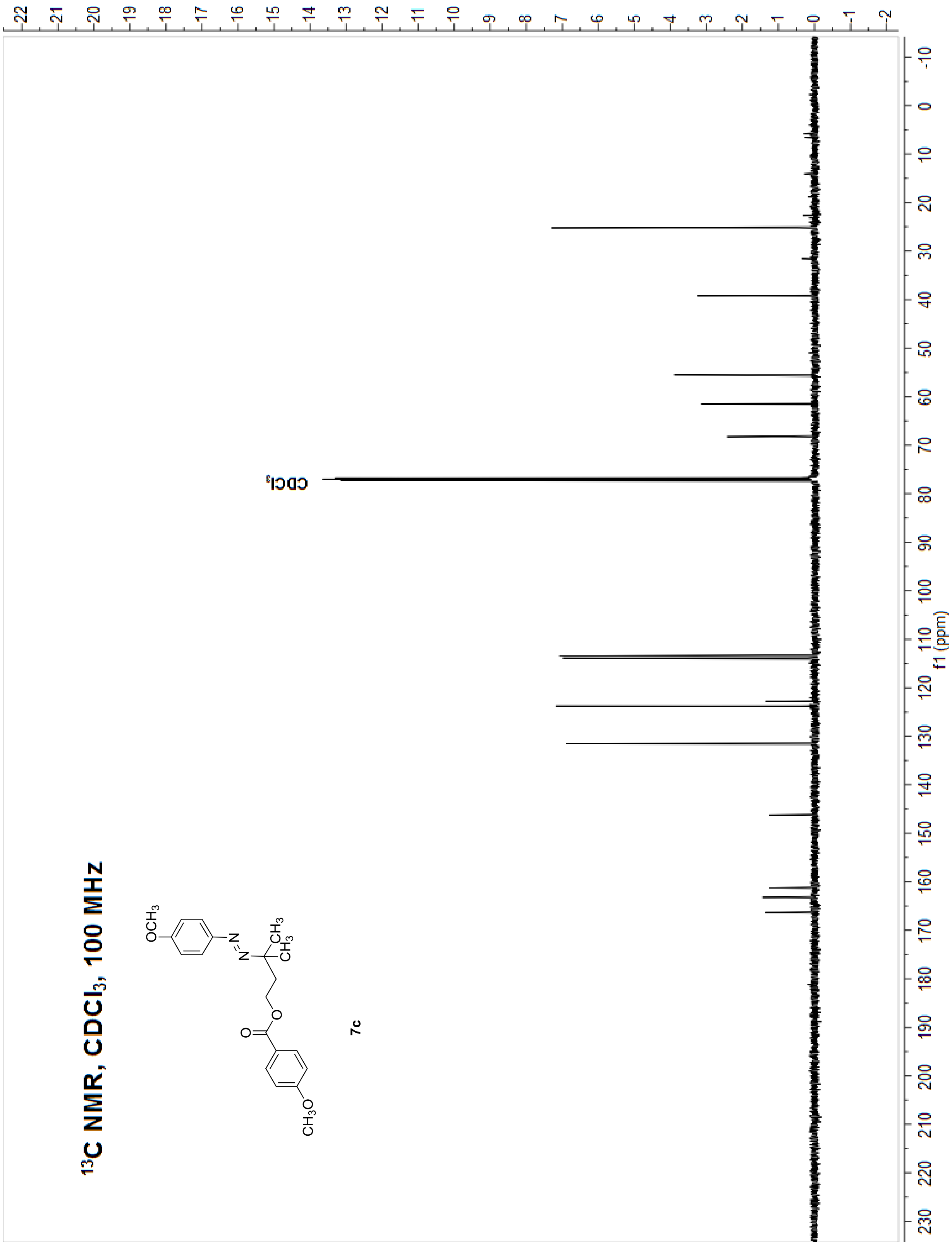

13C NMR, CDCl3, 100 MHz
7c
CDCl3
f1 (ppm)

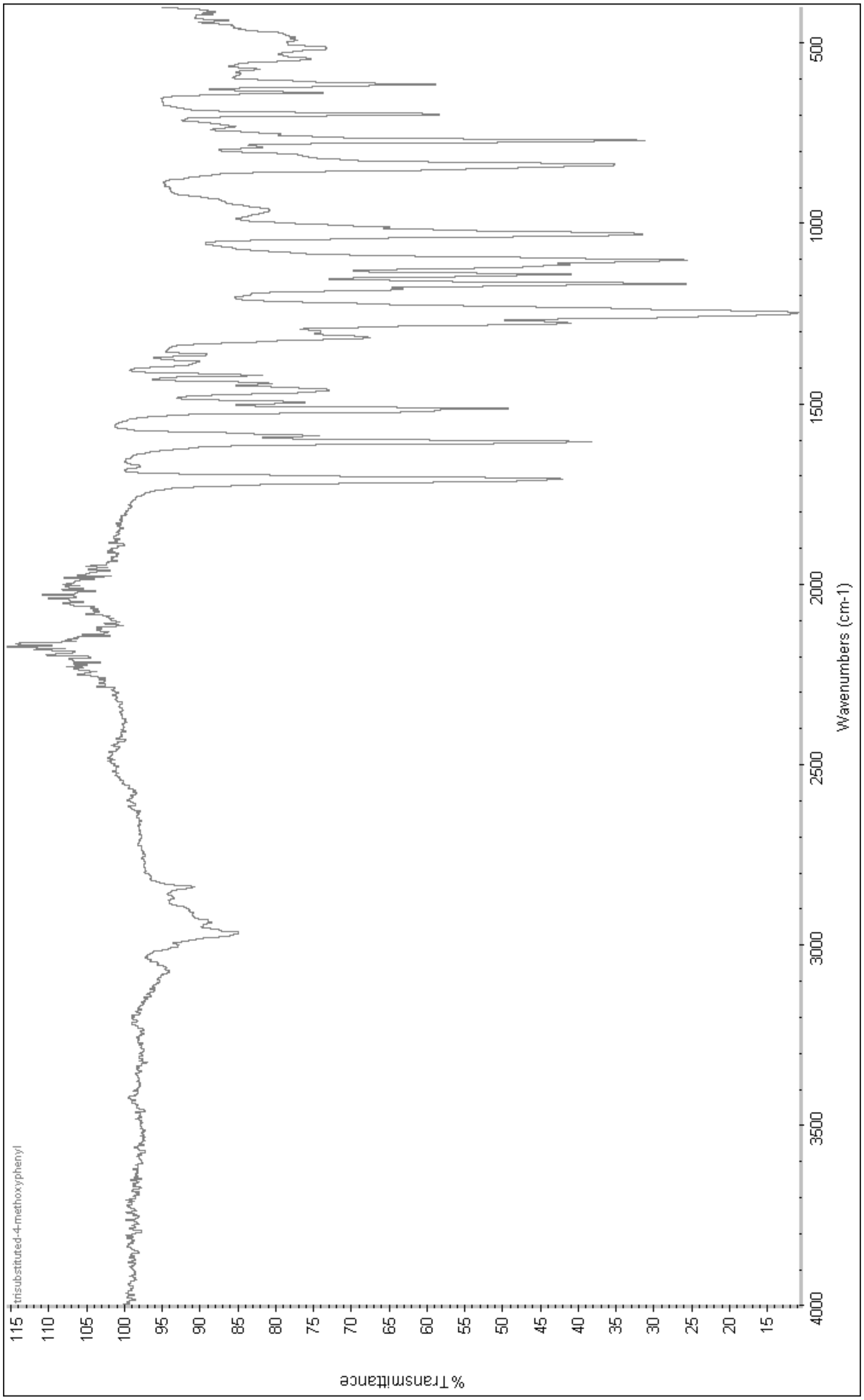
trisubstituted-4-methoxyphenyl
%Transmittance
Wavenumbers (cm-1)

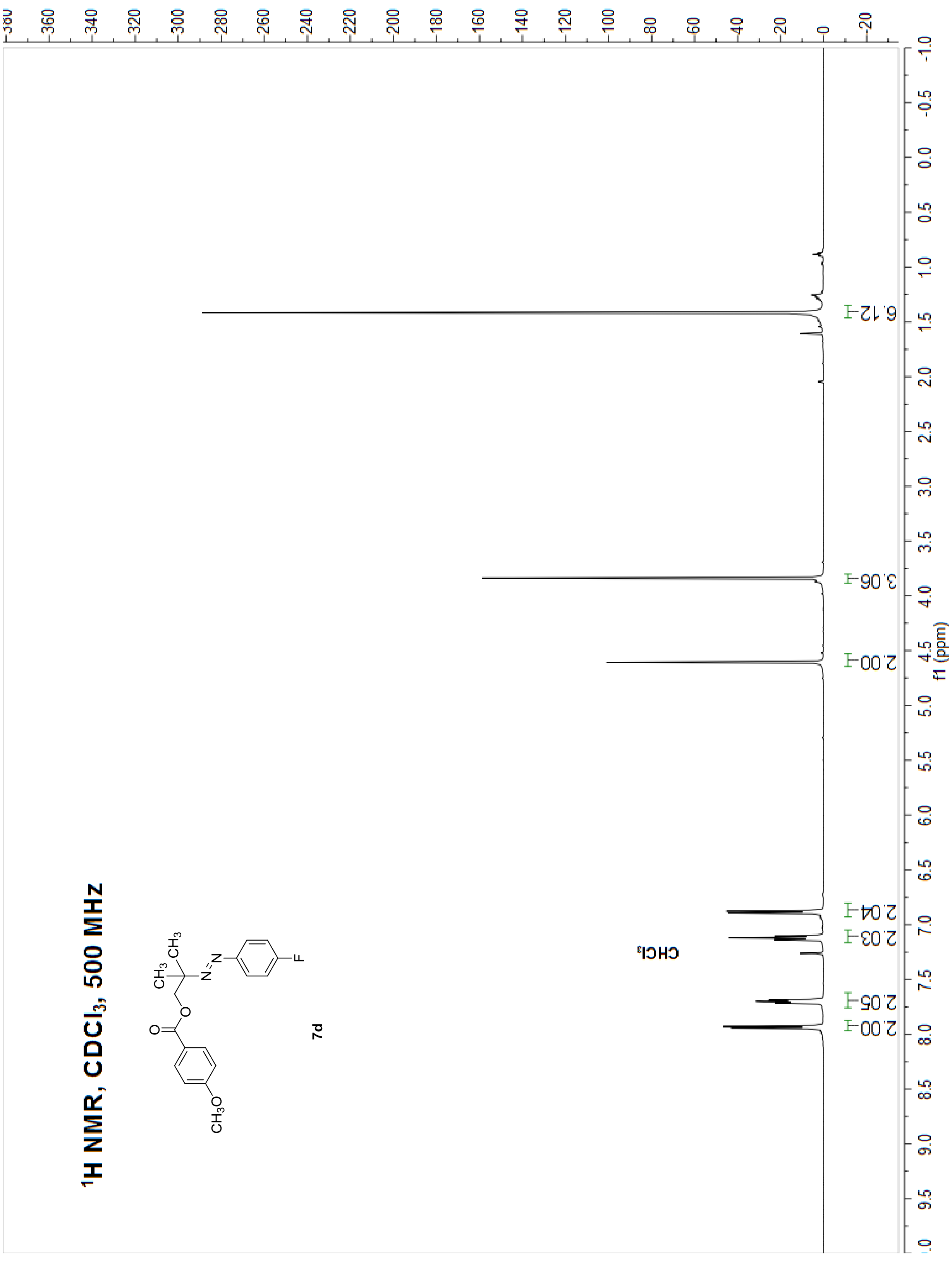

1H NMR, CDCl3, 500 MHz
7d
CHCl3
6.12
3.06
2.00
2.04
2.03
2.05
2.00
f1 (ppm)

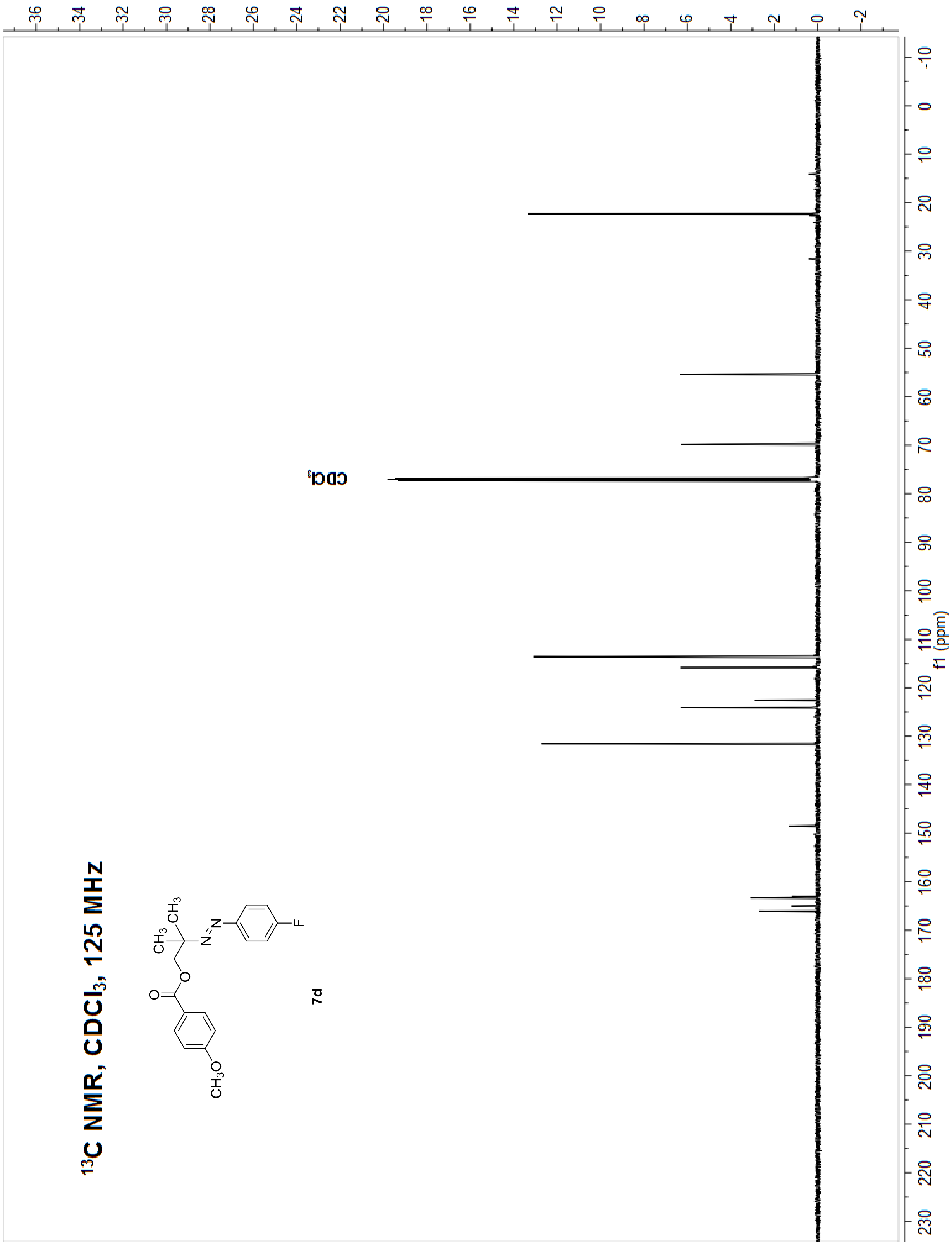

13C NMR, CDCl3, 125 MHz
7d
CDCl3
f1 (ppm)

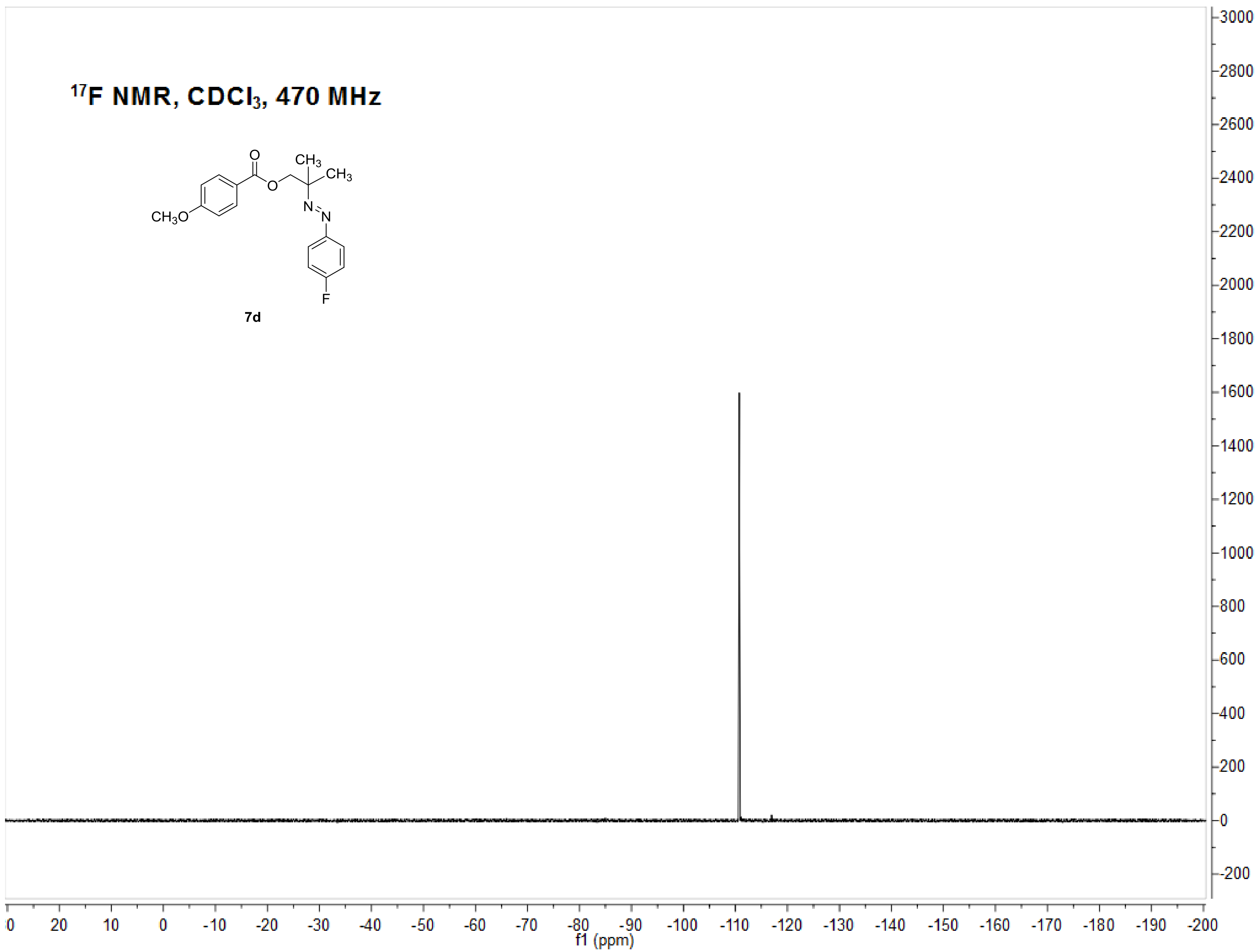

^{17}F NMR, $CDCl_3$, 470 MHz
CH_3O
O
O
CH_3
CH_3
N
N
F
7d
f1 (ppm)

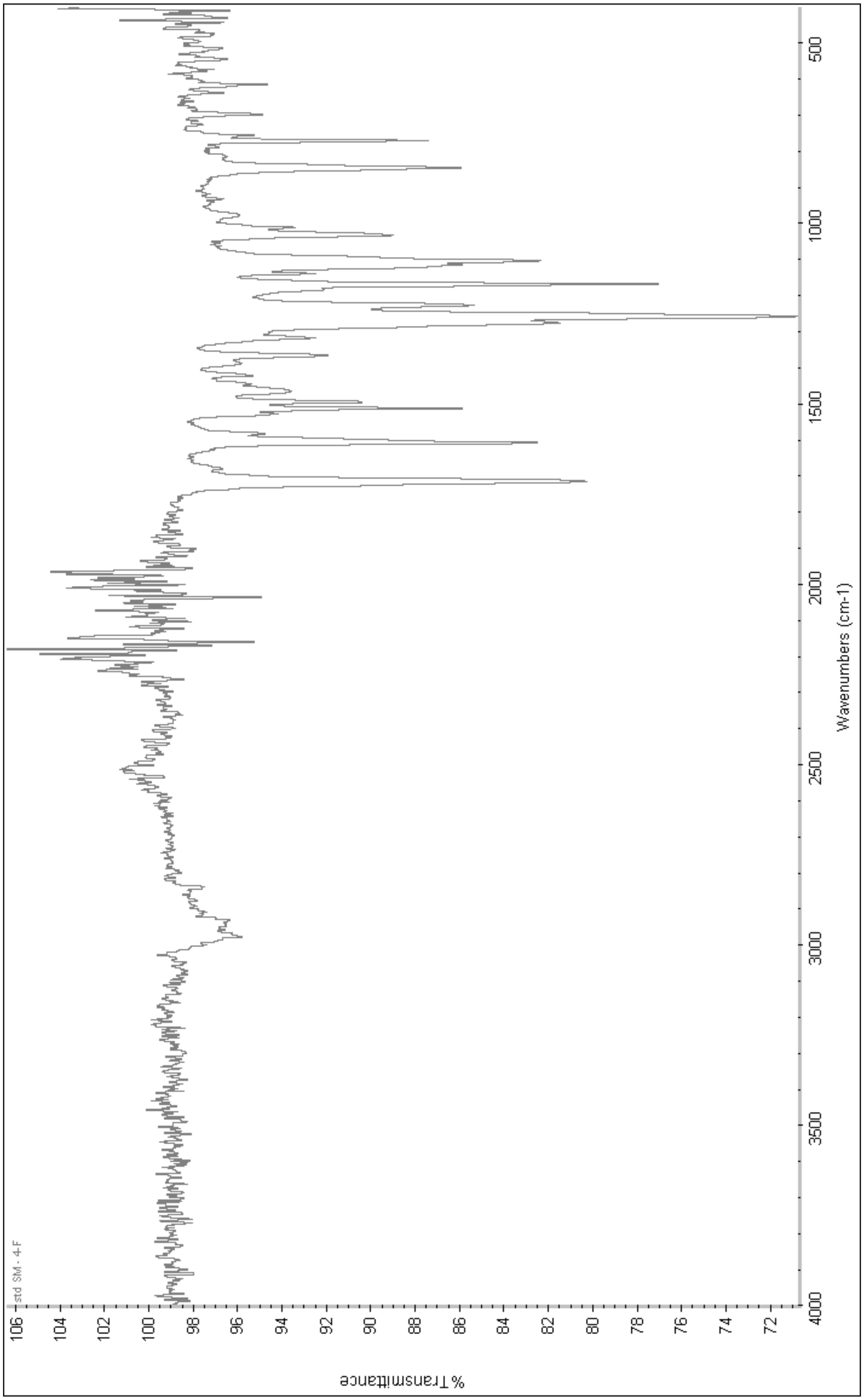
std SM - 4-F
%Transmittance
Wavenumbers (cm-1)

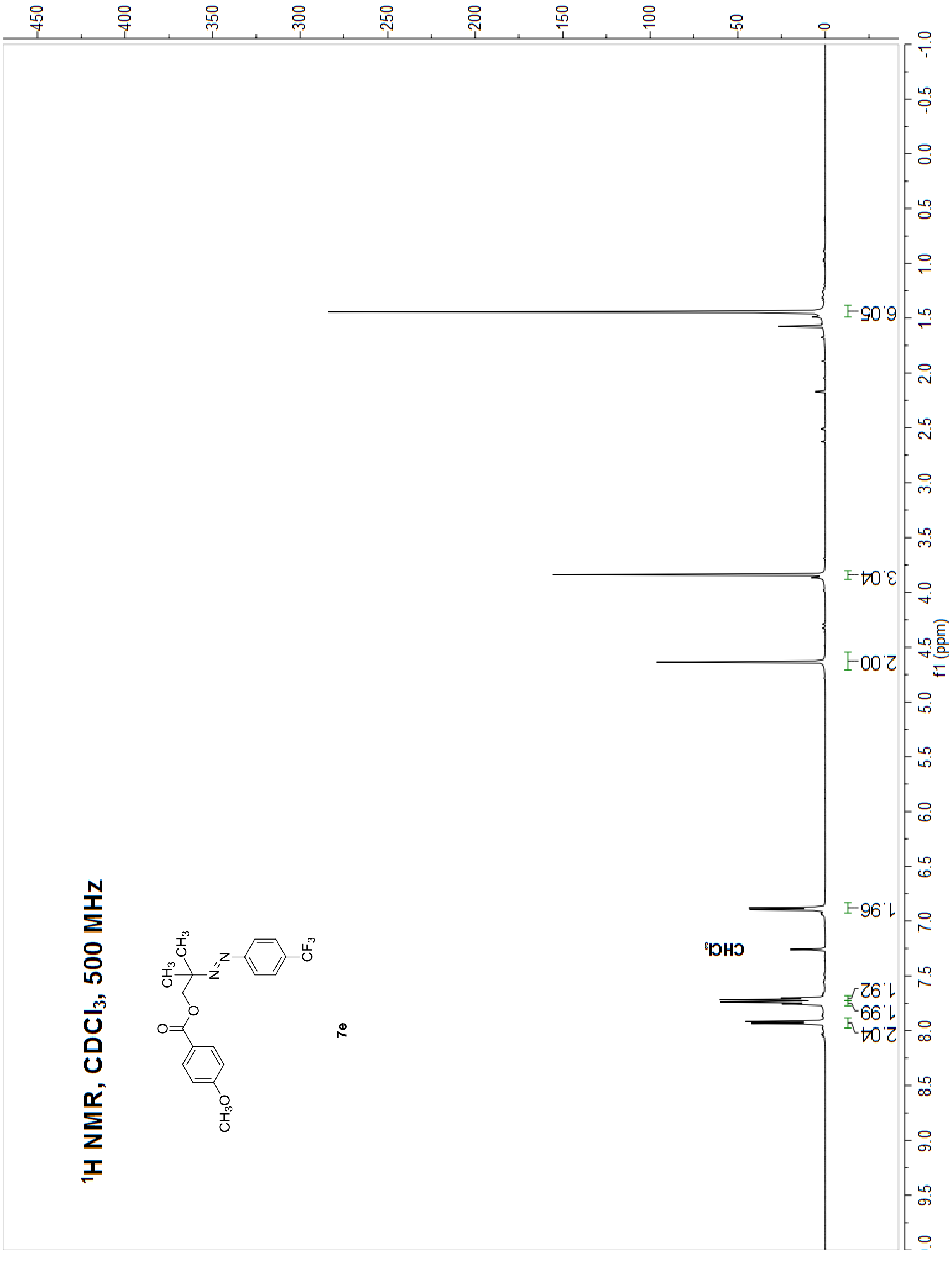

1H NMR, CDCl3, 500 MHz
7e
CHCl3
f1 (ppm)

^{13}C NMR, $CDCl_3$, 125 MHz

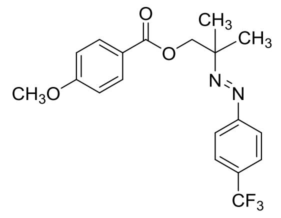

7e

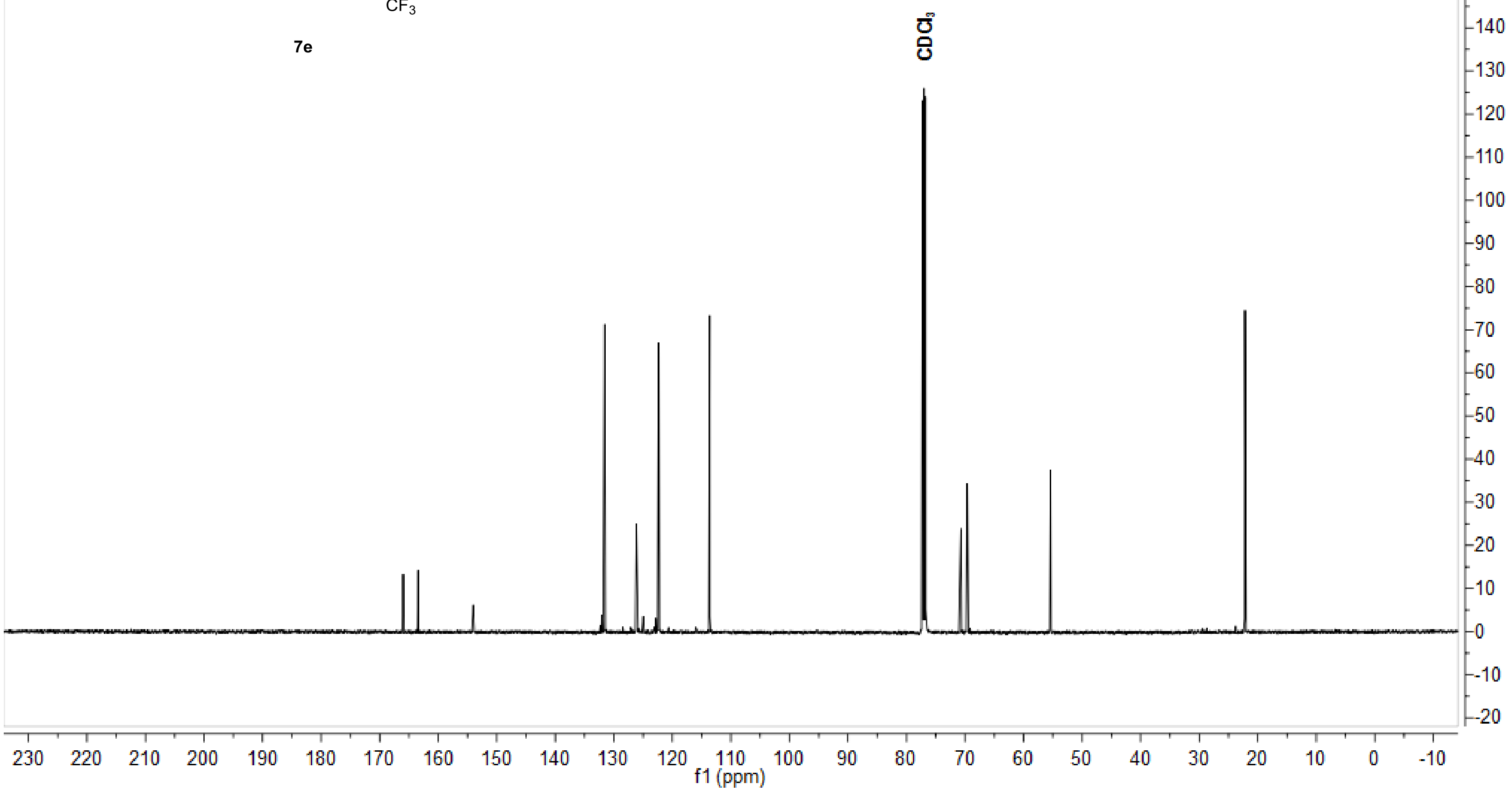

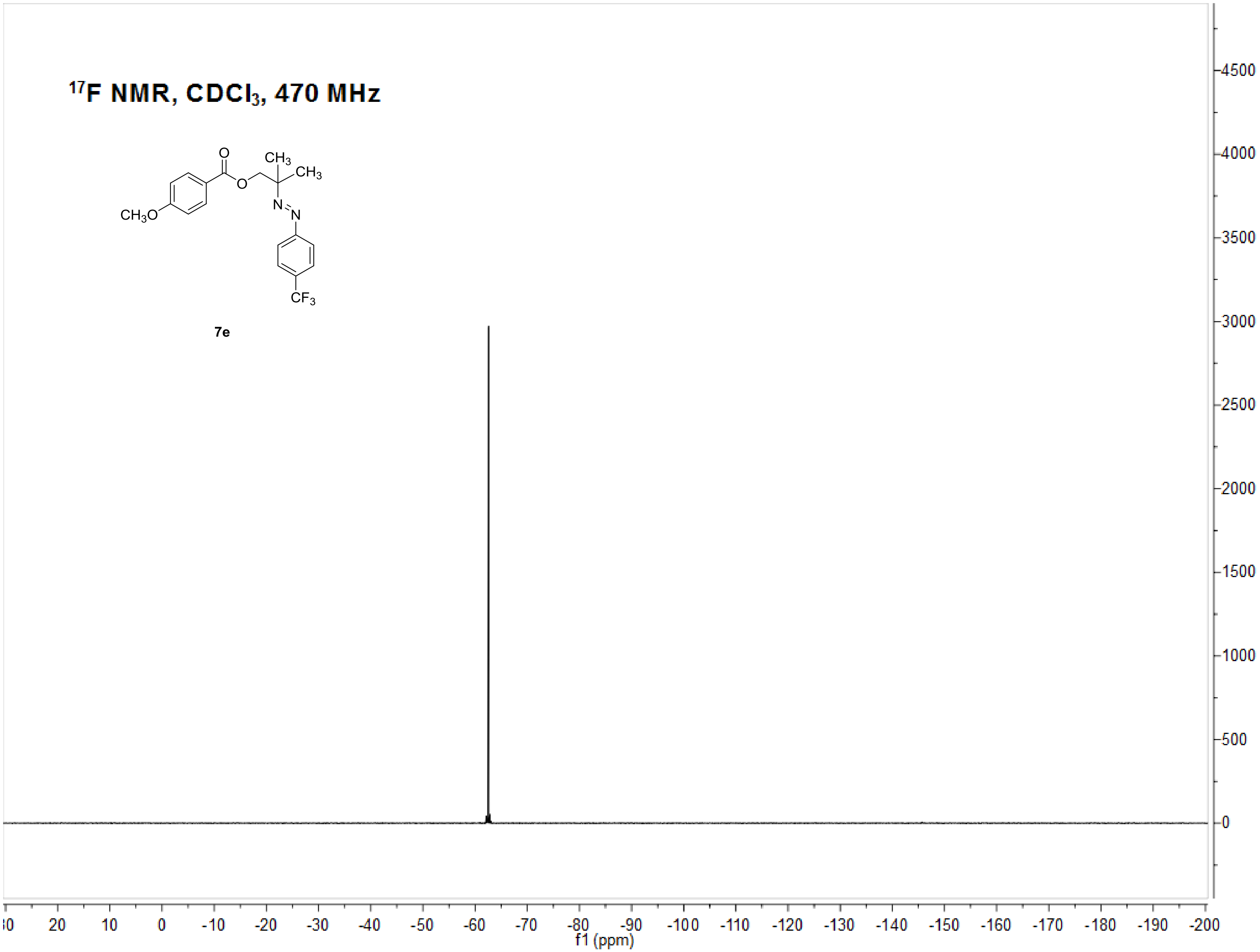

^{17}F NMR, $CDCl_3$, 470 MHz
CH3O
O
O
CH3
CH3
N
N
CF3
7e
4500
4000
3500
3000
2500
2000
1500
1000
500
0
20
10
0
-10
-20
-30
-40
-50
-60
-70
-80
-90
-100
-110
-120
-130
-140
-150
-160
-170
-180
-190
-200
f1 (ppm)

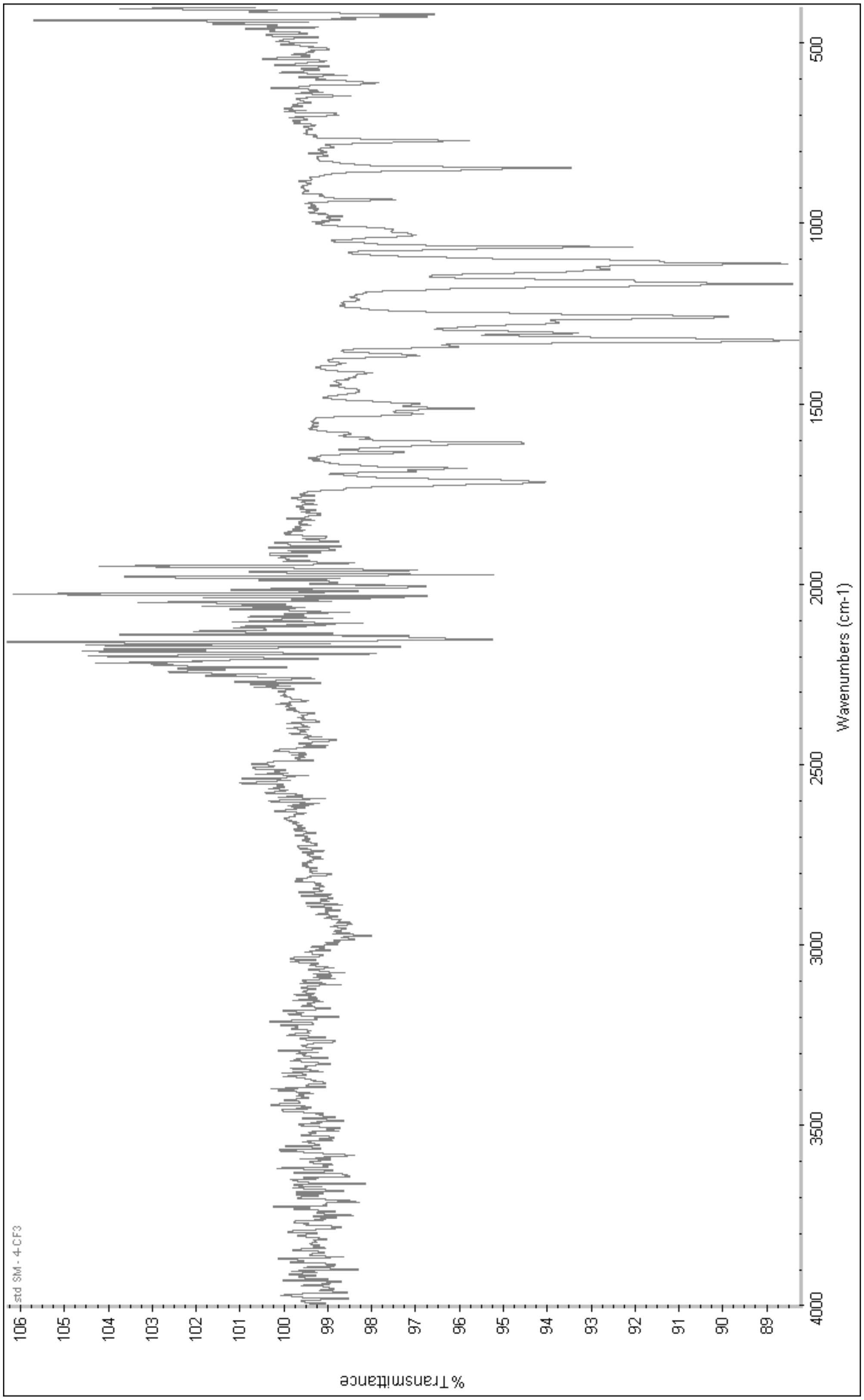
std SM - 4-CF3
%Transmittance
Wavenumbers (cm-1)

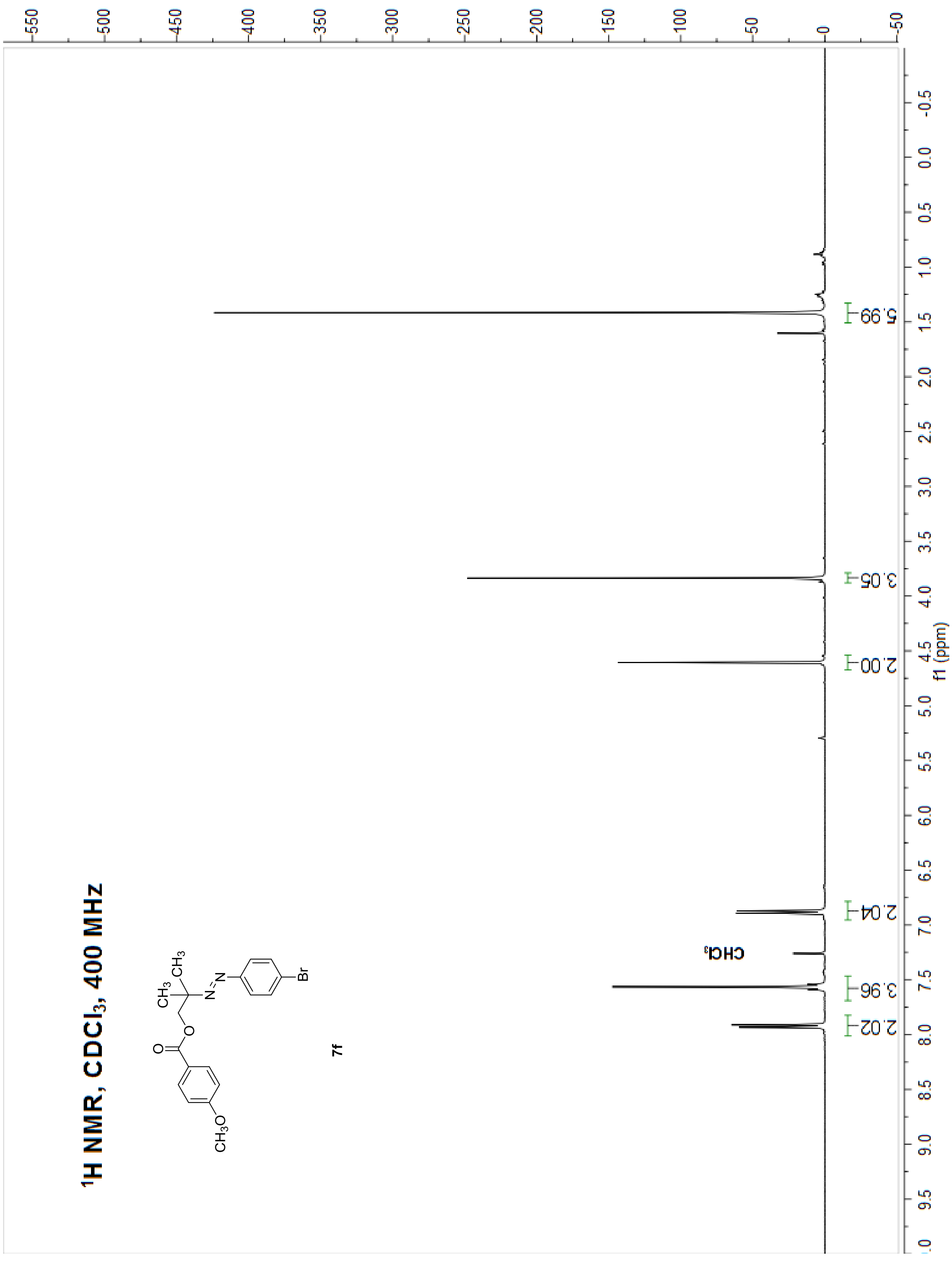

^{1}H NMR, CDCl$_3$, 400 MHz
7f
CHCl$_3$
5.99
3.05
2.00
2.04
3.96
2.02
f1 (ppm)

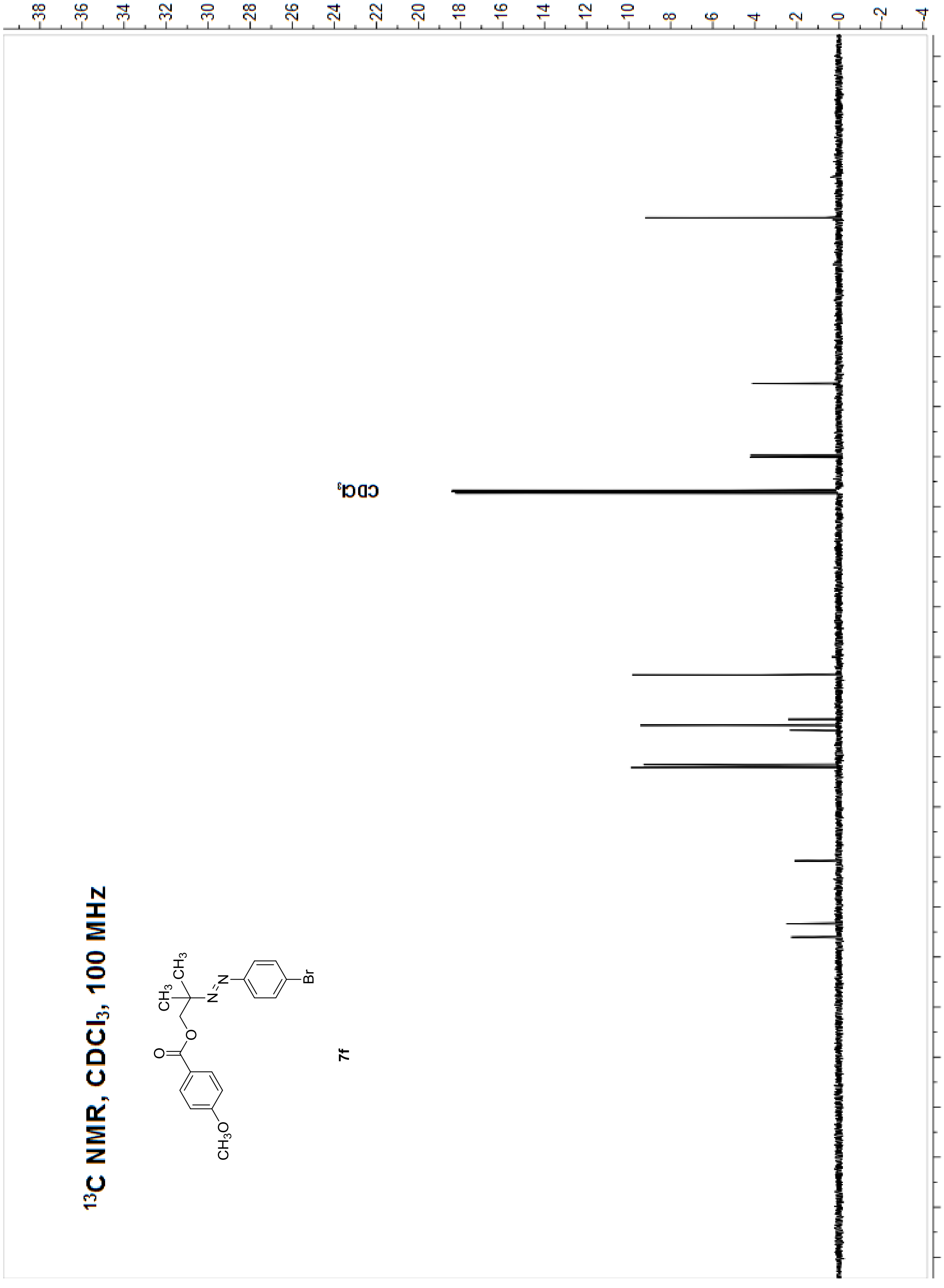

13C NMR, CDCl3, 100 MHz
7f
CDCl3
f1 (ppm)

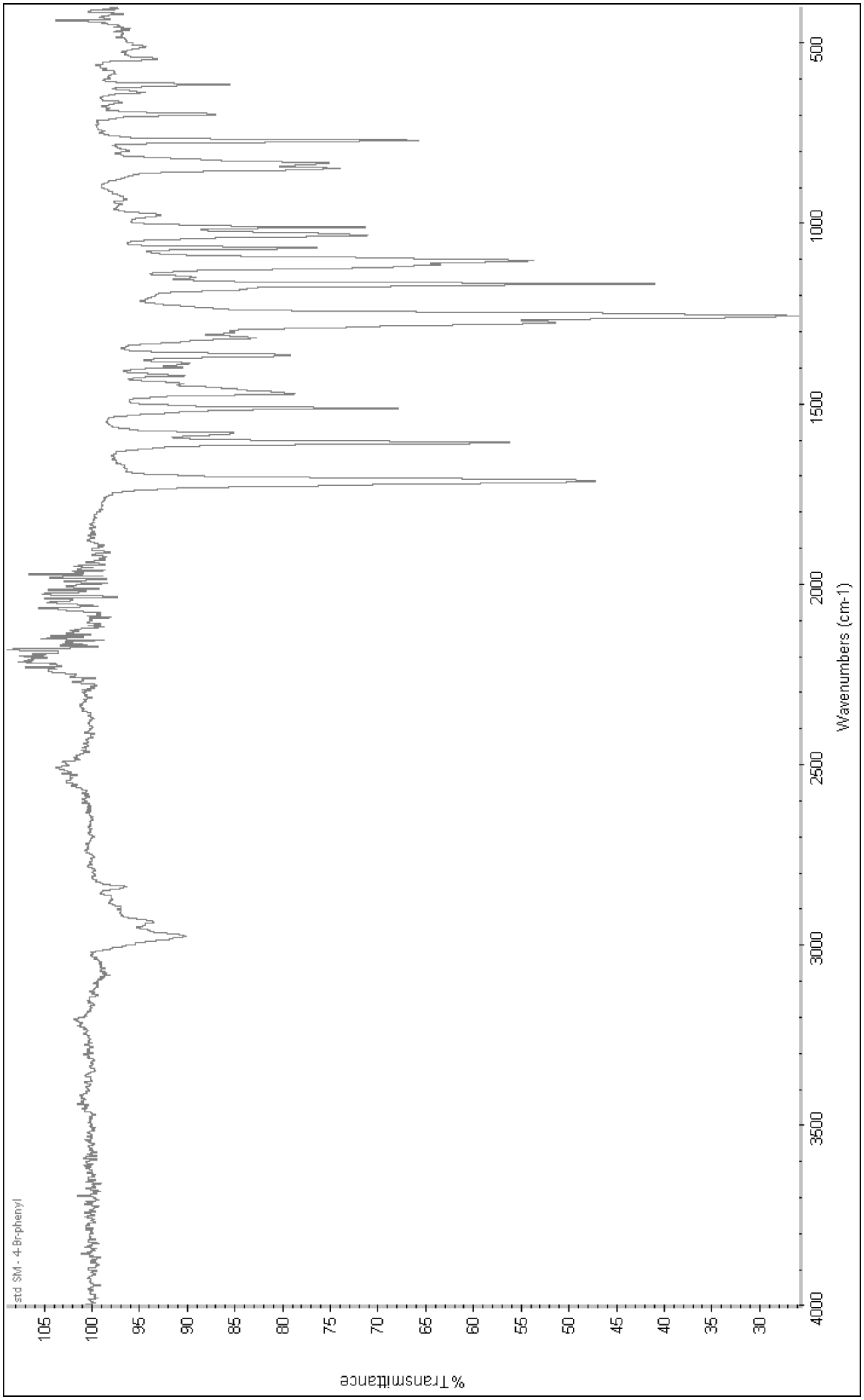
std SM - 4-Br-phenyl
%Transmittance
Wavenumbers (cm-1)
105
100
95
90
85
80
75
70
65
60
55
50
45
40
35
30
4000
3500
3000
2500
2000
1500
1000
500

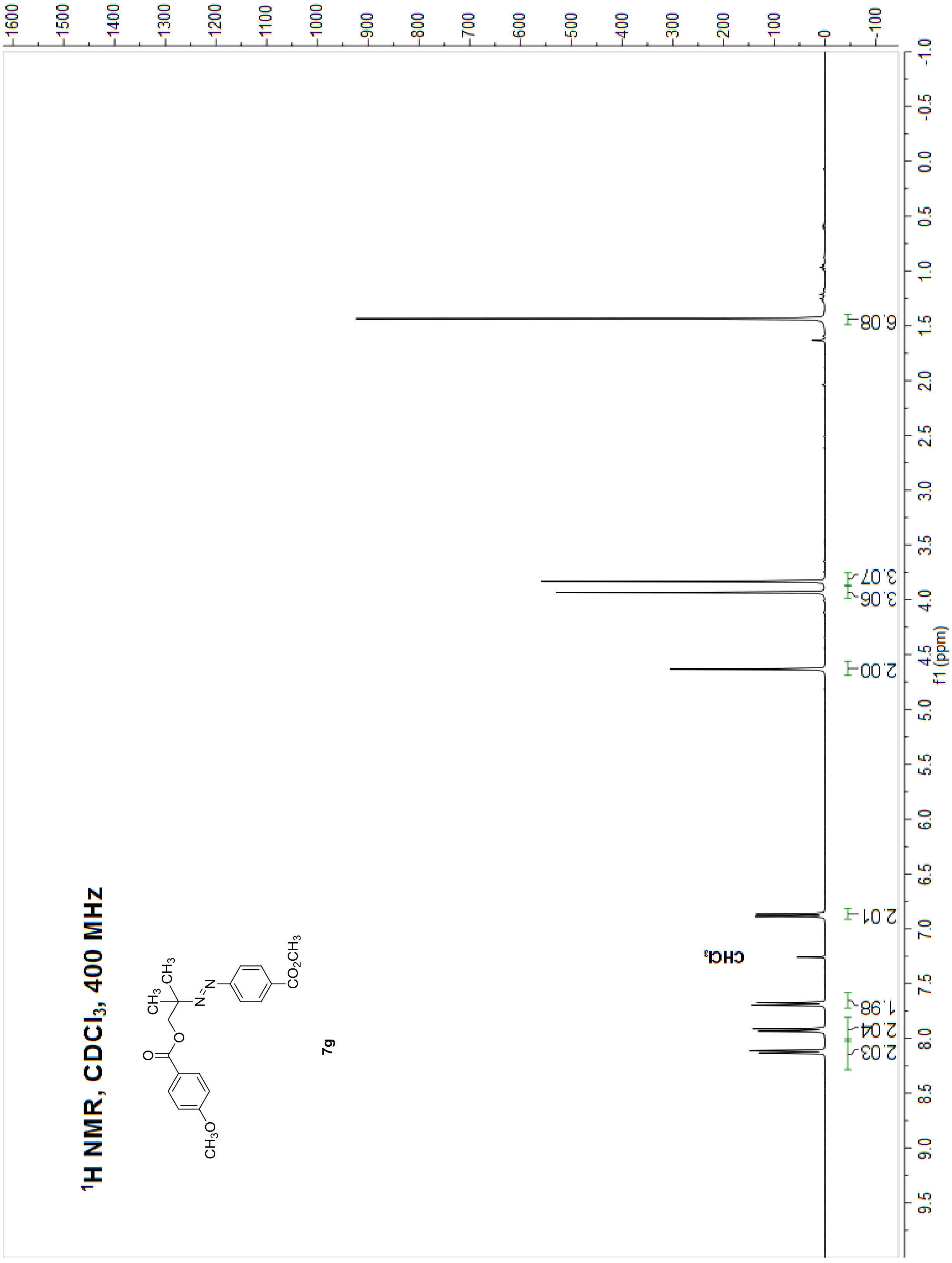

1H NMR, CDCl3, 400 MHz
7g
CHCl3
f1 (ppm)

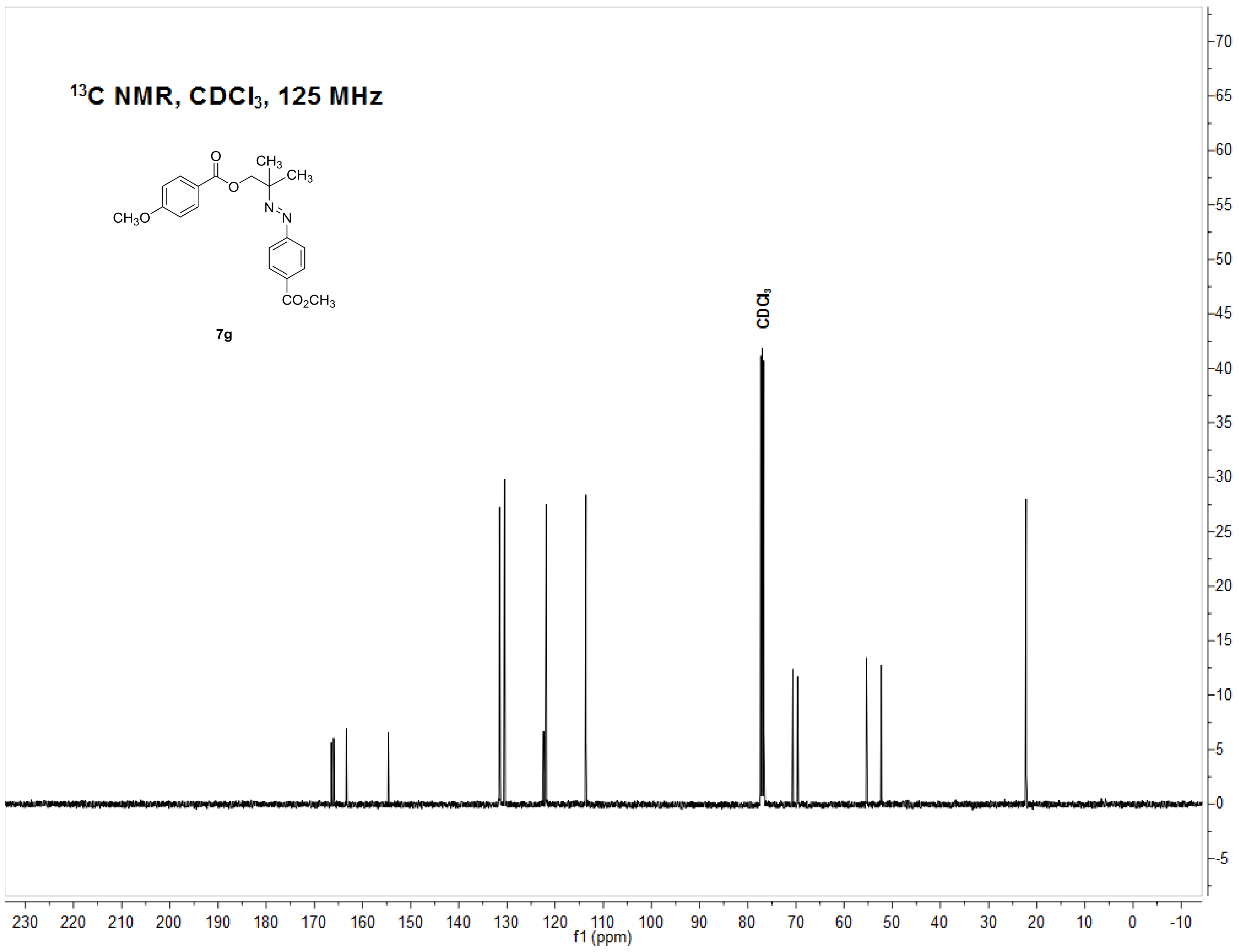

^{13}C NMR, $CDCl_3$, 125 MHz
CH3O
O
O
CH3
CH3
N
N
CO2CH3
7g
CDCl3
f1 (ppm)

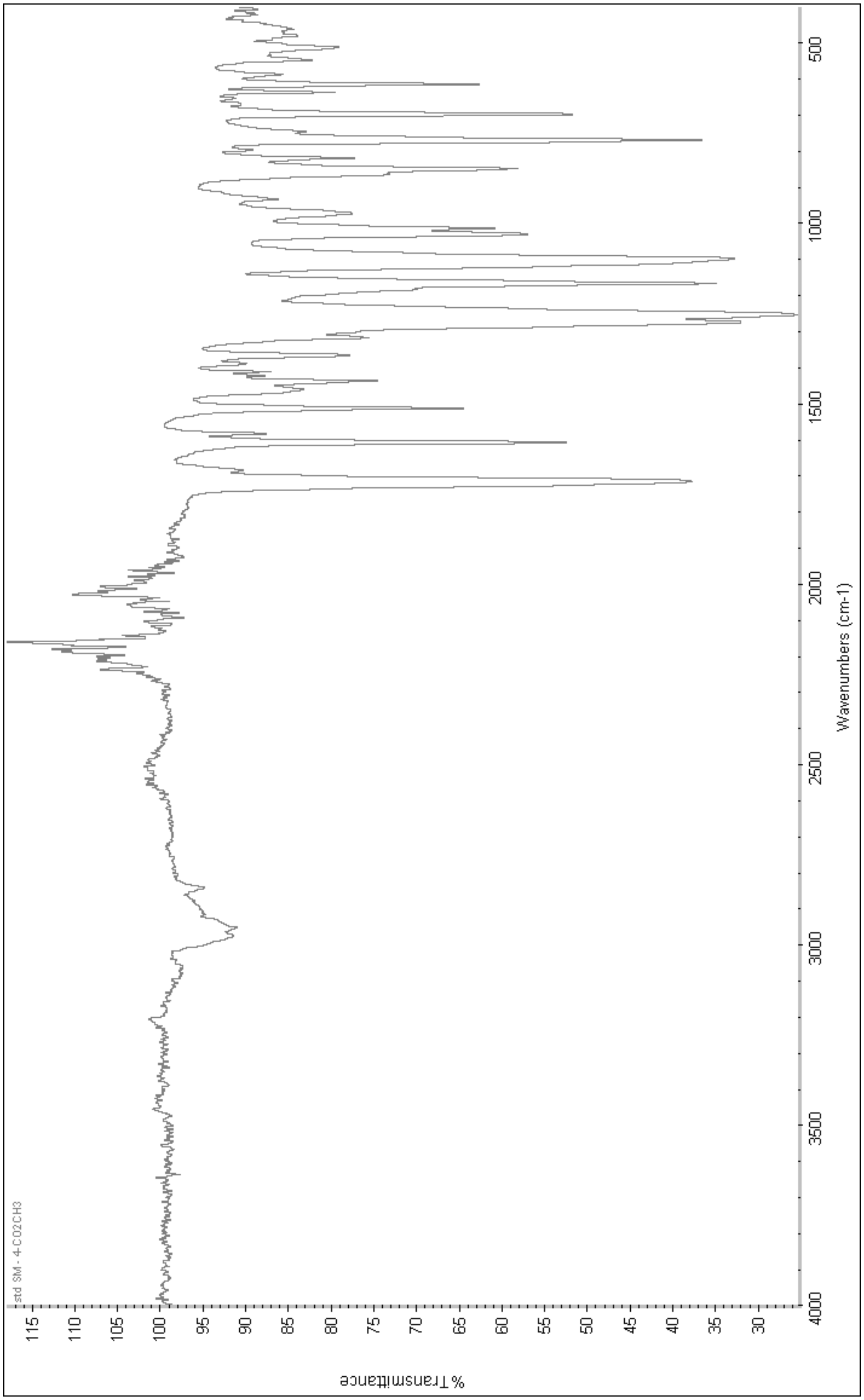

std SM - 4-CO2CH3
%Transmittance
Wavenumbers (cm-1)
115
110
105
100
95
90
85
80
75
70
65
60
55
50
45
40
35
30
4000
3500
3000
2500
2000
1500
1000
500

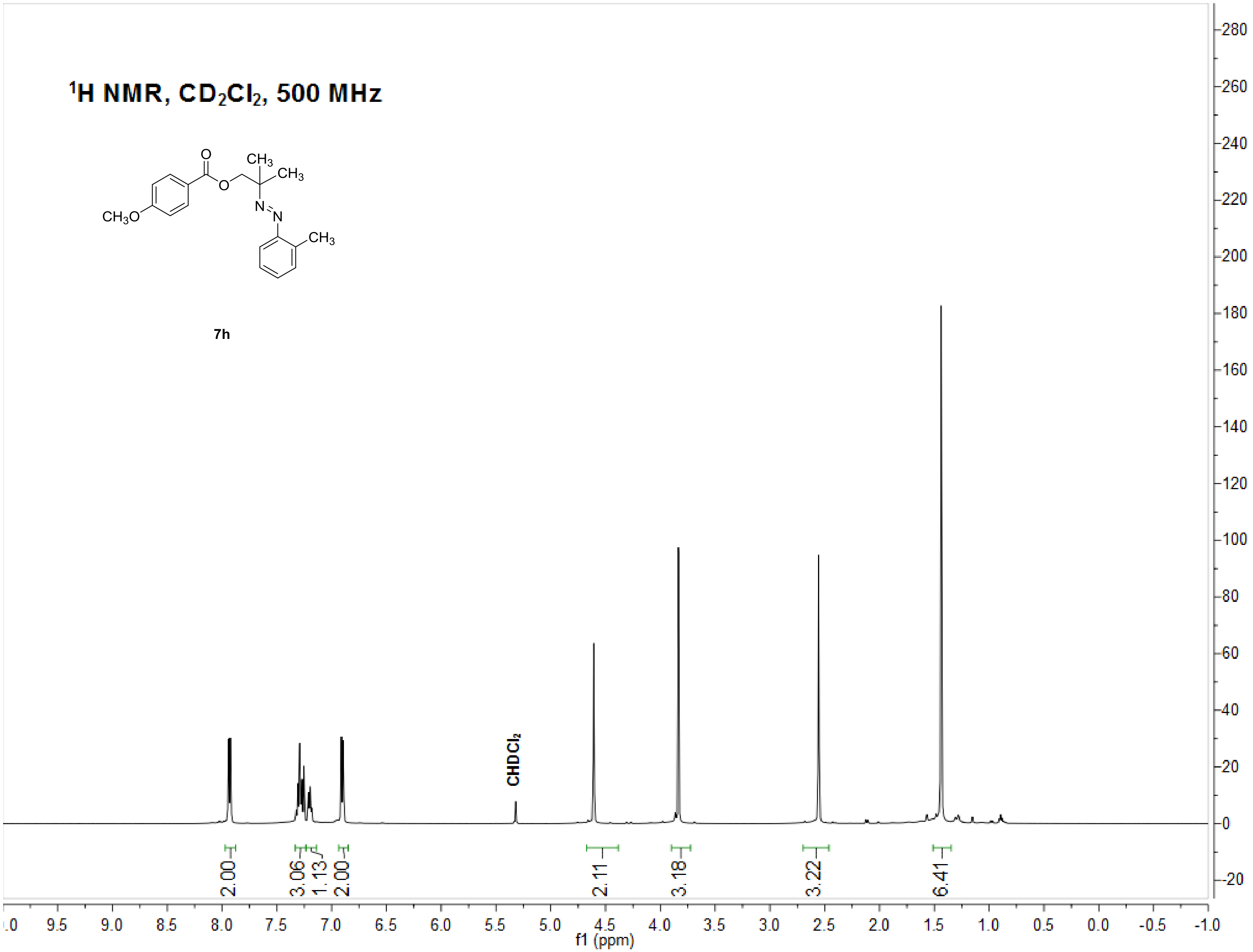

¹H NMR, CD₂Cl₂, 500 MHz
O
CH3
CH3
CH3O
O
N
N
CH3
CHDCl2
2.00
3.06
1.13
2.00
2.11
3.18
3.22
6.41
f1 (ppm)

7h

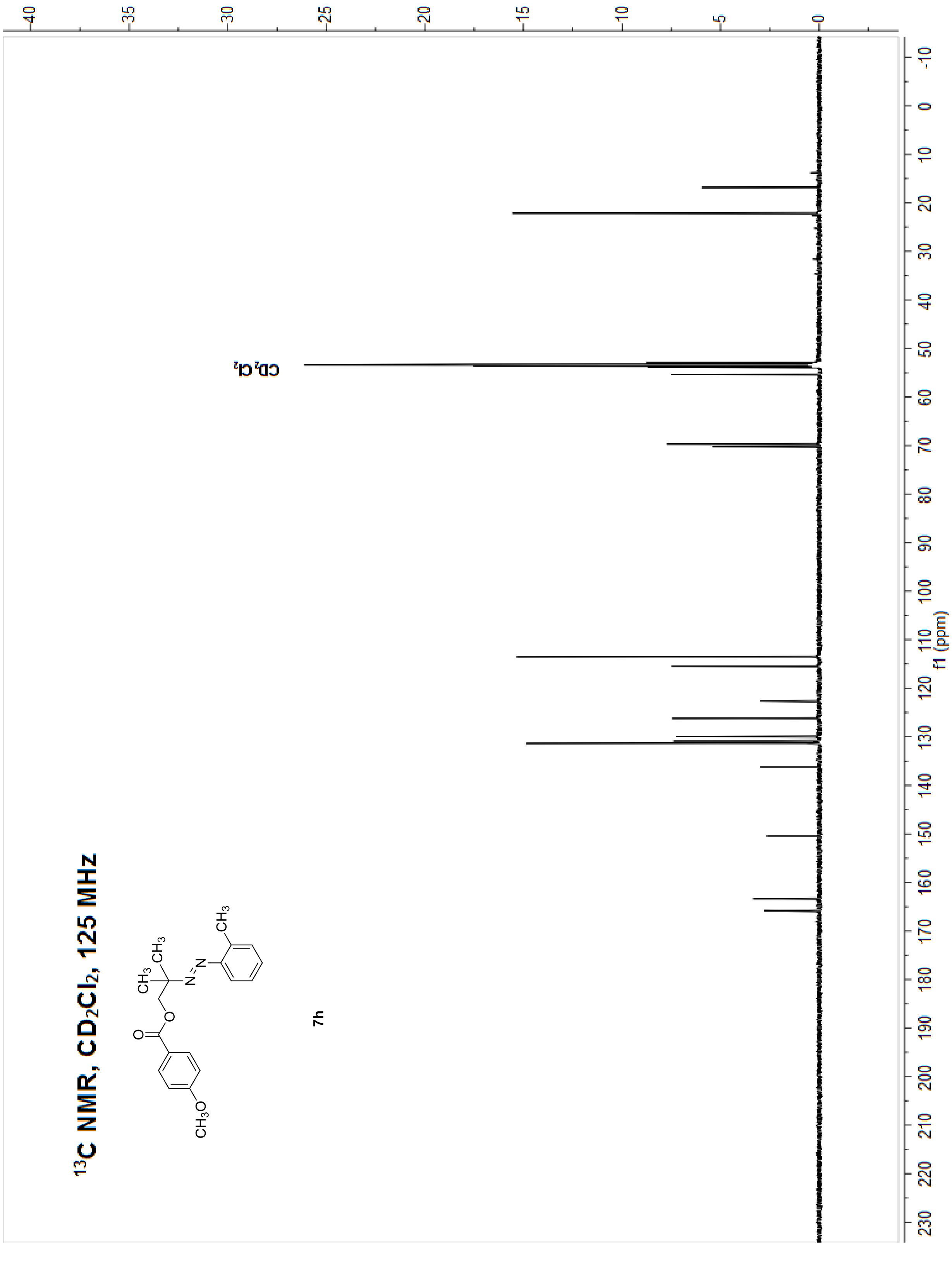

^{13}C NMR, CD_2Cl_2, 125 MHz
7h
CD_2Cl_2
f1 (ppm)

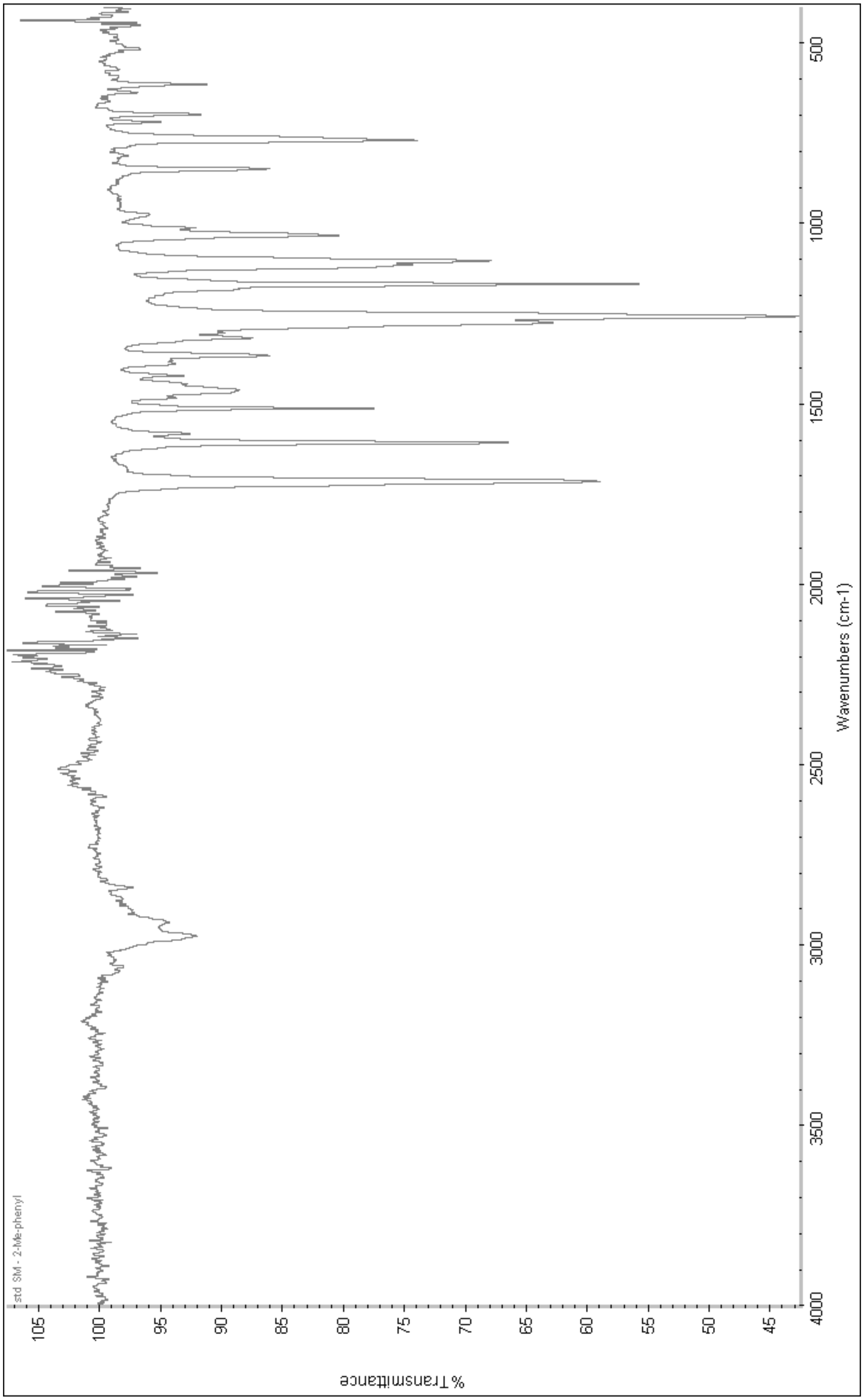
std SM - 2-Me-phenyl
%Transmittance
Wavenumbers (cm-1)
105
100
95
90
85
80
75
70
65
60
55
50
45
4000
3500
3000
2500
2000
1500
1000
500

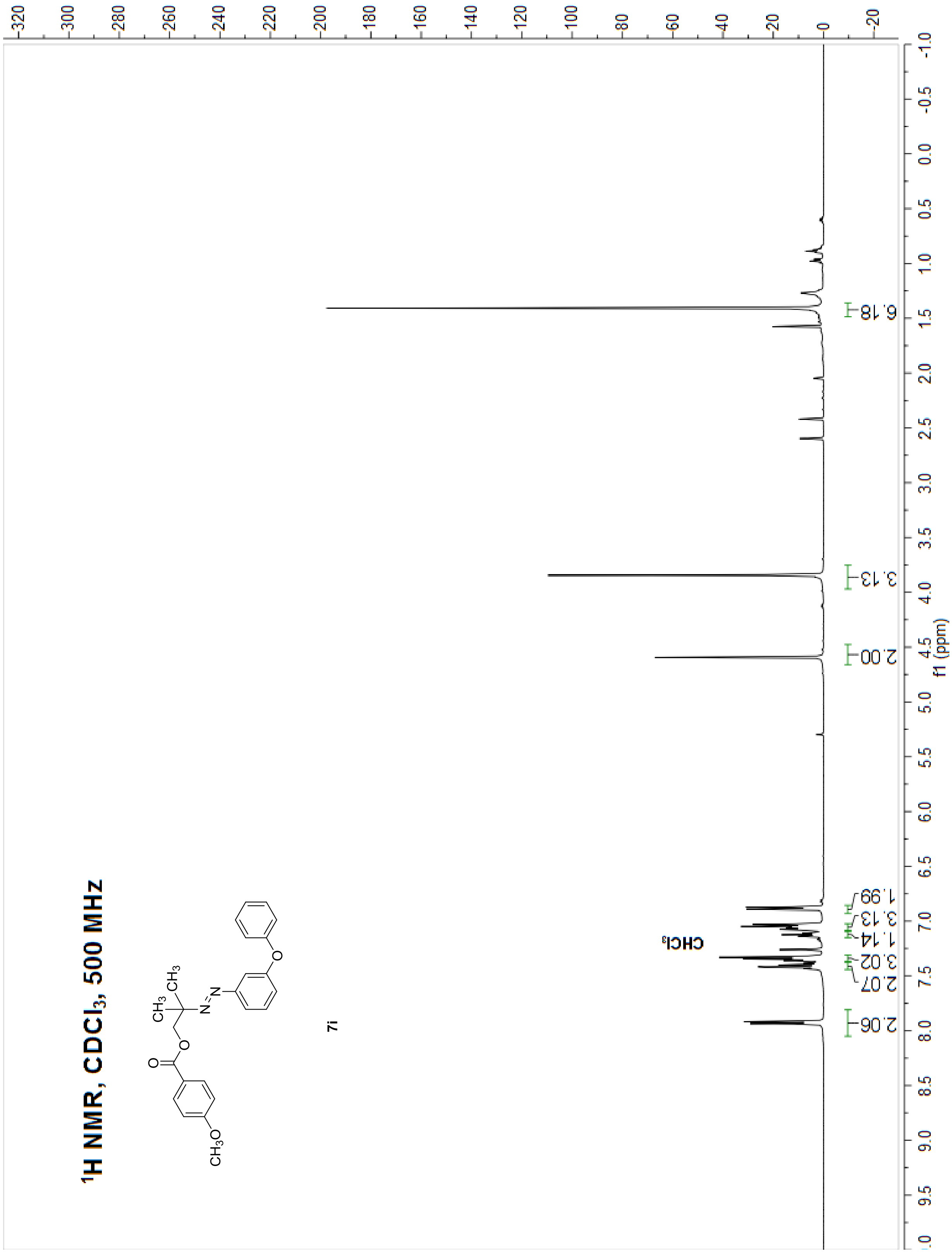

1H NMR, CDCl3, 500 MHz
7i
CHCl3
f1 (ppm)

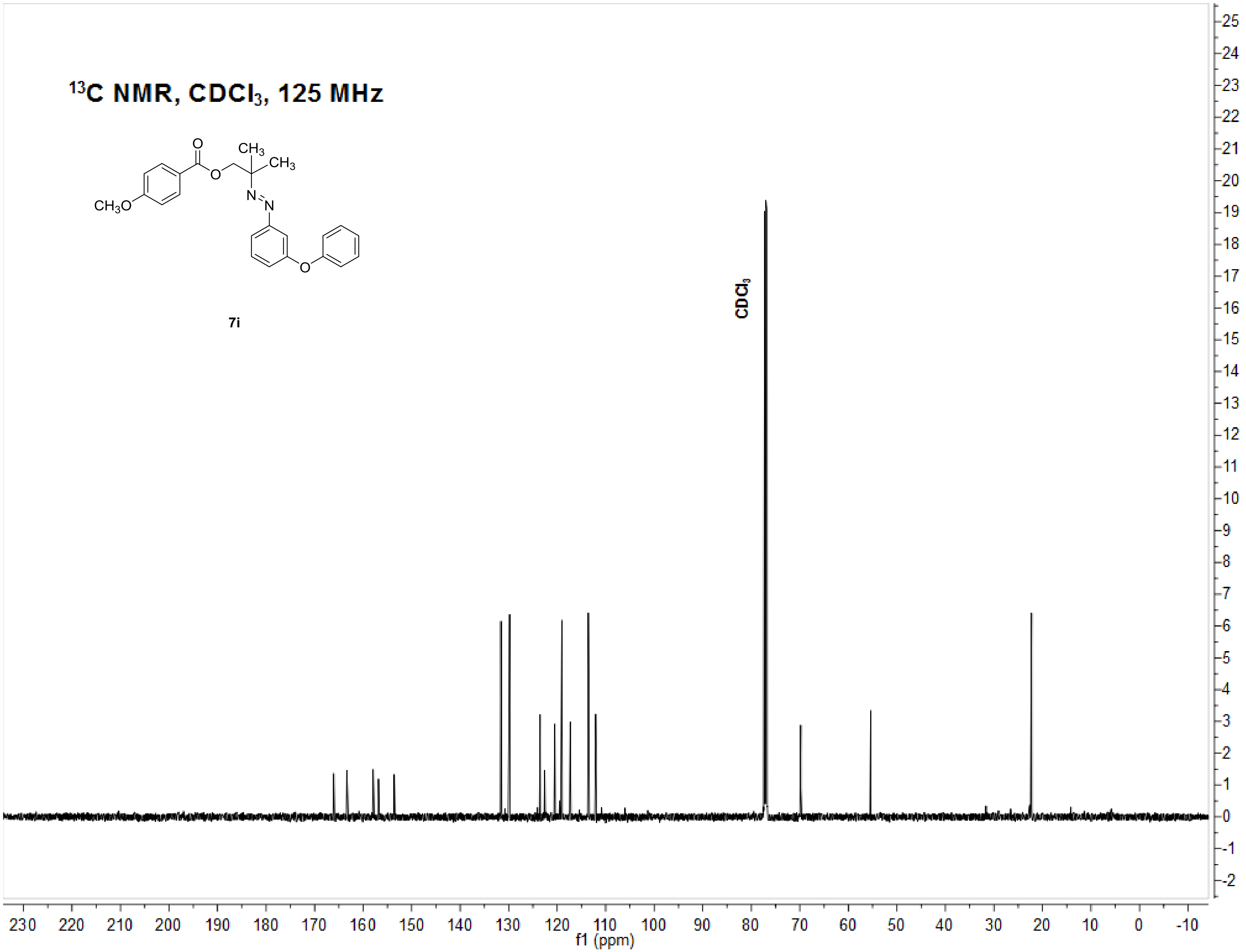

^{13}C NMR, $CDCl_3$, 125 MHz
CH3O
O
CH3
CH3
N
N
7i
CDCl3
f1 (ppm)

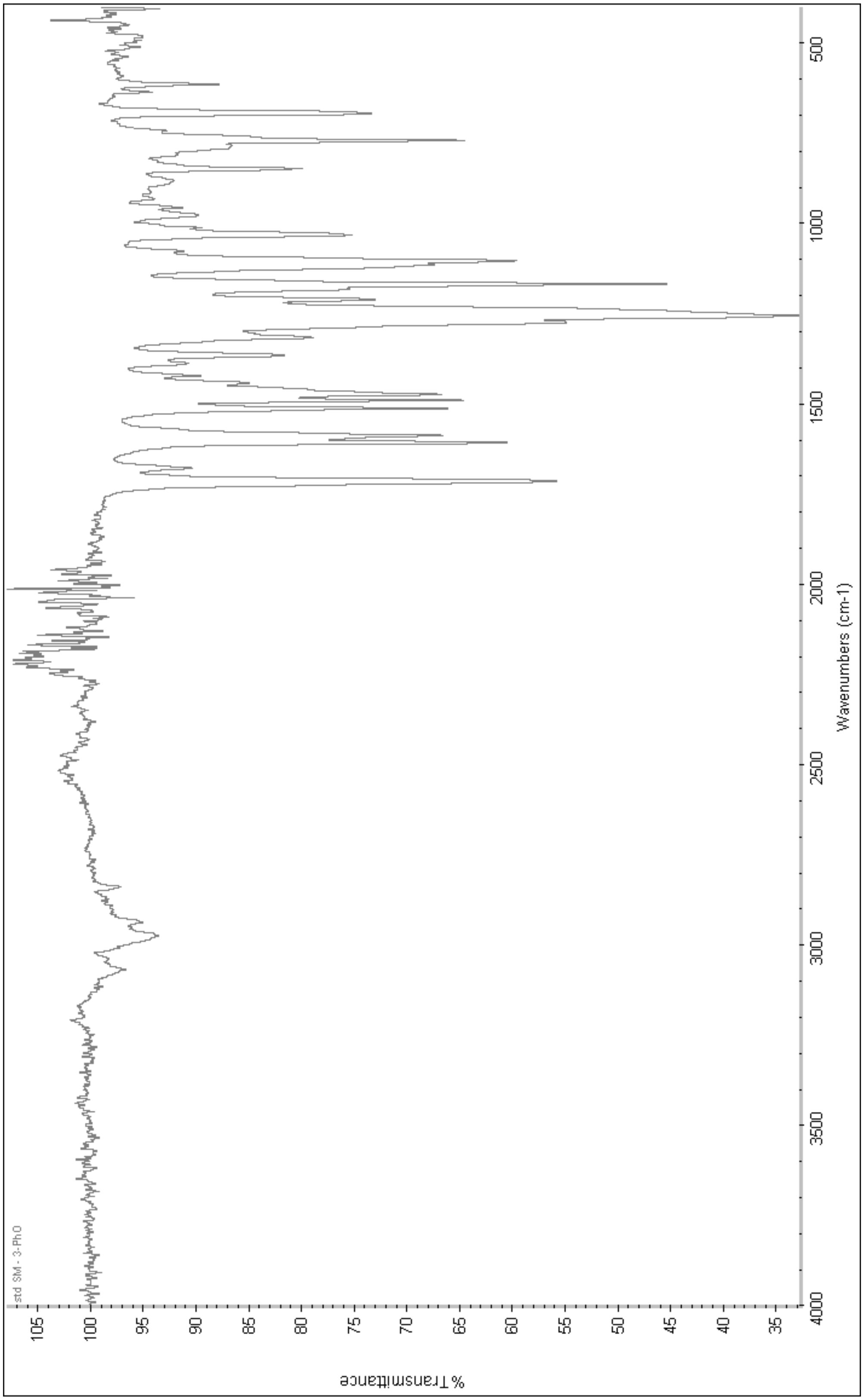
std SM - 3-PhO
%Transmittance
Wavenumbers (cm-1)
105
100
95
90
85
80
75
70
65
60
55
50
45
40
35
4000
3500
3000
2500
2000
1500
1000
500

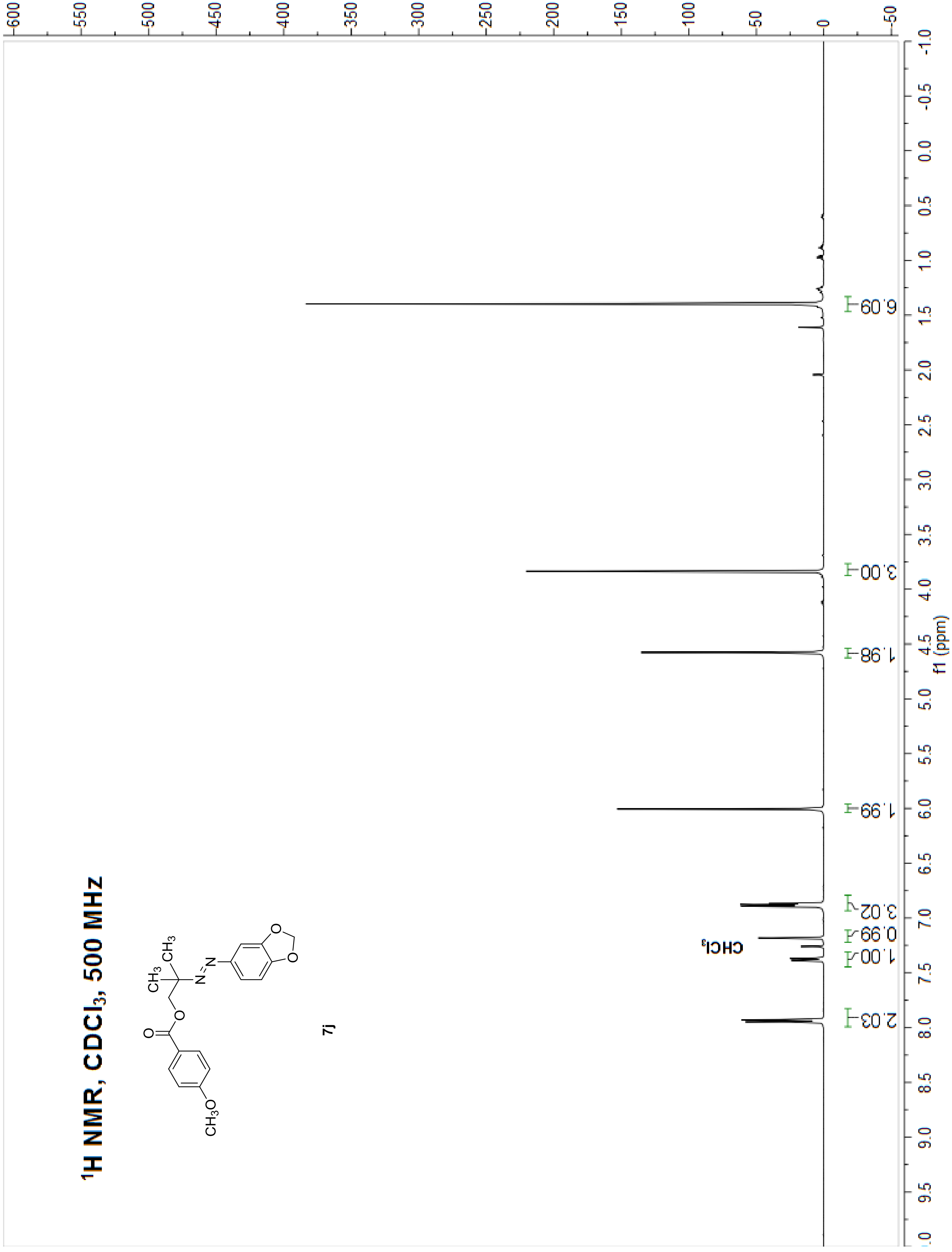

1H NMR, CDCl3, 500 MHz
7j
CHCl3
6.09
3.00
1.98
1.99
3.02
0.99
1.00
2.03
f1 (ppm)

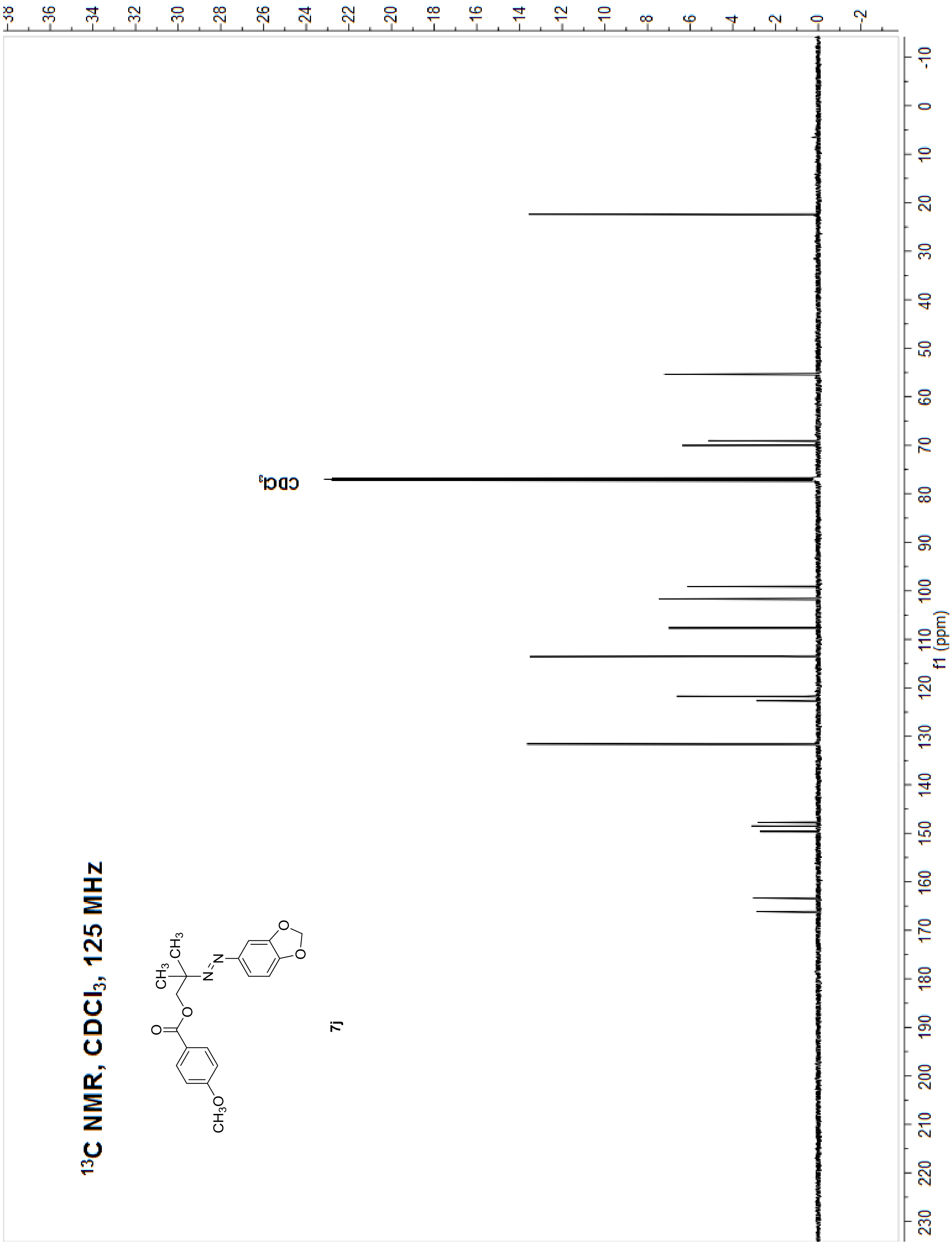

13C NMR, CDCl3, 125 MHz
7j
CDCl3
f1 (ppm)

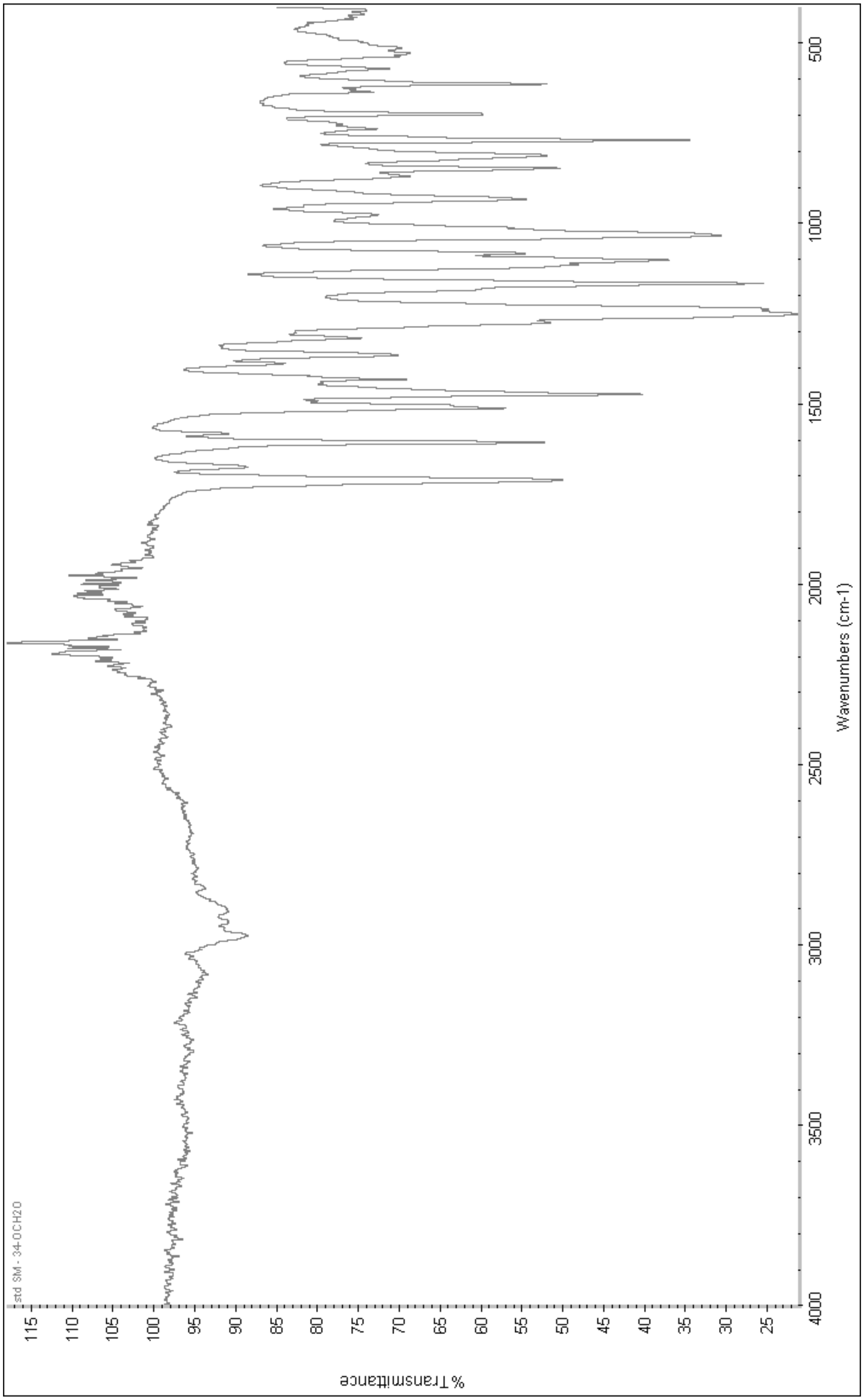
std SM - 34-OCH2O
%Transmittance
Wavenumbers (cm-1)

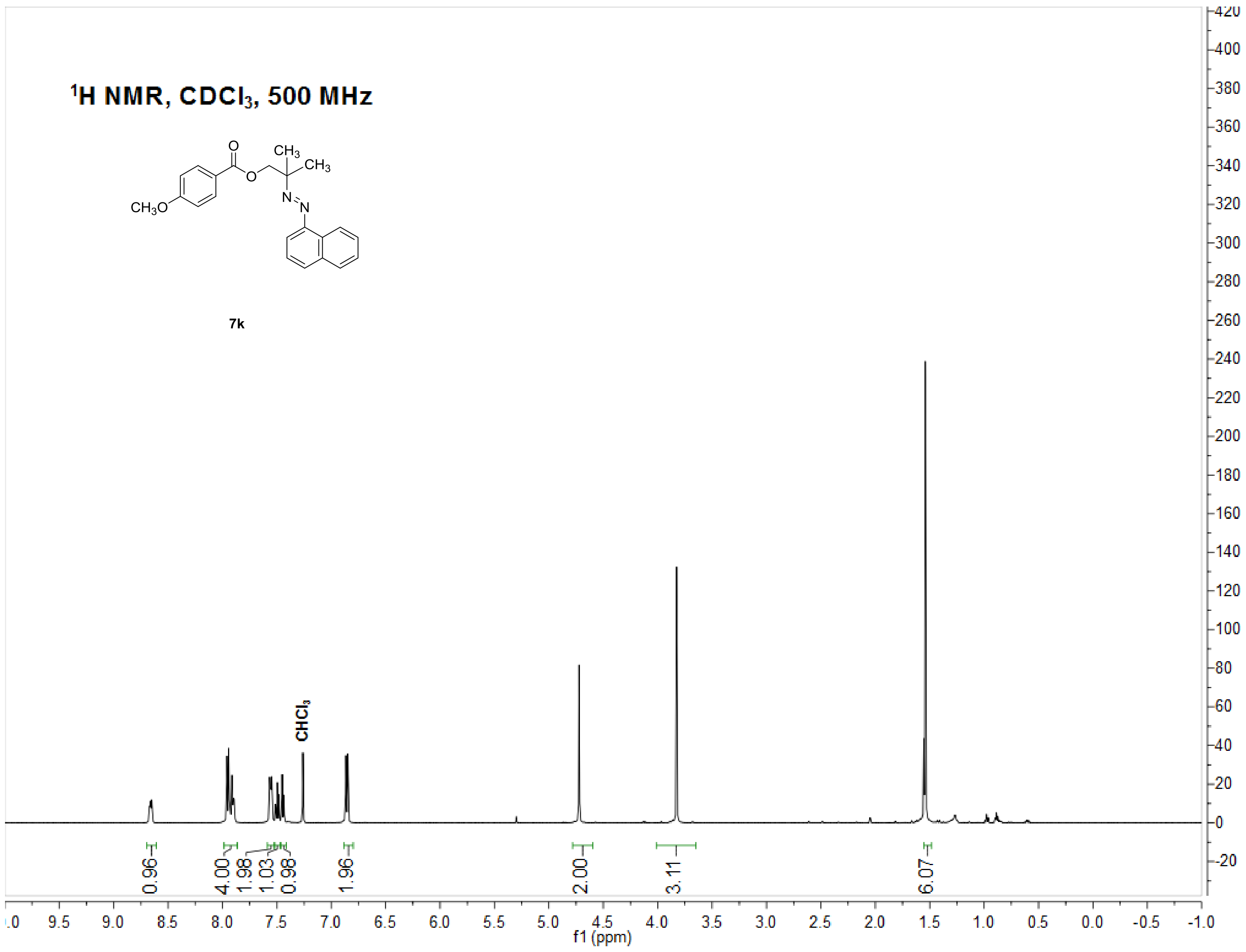

^{1}H NMR, CDCl$_3$, 500 MHz
CH$_3$O
O
O
CH$_3$
CH$_3$
N
N
7k
CHCl$_3$
0.96
4.00
1.98
1.03
0.98
1.96
2.00
3.11
6.07
f1 (ppm)

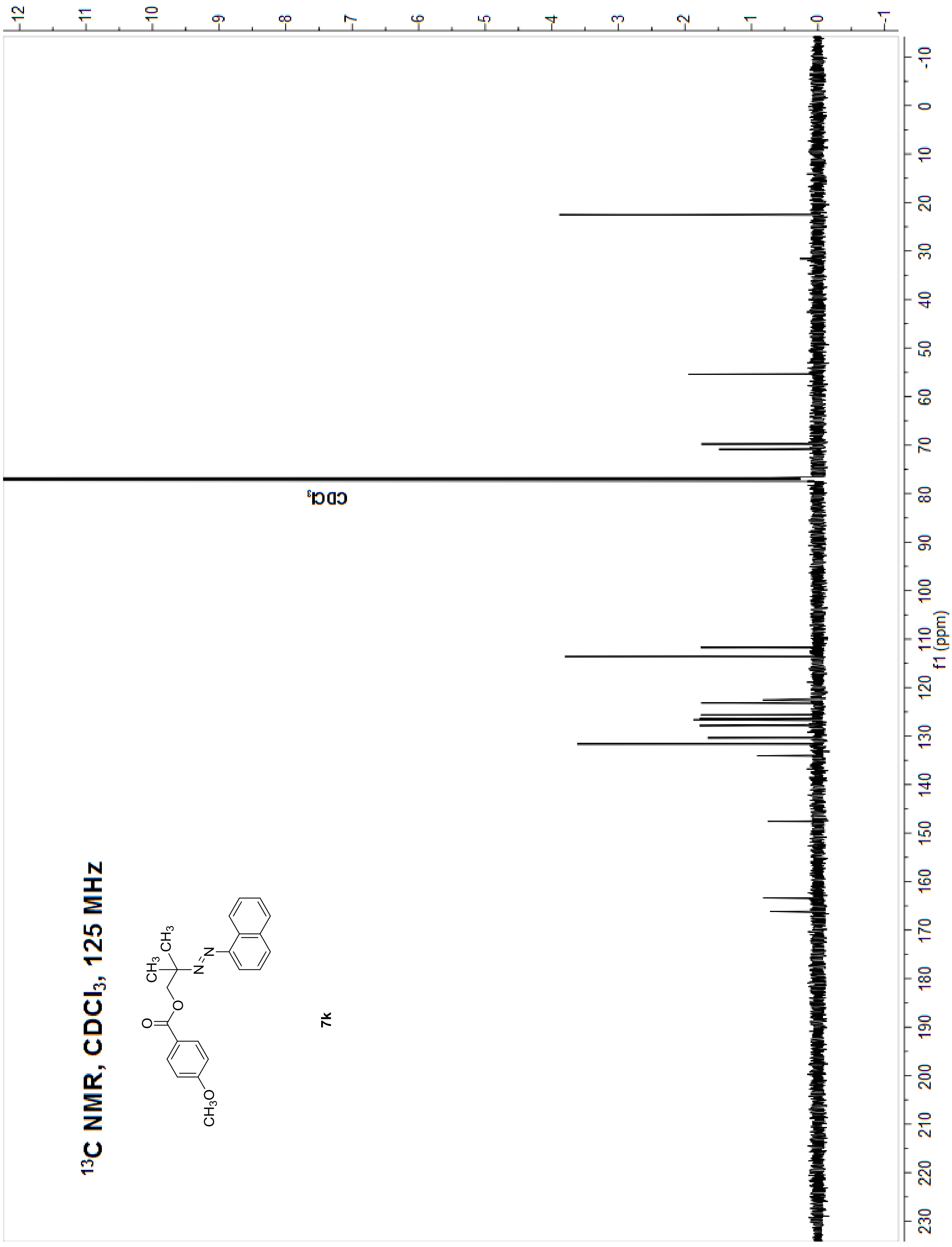

13C NMR, CDCl3, 125 MHz
7k
CDCl3
f1 (ppm)

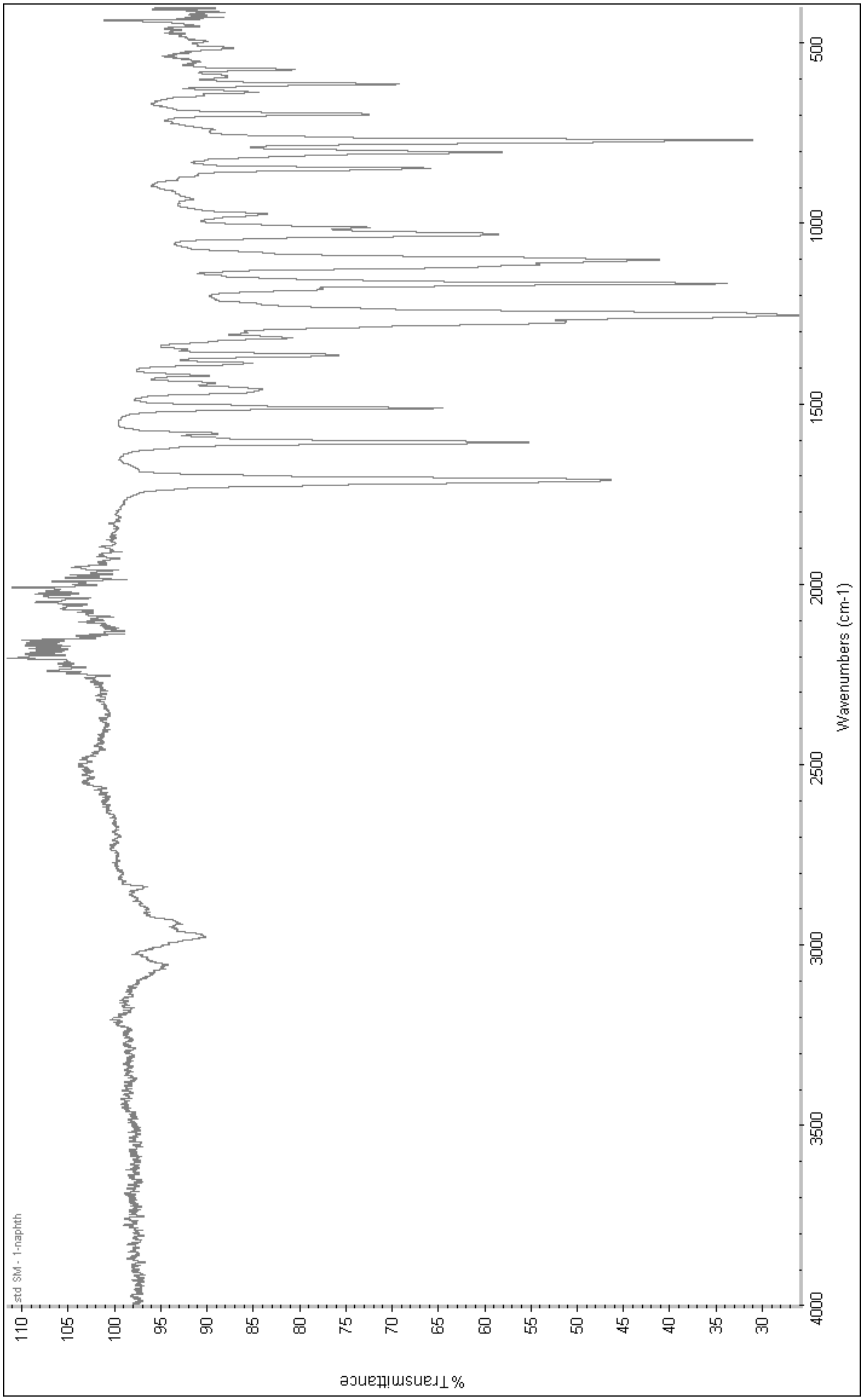
std SM - 1-naphth
%Transmittance
Wavenumbers (cm-1)

Bibliography:

[1]. Pangborn, A. B.; Giardello, M. A.; Grubbs, R. H.; Rosen, R. K.; Timmers, F. J., *Organometallics* **1996,** *15*, 1518-1520.
[2]. Edwards, G. L.; Muldoon, C. A.; Sinclair, D. J., *Tetrahedron* **1996,** *52*, 7779-7788.
[3]. Brookhart, M.; Lamanna, W.; Pinhas, A. R., *Organometallics* **1983,** *2*, 638-649.
[4]. Gaspar, B.; Carreira, E. M., *J. Am. Chem. Soc.* **2009,** *131*, 13214-13215.
[5]. King, S. M.; Ma, X.; Herzon, S. B., *J. Am. Chem. Soc.* **2014,** *136*, 6884-6887.
[6]. Corey, E. J.; Guzman-Perez, A.; Noe, M. C., *J. Am. Chem. Soc.* **1995,** *117*, 10805-10816.
[7]. Debnar, T.; Wang, T.; Menche, D., *Org. Lett.* **2013,** *15*, 2774-2777.
[8]. Poshkus, A. C.; Herweh, J. E.; Magnotta, F. A., *J. Org. Chem.* **1963,** *28*, 2766-2769.
[9]. Chen, D.-W.; Chen, Z.-C., *Tetrahedron Lett.* **1994,** *35*, 7637-7638.
[10]. Schütt, C.; Heitmann, G.; Wendler, T.; Krahwinkel, B.; Herges, R., *J. Org. Chem.* **2016,** *81*, 1206-1215.
[11]. Li, Z.; Ip, F. C. F.; Ip, N. Y.; Tong, R., *Chem. – Eur. J.* **2015,** *21*, 11152-11157.
[12]. Erb, W.; Hellal, A.; Albini, M.; Rouden, J.; Blanchet, J., *Chem. – Eur. J.* **2014,** *20*, 6608-6612.
[13]. Colleville, A. P.; Horan, R. A. J.; Tomkinson, N. C. O., *Org. Process Res. Dev.* **2014,** *18*, 1128-1136.
[14]. Wang, W.; Tang, Z.; Zhang, Y.; Wang, T., *Synlett* **2010**, 804-808.
[15]. Lee, J.-I., *Bull. Korean Chem. Soc.* **2011,** *32*, 1765.
